# Preparation of 6/8/11-Amino/Chloro-Oxoisoaporphine and Group-10 Metal Complexes and Evaluation of Their in Vitro and in Vivo Antitumor Activity

**DOI:** 10.1038/srep37644

**Published:** 2016-11-29

**Authors:** Qi-Pin Qin, Jiao-Lan Qin, Ting Meng, Gui-Ai Yang, Zu-Zhuang Wei, Yan-Cheng Liu, Hong Liang, Zhen-Feng Chen

**Affiliations:** 1 State Key Laboratory for Chemistry and Molecular Engineering of Medicinal Resources, School of Chemistry and Pharmacy, Guangxi Normal University, 15 Yucai Road, Guilin 541004, P.R. China

## Abstract

A series of group-10 metal complexes **1**–**14** of oxoisoaporphine derivatives were designed and synthesized. **1**–**14** were more selectively cytotoxic to Hep-G2 cells comparing with normal liver cells. *In vitro* cytotoxicity results showed that complexes **1**–**6**, **7**, **8**, **10** and **11**, especially **3**, were telomerase inhibitors targeting c-myc, telomeric, and bcl-2 G4s and triggered cell senescence and apoptosis; they also caused telomere/DNA damage and S phase arrest. In addition, **1**–**6** also caused mitochondrial dysfunction. Notably, **3** with 6-amino substituted ligand L^a^ exhibited less side effects than **6** with 8-amino substituted ligand L^b^ and cisplatin, but similar tumor growth inhibition efficacy in BEL-7402 xenograft model. Complex **3** has the potential to be developed as an effective anticancer agent.

G4s were non-canonical secondary structures formed by DNA sequences containing consecutive runs of guanosine. They differ from other nucleic acid secondary structures and are often associated with human diseases like cancer^1^, HIV^2^, and diabetes^3^, which make the G-quadruplex a potential therapeutic target. Recently, a variety of G4s, such as c-myc, c-kit-2, bcl-2, POT1, and c-kit-1, is considered as an appealing opportunity for drugs or compounds intervention in anticancer therapy^4^,^5^,^6^,^7^,^8^,^9^,^10^.

G-quadruplex structures have recently been found in telomeres and in promoter regions of certain genes. These G-quadruplexes are characterized by particular structures, and the formation or stabilization of G-quadruplexes in these regions may be specifically regulated. In particular, it is widely accepted that G4s in the c-myc (Pu27) gene play an important regulatory role in hTERT. Small molecule compounds were developed and synthesized against these targets to regulate telomerase activity, and thereby to selectively induce cancer cell apoptosis or/and senescence^11^,^12^. In addition, several G4 gene/oncogene promoters, such as those associated with the bcl-2 gene, have been associated with cell death/apoptosis and with diseases such as neurodegeneration, autoimmune deficiencies, and cancer^13^. Thus, designing and developing G4 ligands or G4s-based inhibitors are a novel potential anticancer strategy^14^,^15^,^16^,^17^,^18^.

Telomerase plays an key role in cancer biology and telomere maintenance^19^, so the design and synthesis of efficient telomerase inhibitors is a viable strategy towards developing anticancer drugs^20^,^21^. Some G4 ligands efficiently stabilize G-quadruplex DNA, which often leads to telomerase inhibition^15^,^16^,^17^,^18^.

Among the group-10 metals, Ni is unique in its structural versatility and redox activity. In contrast, Pd and Pt complexes have relatively rigid structures. For example, a square-planar geometry is common for Pd^II^ and Pt^II^ complexes. Group-10 metals share the same square-planar geometry, and they could all have the potential to be developed as antitumor agents^22^,^23^.

In the past decade, a large number of nickel(II)^24^,^25^,^26^,^27^,^28^, palladium(II)^29^,^30^, and platinum(II)^31^,^32^,^33^,^34^,^35^,^36^,^37^ complexes have been reported to inhibit telomerase activity and to stabilize G4s^38^,^39^,^40^. However, the antitumor activity and toxicology profiles of these metal complexes are still not satisfactory. It has been reported that alkaloids are an important source of G-quadruplex ligands, and they also exhibit significant anticancer bioactivities^41^. Nevertheless, there are only a few reported metal complexes with alkaloids as G-quadruplex ligands^42^,^43^. To combine the anticancer activity of group-10 metal complexes and those of alkaloids, we synthesized group-10 metal complexes with oxoisoaporphine ligands (6-amino-oxoisoaporphine, L^a^; 8-amino-oxoisoaporphine, L^b^; 8-chloro-oxoisoaporphine, L^c^; and 10-chloro-11-amino-oxoisoaporphine, L^d^)^44^,^45^,^46^. Our findings demonstrated that complexes **1**–**6**, **7**, **8**, **10** and **11** selectively stabilized G4, inhibited telomerase activity, and exhibited remarkable *in vitro* cytotoxicity and tumor growth inhibiting activity, especially complex **3**.

## Results and Discussion

### Synthesis and Characterization

**Figure 1:**
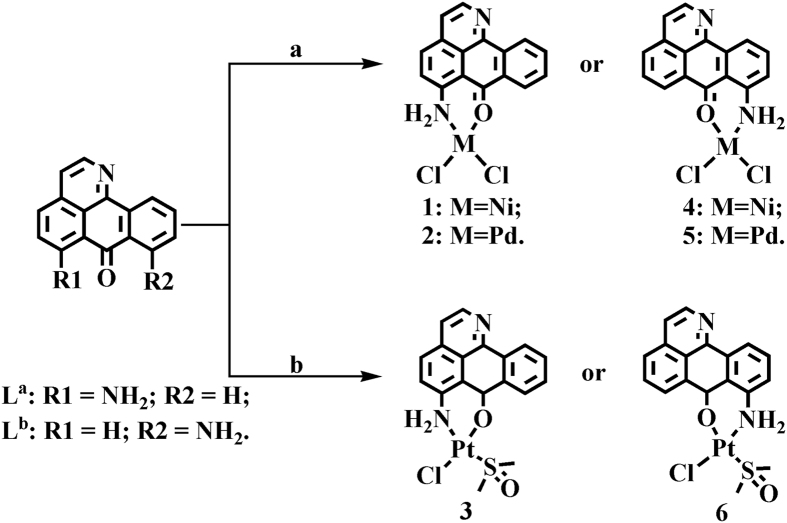
Synthetic routes for group-10 metal(II) complexes **1**–**6** of 6-amino-oxoisoaporphine (L^a^) and 8-amino-oxoisoaporphine (L^b^). Reagents and solvents are the following: (**a**) NiCl_2_ or PdCl_2_, ethanol/water (v/v = 20:1) (reflux); (**b**) *cis*-Pt(DMSO)_2_Cl_2_, ethanol/CH_3_CN (v/v = 20:1) (reflux).

**Figure 2:**
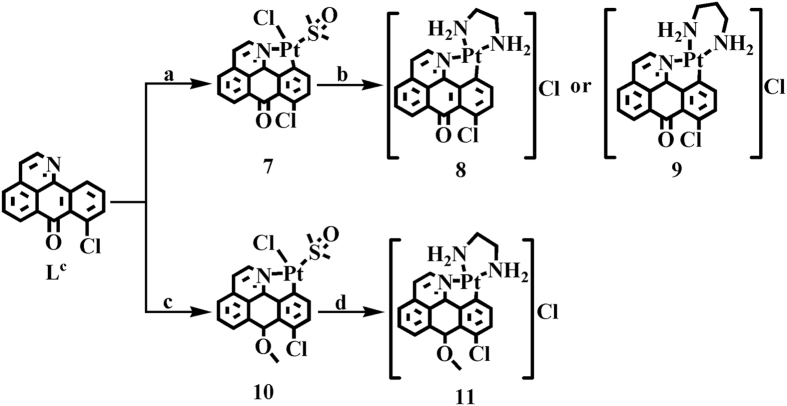
Synthetic routes for group-10 metal(II) complexes **7**–**11** of 8-chloro-oxoisoaporphine (L^c^). Reagents and solvents are the following: (**a**) *cis*-Pt(DMSO)_2_Cl_2_, ethanol/water (v/v = 20:1) (80 °C); (**b**) 1,2-ethylenediamine or 1,3-propanediamine, anhydrous ethanol (reflux); (**c**) *cis*-Pt(DMSO)_2_Cl_2_, methanol/CH_3_CN (v/v = 20:1) (80 °C); (**d**) 1,2-ethylenediamine, anhydrous ethanol (reflux).

**Figure 3:**
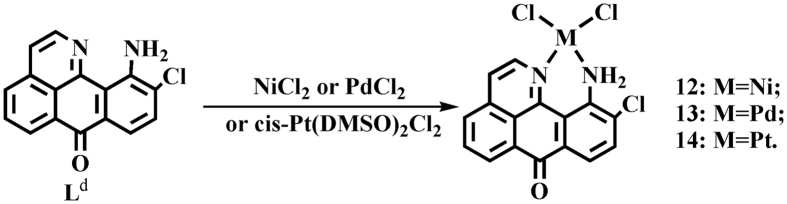
Synthetic routes for group-10 metal(II) complexes **12**–**14** of 10-chloro-11-amino-oxoisoaporphine (L^d^). Reagents and solvents are the following: NiCl_2_, PdCl_2_, or *cis*-Pt(DMSO)_2_Cl_2_, methanol/CH_3_CN/water (v/v/v = 15:5:1) (reflux).

6-amino-oxoisoaporphine, L^a^; 8-amino-oxoisoaporphine, L^b^; 8-chloro-oxoisoaporphine, L^c^; and 10-chloro-11-amino-oxoisoaporphine, L^d^; were synthesized according to reported procedures^44^,^45^,^46^. Acylation of 4-chloro phenylethylamine or β-phenylethylamine with phthalic anhydride (or 3-chloro-phthalic anhydride) gave phenylethylphthalimide and its derivatives. The product was heated in sodium chloride and anhydrous aluminum chloride (1:5) at 180.0–220.0 °C for 3.0 h to yield *o*-isoquinolin-2-ylbenzoic acid or its derivatives. Cyclization of *o*-isoquinolin-2-ylbenzoic acid or its derivatives in concentrated sulfuric acid at 230.0 °C for 3.0 h afforded 6/8/10-chloro-oxoisoaporphine in 30.0–35.0% yield. 6/8-amino-oxoisoaporphine (L^a^ and L^b^) was obtained by treating 6-chloro-oxoisoaporphine or 8-chloro-oxoisoaporphine (L^c^) with concentrated ammonium hydroxide (50.0 mL) and stirring at 185.0 °C for 24.0 h in the reaction vessel. 10-chloro-11-nitro-oxoisoaporphine was obtained by nitration of 10-chloro-oxoisoaporphine with fuming nitric acid in the presence of concentrated sulfuric acid. Reaction of 10-chloro-11-nitro-oxoisoaporphine with Na_2_S yielded 10-chloro-11-amino-oxoisoaporphine (L^d^) in 38.0% yield. Group-10 metal complexes [Ni(L^a^)Cl_2_] (**1**), [Pd(L^a^)Cl_2_] (**2**), [Pt(L^a^)(DMSO)Cl]·H_2_O (**3**), [Ni(L^b^)Cl_2_] (**4**), [Pd(L^b^)Cl_2_] (**5**), [Pt(L^b^)(DMSO)Cl]·H_2_O (**6**), [Pt(L^c^)(DMSO)Cl] (**7**), [Pt(L^c^)(en)]Cl (**8**) (en=1,2-ethylenediamine), [Pt(L^c^)(pn)]Cl (**9**) (pn=1,3-propanediamine), [Pt(L^c′^)(DMSO)Cl] (**10**), [Pt(L^c′^)(en)]Cl (**11**) (en=1,2-ethylenediamine), [Ni(L^c^)Cl_2_] (**12**), [Pd(L^c^)Cl_2_] (**13**) and [Pt(L^d^)Cl_2_] (**14**), were synthesized by the reactions of 6-amino-oxoisoaporphine (L^a^), 8-amino-oxoisoaporphine (L^b^), 8-chloro-oxoisoaporphine (L^c^) and 10-chloro-11-amino-oxoisoaporphine (L^d^) with NiCl_2_, PdCl_2_ and *cis*-[PtCl_2_(DMSO)_2_] under refluxing conditions, respectively, as depicted in Figs 1, 2 and 3. As shown in Figs 1, 2, 3 and S1–S65, the structures of ligands and their complexes **1**–**14** were characterized by IR, single crystal X-ray diffraction analysis, ESI-MS, NMR, and elemental analysis. Their metal centers are four-coordinate and approximately square planar.

### Crystal Structures

**Figure 4:**
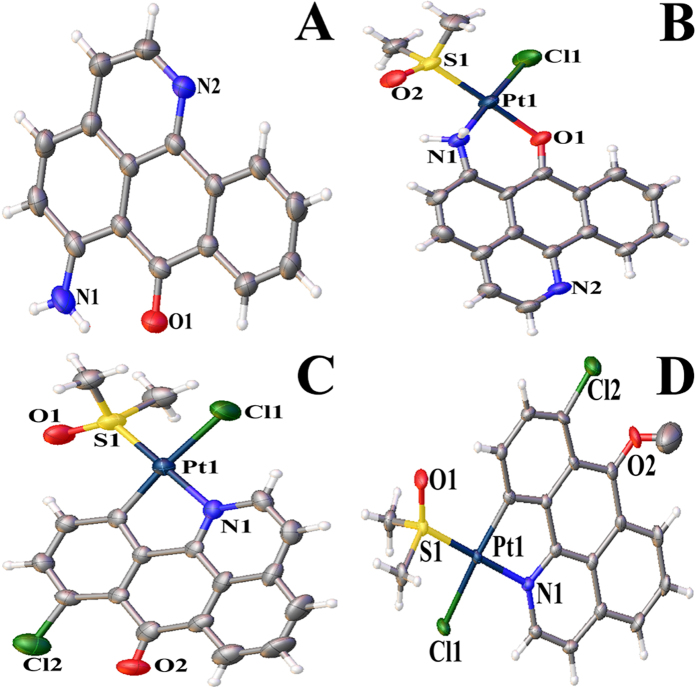
The ORTEP drawings of L^a^ (**A**), complexes 3 (**B**), 7 (**C**) and 10 (**D**) showing atom labeling.

The details of the structural refinement parameters and the crystallographic data for ligand L^a^, complexes **3**, **7** and **10** are summarized in Tables S2, S4, S6 and S8 (Supporting Information), and the selected bond lengths are tabulated in Tables S1, S3, S5 and S7 (Supporting Information). The structure of ligand L^a^ is similar to that 6-hydroxyloxoisoaporphine, 1-azabenzanthrone and 10-chloro-1-azabenzanthrone^43^, due to the planar structure (Fig. 4). As shown in Fig. 4, the central Pt(II) in complex **3** was four-coordinate with one bidentate chelating planar ligand N^O-L^a^, one chloride ligand, and one DMSO ligand, adopting an approximately square planar geometry. Furthermore, as shown in Fig. 4, complexes **7** and **10** are quite close to 1-azabenzanthrone and 6-hydroxyloxoisoaporphine platinum(II) complexes (Pt**1** and Pt**2)** in structure with an organometallic C-Pt bond^43^. Such a feature may affect their cytotoxicities and metal DNA binding properties.

### Stability of complexes 1–14 in Solution

The stability of complexes **1**–**14** in TBS (pH 7.35, 10 mM Tris-KCl-HCl buffer solution, 1% DMSO) was assessed by UV-Vis spectroscopy. Figures S66 and S67 showed no obvious spectral changes in the UV-Vis spectra of complexes **1**–**14** after 24 h, suggesting the compounds were stable in TBS. In addition, as shown in Fig. S68, after 24 h at room temperature, complexes **1**–**14** were also stable in DMSO for 24 h as shown by HPLC experiments.

### Cytotoxicity

The cytotoxic activity of the L^a^–L^d^ and complexes **1**–**14** were tested against a panel of cell lines such as Hep-G2, NCI-H460, SK-OV-3, HCT-8, BEL-7402 cells, and one normal cell line, HL-7702, and cell viability was determined using the MTT assay. Cisplatin was used as the positive control. The inhibitory rates of L^a^–L^d^ and complexes **1**–**14** are listed in Table S9. The growth inhibitory rates of complexes **1**–**14** were higher than that of PdCl_2,_ en, NiCl_2_, pn, L^a^–L^d^, and cis-Pt(DMSO)_2_Cl_2_ at 20.0 *μ*M for 48 h, which were all relatively weak against normal HL-7702 cells. Moreover, the compounds affected the NCI-H460, Hep-G2, HCT-8, BEL-7402, and SK-OV-3 cells in a dose-dependent manner, compared with cisplatin, PdCl_2,_ en, NiCl_2_, pn, and cis-Pt(DMSO)_2_Cl_2_. The IC_50_ values demonstrated that the ligands L^a^–L^d^ and their complexes **1**–**14** were active against Hep-G2, SK-OV-3, BEL-7402, NCI-H460 and HCT-8 cell lines and that they possess great selectivity towards Hep-G2 cells as compared to other human tumor cell lines. Thus, complexes **1**–**14** were more specific against a particular cancer cell (Hep-G2 cells). The IC_50_ values for complexes **1**–**14** against Hep-G2 cells were 8.24, 15.22, 4.61, 18.16, 28.09, 14.25, 16.41, 10.15, 15.88, 12.18, 6.49, 28.76, 33.54 and 22.77 *μ*M, respectively. Table 1 showed that **1**–**14** exhibited significantly enhanced cell cytotoxicity toward five tested human cancer cells as compared to L^a^–L^d^. In particular, the cytotoxicity of **3** was 2.1 times higher than that of cisplatin. Against Hep-G2, BEL-7402, SK-OV-3, HCT-8 and NCI-H460 cells, **3** showed higher cytotoxicity than L^a^–L^d^, **1**, **2, 4**–**14**, and cisplatin. As shown in Table S9 (Supporting Information) and Table 1, **1**–**14** displayed a synergistic effect as compared with the metal alone and the free corresponding 6-amino-oxoisoaporphine (L^a^), 8-amino-oxoisoaporphine (L^b^), 8-chloro-oxoisoaporphine (L^c^) and 10-chloro-11-amino-oxoisoaporphine (L^d^) ligands, respectively. In general, except for the Hep-G2 cells, the *in vitro* cytotoxicity of the group-10 metal complexes and L^a^–L^d^ against the tested tumor cells followed the order of **1** > **4** > **12** > L^d^ > L^a^ > L^b^, **2** > **5** > **13** > L^d^ > L^a^ > L^b^ and **3** > **11** > **8** > **10** > **6** > **9** > **7** > L^c^ > L^a^ > L^b^ (or **3** > **1** > **2** > L^a^, **6** > **4** > **5** > L^b^, **11** > **10** > **8** > **9** > **7** > L^c^ and **14** > **12** > **13** > L^d^). Compared with the 6-hydroxyl-oxoisoaporphine organoplatinum(II) complex, the 6-amino-oxoisoaporphine platinum(II) complex **3** exhibited greater cytotoxicity against Hep-G2, SK-OV-3, NCI-H460 tumor cell lines^43^. In the case of SK-OV-3 and NCI-H460 cells, the 8-chloro substituent on complexes **7** and **10** led to greater cytotoxicity than that of 1-azabenzanthrone platinum(II) complex Pt**1**^43^. This demonstrated the importance and the key role of halogen (e.g. Cl) substitution of 1-azabenzanthrone or oxoisoaporphine^47^. At the same time, L^c^ exhibited greater cytotoxicity than 1-azabenzanthrone did. Notably, complex **3** exhibited a broad spectrum of inhibition against five selected human cancer cells with IC_50_ values ranging from 4.61 to 14.17 *μ*M. This indicated greater cytotoxic effects of complexes **7** and **10** on cancer cells. One of the features of complexes **3**, **7**, and **10** was the different substitution positions of the chloro or amino group on the phenyl ring of oxoisoaporphine or 1-azabenzanthrone, which may be contribute to their various cytotoxicities and differing antitumor activities. The significantly improved biological behavior by complex **3** may be correlated with the substituted 6-amino in ligand L^a^. This was similar to the behavior of the 5-amino-quinolone antimicrobial agents, which were reported to be more cytotoxic than the 5-substituted quinolone antimicrobial agents (e.g. substituent = CH_3_, CH_2_CH_3_, F, Cl and OH, etc.)^48^. On the other hand, compared with complex **3**, the organometallic platinum(II) complexes **7** and **10** with C-Pt bond offered different possibilities for exploration as anticancer agents due to the large structural differences between different ligands (e.g. [(η^6^-arene)Ru(en)(Cl)]^+^, [(η^6^-Bip)Ru(en)Cl]PF_6_, Cp_2_TiCl_2_, oxoisoaporphine platinum(II) complexes Pt**1** and Pt**2,** 6-amino-oxoisoaporphine and 8-chloro-oxoisoaporphine platinum(II) complexes, etc.) and different bonding modes (e.g. π-π-coordination, M-C multiple bonds)^43^,^49^. This might lead to different DNA binding modes (**3** and **10** interacted with G4-DNA by covalent binding and by π-π stacking, respectively) and the weaker binding abilities of complexes **7** and **10** to G4 in comparison with complex **3**^49^. Therefore, complex **3** with the 6-amino substitution in ligand L^a^ exhibited higher selectivity for G4, stronger telomerase inhibition ability by directly down-regulating Pu27 G4 (the c-myc promoter/G4), and improved induction of cell apoptosis in Hep-G2 cells than complexes **7** and **10** did. Compounds **3**, **6**, **10** and **11** showed the highest *in vitro* cytotoxicity, therefore we selected **3** and **6** for investigating their cytotoxic mechanisms and *in vivo* tumor growth inhibition.

### Cellular Uptake

**Figure 5:**
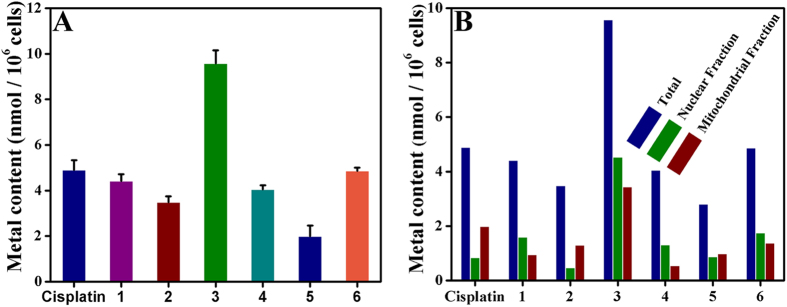
Hep-G2 cells were treated with cisplatin (10 *μ*M), 1 (8 *μ*M), 2 (15 *μ*M), 3 (5 *μ*M), 4 (18 *μ*M), 5 (28 *μ*M) and 6 (14 *μ*M) at 37 °C for 24 h, respectively.

In order to understand the uptake and distribution of cisplatin, complexes **1**–**6**, **7**, **8**, **10** and **11** in Hep-G2 cells, an ICP-MS assay was performed to quantify the amount of metal (Pt, Ni or Pd) taken up by the cells. As shown in Figs 5A and S69A, treatment of Hep-G2 cells with complexes **1** (8 *μ*M), **2** (15 μM), **3** (5 μM), **4** (18 μM), cisplatin (10 μM), **5** (28 *μ*M), **6** (14 *μ*M), **7** (16 *μ*M), **8** (10 *μ*M), **10** (12 *μ*M), and **11** (6 *μ*M) for 24 h led to a substantial increase in the cellular metal (Pt, Ni or Pd) concentrations as compared with untreated cells, demonstrating that each complex was readily internalized after 24 h. Treatment of cells with compound **3** ((9.57 ± 0.58 nmol platinum)/10^6^ cells) led to notably higher cellular accumulation of metal (Pt) than treatment with cisplatin ((4.89 ± 0.43 nmol platinum)/10^6^ cells), **1** ((4.41 ± 0.30 nmol nickel)/10^6^ cells), **2** ((3.48 ± 0.26 nmol palladium)/10^6^ cells), **4** ((4.05 ± 0.18 nmol nickel)/10^6^ cells), **5** ((1.99 ± 0.47 nmol palladium)/10^6^ cells), **6** ((4.86 ± 0.14 nmol platinum)/10^6^ cells), **7** ((2.94 ± 0.35 nmol platinum)/10^6^ cells), **8** ((3.59 ± 0.25 nmol platinum)/10^6^ cells), **10** ((3.28 ± 0.14 nmol platinum)/10^6^ cells) or **11** ((5.55 ± 0.11 nmol platinum)/10^6^ cells).

The distributions of metal (cisplatin, complexes **1**–**6, 7**, **8**, **10** and **11**) in the nuclear fraction and mitochondrial fractions of Hep-G2 cells were studied after exposure of Hep-G2 cells to **1**–**6**, **7**, **8**, **10**, **11** and cisplatin for 24 h according to our previous reports^43^. As shown in Figs S69B and 5B, complexes **1**, **3**, **4**, and **6** were accumulated to a large extent in the mitochondrial fraction and the nuclear fraction in Hep-G2 cells, whereas it only accumulated into the nuclear fraction when cells were treated with complex **7**, **8**, **10** or **11**. In contrast, complexes **2**, **5** and cisplatin were mainly accumulated in the mitochondrial fraction. Taken together, their difference of metal (Pt, Ni or Pd) distribution for complexes **1**–**6**, **7**, **8**, **10**, **11** and cisplatin could be attributed to their differences in cytotoxicity and the apoptotic pathways in Hep-G2 cells.

### Selectivity for Binding with G4 by Complexes

**Figure 6:**
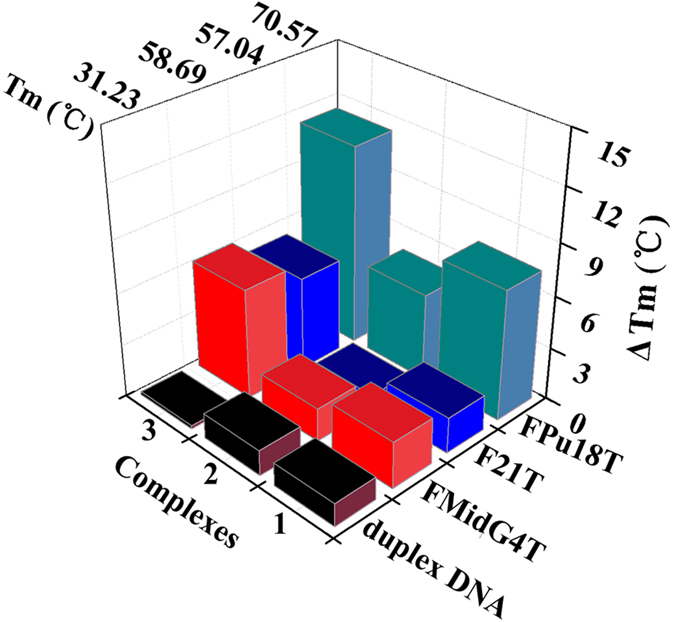
ΔTm data (°C) of 1.0 *μ*M duplex DNA (F32T + H20M DNA), F21T (HTG21 G4), FMidG4T (Pu39 G4) and FPu18T (c-myc/Pu27 G4) G4s after treated with complexes 1–3 (1.0 *μ*M) evaluated by RT-PCR.

The formation and stabilization of duplex DNA and G4 after treatment with complexes **1–6**, **7** and **10** were first examined by various spectroscopic methods. As shown in Figs S70 and S71, FID curves (Supporting Information) were obtained by plotting the percent displacement of TO (thiazole orange) against the concentration of complexes **1**–**6**, **7** and **10**. The concentrations of complexes **1**–**6**, **7** and **10** required to decrease the fluorescence by 50%, reflecting binding to G-quadruplex (^G4^DC_50_) or duplex (^ds26^DC_50_ or/and ^ctDNA^DC_50_) structures, are reported in Table 2. Complexes **1**–**6**, **7** and **10**, and especially **3**, appear to be strong binders of HTG21 and Pu27 G-quadruplex structures with a ^G4^DC_50_ of 0.75–70.83 *μ*M, whereas **1**–**6**, **7** and **10** exhibit the poor affinity for the ctDNA structures. Overall, the FID assay indicated that the selectivity of complexes **1**–**6**, **7** and **10** for Pu27 and HTG21 G4s over duplex DNA (ctDNA) was generally moderate, and they were also the most efficient TO-displacers, with a ^ctDNA^DC_50_/^G4^DC_50_ ratio (or Est. Sel.)^43^,^50^ within the range of 1.67 and 130.49 fold, and 1.41 and 64.81 fold, respectively. As expected, complex **3** showed higher selectivity for quadruplex-DNA than **1**, **2**, **4–6, 7** and **10** did. Because of the weak binding to G4-DNA by **4–6, 7** and **10** in the FID assay, only **1**–**3** were selected for G-quadruplex and duplex DNA binding studies using FRET and CD spectroscopy. Similar trends were observed in the FRET-melting assay. As shown in Fig. 6 and Figs S72–S74, the ΔT_m_ values were 2.06, 0.80 and 5.39 °C for F21T, 7.48, 4.98 and 11.50 °C for FPu18T, and 2.68, 1.89 and 6.32 °C for FMidG4T for 1.0 *μ*M of complexes **1**, **2** and **3**, respectively. In contrast, the ΔT_m_ values were 1.33, 1.45 and 0.19 °C (F32T + H20M) DNA (or duplex DNA) for complexes **1**, **2** and **3** treated were under the same conditions, respectively, suggesting that complexes **1**, **2** and **3** exerted a stronger stabilizing effect on HTG21 (telomeric), FPu18T (Pu39, bcl-2) and Pu27 (c-myc) G4s over (F32T + H20M) DNA/duplex DNA than complexes **1** and **2** did (Figs S72–S76). In addition, the ESI-MS spectra of Pu27 G-quadruplex treated with complexes **3** and **6** indicated that complexes **3** and **6** were able to form adducts with Pu27 G4 {**3** + Pu27 G4: ESI-MS: m/z 9290.47 [Pu27 G4 + Pt(L^a^)(DMSO) + CH_3_CN + H_2_O-2H]^−^ (Calcd. m/z 9290.41); **6** + Pu27 G4: ESI-MS: m/z 9267.58 [Pu27 G4 + Pt(L^b^)(DMSO)Cl-H]^−^ (Calcd. m/z 9267.62); Pu27 G4: ESI-MS: m/z 8713.53 [Pu27-H]^−^ (Calcd. m/z 8713.49)}, as shown in Fig S77. Moreover, the ESI-MS spectra of Pu27 G-quadruplex treated with complex **10** indicated that it was able to form adducts with Pu27 G4 {**10** + Pu27 G4: ESI-MS: m/z 9051.91 [Pu27 G4 + Pt(L^c′^)(DMSO)Cl + CH_3_CN-H]^−^ (Calcd. m/z 9051.87); Pu27 G4: ESI-MS: m/z 8423.38 [Pu27-H]^−^ (Calcd. m/z 8423.30)}. This finding demonstrated that complex **10** interacted with Pu27 G4 DNA most likely by π-π stacking, which was different from 6-amino-oxoisoaporphine platinum(II) complex **3** and the oxoisoaporphine platinum(II) complexes Pt**1** and Pt**2**^43^. Comparison of the ESI-MS spectra before and after complex treated showed that **3** was coordinated to the guanine of G4-DNA (Fig. S77), whereas **6** and **10** might have been interacting with the π-π stacking of the G-quadruplex^10^,^33^. FID and ESI-MS spectra experiments showed that the organometallic platinum(II) complexes **7** and **10** with a direct metal-carbon bond offers different possibilities for exploration as anticancer agents due to the large structural differentiation between the different ligands (e.g. [(η^6^-arene)Ru(en)(Cl)]^+^, [(η^6^-Bip)Ru(en)Cl]PF_6_, Cp_2_TiCl_2_, oxoisoaporphine platinum(II) complexes Pt**1** and Pt**2,** 6-amino-oxoisoaporphine and 8-chloro-oxoisoaporphine platinum(II) complexes, etc.) and their different bonding modes (e.g. π-π-coordination, M-C multiple bonds)^43^,^49^. This might lead to their different DNA binding modes (**3** and **10** interacted with G4-DNA by the covalent binding and π-π stacking, respectively) and weaker binding abilities of complexes **7** and **10** to G4 in comparison with complex **3**^49^. Furthermore, the CD spectra revealed positive peaks near 265 and 300 nm, as shown in Fig. S78, which indicated that the Pu27 sequence in TBS contained both parallel and antiparallel G4s before treated with the complex^43^,^51^,^52^. After treatment with compounds **1**–**3**, the CD intensity of the positive peak near 265 nm decreased, which revealed a structural transformation from a mixture of parallel and antiparallel G4s to an antiparallel G-quadruplex. However, the CD spectrum Pu27 G4 in the presence of complexes **1**–**3** suggested a mixture G-quadruplexes DNA of parallel and antiparallel G-quadruplex conformations, likely because **1**–**3** preferentially folded the Pu27 G4 DNA into a mixed-type or hybrid G4 structure in these experimental conditions^51^,^52^. In all, it is clear that the parallel and antiparallel (mixed-type or hybrid G4 structure) conformation of the G4 DNA after treatment with **1**–**3** was favorable for the binding of Pu27 G4 in TBS, which also suggested that complexes **1**–**3** might interact with the loops and grooves of G-quadruplex DNA^10^,^24^,^33^,^38^,^51^,^52^. In addition, in the presence of K^+^, the CD data indicated that HTG21, and Pu39 DNAs (sequence) exhibited a mixture G-quadruplex DNA in parallel and antiparallel G-quadruplex conformations^24^,^38^,^51^,^52^, similar to **1**–**3** treated Pu27 G4 (Figs S78–S80 and Table S10). In general, complex **3** was considered a highly selective ligand for HTG21 (telomeric), FPu18T (Pu39, bcl-2) and Pu27 (c-myc) G4s.

### Telomere Damage

**Figure 7:**
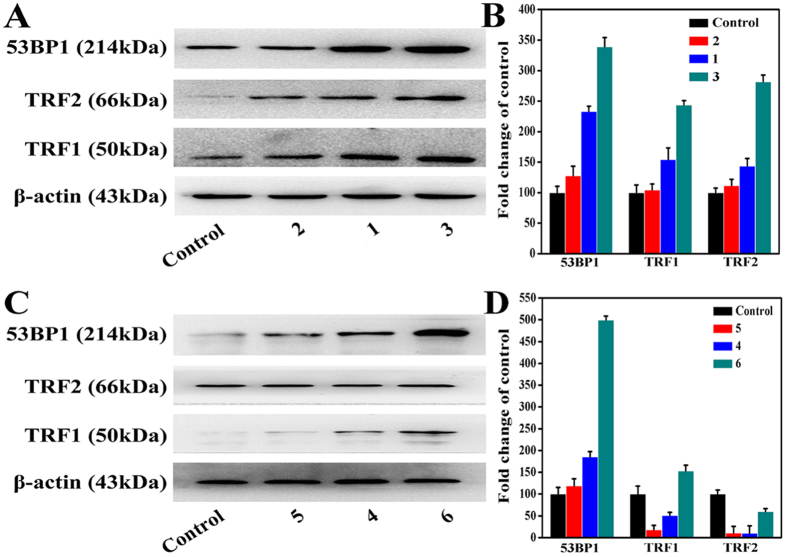
The expression of TRF2, 53BP1, and TRF1 in Hep-G2 cells after treated with complexes 1 (8 *μ*M), 2 (15 *μ*M), 3 (5 *μ*M), 4 (18 *μ*M), **5** (28 *μ*M) and 6 (14 *μ*M) for 24 h was analyzed by Western blot, respectively. (**A**,**C**) TRF2, 53BP1, and TRF1 protein levels in Hep-G2 cells were analyzed by western blot. (**B**,**D**) The whole-cell extracts were prepared and analyzed by Western blot analysis using antibodies against TRF2, 53BP1, and TRF1. The same blots were stripped and re-probed with a β-actin antibody to show equal protein loading. Western blot bands from three independent measurements were quantified with Image J. in (**B**,**D**).

Previous studies have demonstrated that TRF1 and TRF2 regulate the two major functions of telomeres^53^: TRF1 controls telomere length by inhibiting telomerase at chromosome ends and perhaps by inhibiting C-strand synthesis^54^, and TRF2 controls chromosome end protection^55^, which could induce telomere damage and DNA damage by its effect on ATM, 53BP1, *γ*-H2AX, Rif1, and Mre11, etc^56^,^57^,^58^. Herein, to assess the effects of complexes **1** (8 *μ*M), **2** (15 *μ*M), **3** (5 *μ*M), **4** (18 *μ*M), **5** (28 *μ*M) and **6** (14 *μ*M) on telomere dysfunction in Hep-G2 cells, TRF1, 53BP1, and TRF2 telomere damage factors was analyzed by Western blot (Fig. 7). Figure 7 illustrated that complexes **1** (8 *μ*M), **2** (15 *μ*M), **3** (5 *μ*M), **4** (18 *μ*M), **5** (28 *μ*M) and **6** (14 *μ*M) led to a 33.27 ± 8.32%, 154.42 ± 18.77% and 143.74 ± 12.32%, 127.90 ± 15.65%, 104.34 ± 9.65% and 111.94 ± 10.06%, 339.14 ± 15.06%, 243.69 ± 7.23% and 281.57 ± 11.06%, 185.36 ± 12.14%, 50.97 ± 7.04% and 9.78 ± 17.02%, 118.87 ± 16.07%, 18.12 ± 10.13% and 10.54 ± 15.38%, and 499.63 ± 9.11%, 153.05 ± 13.35% and 60.01 ± 6.35% increase of 53BP1, TRF1 and TRF2 protein expression levels, respectively, demonstrating that their abilities to induce telomere damage^53^ were 43.73%, 28.69%, 65.33%, 30.77%, 23.73% and 42.65%, respectively. The results suggested that the selectivity of complex **3** for interacting with telomeric regions was higher than that of complexes **1**, **2** and **4–6**, and the different selectivities for telomeric regions were in the following order: **3** > **1** > **6** > **4** > **2** > **5 (**or **1** > **4**, **2** > **5** and **3** > **6)**.

### Senescence Induction

**Figure 8:**
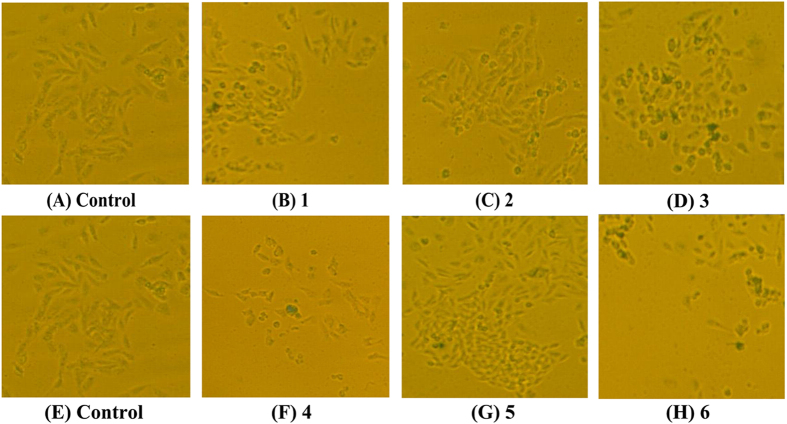
Senescence induced by complexes 1–6 (0.5 *μ*M) or 0.1% DMSO (control) on Hep-G2 cells for 7 days, and examined by Fluorescence microscope (Nikon Te2000 microscope, 100×) with stained β-galactosidase.

Since complexes **1**–**6** were found to be one most promising complexes as c-myc, telomeric, and bcl-2 G4 ligands/G4s-based inhibitors and a telomerase inhibitor in all studies, the following cellular senescence were carried out. These giant/flat cells^59^ also stained positively for SA-*β*-Gal activity after continuous treatment of Hep-G2 cells with 0.5 *μ*M complexes **1**–**6** for 7 days (Fig. 8), respectively, suggesting complexes **1**–**6** can induce accelerated senescence in Hep-G2 cells. In addition, these results also indicated that **3**, featuring a 6-amino substitution in the ligand L^a^, induced cell senescence more readily than complexes **1**, **2** and **4–6**.

### Effects of Tested Complexes on hTERT and c-myc in Hep-G2 Cells

**Figure 9:**
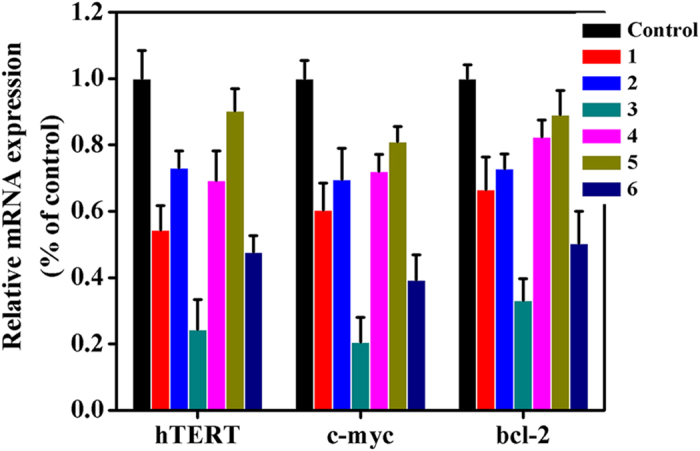
Complexes 1 (8 *μ*M), 2 (15 *μ*M), 3 (5 *μ*M), 4 (18 *μ*M), 5 (28 *μ*M) and 6 (14 *μ*M) effect on hTERT, bcl-2 and c-myc mRNA expression levels in Hep-G2 cells.

**Figure 10:**
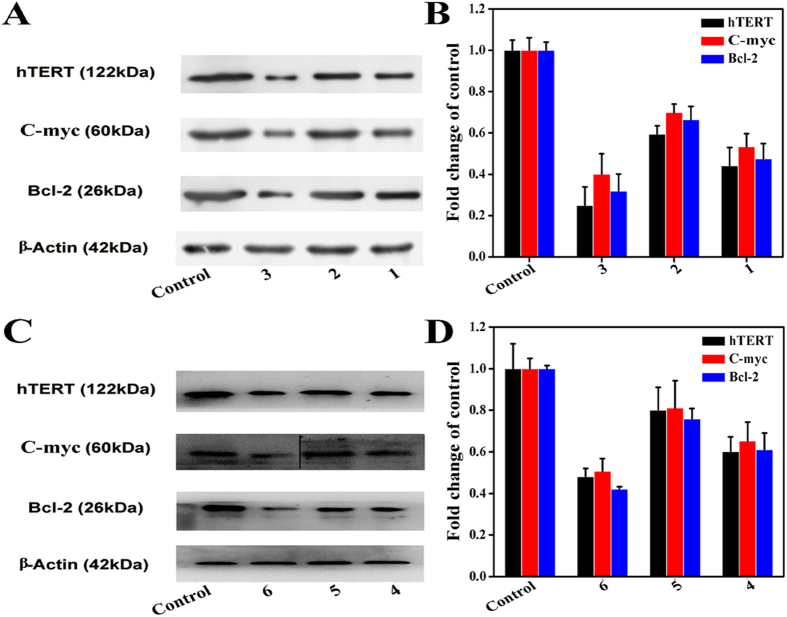
Western blot analysis of hTERT, bcl-2, and c-myc in Hep-G2 cells after 24 h incubation with complexes 1 (8 *μ*M), 2 (15 *μ*M), 3 (5 *μ*M), 4 (18 *μ*M), 5 (28 *μ*M) and 6 (14 *μ*M) for 24 h, respectively. (**A,C**) hTERT, bcl-2, and c-myc protein levels in Hep-G2 cells were analyzed by western blot. (**B,D**) The whole-cell extracts were prepared and analyzed by Western blot analysis using antibodies against hTERT, bcl-2, and c-myc. The same blots were stripped and reprobed with a β-actin antibody to show equal protein loading. Western blotting bands from three independent measurements were quantified with Image J. in (**B,D**).

Previous studies suggested that the hTERT gene core promoter contains two E-box binding sites of c-myc promoter region, which was also related to cell apoptosis and/or senescence^37^,^52^,^60^. Herein, we confirmed the expression levels of two genes (hTERT and c-myc) mRNA in Hep-G2 ells treated with complexes **1** (8 *μ*M), **2** (15 *μ*M), **3** (5 *μ*M), **4** (18 *μ*M), **5** (28 *μ*M), **6** (14 *μ*M), **7** (16 *μ*M), **8** (10 *μ*M), **10** (12 *μ*M), and **11** (6 *μ*M) for 24 h (Figs 9 and S81), respectively. As shown in Figs 9 and S81, the down-regulation of complex **3** on two mRNA in Hep-G2 cells was more than that of complexes **1**, **2**, **4–6**, **7**, **8**, **10** and **11**, and the results showed the different effects on hTERT and c-myc promoter in the following order: **3** > **1** > **6** > **4** > **2** > **5 (**or **1** > **4**, **2** > **5**, **3** > **6)** and **3** > **11** > **6** > **8** > **10** > 7 (or **11** > **8** > **10** > **7**). As shown in Figs 10 and S82, similar trends were observed in Hep-G2 cells by the Western blot assay.

### Transcription and Translation of bcl-2 Gene

Recent studies demonstrate that inhibition of bcl-2 expression can decrease cellular proliferation and enhance the efficacy of chemotherapy^13^. Herein, we were also interested in bcl-2 gene transcription and translation levels in Hep-G2 cells after incubation with complexes **1** (8 *μ*M), **2** (15 *μ*M), **3** (5 *μ*M), **4** (18 *μ*M), **5** (28 *μ*M), **6** (14 *μ*M), **8** (10 *μ*M) and **11** (6 *μ*M). As shown in Figs 9 and 10, S81 and S82, complexes **1** (8 *μ*M), **2** (15 *μ*M), **3** (5 *μ*M), **4** (18 *μ*M), **5** (28 *μ*M), **6** (14 *μ*M), **8** (10 *μ*M) and **11** (6 *μ*M) could down-regulate the bcl-2 gene in Hep-G2 cells, and complex **3** decreased the transcription and protein level of bcl-2 more than that of complexes **1**, **2**, **4–6**, **8** and **11**, which inhibited the expression of bcl-2 via its specific interaction with Pu39 G4^13^.

### Transfection Assay

**Figure 11:**
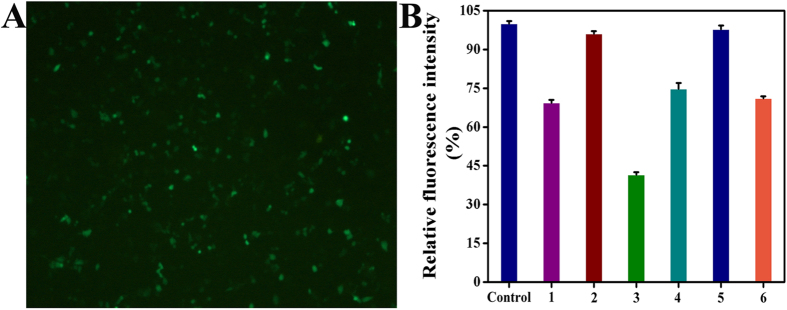
The successful transfection of 2.0 *μ*g EGFP plasmid vector (**A**) and c-myc promoter plasmid vector (**B**) in Hep-G2 cells treatment of complexes **1** (8 *μ*M), **2** (15 *μ*M), **3** (5 *μ*M), **4** (18 *μ*M), **5** (28 *μ*M) and **6** (14 *μ*M) for 24 h was examined by fluorescence microcopy or/and Multimodel Plate Reader with luciferase reporter gene assay kit, respectively.

To further demonstrate the role of the c-myc (Pu27 or c-myc G4) promoter on the telomerase and/or hTRET gene in Hep-G2 cells, transfection assays of the c-myc promoter gene vector and EGFP gene vector were performed as Chalfie, Chen, and He have reported^43^,^61^,^62^. As shown in Figs 11(A) and S83(A and C), the transfection of a EGFP gene vector was successful when Hep-G2 cells showed green fluorescence after EGFP plasmid transfection. Moreover, the Hep-G2 cells were then transfected by c-myc plasmid and treated with complexes **1** (8 *μ*M), **2** (15 *μ*M), **3** (5 *μ*M), **4** (18 *μ*M), **5** (28 *μ*M), **6** (14 *μ*M), **7** (16 *μ*M), **8** (10 *μ*M), **10** (12 *μ*M), and **11** (6 *μ*M). Consequently, they were examined under the Luciferase reporter gene assay kit and demonstrated a remarkable decrease in emission of bright green fluorescence, as shown in Figs 11(B) and S83(B and D). As a result, treatment with complex **3** (5 *μ*M) reduced the fluorescence emission by 58.5%, whereas complexes **1** (8 *μ*M), **2** (15 *μ*M), **4** (18 *μ*M), **5** (28 *μ*M), **6** (14 *μ*M), **7** (16 *μ*M), **8** (10 *μ*M), **10** (12 *μ*M), and **11** (6 *μ*M) only reduced the fluorescence emission by 30.6%, 3.9%, 58.5%, 25.3%, 2.2%, 29.0%, 7.0%, 19.8%, 15.6% and 44.1% under the same conditions, respectively. This further confirmed the superior efficacy of complex **3** (5 *μ*M) in inhibiting telomerase activity via directly down-regulating c-myc/Pu27 G4 in Hep-G2 cells, even at lower concentration. Taken together, these results demonstrated that the inhibitory effects of complex **3** in the Hep-G2 cells were more than that of **1**, **2**, **4–6**, **7**, **8**, **10** and **11**, which were in agreement with all the Western blot and RT-PCR results.

### TRAP-Silver Staining Assay

**Figure 12:**
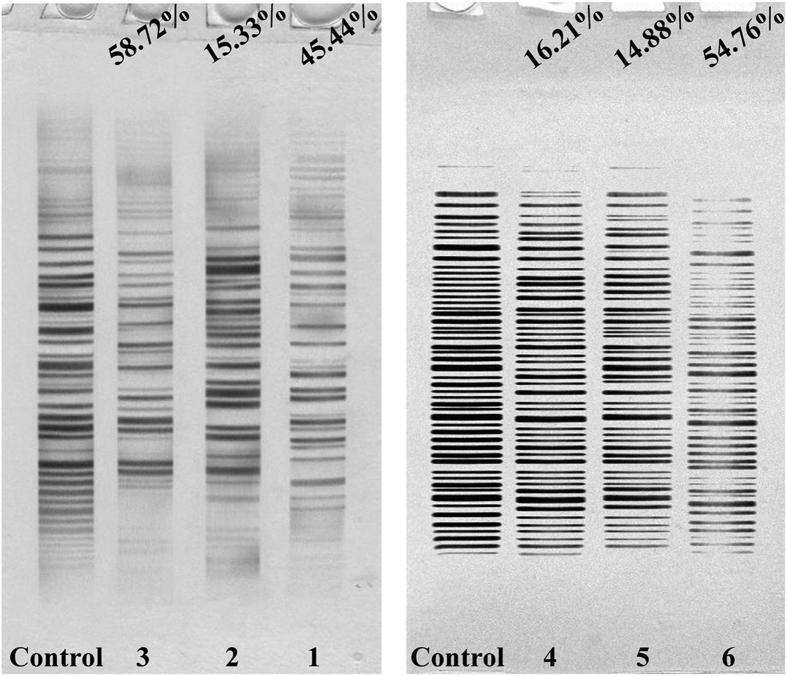
The influence of complexes 1 (8 *μ*M), 2 (15 *μ*M), 3 (5 *μ*M), 4 (18 *μ*M), 5 (28 *μ*M) and 6 (14 *μ*M) on telomerase activity in Hep-G2 cells for 24 h, respectively.

A large number of studies have demonstrated that G4 ligands, such as c-myc, telomeric, and bcl-2 G4s have been previously reported to inhibit telomerase activity and induce cell apoptosis and/or senescence^43^,^63^,^64^. Herein, it was of interest to compare the ability of complexes **1** (8 *μ*M), **2** (15 *μ*M), **3** (5 *μ*M), **4** (18 *μ*M), **5** (28 *μ*M), **6** (14 *μ*M), **7** (16 *μ*M), **8** (10 *μ*M), **10** (12 *μ*M), and **11** (6 *μ*M) on the telomerase activity as examined by the TRAP-silver staining assay. As shown in Figs 12 and S84, all the results demonstrated that the inhibitory ratio for telomerase activity induced by 5 μM of complex **3** reached 58.72%, whereas that induced by complexes **1** (8 *μ*M), **2** (15 *μ*M), **3** (5 *μ*M), **4** (18 *μ*M), **5** (28 *μ*M), **6** (14 *μ*M), **7** (16 *μ*M), **8** (10 *μ*M), **10** (12 *μ*M), and **11** (6 *μ*M) only reached 45.44%, 15.33%, 58.72%, 16.21%, 14.88%, 54.76%, 15.57%, 22.94%, 16.84% and 44.99%, respectively. This was consistent with the levels of c-myc, hTERT, and bcl-2 determined using RT-PCR, the transfection assay, and Western blotting.

### Cell Cycle

**Figure 13:**
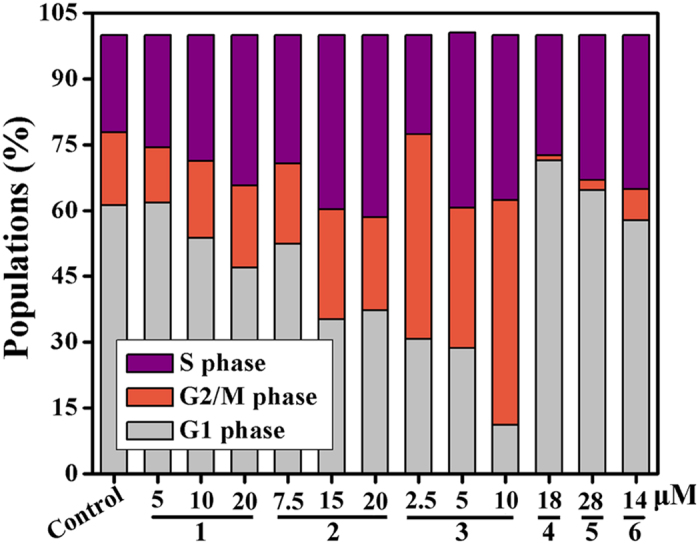
The different phase percentages of Hep-G2 cell cycle treated with complexes 1 (8 *μ*M), 2 (15 *μ*M), 3 (5 *μ*M), 4 (18 *μ*M), 5 (28 *μ*M), 6 (14 *μ*M), 8 (10 *μ*M) and 11 (6 *μ*M) for 24 h, respectively.

Many studies have demonstrated that telomerase activity is related to cell cycle arrest. Various compounds can cause S phase and/or G2-M phase arrest in human tumor cells^65^,^66^. Cell cycle analysis (Figs 13 and S85–S87) clearly showed that complexes **1**–**6**, **8** and **11** caused S phase arrest in Hep-G2 cells for 24 h, in comparison with the control cells. As shown in Figs 13 and S84–S86, S populations of 28.73%, 39.73%, 39.99%, 27.45%, 33.00%, 35.13%, 29.08% and 37.31% for complexes **1** (8 *μ*M), **2** (15 *μ*M), **3** (5 *μ*M), **4** (18 *μ*M), **5** (28 *μ*M), **6** (14 *μ*M), **8** (10 *μ*M) and **11** (6 *μ*M), respectively, were observed, as compared with an S population of 22.15% (or 17.20%) for untreated cells.

### Effect on Cell Cycle Protein Regulators

**Figure 14:**
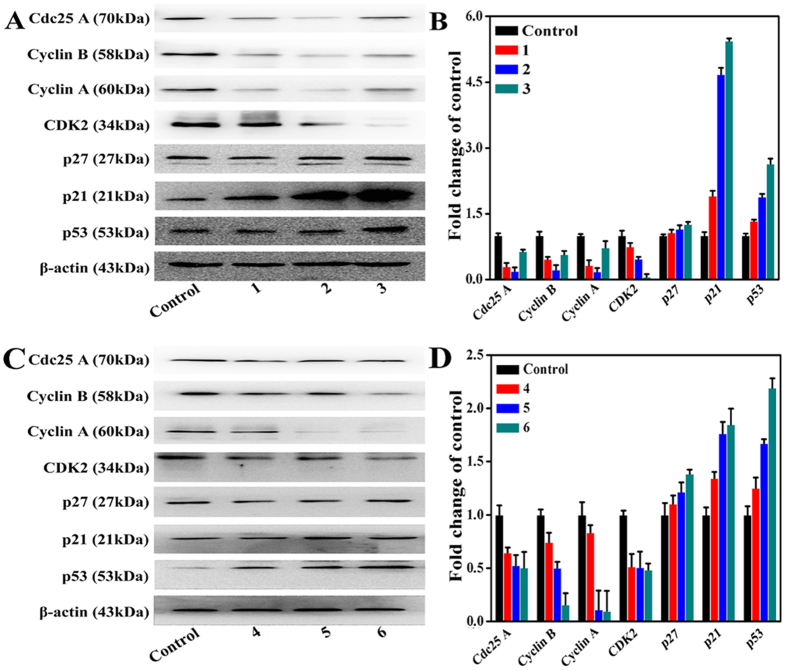
The protein levels of cell cycle protein regulators in Hep-G2 cells after treatment with complexes 1 (8 *μ*M), 2 (15 *μ*M), 3 (5 *μ*M), 4 (18 *μ*M), 5 (28 *μ*M) and 6 (14 *μ*M) for 24 h, respectively. (**A,C**) Cell cycle protein regulators protein levels in Hep-G2 cells were analyzed by western blot. (**B,D**) The whole-cell extracts were prepared and analyzed by Western blot analysis using antibodies against cell cycle protein regulators proteins. The same blots were stripped and reprobed with a β-actin antibody to show equal protein loading. Western blotting bands from three independent measurements were quantified with Image J. in (**B,D**).

It is known that cdc25 A, CDK2, cyclin D, cyclin B, and cyclin A play an important role in G1 arrest and/or S phase progression. Therefore, whether complexes **1** (8 *μ*M), **2** (15 *μ*M), **3** (5 *μ*M), **4** (18 *μ*M), **5** (28 *μ*M), **6** (14 *μ*M), **8** (10 *μ*M) and **11** (6 *μ*M) treatment effected on these five proteins in Hep-G2 cells was determined by Western blotting. As shown in Fig. 14 and Fig. S88, treatment with complexes **1** (8 *μ*M), **2** (15 *μ*M), **3** (5 *μ*M), **4** (18 *μ*M), **5** (28 *μ*M), **6** (14 *μ*M), **8** (10 *μ*M) and **11** (6 *μ*M) led to a decrease in these proteins in the Hep-G2 cells, likely due to complexes **1**–**6**, **8** and **11**, which caused Hep-G2 cell cycle arrest at S phase^67^. Because the CDK inhibitors p53, p21 or/and p27 are known to inhibit CDK activity^68^, we subsequently examined the effect of complexes **1** (8 *μ*M), **2** (15 *μ*M), **3** (5 *μ*M), **4** (18 *μ*M), **5** (28 *μ*M), **6** (14 *μ*M), **8** (10 *μ*M) and **11** (6 *μ*M) on these protein in Hep-G2 cells. Figures 14 and S88 shows that the levels of p53, p27, and p21 protein increased when treated with complexes **1** (8 *μ*M), **2** (15 *μ*M), **3** (5 *μ*M), **4** (18 *μ*M), **5** (28 *μ*M), **6** (14 *μ*M), **8** (10 *μ*M) and **11** (6 *μ*M) for 24 h. The results indicated that complexes **1**–**6**, **8** and **11** could block the Hep-G2 cell cycle progression into S phase.

### Comet Assay

**Figure 15:**
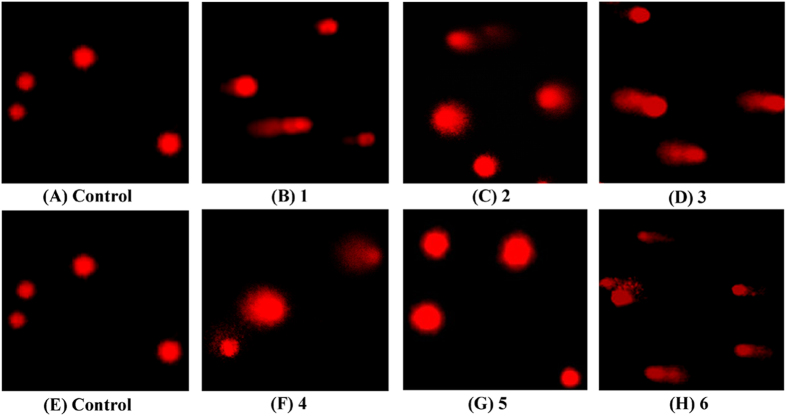
Complexes 1–6 -induced DNA damage in Hep-G2 cells. Cells were treated with complexes **1** (8 *μ*M), **2** (15 *μ*M), **3** (5 *μ*M), **4** (18 *μ*M), **5** (28 *μ*M) and **6** (14 *μ*M) with the indicated concentrations for 24 h and analyzed by comet assay, respectively. The length of the tail reflects the DNA damage in Hep-G2 cells.

To determine whether complexes **1** (8 *μ*M), **2** (15 *μ*M), **3** (5 *μ*M), **4** (18 *μ*M), **5** (28 *μ*M) and **6** (14 *μ*M) induced DNA damage in S phase, **a** comet assay at the single-cell level was used to detect DNA damage^69^. As shown in Fig. 15 and Table 3, treatment of Hep-G2 cells with **1** (8 *μ*M), **2** (15 *μ*M), **3** (5 *μ*M), **4** (18 *μ*M), **5** (28 *μ*M), and **6** (14 *μ*M), especially in cells treated with complex **3**, resulted in marked DNA damage as shown by the increased DNA tail size. Taken together, these results demonstrated that complexes **1** (8 *μ*M), **2** (15 *μ*M), **3** (5 *μ*M), **4** (18 *μ*M), **5** (28 *μ*M) and **6** (14 *μ*M) inhibited Hep-G2 cell growth via the induction of DNA damage and associated S-phase cell cycle arrest^70^.

### Assessment of Changes in Δψ

**Figure 16:**
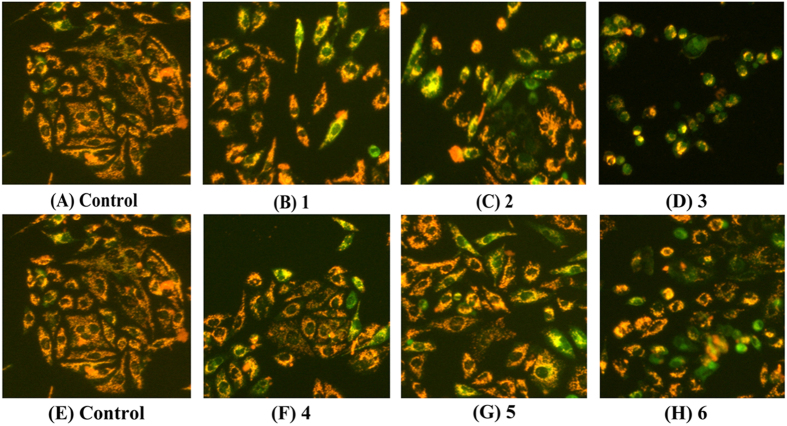
Loss of Δψ in Hep-G2 cells treated with complexes 1 (8 *μ*M), 2 (15 *μ*M), 3 (5 *μ*M), 4 (18 *μ*M), 5 (28 *μ*M) and 6 (14 *μ*M) for 24 h, and the cells was examined by a fluorescence microscope (Nikon Te2000, 200×) with stained by JC-1.

Complexes **1** (8 *μ*M), **2** (15 *μ*M), **3** (5 *μ*M), **4** (18 *μ*M), **5** (28 *μ*M) and **6** (14 *μ*M) accumulated in the mitochondrial fraction, whereas complexes **7**, **8**, **10** and **11** were only accumulated into the nuclear fraction. Therefore, only complexes **1**–**6** were selected to study the disruption of mitochondrial function using fluorescence morphological examination and a flow cytometry assay. The Hep-G2 cells treated with complexes **1** (8 *μ*M), **2** (15 *μ*M), **3** (5 *μ*M), **4** (18 *μ*M), **5** (28 *μ*M) and **6** (14 *μ*M) demonstrated a change in mitochondrial membrane potential (Δψ) as evidenced by a fluorescent emission shift from orange-red to green during JC-1 staining. As shown in Fig. 16, control cells showed a weak green fluorescence and an intense red fluorescence. However, the Hep-G2 cells treated with complexes **1** (8 *μ*M), **2** (15 *μ*M), **3** (5 *μ*M), **4** (18 *μ*M), **5** (28 *μ*M) and **6** (14 *μ*M), especially with complex **3** (5 *μ*M), exhibited a bright green fluorescence with a marked decrease in red fluorescence (orange-red), along with a remarkable decrease (green) in the orange-red fluorescence emission, indicating a loss of Δψ and a typical apoptotic morphology in Hep-G2 cells after 24 h. Therefore, it was concluded that complexes **1** (8 *μ*M), **2** (15 *μ*M), **3** (5 *μ*M), **4** (18 *μ*M), **5** (28 *μ*M) and **6** (14 *μ*M) induced Hep-G2 cell apoptosis.

**Figure 17:**
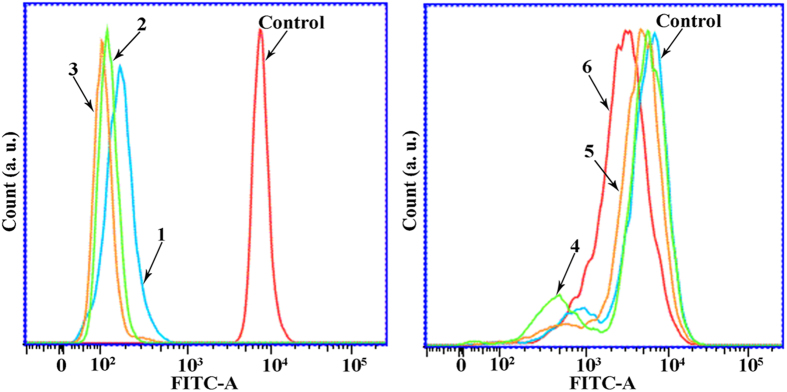
Effect of complexes 1 (8 *μ*M), 2 (15 *μ*M), 3 (5 *μ*M), 4 (18 *μ*M), 5 (28 *μ*M) and 6 (14 *μ*M) on Δψ in Hep-G2 cells, respectively. After treatment with complexes **1** (8 *μ*M), **2** (15 *μ*M), **3** (5 *μ*M), **4** (18 *μ*M), **5** (28 *μ*M) and **6** (14 *μ*M) for 24 h, the cells was examined by flow-cytometry with Rh 123 staining.

To obtain a detailed understanding of the cytotoxicity mechanism of complexes **1** (8 *μ*M), **2** (15 *μ*M), **3** (5 *μ*M), **4** (18 *μ*M), **5** (28 *μ*M) and **6** (14 *μ*M)-induced Hep-G2 cell apoptosis, Rh123 (Rhodamine 123) was used to determine the change of Δψ in Hep-G2 cells. As shown in Fig. 17, upon treatment with complexes **1** (8 *μ*M), **2** (15 *μ*M), **3** (5 *μ*M), **4** (18 *μ*M), **5** (28 *μ*M) and **6** (14 *μ*M), the fluorescent intensity decreased in Hep-G2 cells as compared with the control cells. These results indicated that complexes **1** (8 *μ*M), **2** (15 *μ*M), **3** (5 *μ*M), **4** (18 *μ*M), **5** (28 *μ*M) and **6** (14 *μ*M) were able to induce Δψ disruption in Hep-G2 cells, which was in agreement with the Δψ measurements obtained by fluorescent microscopy by JC-1 staining.

### Expressions of Apoptosis Related Proteins

**Figure 18:**
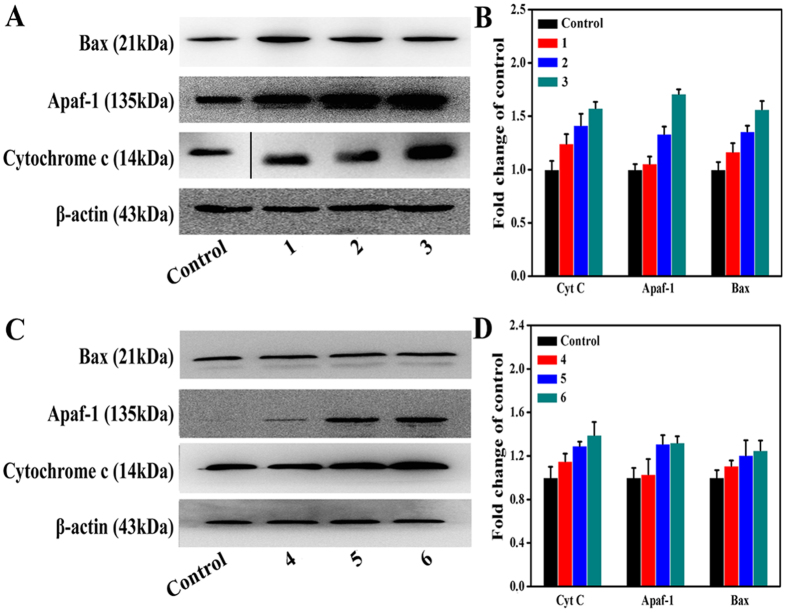
Western blot assay of apoptosis related protein levels in Hep-G2 cells treatmented with complexes 1 (8 *μ*M), 2 (15 *μ*M), 3 (5 *μ*M), 4 (18 *μ*M), 5 (28 *μ*M) and 6 (14 *μ*M) for 24 h, respectively. (**A,C**) Apoptosis related proteins protein levels in Hep-G2 cells were analyzed by western blot. (**B,D**) The whole-cell extracts were prepared and analyzed by Western blot analysis using antibodies against apoptosis related proteins. The same blots were stripped and reprobed with a β-actin antibody to show equal protein loading. Western blotting bands from three independent measurements were quantified with Image J. in (**B,D**).

To demonstrate the effects of complexes **1** (8 *μ*M), **2** (15 *μ*M), **3** (5 *μ*M), **4** (18 *μ*M), **5** (28 *μ*M) and **6** (14 *μ*M) on cytochrome c (Cyt C), apaf-1, and bax proteins, we carried out western blot analyses on these proteins in Hep-G2 cells. As shown in Fig. 18, significant increases in Cyt C, bax, and apaf-1 levels were observed, suggesting that complexes **1** (8 *μ*M), **2** (15 *μ*M), **3** (5 *μ*M), **4** (18 *μ*M), **5** (28 *μ*M) and **6** (14 *μ*M) induced cell apoptosis^71^.

### Measurement of ROS Generation

**Figure 19:**
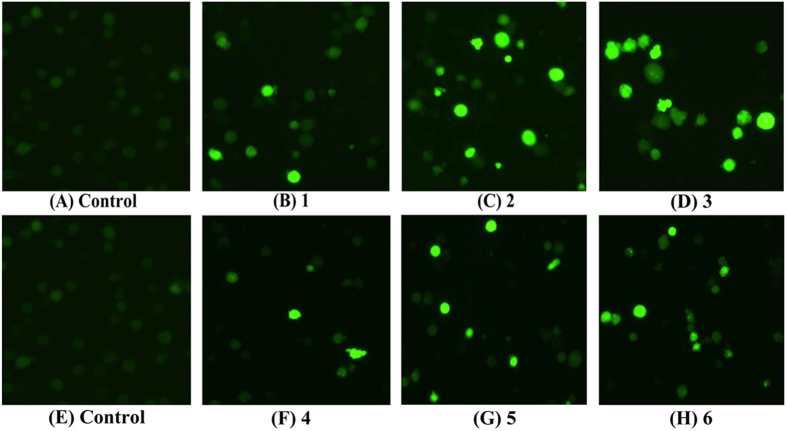
ROS generation assay in Hep-G2 cells was examined by a fluorescence microscope (Nikon Te2000, 200×). (**A,E**) Control and (**B–D**,**F–H**) Complexes **1** (8 *μ*M), **2** (15 *μ*M), **3** (5 *μ*M), **4** (18 *μ*M), **5** (28 *μ*M) and **6** (14 *μ*M) treatment with Hep-G2 cells for 24 h, respectively.

**Figure 20:**
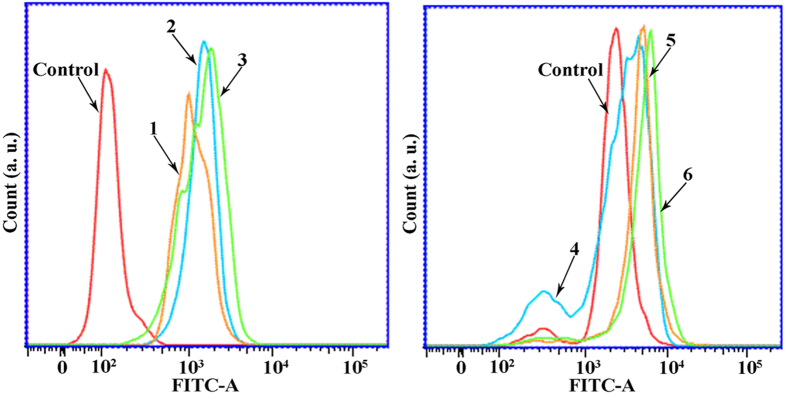
ROS generation assay in Hep-G2 cells was examined by flow cytometry of Hep-G2 cells after treated with complexes 1 (8 *μ*M), 2 (15 *μ*M), 3 (5 *μ*M), 4 (18 *μ*M), 5 (28 *μ*M) and 6 (14 *μ*M), respectively.

Compared with the control cells, complex **3** (5 *μ*M) showed much higher ability than complexes **1** (8 *μ*M), **2** (15 *μ*M), **4** (18 *μ*M), **5** (28 *μ*M) and **6** (14 *μ*M) to induce ROS generation (Fig. 19). These results are consistent with the results of the flow cytometric analysis (from left to right) in Hep-G2 cells (Fig. 20). In general, the results demonstrated that the effects of complexes **1** (8 *μ*M), **2** (15 *μ*M), **3** (5 *μ*M), **4** (18 *μ*M), **5** (28 *μ*M), and **6** (14 *μ*M) on the accumulation of ROS generation and finally on the induction of cell death/apoptosis^72^.

### 1–6 Induced Ca^2+^ Fluctuation

**Figure 21:**
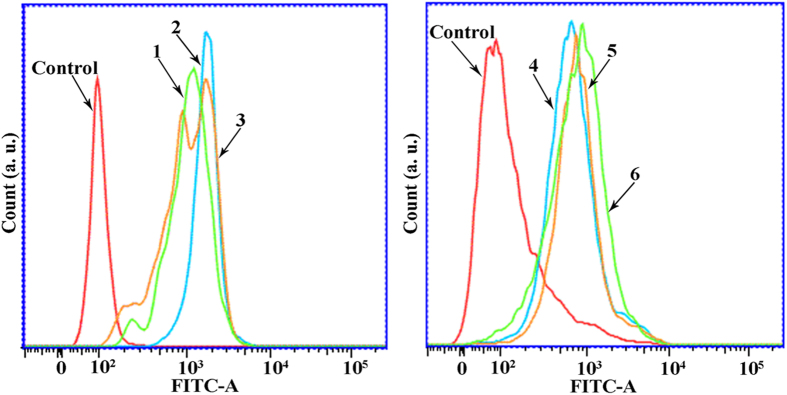
Effects of complexes 1 (8 *μ*M), 2 (15 *μ*M), 3 (5 *μ*M), 4 (18 *μ*M), 5 (28 *μ*M) and 6 (14 *μ*M) on Ca^2+^ activation level in Hep-G2 cells.

It was reported that disruption of intracellular Ca^2+^ (calcium) homeostasis is one of the characteristic events associated with human cancer cell apoptosis and mitochondrial membrane disruption^73^. For this, we examined the effects of complexes **1–6** on intracellular Ca^2+^ mobilization in Hep-G2 cells. Figure 21 displayed that the level of intracellular Ca^2+^ increased steadily (from left to right) in Hep-G2 cells after treated with complexes **1** (8 *μ*M), **2** (15 *μ*M), **3** (5 *μ*M), **4** (18 *μ*M), **5** (28 *μ*M), and **6** (14 *μ*M), especially in complex **3** (5 *μ*M) treated cells, whereas the values for the control cells were the lowest. These results demonstrated that Ca^2+^ activation was closely related to complexes **1–6** -induced cell apoptosis, which was consistent with the results of the Δψ disruption assay by JC-1 staining and Rhodamine 123 and cell apoptosis studies.

### Caspase 3/8/9 Activation Assay

**Figure 22:**
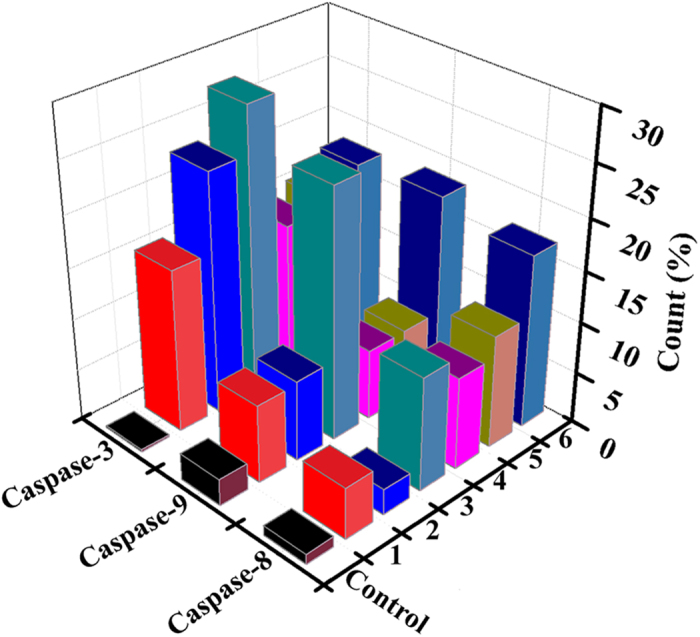
Activation of caspase-3/8/9 caused by complexes 1 (8 *μ*M), 2 (15 *μ*M), 3 (5 *μ*M), 4 (18 *μ*M), 5 (28 *μ*M) and 6 (14 *μ*M) in Hep-G2 cells for 24 h.

In order to assess the effects of **1** (8 *μ*M), **2** (15 *μ*M), **3** (5 *μ*M), **4** (18 *μ*M), **5** (28 *μ*M), and **6** (14 *μ*M) on caspase-3, caspase-8, and caspase-9 promoter activity and to investigate the potential molecular mechanisms involved^74^, flow cytometric analysis was used to determine their activated expression levels. Figures 22, S89, and S90 show that the activated expression levels of caspase-3 were 15.9%, 23.4%, 27.9%, 15.0%, 16.1%, and 17.9% in cells treated with **1** (8 *μ*M), **2** (15 *μ*M), **3** (5 *μ*M), **4** (18 *μ*M), **5** (28 *μ*M) and **6** (14 *μ*M), respectively. Similarly, the activated expression levels were 5.0%, 2.5%, 11.1%, 9.0%, 11.0%, and 16.9% for caspase-8, and 7.6%, 7.9%, 24.3%, 6.9%, 7.0% and 18.5% for caspase-9. Therefore, all the results indicated that complex **3** induced more cell apoptosis via triggering caspase-3/8/9 in Hep-G2 cells, comparing with that of complexes **1**, **2** and **4–6**.

### The Morphological Changes of Hep-G2 Cell Apoptosis

**Figure 23:**
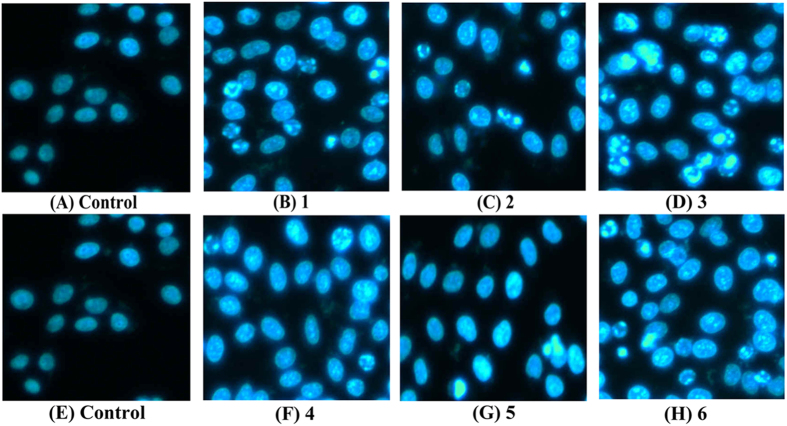
The morphological changes of apoptotic cell nucleus of Hep-G2 cells induced by complexes 1 (8 *μ*M), 2 (15 *μ*M), 3 (5 *μ*M), 4 (18 *μ*M), 5 (28 *μ*M), and 6 (14 *μ*M) for 24 h, and the Hep-G2 cells were examined by fluorescence microscope (Nikon Te2000, 400×) by staining with Hoechst 33258.

**Figure 24:**
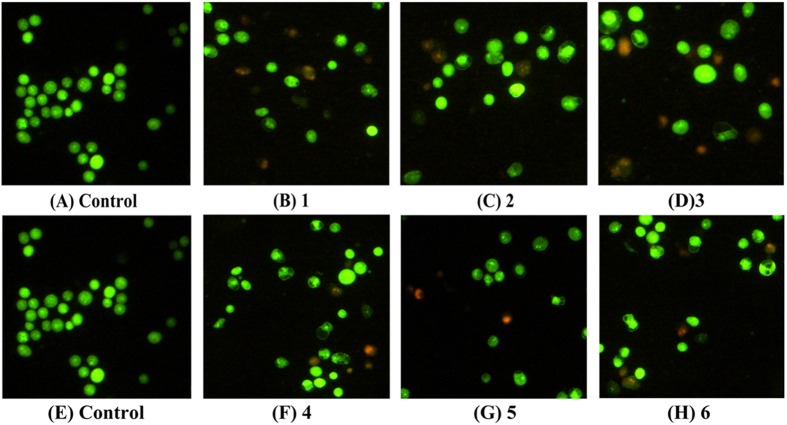
Apoptosis of Hep-G2 cells treated with complexes 1 (8 *μ*M), 2 (15 *μ*M), 3 (5 *μ*M), 4 (18 *μ*M), 5 (28 *μ*M), and 6 (14 *μ*M) for 24 h, respectively, and these cells the Hep-G2 cells were examined by fluorescence microscope (Nikon Te2000, 200×) by stained with AO/EB.

Various experiments showed that complexes **1**–**6**, especially **3** treated cells, were telomerase inhibitors targeting c-myc, telomeric, and bcl-2 G4s, which also caused telomeres/DNA damage, S phase arrest, and mitochondrial dysfunction, likely triggered cell apoptosis^13^,^43^,^60^. Thus, to further investigate the cell apoptosis or cell death mechanism, complexes **1** (8 *μ*M), **2** (15 *μ*M), **3** (5 *μ*M), **4** (18 *μ*M), **5** (28 *μ*M), and **6** (14 *μ*M)-treated Hep-G2 cells were stained with AO/EB and Hoechst 33258. The Hep-G2 cells stained with Hoechst 33258 showed typical apoptotic features, such as nuclear shrinkage (brightly stained) and condensation after treatment with complexes **1** (8 *μ*M), **2** (15 *μ*M), **3** (5 *μ*M), **4** (18 *μ*M), **5** (28 *μ*M), and **6** (14 *μ*M) for 24 h (Fig. 23), respectively. The amount of cell apoptosis significantly increased in Hep-G2 cells as the result of treatment with complexes **1** (8 *μ*M), **2** (15 *μ*M), **3** (5 *μ*M), **4** (18 *μ*M), **5** (28 *μ*M) and **6** (14 *μ*M). In addition, the number of apoptotic nuclei induced by complex **3** (5 *μ*M) increased more significantly compared with that induced by complexes **1** (8 *μ*M), **2** (15 *μ*M), **4** (18 *μ*M), **5** (28 *μ*M), and **6** (14 *μ*M). As shown in Fig. 24, the results of Hep-G2 cell apoptosis (orange) caused by complexes **1**–**6** agreed with cell apoptosis by AO and EB staining and double staining (Annexin V and PI).

### Apoptosis

**Figure 25:**
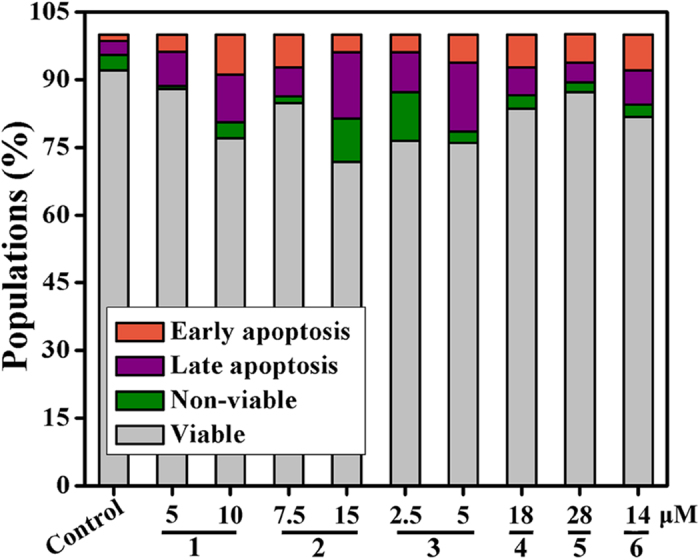
Populations of apoptotic Hep-G2 cells treated with complexes 1–6 were examined by FACS analysis with double staining by Annexin V and PI for visualization.

Cell apoptosis in Hep-G2 cells caused by complexes **1**–**6, 7**, **8**, **10** and **11** was further analyzed by flow cytometry with double staining with Annexin V and PI for visualization. 24 h treatment of the Hep-G2 cells with complexes **1** (8 *μ*M), **2** (15 *μ*M), **3** (5 *μ*M), **4** (18 *μ*M), **5** (28 *μ*M), **6** (14 *μ*M), **7** (16 *μ*M), **8** (10 *μ*M), **10** (12 *μ*M), and **11** (6 *μ*M) significantly induced Hep-G2 cell apoptosis (Q2 + Q3), respectively. As shown in Figs 25 and S91–S93, **3** (5 *μ*M) caused a higher percentage of apoptotic Hep-G2 cells (Q2 + Q3, ca. 21.43%) after 24 h of treatment as compared with 10 *μ*M **1** (ca. 19.33%), 15 *μ*M **2** (ca. 18.61%), 18 *μ*M **4** (ca. 13.49%), 28 *μ*M **5** (ca. 10.60%), 14 *μ*M **6** (ca. 15.45%), 16 *μ*M **7** (ca. 8.30%), 10 *μ*M **8** (ca. 10.35%), 11 *μ*M **10** (ca. 9.90%), and 6 *μ*M **11** (ca. 21.10%). All the results indicated that complex **3** could cause more apoptotic cell death than complexes **1**, **2**, **4–8**, **10** and **11** could in Hep-G2 cells, and the differential induction of cell apoptosis was in the following order: **3** > **1** > **6** > **4** > **2** > **5** and **3** > **11** > **6** > **8** > 10 > 7 (or **1** > **4**, **2** > **5, 3** > **6** and **11** > **8** > **10** > **7**).

### Effect on a Panel of Genes by RT-qPCR Array

**Figure 26:**
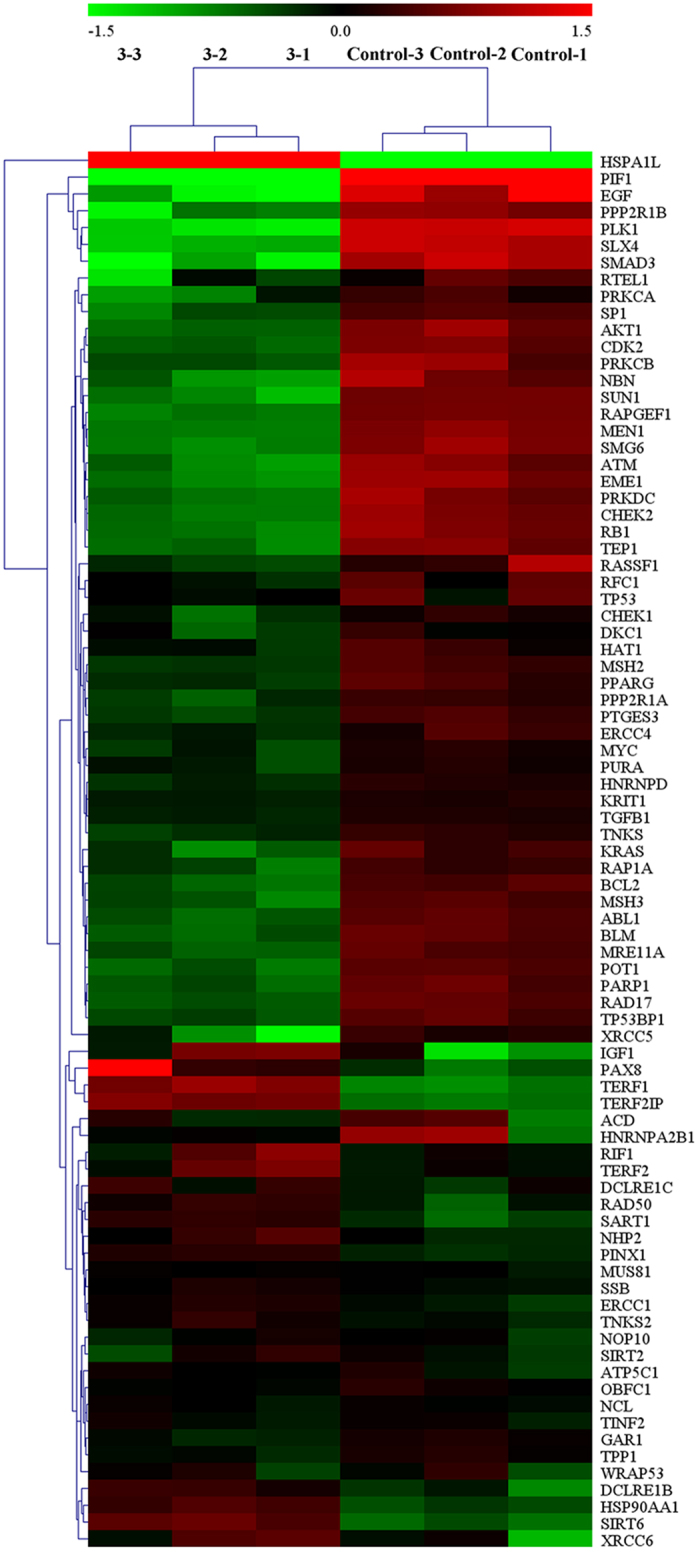
The mRNA expression level of telomeres/telomerase-related genes in the Hep-G2 cells after treated with complex 3 (5 *μ*M) for 24 h.

**Figure 27:**
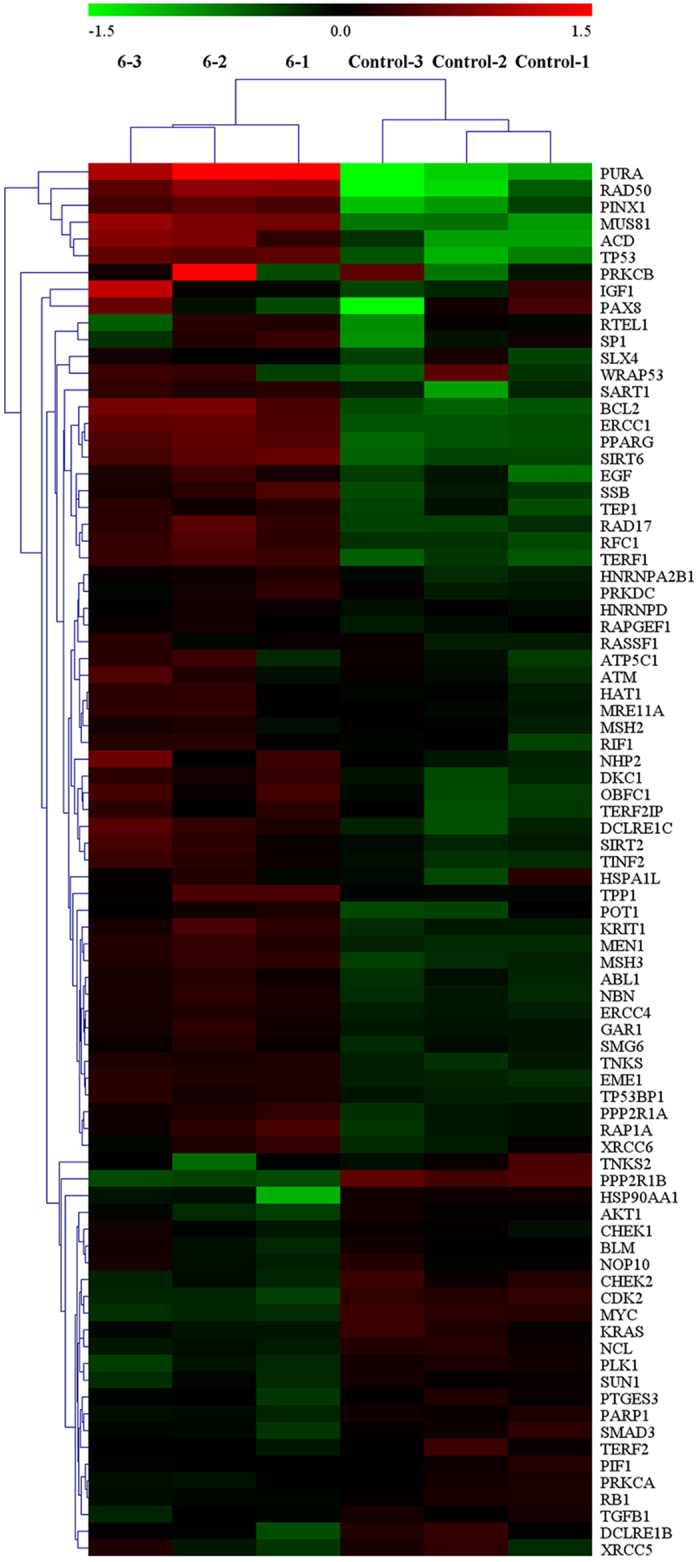
The mRNA expression level of telomeres/telomerase-related genes in the Hep-G2 cells after treated with complex 6 (14 *μ*M) for 24 h.

A Telomeres & Telomerase PCR Array was performed to confirm whether complex **3** (5 μM) or **6** (14 *μ*M) could down-regulate or up-regulate the telomeres/telomerase-related gene mRNA expression in the treated Hep-G2 cells^75^. As shown in Fig. 26 and Table S11, as compared with the untreated cells, of the 84 genes, 50 telomeres/telomerase-related gene mRNA levels more than 1.5-fold greater after treatment with complex **3** (5 *μ*M) for 24 h (i.e., up-regulated: DCLRE1B, HSP90AA1, HSPA1L, IGF1, PAX8, SART1, SIRT6, TERF1, TERF2IP and XRCC6; down-regulated: ABL1, AKT1, ATM, BCL2, BLM, CDK2, CHEK2, EGF, EME1, KRAS, MEN1, MRE11A, MSH2, MSH3, NBN, PARP1, PIF1, PLK1, POT1, PPARG, PPP2R1A, PPP2R1B, PRKCA, PRKCB, PRKDC, PTGES3, RAD17, RAP1A, RAPGEF1, RASSF1, RB1, RTEL1, SLX4, SMAD3, SMG6, SP1, SUN1, TEP1, TP53BP1 and XRCC5). In contrast, as shown in Fig. 27 and Table S12, only 18 genes telomeres/telomerase-related mRNA levels were higher than 1.5-fold after treatment with complex **6** (14 *μ*M) for 24 h (i.e., up-regulated: ACD, BCL2, DCLRE1C, EGF, ERCC1, MUS81, PINX1, PPARG, PURA, RAD17, RAD50, RFC1, SART1, SIRT6, SSB, TERF1 and TP53; down-regulated: PPP2R1B), which was consistent with the RT-PCR, transfection, and western blot analyses.

### Preliminary Safety Evaluation

0.6 mL/20 g of complex **3** (5% v/v DMSO/saline) was administered by intraperitoneal injection in mice to study its acute toxicity^76^. Treatment of 16 mg/kg/day (qd) or two times (bid) with complex **3**, KM mice (n = 6) showed similar trend (no signs of damage or peritonitis) in comparison with the control group (5% DMSO in saline, Tables S13–S15, Figs S28 and S94A). The result suggested that injection of **3** at 16 mg/kg two times a day was safe for mice; therefore, this dose was taken as the highest dose in antitumor studies. The same test method on **6** was carried out, and the results indicated that injection with **6** at 3.2 mg/kg every day was safe (Figs S94B, S95 and Tables S13–S15).

### *In vivo* antitumor activity of 3 and 6

**Figure 28:**
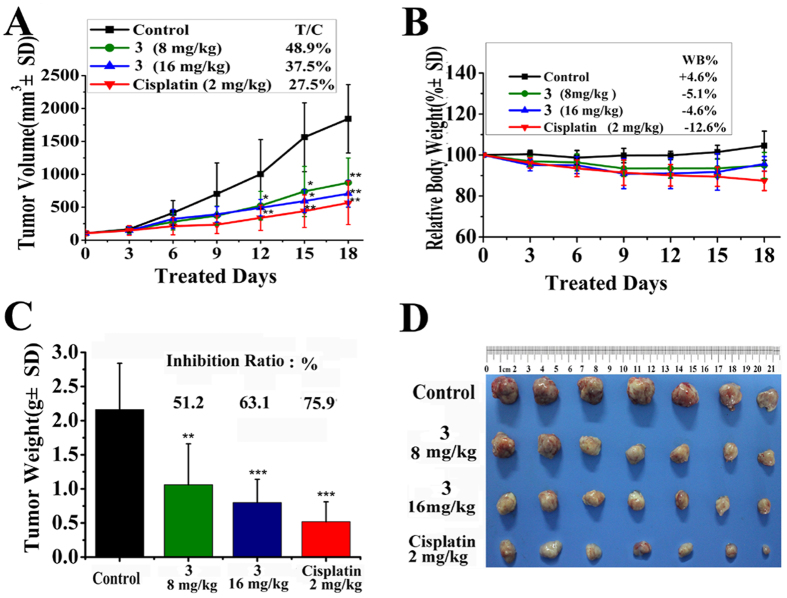
Complex 3 (16, 8 mg/kg/bid) and cisplatin (2 mg/kg/q2d) inhibited the growth of BEL-7402 tumor xenograft in compared with vehicle group, respectively. ***P < 0.01, **P < 0.05, *p* vs vehicle control.

The *in vitro* cytotoxicity studies of the group-10 metal complexes suggested that the newly synthesized complexes exhibited significant cytotoxicity in several human tumor cells. To investigate the efficacy of **3** to inhibit tumor growth *in vivo*, a mouse model of human hepatoma (BEL-7402) xenograft was used. The tumor-bearing nude mice received treatment when the tumor size was between 100 and 160 mm^3^. The mice were treated with **3** at 16 and 8 mg/kg two times every day, and treatment of cisplatin with 2 mg/kg/2days as the positive control. Fig. 28A and Tables S13–S15 showed the change in tumor volume in the control group and the treatment group. The tumor volume of vehicle control group grew rapidly from day 6, and reached a mean of 1840 mm^3^ on day 18. In comparison, the tumor volume grew slowly in groups receiving either **3** or cisplatin, with a mean volume of 705 mm^3^, 873 mm^3^ and 566 mm^3^ in groups treated with 16 mg/kg, 8 mg/kg of **3** and 2 mg/kg of cisplatin (ip, q2d), respectively. The relative tumor growth rates (T/C) were 37.5%, 48.9% (*p* < 0.01) and 27.5% (*p* < 0.01), respectively. Figure 28C showed that the antitumor activity of complex **3** in BEL-7402 model was 63.1% (*p* < 0.001) for day 18, but it was lower than that of cisplatin (75.9%, *p* < 0.001). However, **6** which possesses an 8-amino moiety on the benzene of L^b^, had an inhibition ratio of 27.8% (*p* < 0.05) at the highest dose, which was lower than that of **3** and cisplatin(Fig. S95).

No adverse effects were observed in the treatment. Mice treated with **3** and **6** showed favorable results without obvious losses in body weight, whereas treatment with cisplatin led to severe body weight loss (Figs 28 and S95), suggesting that **3** and **6** were safer antitumor agents and could inhibit the growth of BEL-7402 tumor *in vivo* comparably to cisplatin.

### SARs

Based on the above described results, the cytotoxic mechanism studies of different ligand (L^a^ and L^b^, L^c^ and L^c′^) and their complexes, certain SAR (structure-activity relationships) trends emerged among the different groups substituted in oxoisoaporphine ligands, the second ligands, and the metal centers.

- For the same metal center, with different ligands (L^a^ and L^b^, L^c^ and L^c′^), the *in vitro* antitumor activity and cellular uptake ability follow the order of 6-NH_2_ (L^a^) > 8-NH_2_ (L^b^), 7-methoxy (L^c^) > 7-carbenyl (L^c′^). Such SAR trends are further supported by the cytotoxic mechanism analyses, such as teleromerase activity, RT-PCR and Western blot of c-myc/hTERT/bcl-2, transfection and cell apoptosis, as well as cell cycle studies. In addition, the greater efficacy of 6-NH_2_ (L^a^) compared to 8-NH_2_ (L^b^) was also supported by telomere damage (or DNA damage) assays, cell senescence assays, a RT-qPCR array in Hep-G2 cells. Both **3** and **6** inhibited the growth of BEL-7402 xenograft *in vivo*.
- For the same metal center and L^c^ (L^c′^), with different second ligand, the *in vitro* antitumor activity is follow the order of en > pn > Cl and DMSO.
- For the same ligand complexes, with different metal centers, the *in vitro* antitumor activity and cellular uptake are follow the order of Pt > Ni > Pd.

## Conclusions

To incorporate a group-10 metal ion and an alkaloid into one molecule, complexes **1**–**14** of 6-amino-oxoisoaporphine (L^a^), 8-amino-oxoisoaporphine (L^b^), 8-chloro-oxoisoaporphine (L^c^) and 10-chloro-11-amino-oxoisoaporphine (L^d^) were synthesized and fully characterized. Complexes **1**–**14** displayed higher selectivity against Hep-G2 cells than against other selected human tumor cells, but lower toxicity in normal human cell line HL-7702. In particular, **3** showed the highest selectivity against Hep-G2 cells. In cytotoxicity studies, complexes **1**–**14** also displayed synergistic effects as compared with 6-amino-oxoisoaporphine (L^a^), 8-amino-oxoisoaporphine (L^b^), 8-chloro-oxoisoaporphine (L^c^), 10-chloro-11-amino-oxoisoaporphine (L^d^), and the free ions. Further studies demonstrated that complexes **1**–**6**, 7, **8**, **10** and **11**, especially **3**, were telomerase inhibitors targeting c-myc, telomeric, and bcl-2 G4s and triggered cell apoptosis and senescence, which also caused telomere/DNA damage and S phase arrest in the order **3** > **1** > **6** > **4** > **2** > **5** and **3** > **11** > **6** > **8** > 10 > 7 (or **1** > **4**, **2** > **5**, **3** > **6** and **11** > **8** > **10** > **7)**. In addition, **1**–**6** also caused mitochondrial dysfunction in Hep-G2 cells, which led to a significant increase in ROS, loss of Δψ, apaf-1, Ca^2+^ fluctuation, cytochrome c, and caspase-3/9 ratio, especially in **3** treated cells. In particular, **3** with 6-amino substituted in the ligand L^a^, exhibited better *in vivo* safety profiles than that of complex **6** and cisplatin as well as significant *in vivo* tumor growth efficacy in BEL-7402 xenograft mouse model. These results warrant further studies of other platinum-alkaloids complexes for their anticancer activities.

## Experimental Section

### Synthesis and Characterization of Ligands

Synthesis and characterization of 6-amino-oxoisoaporphine (L^a^), 8-amino-oxoisoaporphine (L^b^), 8-chloro-oxoisoaporphine (L^c^) and 10-chloro-11-amino-oxoisoaporphine (L^d^) have been reported^44^,^45^,^46^. In addition, the detailed synthetic procedures for four ligands L^a^−L^d^ were described in supporting information.

Data for 6-amino-oxoisoaporphine (L^a^): The brown color product of 6-amino-oxoisoaporphine (L^a^) was suitable for structural characterization. Yield (0.1230 g, 50.0%). ^1^H NMR (500 MHz, DMSO-*d*_*6*_): δ 7.08 (d, 1H, *J* = 9.5 Hz), 7.87–7.90 (m, 1H), 7.98 (t, 1H, *J* = 7.1Hz), 8.06 (d, 1H, *J* = 9.5 Hz), 8.73 (d, 1H, *J* = 8.2 Hz), 8.78 (d, 1H, *J* = 5.0 Hz), 9.21 (d, 1H, *J* = 8.1 Hz), 9.52 (s, Ar–NH, 1H), 12.43 (s, Ar–NH, 1H). ESI-MS m/z: 247.1 [M + H]^+^. Elemental analysis calcd (%) for C_16_H_10_N_2_O: C 78.03, H 4.09, N 11.38; found: C 78.01, H 4.14, N 11.42.

Data for 8-amino-oxoisoaporphine (L^b^): The brown color product was suitable for structural characterization. Yield (0.0984 g, 40.0%). ^1^H NMR (600 MHz, DMSO-*d*_*6*_) δ 8.71 (d, *J* = 5.5 Hz, 1H), 8.43 (dd, *J* = 7.2, 0.8 Hz, 1H), 8.27 (d, *J* = 7.6 Hz, 1H), 8.03 (dd, *J* = 7.4, 0.9 Hz, 1H), 7.98 (dd, *J* = 8.0, 7.4 Hz, 1H), 7.92 (d, *J* = 5.5 Hz, 1H), 7.54–7.43 (m, 1H), 6.98 (dd, *J* = 8.4, 0.9 Hz, 1H). ^13^C NMR (151 MHz, DMSO-*d*_*6*_) δ 184.12, 153.17, 148.74, 144.08, 136.83, 134.99, 134.94, 132.46, 131.05, 129.82, 128.26, 122.17, 120.70, 119.09, 112.59, 112.55. ESI-MS m/z: 247.1 [M + H]^+^; IR (KBr): 3853, 3744, 3421, 3304, 3047, 2936, 1932, 1731, 1600, 1538, 1447, 1405, 1324, 1278, 1205, 1167, 1034, 934, 853, 819, 706, 606, 541, 474 cm^−1^. Elemental analysis calcd (%) for C_16_H_10_N_2_O: C 78.03, H 6.09, N 11.38; found: C 77.96, H 6.14, N 12.42.

Data for 8-chloro-oxoisoaporphine (L^c^): The yellow color product (8-chloro-oxoisoaporphine (L^c^)) was suitable for structural characterization. Yield (0.0191 g, 72.0%). ^1^H NMR (600 MHz, DMSO-*d*_*6*_) δ 8.74 (s, 1H), 8.67 (s, 1H), 8.34 (s, 1H), 8.28 (s, 1H), 7.92 (s, 2H), 7.69 (s, 1H), 7.64 (s, 1H). ^13^C NMR (151 MHz, DMSO-*d*_*6*_) δ 181.57, 147.24, 144.59, 139.50, 135.07, 134.69, 134.60, 134.08, 131.80, 130.41, 129.41, 128.39, 125.11, 122.25, 122.02. ESI-MS m/z: 443.9 [M + Cl + DMSO + 2CH_3_OH]^−^; IR (KBr): 3852, 3741, 3304, 3056, 1972, 1660, 1613, 1581, 1495, 1442, 1392, 1330, 1291, 1263, 1207, 1166, 1076, 1029, 953, 904, 856, 810, 737, 697, 536, 464, 416 cm^−1^. Elemental analysis calcd (%) for C_16_H_8_ClNO: C 72.33, H 3.03, N 5.27; found: C 72.29, H 3.10, N 5.21.

Data for 10-chloro-11-amino-oxoisoaporphine (L^d^): The brown color product was suitable for structural characterization. Yield (0.6826 g, 38.0%). ^1^H NMR (600 MHz, DMSO-*d*_*6*_) δ 8.80 (d, *J* = 5.4 Hz, 1H), 8.45 (d, *J* = 7.2 Hz, 1H), 8.32 (d, *J* = 8.1 Hz, 1H), 8.01–7.98 (m, 1H), 7.96 (d, *J* = 5.5 Hz, 1H), 7.53 (d, *J* = 9.1 Hz, 1H), 7.01 (d, *J* = 9.1 Hz, 1H). ^13^C NMR (151 MHz, DMSO-*d*_*6*_) δ 183.98, 153.26, 148.75, 143.28, 139.99, 135.07, 133.42, 132.20, 131.25, 129.36, 128.57, 122.93, 121.20, 120.82, 118.82, 114.00. ESI-MS m/z: 281.0 [M + H]^+^; IR (KBr): 3853, 3746, 3423, 3294, 3054, 2926, 1916, 1605, 1538, 1445, 1384, 1316, 1264, 1122, 1081, 967, 856, 823, 751, 718, 504 cm^−1^. Elemental analysis calcd (%) for C_16_H_9_ClN_2_O: C 68.46, H 3.23, N 9.98; found: C 68.50, H 3.18, N 10.02.

### Synthesis of 6-Amino-Oxoisoaporphine (L^a^) and 8-Amino-Oxoisoaporphine (L^b^) Metal Complexes (1–6)

#### Synthesis of [Ni(L^a^)Cl_2_] (1), [Pd(L^a^)Cl_2_] (2), [Ni(L^b^)Cl_2_] (4), [Pd(L^b^)Cl_2_] (5) and [Pt(L^b^)(DMSO)Cl]·H_2_O (6)

NiCl_2_·6H_2_O, PdCl_2_ or cis-Pt(DMSO)_2_Cl_2_ (5.0 mmol) was first dissolved in distilled water (2.5 mL) or CH_3_CN (2.5 mL) and heated to near boiling. The hot solution was added to 5.0 mmol 6-amino-oxoisoaporphine (L^a^) or 8-amino-oxoisoaporphine (L^b^) in ethanol (50.0 mL), and the mixture was stirred at reflux for 12 h, cooled overnight, filtered for about four days, and each product of **1** (clear green), **2, 5** and **6** (black) and **4** (brown) suitable for structural characterization were isolated, washed with ethanol and water or ethanol and CH_3_CN, similar to the synthesis of Ni(L)Cl_2_^77^.

Data for **1**: The green color product ([Ni(L^a^)Cl_2_] (**1**)) was suitable for its structural characterization. Yield (0.033 g, 87%). ^1^H NMR (600 MHz, DMSO-*d*_*6*_) δ 10.18 (s, 1H), 8.73 (s, 1H), 8.49 (d, *J* = 55.3 Hz, 2H), 8.21 (s, 1H), 7.85 (s, 1H), 7.62 (s, 2H), 7.52 (s, 1H), 7.29 (s, 1H). ^13^C NMR (151 MHz, DMSO-*d*_*6*_) δ 182.05, 156.07, 142.76, 141.91, 136.39, 135.84, 132.89, 132.68, 129.75, 129.23, 126.43, 125.37, 124.82, 123.33, 122.19, 103.49. ESI-MS m/z: 392.92 [M−Cl + 3H_2_O]^+^; IR (KBr): 3417, 3141, 3034, 1630, 1578, 1524, 1446, 1286, 1221, 1163, 854 cm^−1^. Elemental analysis calcd (%) for C_16_H_10_Cl_2_N_2_ONi: C 51.13, H 2.68, N 7.45; found: C 51.10, H 2.73, N 7.48.

Data for **2**: The black color product ([Pd(L^a^)Cl_2_] (**2**)) was suitable for its structural characterization. Yield (0.034 g, 80%). ^1^H NMR (600 MHz, DMSO-*d*_*6*_) δ 10.27 (s, 1H), 9.03 (s, 1H), 8.88–8.72 (m, 1H), 8.35 (d, *J* = 11.9 Hz, 1H), 8.12 (d, *J* = 9.3 Hz, 1H), 8.02 (s, 1H), 7.91 (t, *J* = 5.9 Hz, 2H), 7.58 (d, *J* = 9.3 Hz, 1H), 7.50 (d, *J* = 9.8 Hz, 1H). ESI-MS m/z: 422.91 [M−Cl + 2H_2_O]^+^; IR (KBr): 3450, 3313, 2917, 1618, 1553, 1517, 1478, 1421, 1342, 1248, 1174, 848, 787 cm^−1^. Elemental analysis calcd (%) for C_16_H_10_Cl_2_N_2_OPd: C 45.37, H 2.38, N 6.61; found: C 45.35, H 2.41, N 6.65.

Data for **4**: The brown color product ([Ni(L^b^)Cl_2_] (**4**)) was suitable for its structural characterization. Yield (0.028 g, 75%). ^1^H NMR (600 MHz, DMSO-*d*_*6*_) δ 8.73 (d, *J* = 5.5 Hz, 1H), 8.47–8.42 (m, 1H), 8.29 (d, *J* = 8.2 Hz, 1H), 8.05 (d, *J* = 7.4 Hz, 1H), 8.02–7.97 (m, 1H), 7.94 (d, *J* = 5.5 Hz, 1H), 7.50 (t, *J* = 7.9 Hz, 1H), 6.99 (d, *J* = 8.3 Hz, 1H). ^13^C NMR (151 MHz, DMSO-*d*_*6*_) δ 184.13, 153.18, 148.76, 144.10, 136.84, 135.01, 134.96, 132.48, 131.08, 129.83, 128.28, 122.18, 120.72, 119.11, 112.59, 112.55. ESI-MS m/z: 372.31 [M−Cl + CH_3_OH]^+^; IR (KBr): 3850, 3743, 3425, 3308, 3051, 2927, 1601, 1539, 1448, 1405, 1324, 1278, 1206, 1167, 1037, 934, 820, 706, 541 cm^−1^. Elemental analysis calcd (%) for C_16_H_10_Cl_2_N_2_ONi: C 51.13, H 2.68, N 7.45; found: C 51.16, H 2.65, N 7.50.

Data for **5**: The black color product ([Pd(L^b^)Cl_2_] (**5**)) was suitable for its structural characterization. Yield (0.029 g, 68%). ^1^H NMR (600 MHz, DMSO-*d*_*6*_) δ 8.86 (d, *J* = 4.2 Hz, 1H), 8.25 (d, *J* = 5.3 Hz, 1H), 8.17 (d, *J* = 6.7 Hz, 1H), 8.02–7.98 (m, 1H), 7.92 (d, *J* = 7.9 Hz, 1H), 7.76 (d, *J* = 5.6 Hz, 1H), 7.63 (d, *J* = 8.0 Hz, 1H), 6.65 (d, *J* = 7.9 Hz, 1H). ^13^C NMR (151 MHz, DMSO-*d*_*6*_) δ 183.02, 150.81, 144.89, 142.78, 142.50, 138.40, 135.11, 133.62, 131.58, 129.97, 129.09, 121.43, 119.66 × 2, 118.80, 112.61. ESI-MS m/z: 527.45 [M + Cl + CH_3_OH + 2H_2_O]^−^; IR (KBr): 3854, 3745, 3321, 2923, 1597, 1514, 1391, 1276, 1168, 1115, 939, 816, 718, 649, 535, 493 cm^−1^. Elemental analysis calcd (%) for C_16_H_10_Cl_2_N_2_OPd: C 45.37, H 2.38, N 6.61; found: C 45.30, H 2.32, N 6.68.

Data for **6**: The black color product ([Pt(L^b^)(DMSO)Cl]·H_2_O (**6**)) was suitable for its structural characterization. Yield (0.0466 g, 84%). ^1^H NMR (600 MHz, DMSO-*d*_*6*_) δ 8.93 (d, *J* = 6.4 Hz, 1H), 8.15 (d, *J* = 7.2 Hz, 1H), 8.05 (d, *J* = 8.1 Hz, 1H), 8.02 (d, *J* = 8.8 Hz, 1H), 7.95–7.84 (m, 2H), 7.68 (d, *J* = 6.6 Hz, 1H), 6.64 (d, *J* = 8.8 Hz, 1H), 2.46 (s, 6 H). ^13^C NMR (151 MHz, DMSO-*d*_*6*_) δ 183.22, 161.84, 150.33, 144.22, 139.68, 135.74, 135.26, 134.34, 131.56, 130.75, 130.59, 129.42, 121.57, 120.22, 119.65, 112.74, 40.91 × 2. ESI-MS m/z: 589.11 [M + Cl]^−^; IR (KBr): 3853, 3746, 3441, 3311, 3067, 3007, 2919, 1598, 1565, 1509, 1467, 1405, 1340, 1282, 1237, 1196, 1125, 1023, 979, 937, 820, 699, 540, 502, 446 cm^−1^. Elemental analysis calcd (%) for C_18_H_17_ClN_2_O_2_PtS: C 38.89, H 3.08, N 5.04; Found: C 38.85, H 3.05, N 5.10.

#### Synthesis of [Pt(L^a^)(DMSO)Cl]·H_2_O (3)

The brown block crystals of [Pt(L^**a**^)(DMSO)Cl]·H_2_O (**3**) was prepared by treating 0.0422 g cis-Pt(DMSO)_2_Cl_2_ (0.1 mmol) and 0.1 mmol 6-amino-oxoisoaporphine (0.0246 g) in ethanol/water (20:1) under solvothermal conditions. Yield (0.0505 g, 91%). ^1^H NMR (500 MHz, DMSO-*d*_*6*_) δ 9.06 (d, *J* = 7.4 Hz, 1H), 8.84 (d, *J* = 4.7 Hz, 1H), 8.63 (d, *J* = 7.5 Hz, 1H), 8.27 (s, 1H), 8.01–7.96 (m, 1H), 7.92 (dd, *J* = 12.5, 7.1 Hz, 2H), 7.87–7.79 (m, 1H), 7.62 (d, *J* = 9.5 Hz, 1H), 3.17–3.20 (m, 2H), 2.54 (s, 6 H). ^13^C NMR (151 MHz, DMSO-*d*_*6*_) δ 181.87, 162.94, 156.20, 136.36, 133.00, 132.84, 130.21, 129.83, 126.64, 126.03, 124.87, 123.57, 122.52, 121.56, 103.49, 40.91×2, 40.54. ESI-MS *m/z*: 555.1 [M-Cl + 2H_2_O]^+^. IR (KBr): 3440, 3298, 2995, 1627, 1572, 1528, 1440, 1405, 1357, 1270, 1122, 1036, 982, 851, 787, 716 cm^−1^. Elemental analysis calcd (%) for C_18_H_17_ClN_2_O_2_PtS: C 38.89, H 3.08, N 5.04; Found: C 38.83, H 3.12, N 5.08.

### Synthesis of 8-Chloro-Oxoisoaporphine (L^c^) Platinum Complexes (7–11)

#### Synthesis of [Pt(L^c^)(DMSO)Cl] (7)

0.0422 g cis-Pt(DMSO)_2_Cl_2_ (0.1 mmol), 0.0265 g L^c^ (0.1 mmol), 1.0 mL ethanol, 0.05 mL distilled water were placed into 25 cm long Pyrex tube that was then quenched in liquid N_2_, before evacuated and sealed, which was heated at 80 °C for four days. Received black block crystals were used for X-ray diffraction analysis. Yield (0.0441 g, 77%). ESI-MS m/z: 529.9 [M−DMSO + Cl]^−^; ^1^H NMR (600 MHz, DMSO-*d*_*6*_) δ 8.82 (s, 1H), 8.71 (s, 1H), 8.56 (s, 1H), 8.26 (s, 1H), 8.15 (s, 1H), 7.78 (s, 1H), 7.37 (s, 1H), 2.53 (s, 6 H). IR (KBr): 3851, 3742, 3442, 3066, 3008, 2920, 2380, 2237, 1932, 1660, 1618, 1542, 1501, 1462, 1413, 1277, 1243, 1205, 1125, 1029, 982, 950, 823, 757, 722, 688, 581, 520, 448 cm^−1^. Elemental analysis calcd (%) for C_18_H_13_Cl_2_NO_2_PtS: C 37.71, H 2.29, N 2.44; found: C 37.65, H 2.34, N 2.40.

#### Synthesis of [Pt(L^c^)(en)]Cl (8) and [Pt(L^c^)(pn)]Cl (9)

5.0 mmol [Pt(L^c^)(DMSO)Cl] **(7)** was dissolved into 20 mL anhydrous ethanol and heated to near boiling. The hot solution was added to 5.0 mmol 1,2-ethylenediamine (en) or 1,3-propanediamine (pn), and the mixture was stirred at reflux for 12 h, cooled overnight, filtered for about four days, and the black product of **8** and **9** suitable for structural characterization were isolated, washed with anhydrous ethanol, similar to the synthesis of Ni(L)Cl_2_^77^.

Data for **8**: The black color product ([Pt(L^c^)(en)]Cl (**8**)) was suitable for its structural characterization. Yield (0.0499 g, 90%). ^1^H NMR (600 MHz, DMSO-*d*_*6*_) δ 8.54 (s, 1H), 8.33 (s, 2H), 8.11 (s, 1H), 8.00 (s, 1H), 7.59 (s, 1H), 7.40 (s, 1H), 6.36 (s, 4H), 5.65 (s, 4H). ^13^C NMR (151 MHz, DMSO-*d*_*6*_) δ 181.53, 160.70, 149.35, 144.78, 134.78, 134.20, 133.34, 131.91, 129.29, 128.09, 122.94, 121.78, 75.91, 48.58, 46.42, 44.49. ESI-MS m/z: 520.0 [M−Cl]^+^; IR (KBr): 3853, 3744, 3368, 3182, 3068, 2961, 2384, 2297, 1910, 1660, 1606, 1469, 1387, 1320, 1281, 1237, 1154, 1057, 1005, 952, 822, 764, 724, 583, 523, 445 cm^−1^. Elemental analysis calcd (%) for C_18_H_15_Cl_2_N_3_OPt: C 38.93, H 2.72, N 7.57; found: C 39.01, H 2.69, N 7.51.

Data for **9**: The black color product ([Pt(L^c^)(pn)]Cl (**9**)) was suitable for its structural characterization. Yield (0.0494 g, 87%). ^1^H NMR (600 MHz, DMSO-*d*_*6*_) δ 8.57 (s, 1H), 8.38 (s, 1H), 8.36 (s, 1H), 8.14 (s, 1H), 8.03 (s, 1H), 7.62 (s, 1H), 7.43 (s, 1H), 6.36 (s, 4H), 5.64 (s, 6 H). ESI-MS m/z: 534.0 [M−Cl]^+^; IR (KBr): 3151, 3084, 2939, 2878, 2376, 1900, 1660, 1573, 1470, 1287, 1234, 1200, 1161, 1081, 947, 818, 762, 725, 585, 521, 436 cm^−1^. Elemental analysis calcd (%) for C_19_H_17_Cl_2_N_3_OPt: C 40.08, H 3.01, N 7.38; found: C 40.15, H 2.94, N 3.05.

#### Synthesis of [Pt(L^c′^)(DMSO)Cl] (10)

The brown block crystals of 0.0265 g L^c^ (0.1 mmol), 0.0422 g cis-Pt(DMSO)_2_Cl_2_ (0.1 mmol), 1.0 mL methanol, 0.05 mL CH_3_CN were placed into 25 cm long Pyrex tube that was then quenched in liquid N_2_, before evacuated and sealed, which was heated at 80 °C for four days. Yield (0.0541 g, 92%). ESI-MS m/z: 515.1 [M−DMSO−Cl + CH_3_CN]^+^; ^1^H NMR (600 MHz, DMSO-*d*_*6*_) δ 8.96 (s, 1H), 8.86 (s, 1H1H), 8.45 (s, 1H), 8.42 (s, 1H), 8.06 (s, 1H), 7.99 (s, 1H), 7.82 (s, 1H), 7.64 (s, 1H), 2.53 (s, 9 H). IR (KBr): 3850, 3743, 3443, 3023, 2920, 2379, 1926, 1630, 1604, 1567, 1483, 1440, 1336, 1303, 1263, 1230, 1132, 1024, 978, 854, 798, 949, 686, 595, 519, 450 cm^−1^. Elemental analysis calcd (%) for C_19_H_17_Cl_2_NO_2_PtS: C 38.72, H 2.91, N 2.38; found: C 38.65, H 2.97, N 2.35.

#### Synthesis of [Pt(L^c′^)(en)]Cl (11)

5.0 mmol [Pt(L^c′^)(DMSO)Cl] **(10)** was dissolved into 20 mL anhydrous methanol and heated to near boiling. The hot solution was added to 5.0 mmol 1,2-ethylenediamine (en), and the mixture was stirred at reflux for 12 h, cooled overnight, filtered for about four days, and the brown product of **11** suitable for structural characterization were isolated, washed with anhydrous ethanol, similar to the synthesis of Ni(L)Cl_2_^77^. Yield (0.0473 g, 83%). ^1^H NMR (600 MHz, DMSO-*d*_*6*_)δ 8.13 (s, 1H), 7.82 (s, 1H), 7.62 (s, 1H), 7.48 (s, 1H), 7.43 (s, 1H), 7.26 (s, 1H), 6.87 (s, 1H), 6.11 (s, 4H), 5.38 (s, 4H), 2.58 (s, 3H). ESI-MS m/z: 534.1 [M−Cl]^+^; IR (KBr): 3398, 3310, 3183, 3061, 2348, 1935, 1610, 1564, 1513, 1436, 1363, 1301, 1126, 1146, 1056, 1011, 946, 850, 779, 747, 618, 589, 520, 467, 428 cm^−1^. Elemental analysis calcd (%) for C_19_H_19_Cl_2_N_3_OPt: C 39.94, H 3.35, N 7.35; found: C 39.87, H 3.42, N 7.32.

### Synthesis of [Ni(L^d^)Cl_2_] (12), [Pd(L^d^)Cl_2_] (13) and [Pt(L^d^)Cl_2_] (14)

NiCl_2_·6H_2_O, PdCl_2_ or cis-Pt(DMSO)_2_Cl_2_ (5.0 mmol) was first dissolved in distilled water (2.0 mL) and heated to near boiling. The hot solution was added to a hot solution of 10-chloro-11-amino-oxoisoaporphine (L^c^) (5.0 mmol) in methanol and CH_3_CN (v/v = 30.0 mL/10.0 mL) mixture solution, and the mixture was stirred at reflux for 12 h, cooled overnight, filtered for about four days, and each product of **12** (clear brown), **13** and **14** (black) suitable for structural characterization were isolated, washed with methanol, CH_3_CN and water mixture solution (v/v = 15:5:1), very similar to the synthesis of Ni(L)Cl_2_^77^.

Data for **12**: The brown color product ([Ni(L^c^)Cl_2_] (**12**)) was suitable for its structural characterization. Yield (0.029 g, 72%). ^1^H NMR (600 MHz, DMSO-*d*_*6*_) δ 8.63 (d, *J* = 6.5 Hz, 1H), 8.28 (d, *J* = 7.0 Hz, 1H), 8.15 (d, *J* = 8.0 Hz, 1H), 7.87–7.74 (m, 2H), 7.35 (d, *J* = 6.1 Hz, 1H), 6.88 (d, *J* = 6.2 Hz, 1H). ^13^C NMR (151 MHz, DMSO-*d*_*6*_) δ 184.70, 153.99, 149.44, 144.06, 140.71, 135.78, 134.26, 132.86, 132.09, 130.05, 129.33, 123.62, 122.03, 121.71, 119.51, 114.68. ESI-MS m/z: 514.29 [M + Cl + CH_3_OH + H_2_O]^−^; IR (KBr): 3852, 3744, 3422, 3293, 2927, 1604, 1538, 1444, 1384, 1314, 1263, 1120, 856, 823, 651, 537 cm^−1^. Elemental analysis calcd (%) for C_16_H_9_Cl_3_N_2_ONi: C 46.84, H 2.21, N 6.83; found: C 46.80, H 2.23, N 6.88.

Data for **13**: The black color product ([Pd(L^c^)Cl_2_] (**13**)) was suitable for its structural characterization. Yield (0.027 g, 60%). ^1^H NMR (600 MHz, DMSO-*d*_*6*_) δ 8.71 (d, *J* = 5.4 Hz, 1H), 8.35 (d, *J* = 7.1 Hz, 1H), 8.22 (d, *J* = 7.8 Hz, 1H), 7.91–7.88 (m, 1H), 7.87 (d, *J* = 5.4 Hz, 1H), 7.45 (d, *J* = 9.1 Hz, 1H), 6.95 (d, *J* = 9.1 Hz, 1H). ^13^C NMR (151 MHz, DMSO-*d*_*6*_) δ 183.60, 152.96, 148.36, 142.79, 139.63, 134.78, 133.06, 131.79, 130.94, 129.05, 128.25, 122.62, 120.92, 120.58, 118.62, 113.70. ESI-MS m/z: 587.9 [M + Cl + DMSO + H_2_O]^−^; IR (KBr): 3777, 3426, 2925, 2361, 1609, 1532, 1385, 1278, 1175, 1119, 818, 718, 479 cm^−1^. Elemental analysis calcd (%) for C_16_H_9_Cl_3_N_2_OPd: C 41.96, H 1.98, N 6.12; found: C 41.90, H 2.05, N 6.17.

Data for **14**: The black color product ([Pt(L^d^)Cl_2_] (**14**)) was suitable for its structural characterization. Yield (0.0447 g, 82%). ^1^H NMR (600 MHz, DMSO-*d*_*6*_) δ 8.72 (d, *J* = 5.4 Hz, 1H), 8.35 (d, *J* = 6.6 Hz, 1H), 8.22 (d, *J* = 8.1 Hz, 1H), 7.90 (t, *J* = 7.8 Hz, 1H), 7.87 (d, *J* = 6.2 Hz, 1H), 7.45 (d, *J* = 9.1 Hz, 2H), 6.95 (d, *J* = 9.0 Hz, 1H). ^13^C NMR (151 MHz, DMSO-*d*_*6*_) δ 183.76, 153.11, 148.52, 139.79, 138.89, 134.92, 133.21, 131.95, 131.09, 129.19, 128.40, 122.77, 121.07, 120.73, 118.77, 113.86. ESI-MS m/z: 585.2 [M-Cl + CH_3_CN + CH_3_OH]^+^; IR (KBr): 3854, 3746, 3433, 3068, 2921, 1614, 1570, 1512, 1387, 1334, 1125, 1025, 820, 706, 535 cm^−1^. Elemental analysis calcd (%) for C_16_H_9_Cl_3_N_2_OPt: C 35.15, H 1.66, N 5.12; Found: C 35.11, H 1.62, N 5.18.

## Materials and Methods

Abbreviations, DNA oligomers and cell lines used in this work are listed in Table 4. The X-Ray crystallography structures of L^a^, complexes **3**, **7** and **10** were solved by Sheldrick method^78^. The antitumor mechanism of complexes **1**–**14** were similar to that reported by Chen^43^,^79^,^80^. The TRAP-silver staining assay of complexes **1**–**6**, **8** and **11** were performed as reported by Neidle and Reed^31^. In addition, the comet assay of complexes **1**–**6** were performed as Hofer and co-workers reported^81^. Animal used, acute toxicity studies and antitumor activity toward BEL-7402 *in vivo* of complex **3** (16 and 8 mg/kg, two time a day,17 days (ip)), complex **6** (3.2 and 1.6 mg/kg/ day, 17 days) and cisplatin (2 mg/kg/2days), were similar to that reported by Chen^43^,^80^. BEL-7402 xenograft mouse models were purchased from Beijing HFK Bioscience Co., Ltd (Beijing, China, approval No. SCXK 2014-004). The animal procedures were approved by the Institute of Radiation Medicine Chinese Academy of Medical Sciences (Tian Jin, China, approval No. SYXK 2014-0002). And all of the experimental procedures were carried out in accordance with the NIH Guidelines for the Care and Use of Laboratory Animals. Animal experiments were approved by the Animal Care and Use Committee of Institute of Radiation Medicine Chinese Academy of Medical Sciences. In addition, statistical analysis and abbreviations used have been reported^43^,^80^.

## Additional Information

**How to cite this article**: Qin, Q.-P. *et al.* Preparation of 6/8/11-Amino/Chloro-Oxoisoaporphine and Group-10 Metal Complexes and Evaluation of Their in Vitro and in Vivo Antitumor Activity. *Sci. Rep.* **6**, 37644; doi: 10.1038/srep37644 (2016).

**Publisher’s note:** Springer Nature remains neutral with regard to jurisdictional claims in published maps and institutional affiliations.

## Supplementary Material

Supplementary Information

## Figures and Tables

**Table 1:** IC_50_ (*μ*M) values of L^a^–L^d^, **1**–**14** and cisplatin toward on six cells for 48 h.

Compds	Hep-G2	SK-OV-3	BEL-7402	NCI-H460	HCT-8	HL-7702
L^a^	128.50 ± 1.32	141.90 ± 1.53	95.33 ± 1.09	112.52 ± 0.23	98.13 ± 1.21	108.43 ± 1.27
L^b^	141.21 ± 0.53	143.64 ± 1.24	94.75 ± 0.55	135.78 ± 1.29	101.19 ± 0.94	86.08 ± 2.07
**1**	8.24 ± 0.76	10.21 ± 1.23	20.70 ± 1.71	10.79 ± 2.01	22.74 ± 1.65	102.58 ± 0.31
**4**	18.16 ± 0.79	15.04 ± 0.92	32.17 ± 0.36	15.64 ± 0.83	46.31 ± 2.05	95.05 ± 1.87
**2**	15.22 ± 0.98	21.44 ± 1.48	59.04 ± 0.95	21.12 ± 0.45	65.34 ± 2.29	87.38 ± 2.02
**5**	28.09 ± 0.25	30.82 ± 0.31	67.01 ± 2.04	29.43 ± 0.57	70.23 ± 1.45	75.08 ± 0.99
**3**	4.61 ± 1.37	7.12 ± 0.75	12.32 ± 1.03	8.67 ± 0.83	14.17 ± 0.64	126.84 ± 1.17
**6**	14.25 ± 0.77	15.41 ± 0.83	25.02 ± 2.17	55.34 ± 0.45	20.87 ± 1.56	100.17 ± 0.85
L^c^	108.26 ± 0.55	154.09 ± 1.02	91.02 ± 2.03	167.06 ± 0.71	100.14 ± 0.98	115.16 ± 0.65
**7**	16.41 ± 0.59	31.02 ± 1.24	50.17 ± 0.85	28.47 ± 1.58	41.12 ± 1.36	76.64 ± 0.67
**8**	10.15 ± 0.99	27.72 ± 0.45	35.18 ± 0.94	20.11 ± 0.36	24.18 ± 0.74	84.42 ± 0.73
**9**	15.88 ± 2.11	30.44 ± 1.19	45.02 ± 0.86	25.14 ± 1.09	25.19 ± 1.82	80.01 ± 1.02
**10**	12.18 ± 1.26	13.24 ± 0.77	21.38 ± 0.96	12.27 ± 1.56	16.96 ± 2.07	86.14 ± 0.75
**11**	6.49 ± 1.04	11.02 ± 1.19	17.64 ± 1.46	9.56 ± 0.65	16.08 ± 1.25	90.18 ± 0.69
L^d^	60.62 ± 0.19	55.94 ± 0.51	88.06 ± 1.65	42.72 ± 0.80	132.16 ± 2.88	72.16 ± 2.05
**12**	28.76 ± 1.42	29.06 ± 0.82	50.02 ± 1.06	35.18 ± 1.49	66.18 ± 1.26	86.03 ± 1.24
**13**	33.54 ± 0.71	36.74 ± 0.60	70.15 ± 0.47	40.02 ± 0.99	85.04 ± 0.99	69.04 ± 0.89
**14**	22.77 ± 1.05	25.96 ± 0.57	33.06 ± 1.45	27.16 ± 0.92	36.42 ± 1.57	94.02 ± 0.53
Cisplatin	9.65 ± 0.87	14.06 ± 1.07	12.68 ± 0.94	18.06 ± 1.26	16.27 ± 1.54	12.35 ± 1.19

**Table 2:** FID assay for 1–6 interactions with DNA.

	1	2	3	4	5	6	7	10
^Pu27^DC_50_ (^ctDNA^DC_50_/^Pu27^DC_50_)	1.31 (45.90)	3.88 (4.80)	0.75 (130.49)	14.22 (6.21)	42.51 (1.82)	12.35 (7.45)	60.02 (1.67)	33.15 (2.98)
^HTG21^DC_50_ (^ctDNA^DC_50_/^HTG21^DC_50_)	3.35 (17.95)	3.91 (4.76)	1.52 (64.81)	26.45 (3.34)	49.08 (1.58)	19.87 (4.63)	70.83 (1.41)	55.16 (1.79)
^c-kit-2^DC_50_ (^ctDNA^DC_50_/^c-kit-2^DC_50_)	3.61 (16.66)	5.01 (3.71)	2.25 (43.50)	45.30 (1.95)	>100 (<0.77)	55.28 (1.66)	87.29 (1.15)	79.69 (1.24)
^c-kit-1^DC_50_ (^ctDNA^DC_50_/^c-kit-1^DC_50_)	9.11 (6.60)	18.75 (0.99)	2.33 (42.00)	73.65 (1.20)	78.46 (0.98)	71.64 (1.28)	90.02 (1.11)	79.95 (1.23)
^Pu22^DC_50_ (^ctDNA^DC_50_/^Pu22^DC_50_)	7.63 (7.88)	9.09 (2.05)	4.78 (20.47)	90.18 (0.98)	89.64 (0.86)	42.61 (2.16)	99.81 (1.01)	74.91 (1.32)
^ds26^DC_50_ (^ctDNA^DC_50_/^ds26^DC_50_)	37.01 (1.62)	21.76 (0.86)	23.03 (4.25)	70.46 (1.25)	90.63 (0.85)	57.71 (1.59)	96.11 (1.04)	91.48 (1.07)
^ctDNA^DC_50_ (^ctDNA^DC_50_/^ctDNA^DC_50_)	60.13 (1.00)	18.61 (1.00)	97.87 (1.00)	88.29 (1.00)	77.37 (1.00)	91.97 (1.00)	99.99 (1.00)	98.64 (1.00)

**Table 3:** DNA damage induced by complexes 1 (8 *μ*M), 2 (15 *μ*M), 3 (5 *μ*M), 4 (18 *μ*M), 5 (28 *μ*M) and 6 (14 *μ*M) on Hep-G2 cells detected by Comet assay.

	Comet length	Tail length	Tail moment	Olive tail moment
control	52.97 ± 7.92	1.07 ± 1.42	0.06 ± 0.13	0.06 ± 0.12
**1**	98.24 ± 8.32*	38.42 ± 7.93*	25.86 ± 5.37*	17.95 ± 6.58*
**4**	87.76 ± 7.65*	34.58 ± 9.72*	23.97 ± 6.9*	16.94 ± 5.21*
**2**	67.00 ± 7.99*	18.76 ± 4.68 *	10.78 ± 3.65*	7.63 ± 2.19*
**5**	53.29 ± 8.46	2.43 ± 1.01	0.97 ± 0.34	1.05 ± 0.58
**3**	115.27 ± 9.71**	45.76 ± 7.99**	38.97 ± 5.96**	21.95 ± 4.76**
**6**	98.54 ± 9.63*	30.47 ± 5.19*	31.39 ± 8.58*	20.52 ± 5.65*

^**^P < 0.01; *P < 0.05 comparing with control.

**Table 4:** Abbreviations, DNA oligomers and cell lines of used in this work.

G4	G-quadruplex
FID	fluorescent intercalator displacement
TO	thiazole orange
SD	standard deviation
T_m_	melting temperature
FRET	fluorescence resonance energy transfer
TBS	pH 7.35, 10 mM Tris-KCl-HCl buffer solution, containing 100 mM KCl
MTT	3-(4,5-dimethylthiazol-2-yl)-2,5-diphenyltetrazolium bromide
PI	propidium iodide
TAMRA	6-carboxytetramethylrhodamine
PBS	phosphate buffered saline
TRF	the duplex TTAGGG repeat-binding factors (such as TRF1 and TRF2)
FAM	6-carboxyfluorescein
hTERT	human telomerase reverse transcriptase
53BP1	p53 binding protein 1
RT-PCR	reverse transcription-polymerase chain reaction
Pu27	5′-TGGGGAGGGTGGGGAGGGTGGGGAAGG-3′
c-kit-2	5′-CGGGCGGGCGCTAGGGAGGGT-3′
c-kit-1	5′-CGGGCGGGCACGAGGGAGGGT-3′
HTG21	5′-GGGTTAGGGTTAGGGTTAGGG-3′
Pu39	5′-AGGGGCGGGCGCGGGAGGAAGGGGGCGGGAGCGGGGCTG-3′
ds26	5′-CAATCGGATCGAATTCGATCCGATTG-3′
Pu22	5′-TGAGGGTGGGTAGGGTGGGTAA-3′
F21T	5′-FAM-GGGCTAGGGCTAGGGCTAGGG-TAMRA-3′
FMidG4T	5′-FAM-CGGGCGCGGGAGGAAGGGGGCGGGAGC-TAMRA-3′
FPu18T	5′-FAM-AGGGTGGGGAGGGTGGGG-TAMRA-3′
H20M	5′-GCCAGTTCTTGAATGTAGAG-3′
F32T	5′-FAM-CCGCATCTCTACATTCAAGAACTGGCATGCGG-TAMRA-3′
bcl-2	Cx: 5′-GAGGATTGTGGCCTTCTTTG-3′
	Ts: 5′-GCCGGTTCAGGTACTCAGTC-3′
hTERT	Cx: 5′-CATCCACATAGAGGCCACCACGT-3′
	Ts: 5′-TGGTCTCCACGAGCCTCCGAGCG-3′
c-myc	Cx: 5′-GTGGCACCTCTTGAGGACCT-3′
	Ts: 5′-TGGTGCTCCATGAGGAGACA-3′
GAPDH	Cx: 5′-CGGAAGGCCATGCCT GTCAG-3′
	Ts: 5′-GCCTCTTGCACGACCAACTG-3′
Hep-G2	the hepatoblastoma cell line
NCI-H460	the non-small cell lung cancer cell line
SK-OV-3	the human ovarian cancer cell line
HCT-8	the human ileocecal adenocarcinoma cell line
BEL-7402	the human hepatoma cell line
HL-7702	an human normal hepatocytes cell line
