# Supplementary material for: Preparation of 6/8/11-Amino/Chloro-Oxoisoaporphine and Group-10 Metal Complexes and Evaluation of Their in Vitro and in Vivo Antitumor Activity
Source: Sci Rep. 2016 Nov 29;6:37644. doi: 10.1038/srep37644 (PMC5127189; doi:10.1038/srep37644)
Supplement: Supplementary Information [file srep37644-s1.pdf]

**Electronic Supporting Information Materials**

# Preparation of 6/8/11-Amino/Chloro-Oxoisoaporphine and Group-10 Metal Complexes and Evaluation of Their in Vitro and in Vivo Antitumor Activity

*Qi-Pin Qin[†], Jiao-Lan Qin[†], Ting Meng, Gui-Ai Yang, Zu-Zhuang Wei, Yan-Cheng Liu, Hong Liang*, Zhen-Feng Chen**

**Table S1** Selected bond lengths (Å) and bond angles (°) for L[a].

| | | | |
|---|---|---|---|
| O1-C14 | 1.247 (2) | C6-H6 | 0.9300 |
| N1-H1A | 0.8600 | C7-C8 | 1.457 (3) |
| N1-H1B | 0.8600 | C7-C16 | 1.420 (3) |
| N1-C1 | 1.334 (2) | C8-C9 | 1.401 (3) |
| N2-C6 | 1.340 (3) | C8-C13 | 1.407 (2) |
| N2-C7 | 1.341 (2) | C9-H9 | 0.9300 |
| C1-C2 | 1.430 (3) | C9-C10 | 1.369 (3) |
| C1-C15 | 1.418 (3) | C10-H10 | 0.9300 |
| C2-H2 | 0.9300 | C10-C11 | 1.380 (3) |
| C2-C3 | 1.346 (3) | C11-H11 | 0.9300 |
| C3-H3 | 0.9300 | C11-C12 | 1.367 (3) |
| C3-C4 | 1.429 (3) | C12-H12 | 0.9300 |
| C4-C5 | 1.396 (3) | C12-C13 | 1.395 (3) |
| C4-C16 | 1.412 (3) | C13-C14 | 1.482 (3) |
| C5-H5 | 0.9300 | C14-C15 | 1.441 (3) |
| C5-C6 | 1.366 (3) | C15-C16 | 1.427 (2) |
| H1A-N1-H1B | 120.0 | C9-C8-C13 | 118.65 (18) |
| C1-N1-H1A | 120.0 | C13-C8-C7 | 119.50 (17) |
| C1-N1-H1B | 120.0 | C8-C9-H9 | 119.7 |
| C6-N2-C7 | 118.29 (18) | C10-C9-C8 | 120.51 (19) |
| N1-C1-C2 | 118.08 (19) | C10-C9-H9 | 119.7 |
| N1-C1-C15 | 122.86 (19) | C9-C10-H10 | 119.7 |
| C15-C1-C2 | 119.03 (18) | C9-C10-C11 | 120.6 (2) |
| C1-C2-H2 | 119.2 | C11-C10-H10 | 119.7 |
| C3-C2-C1 | 121.68 (19) | C10-C11-H11 | 120.0 |
| C3-C2-H2 | 119.2 | C12-C11-C10 | 120.1 (2) |
| C2-C3-H3 | 119.5 | C12-C11-H11 | 120.0 |
| C2-C3-C4 | 121.09 (19) | C11-C12-H12 | 119.6 |
| C4-C3-H3 | 119.5 | C11-C12-C13 | 120.73 (19) |
| C5-C4-C3 | 122.59 (19) | C13-C12-H12 | 119.6 |
| C5-C4-C16 | 118.90 (18) | C8-C13-C14 | 121.40 (17) |

| | | | |
|---|---|---|---|
| C16-C4-C3 | 118.48 (19) | C12-C13-C8 | 119.33 (18) |
| C4-C5-H5 | 120.6 | C12-C13-C14 | 119.26 (17) |
| C6-C5-C4 | 118.73 (19) | O1-C14-C13 | 119.23 (18) |
| C6-C5-H5 | 120.6 | O1-C14-C15 | 123.11 (18) |
| N2-C6-C5 | 124.2 (2) | C15-C14-C13 | 117.65 (16) |
| N2-C6-H6 | 117.9 | C1-C15-C14 | 120.90 (17) |
| C5-C6-H6 | 117.9 | C1-C15-C16 | 118.76 (18) |
| N2-C7-C8 | 118.21 (17) | C16-C15-C14 | 120.33 (17) |
| N2-C7-C16 | 122.28 (17) | C4-C16-C7 | 117.55 (17) |
| C16-C7-C8 | 119.51 (16) | C4-C16-C15 | 120.91 (18) |
| C9-C8-C7 | 121.84 (17) | C7-C16-C15 | 121.53 (17) |

**Table S2.** Crystal data and structure refinement details for L[a].

| | |
|---|---|
| Empirical formula | $C_{16}H_{10}N_2O$ |
| Formula weight | 246.26 |
| Temperature/K | 293(2) |
| Crystal system | monoclinic |
| Space group | C2/c |
| a/Å, b/Å, c/Å | 18.0644(13), 7.9686(4), 17.1313(12) |
| α/°, β/°, γ/° | 90.00, 113.295(9), 90.00 |
| Volume/Å$^3$ | 2265.0(3) |
| Z | 8 |
| ρcalc/mg mm$^{-3}$ | 1.444 |
| μ/mm$^{-1}$ | 0.093 |
| F(000) | 1024 |
| Crystal size/mm$^3$ | 0.44 × 0.20 × 0.15 |
| 2Θ range for data collection | 5.82 to 52.74° |
| Index ranges | $-22 \le h \le 21$, $-9 \le k \le 9$, $-17 \le l \le 21$ |
| Reflections collected | 6047 |
| Independent reflections | 2315[R(int) = 0.0251] |
| Data/restraints/parameters | 2315/0/172 |
| Goodness-of-fit on F2 | 1.084 |
| Final R indexes [I>2σ (I)] | R1 = 0.0516, wR2 = 0.1245 |
| Final R indexes [all data] | R1 = 0.0831, wR2 = 0.1429 |
| Largest diff. peak/hole/e Å$^{-3}$ | 0.171/-0.183 |

[a] $R_1 = \Sigma\, ||F_o| - |F_c||/\Sigma|F_o|$; [b] $wR_2 = [\Sigma w(F_o^2 - F_c^2)^2/\Sigma w(F_o^2)^2]^{½}$.

**Table S3.** Selected bond lengths (Å) and bond angles (°) for **3.**

| | | | |
|---|---|---|---|
| Pt(1)–Cl(1) | 2.3208(19) | Pt(1)–N(1) | 1.950(5) |
| Pt(1)–S(1) | 2.1895(18) | Pt(1)–O(1) | 2.003(4) |
| S(1)–Pt(1)–Cl(1) | 91.87(7) | N(1)–Pt(1)–S(1) | 90.10(16) |
| O(1)–Pt(1)–Cl(1) | 177.85(15) | N(1)–Pt(1)–O(1) | 89.46(19) |
| N(1)–Pt(1)–Cl(1) | 177.83(17) | | |

**Table S4.** Crystal data and structure refinement details for **3**.

| | |
|---|---|
| Empirical formula | $C_{18}H_{16}ClN_2O_2PtS$ |
| Formula weight | 554.93 |
| Crystal system,Space group | Monoclinic, $P2_1/c$ |
| Unit Cell Dimensions | a=11.9868(3)Å , α=90.00°<br>b=19.3830(5)Å , β=92.130(3)°<br>c=7.6337(2)Å , γ=90.00° |
| Temperature(K) | 293(2) |
| Crystal size(mm$^3$) | 0.41 × 0.23 × 0.12 |
| Volume(Å$^3$) | 1772.39(8) |
| Z | 4 |
| $\rho_{calc}$(mg/mm$^{-3}$) | 2.020 |
| μ(mm$^{-1}$) | 8.197 |
| F(000) | 997 |
| 2θ range for data collection | 5.74 to 52.74° |
| Index ranges | $-14 \le h \le 14$, $-24 \le k \le 24$, $-9 \le l \le 9$ |
| Independent reflections | 3615[R(int) = 0.0449] |
| Reflections collected | 14382 |
| Data/restraints/parameters | 3615/0/237 |
| R(all data) | $R_1$ = 0.0500, $\omega R_2$ = 0.0869 |
| R[I>2σ(I)] | $R_1$ = 0.0373, $\omega R_2$ = 0.0810 |
| Goodness-of-fit on $F^2$ | 1.086 |
| Largest diff.peak/hole(e Å$^{-3}$) | 1.156 and −0.773 |

[a] $R_1 = \Sigma ||F_o| - |F_c||/\Sigma|F_o|$; [b] $wR_2 = [\Sigma w(F_o^2 - F_c^2)^2/\Sigma w(F_o^2)^2]^{½}$.

**Table S5.** Selected bond lengths (Å) and bond angles (°) for **7.**

| | | | |
|---|---|---|---|
| Pt1-Cl1 | 2.381(2) | S1-Pt1-Cl1 | 88.72(8) |
| Pt1-S1 | 2.212(2) | N1-Pt1-S1 | 177.76(16) |
| Pt1-N1 | 2.056(6) | C11-Pt1-Cl1 | 173.5(2) |
| Pt1-C11 | 2.013(7) | C11-Pt1-S1 | 97.8(2) |
| N1-Pt1-Cl1 | 92.20(18) | C11-Pt1-N1 | 81.3(3) |

**Table S6.** Crystal data and structure refinement details for **7**.

| | |
|---|---|
| Identification code | |
| Empirical formula | $C_{18}H_{13}Cl_2NO_2PtS$ |
| Formula weight | 573.34 |
| Temperature/K | 293(2) |
| Crystal system | orthorhombic |
| Space group | Pbca |
| a/Å | 7.1678(3) |
| b/Å | 20.161(4) |
| c/Å | 23.922(3) |
| α/° | 90.00 |
| β/° | 90.00 |
| γ/° | 90.00 |
| Volume/Å$^3$ | 3457.0(8) |
| Z | 8 |
| $\rho_{calc}$mg/mm$^3$ | 2.203 |
| m/mm$^{-1}$ | 8.560 |
| F(000) | 2175.0 |
| Crystal size/mm$^3$ | 0.41 × 0.23 × 0.12 |
| 2Θ range for data collection | 6.82 to 52.74° |
| Index ranges | $-6 \le h \le 8$, $-25 \le k \le 23$, $-15 \le l \le 29$ |
| Reflections collected | 8806 |
| Independent reflections | 3522[R(int) = 0.0602] |
| Data/restraints/parameters | 3522/0/228 |
| Goodness-of-fit on F$^2$ | 1.045 |
| Final R indexes [I>=2σ (I)] | $R_1 = 0.0452$, $wR_2 = 0.1014$ |
| Final R indexes [all data] | $R_1 = 0.0626$, $wR_2 = 0.1149$ |
| Largest diff. peak/hole / e Å$^{-3}$ | 2.59/-2.15 |

[a] $R_1 = \Sigma ||F_o| - |F_c||/\Sigma|F_o|$; [b] $wR_2 = [\Sigma w(F_o^2 - F_c^2)^2/\Sigma w(F_o^2)^2]^{1/2}$.

**Table S7.** Selected bond lengths (Å) and bond angles (°) for **10.**

| | | | |
|---|---|---|---|
| Pt1-Cl2 | 2.3826(11) | C1-Pt1-N1 | 80.87(18) |
| Pt1-S1 | 2.2156(13) | C1-Pt1-S1 | 97.93(15) |
| Pt1-N1 | 2.064(4) | C1-Pt1-Cl2 | 173.16(15) |
| Pt1-C1 | 2.014(5) | N1-Pt1-S1 | 177.05(11) |
| Pt2-Cl4 | 2.3906(11) | N1-Pt1-Cl2 | 92.78(11) |
| Pt2-S2 | 2.2156(13) | S1-Pt1-Cl2 | 88.53(4) |
| Pt2-N2 | 2.060(4) | S2-Pt2-Cl4 | 88.70(4) |
| Pt2-C20 | 2.014(5) | N2-Pt2-Cl4 | 92.70(11) |
| Pt3-Cl6 | 2.3931(12) | N2-Pt2-S2 | 175.88(11) |
| Pt3-S3 | 2.2162(12) | C20-Pt2-Cl4 | 172.14(15) |
| Pt3-N3 | 2.060(4) | C20-Pt2-S2 | 98.01(15) |
| Pt3-C39 | 2.003(5) | C20-Pt2-N2 | 80.89(18) |
| Pt4-Cl8 | 2.3845(12) | S3-Pt3-Cl6 | 89.30(4) |
| Pt4-S4 | 2.2144(12) | N3-Pt3-Cl6 | 91.82(12) |

**Table S8.** Crystal data and structure refinement details for **10**.

| | |
|---|---|
| Empirical formula | $C_{76}H_{61}Cl_8N_4O_8Pt_4S_4$ |
| Formula weight | 2350.49 |
| Temperature/K | 150.00(14) |
| Crystal system | monoclinic |
| Space group | $C_{2/c}$ |
| a/Å, b/Å, c/Å | 40.6850(4), 23.2428(3), 14.14577(18) |
| α/°, β/°, γ/° | 90.00, 93.6963(11), 90.00 |
| Volume/Å$^3$ | 13348.9(3) |
| Z | 8 |
| ρcalc/mg mm$^{-3}$ | 2.339 |
| μ/mm$^{-1}$ | 8.870 |
| F(000) | 8968 |
| Crystal size/mm$^3$ | 0.22 × 0.15 × 0.08 |
| 2Θ range for data collection | 6.02 to 52.74° |
| Index ranges | $-50 \leq h \leq 50$, $-29 \leq k \leq 28$, $-15 \leq l \leq 17$ |
| Reflections collected | 35465 |
| Independent reflections | 13617[R(int) = 0.0247] |
| Data/restraints/parameters | 13617/1/948 |
| Goodness-of-fit on F2 | 1.109 |
| Final R indexes [I>2σ (I)] | R1 = 0.0302, wR2 = 0.0786 |
| Final R indexes [all data] | R1 = 0.0354, wR2 = 0.0818 |
| Largest diff. peak/hole/e Å$^{-3}$ | 1.607/-1.888 |

**Table S9.** Inhibitory rates (%) of $L^a$–$L^d$, **1–14**, the corresponding salts and cisplatin toward on the selected cells for 48 h.

| Compounds | Hep-G2 | SK-OV-3 | BEL-7402 | NCI-H460 | HCT-8 | HL-7702 |
|---|---|---|---|---|---|---|
| $L^{a}$ [a] | 28.99±1.35 | 20.38±1.41 | 30.22±1.27 | 25.38±0.33 | 30.54±1.25 | 29.15±1.44 |
| **1** [a] | 70.71±0.54 | 68.34±1.37 | 48.36±1.76 | 61.34±1.94 | 45.13±1.69 | 28.33±0.42 |
| **2**[a] | 58.22±1.02 | 48.72±1.52 | 35.16±0.84 | 47.37±0.59 | 35.88±2.23 | 35.05±2.04 |
| **3**[a] | 75.33±1.42 | 72.66±0.82 | 60.96±1.01 | 78.73±0.76 | 57.04±0.73 | 22.71±1.18 |
| $L^{b}$ [a] | 22.05±0.42 | 20.34±1.21 | 29.51±0.63 | 23.85±1.36 | 25.01±1.08 | 35.07±2.14 |
| **4** [a] | 55.01±0.66 | 55.84±1.03 | 39.01±0.26 | 56.07±1.02 | 38.22±1.47 | 30.17±1.77 |
| **5** [a] | 40.33±0.34 | 35.04±0.57 | 31.84±1.72 | 45.06±0.79 | 30.28±1.94 | 38.07±0.82 |
| **6** [a] | 57.33±0.84 | 54.39±1.77 | 45.02±1.97 | 38.04±0.44 | 49.11±1.73 | 30.88±0.92 |
| **$L^{c}$ [a]** | 31.02±0.58 | 18.53±1.05 | 35.14±2.01 | 20.19±0.67 | 29.87±1.01 | 27.85±0.58 |
| **7** | 56.14±0.61 | 33.89±1.26 | 39.44±0.83 | 45.98±1.51 | 30.52±1.39 | 37.52±0.65 |
| **8** | 62.88±1.02 | 37.86±0.42 | 42.18±0.96 | 49.33±0.35 | 42.65±0.70 | 37.11±0.69 |
| **9** | 58.11±2.05 | 36.74±1.21 | 40.64±0.85 | 47.28±1.07 | 40.89±1.79 | 39.56±1.01 |
| **10** | 60.51±1.21 | 58.04±0.78 | 47.36±0.99 | 59.02±1.49 | 55.62±2.04 | 35.42±0.73 |
| **11** | 72.52±1.03 | 61.12±1.17 | 58.45±1.42 | 62.35±0.58 | 58.87±1.28 | 33.74±0.61 |
| **$L^{d}$ [a]** | 38.47±0.32 | 32.58±0.38 | 32.12±1.58 | 42.03±0.94 | 25.02±2.16 | 39.33±1.76 |
| **12** [a] | 40.08±1.29 | 40.82±0.76 | 39.75±0.93 | 45.17±1.23 | 34.89±1.93 | 30.05±1.17 |
| **13** [a] | 39.64±0.85 | 36.42±0.87 | 38.25±0.59 | 43.94±1.09 | 32.77±0.84 | 40.11±1.04 |
| **14** [a] | 43.25±1.14 | 41.13±0.64 | 42.19±1.33 | 46.37±0.83 | 44.01±1.66 | 29.78±0.66 |
| en | 13.64±2.03 | 18.42±0.98 | 17.01±0.49 | 20.58±1.66 | 16.45±1.92 | 15.03±0.65 |
| pn | 15.18±1.74 | 21.24±0.54 | 16.76±1.09 | 18.08±0.39 | 10.19±0.82 | 14.19±0.73 |
| $NiCl_2 \cdot 6H_2O$ [b] | 15.06±1.09 | 20.88±0.61 | 11.28±1.08 | 10.44±1.82 | 12.75±1.09 | 17.03±1.16 |
| $PdCl_2$ [b] | 20.14±1.18 | 19.02±1.04 | 18.59±1.77 | 9.91±1.49 | 15.05±1.84 | 18.24±1.33 |
| cis-Pt(DMSO)$_2$Cl$_2$ [b] | 17.83±0.57 | 15.11±1.17 | 10.74±0.95 | no activity | no activity | no activity |
| cisplatin [c] | 68.71±0.56 | 58.06±1.03 | 65.82±1.11 | 59.13± 1.35 | 56.07± 1.67 | 63.09±1.35 |

Results represent mean ± SD of at least five independent experiments. SD represents the standard deviation. [a] The concentration is 20 μM. [b] The

concentration is 100 μM. [c] Cisplatin was dissolved at a concentration of 1 mM in 0.154 M NaCl.

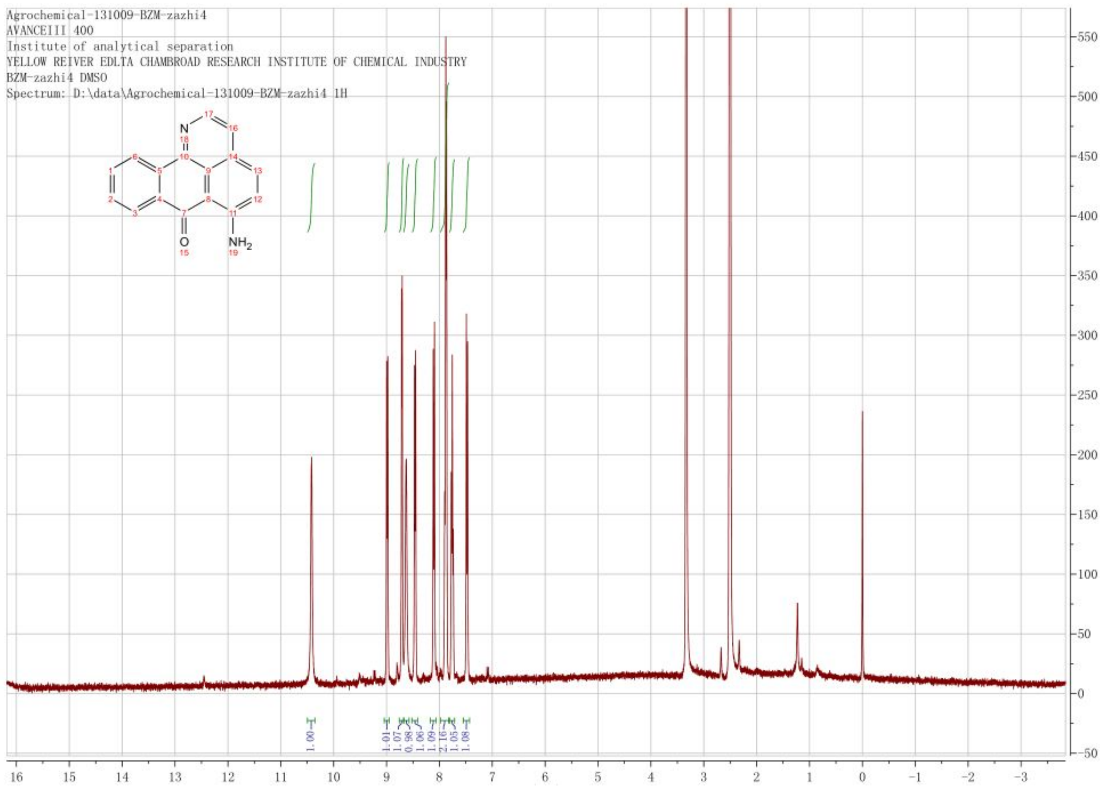


**Figure S1.** $^1$H NMR (500MHz, DMSO-d$_6$) for **L**[a]

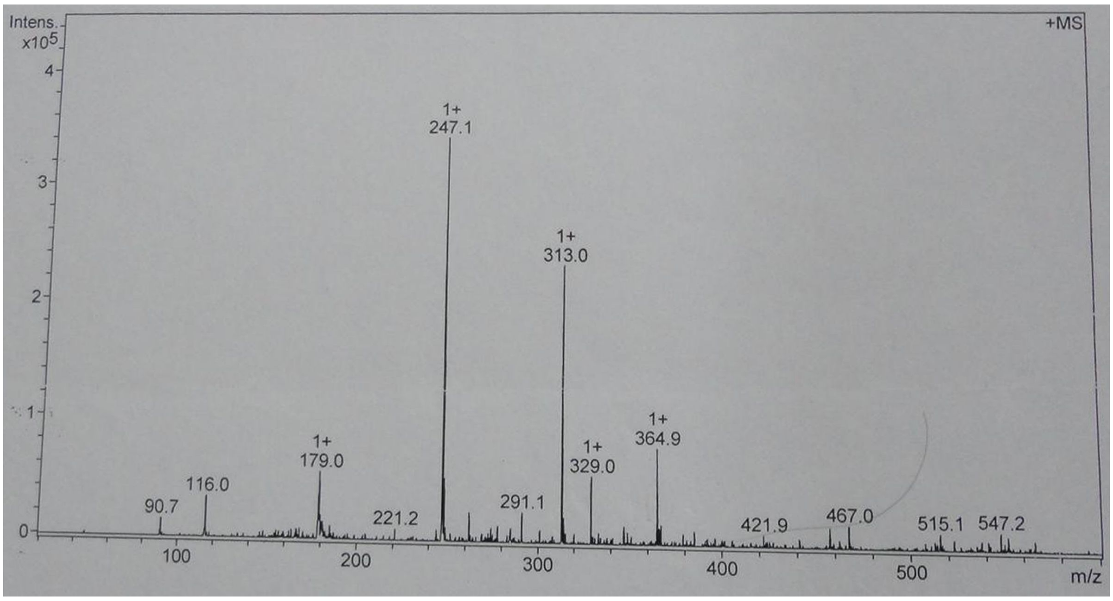


**Figure S2**. MS-EI spectra of **L**[a]

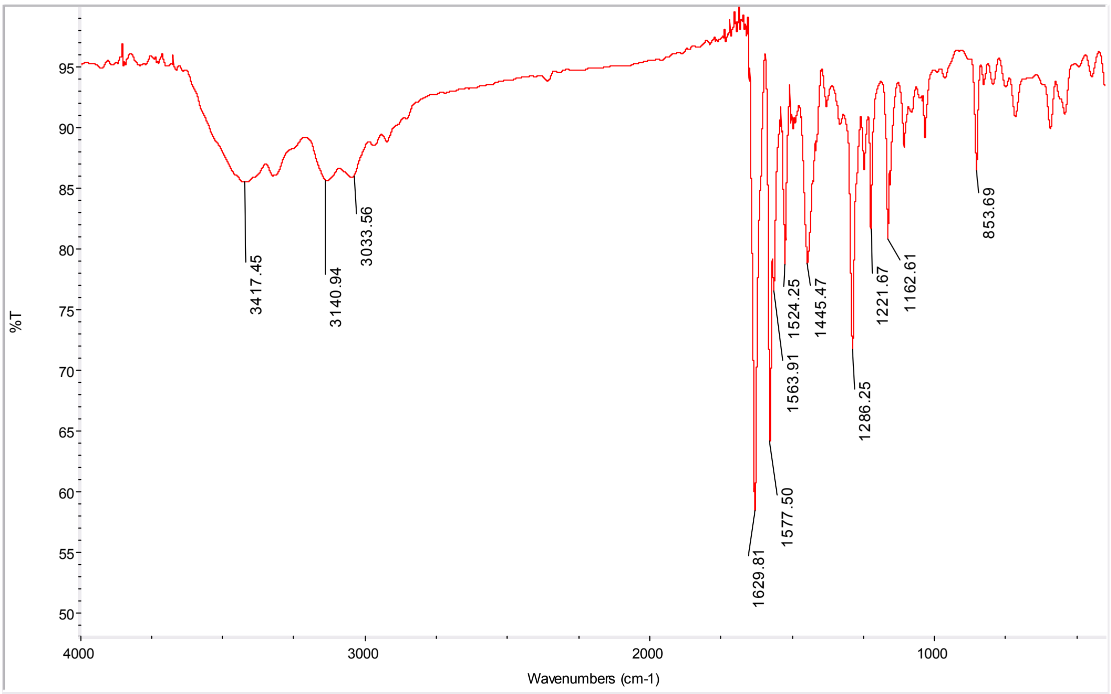


**Figure S3.** IR (KBr) spectra of **1**

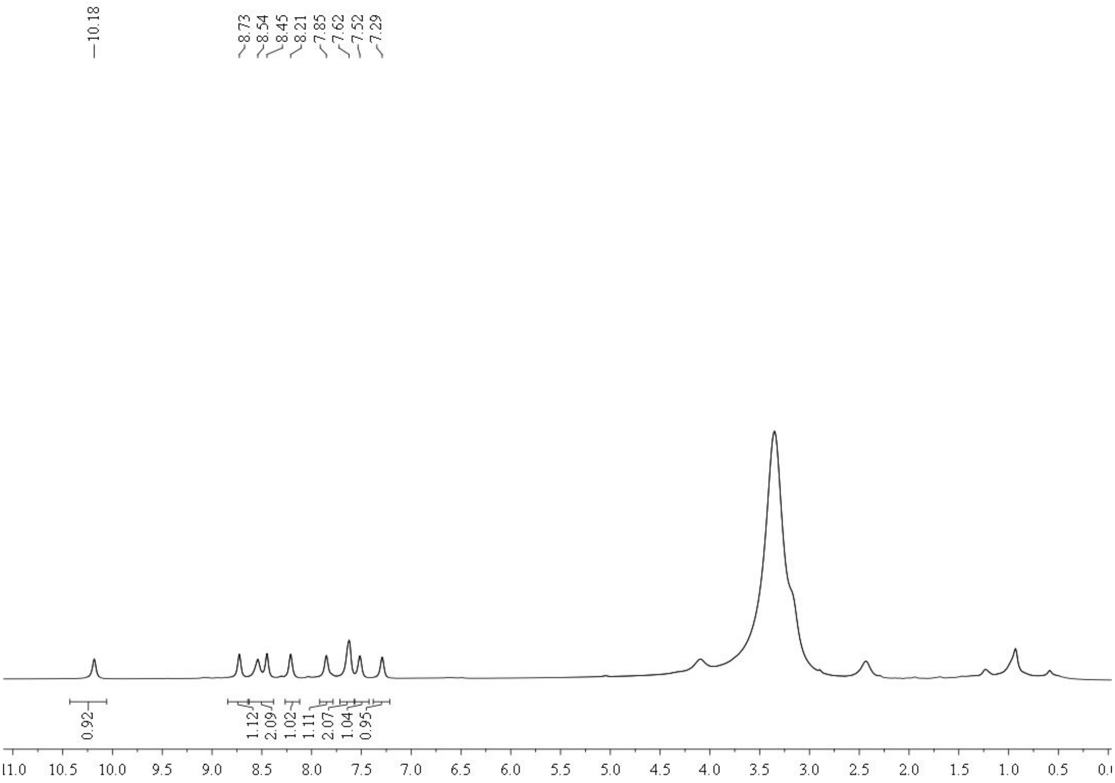


**Figure S4.** $^{1}$H NMR (600MHz, DMSO-$d_6$) for **1**

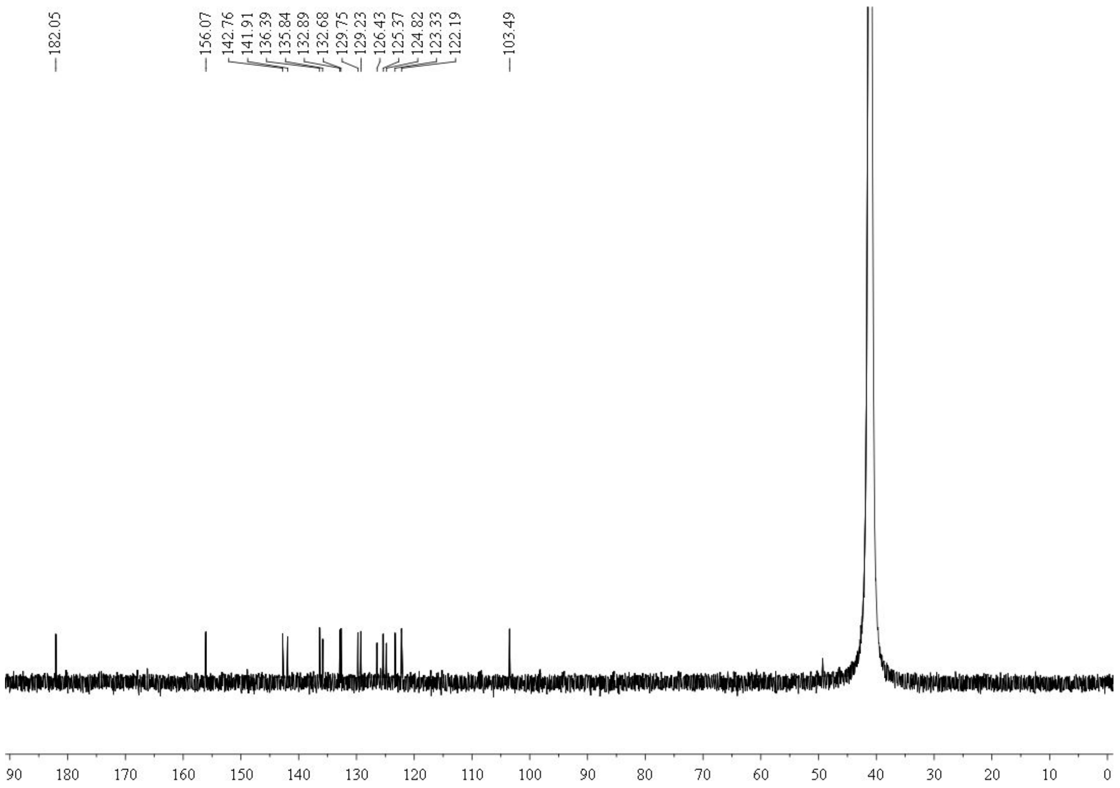


**Figure S5.** $^{13}C$ NMR (600MHz, DMSO-$d_6$) for **1**

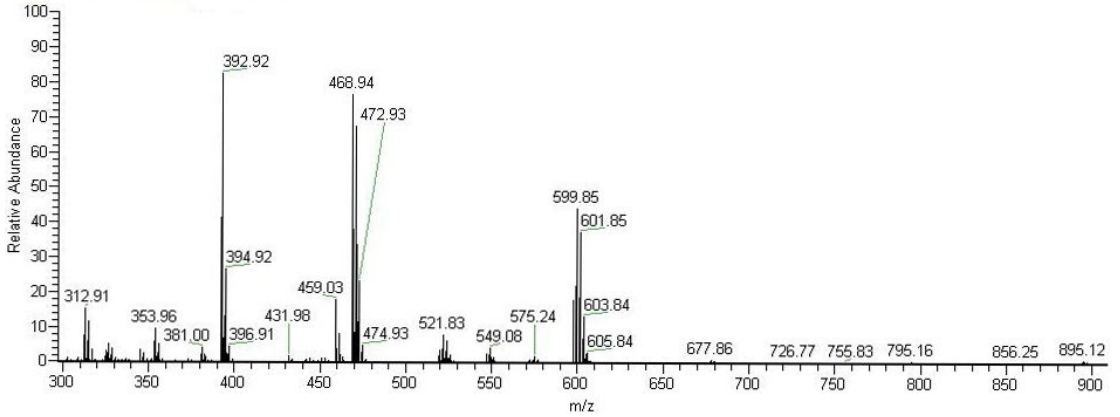


**Figure S6**. MS-EI spectra of **1**

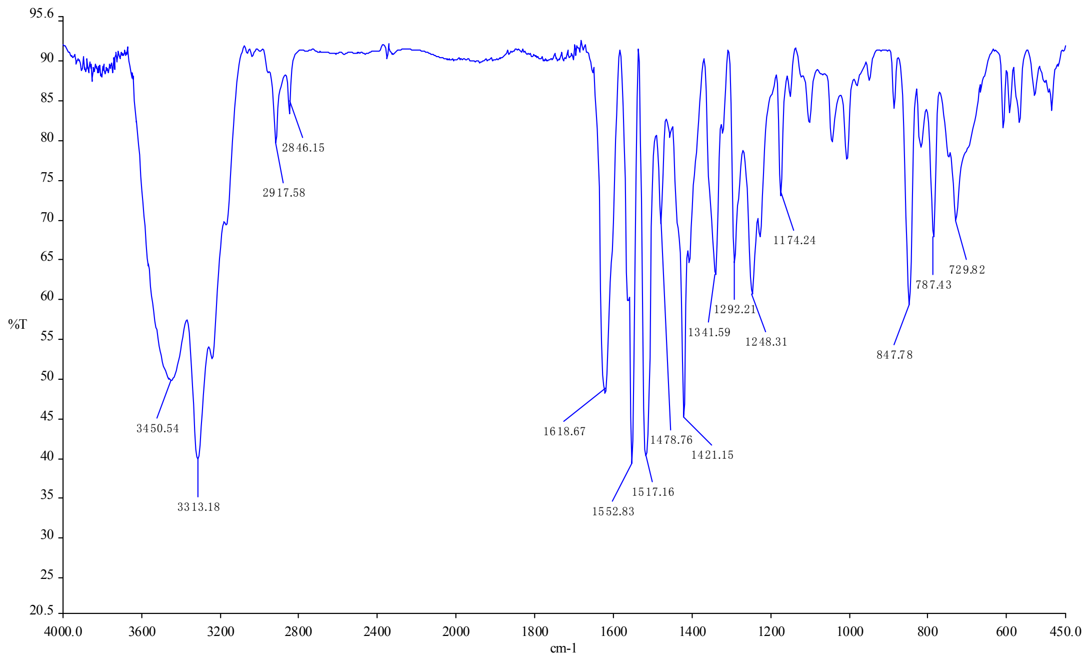


**Figure S7.** IR (KBr) spectra of **2**

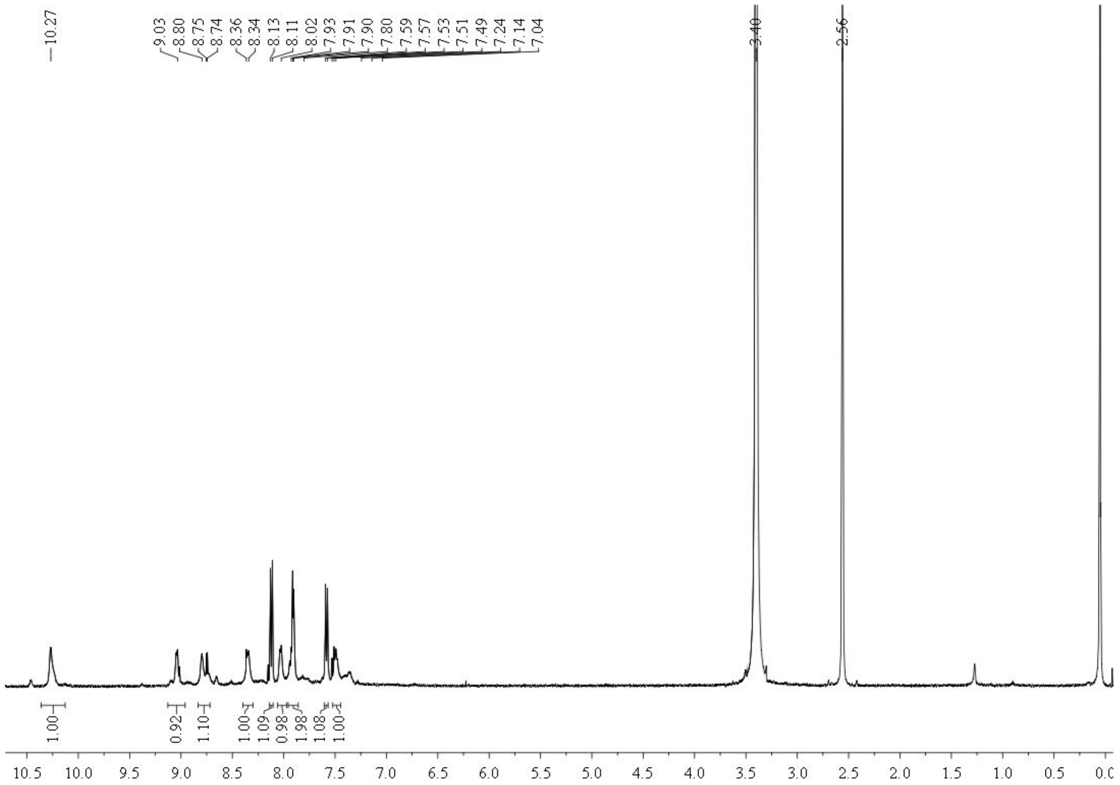


**Figure S8.** $^1$H NMR (600MHz, DMSO-$d_6$) for **2**

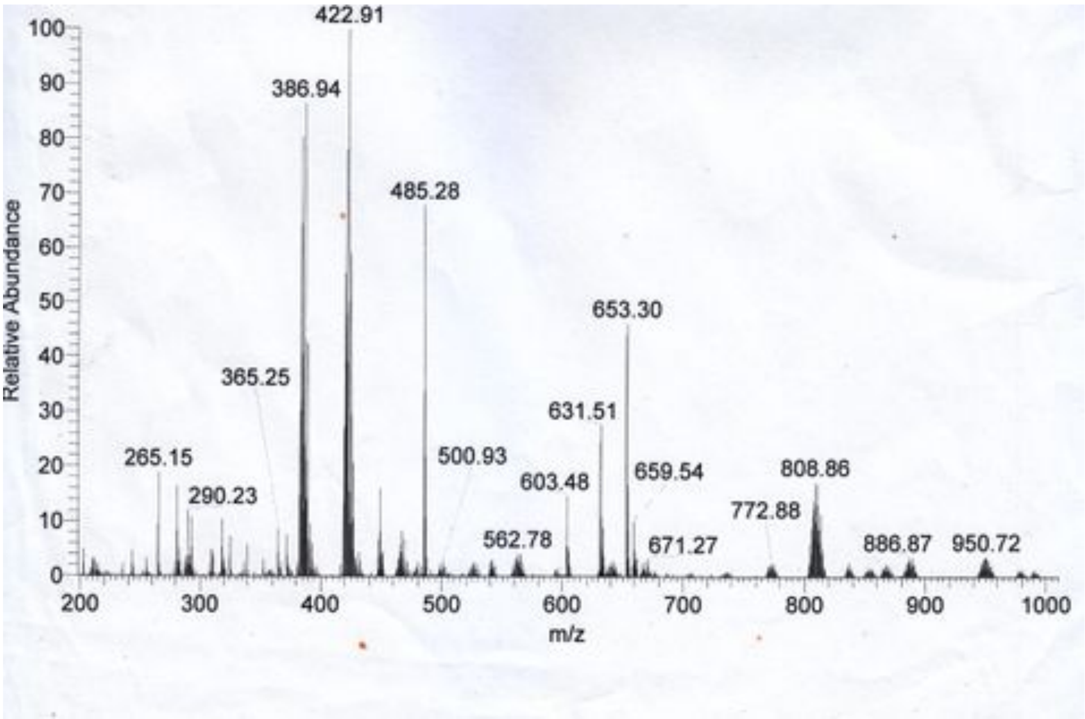


**Figure S9**. MS-EI spectra of **2**

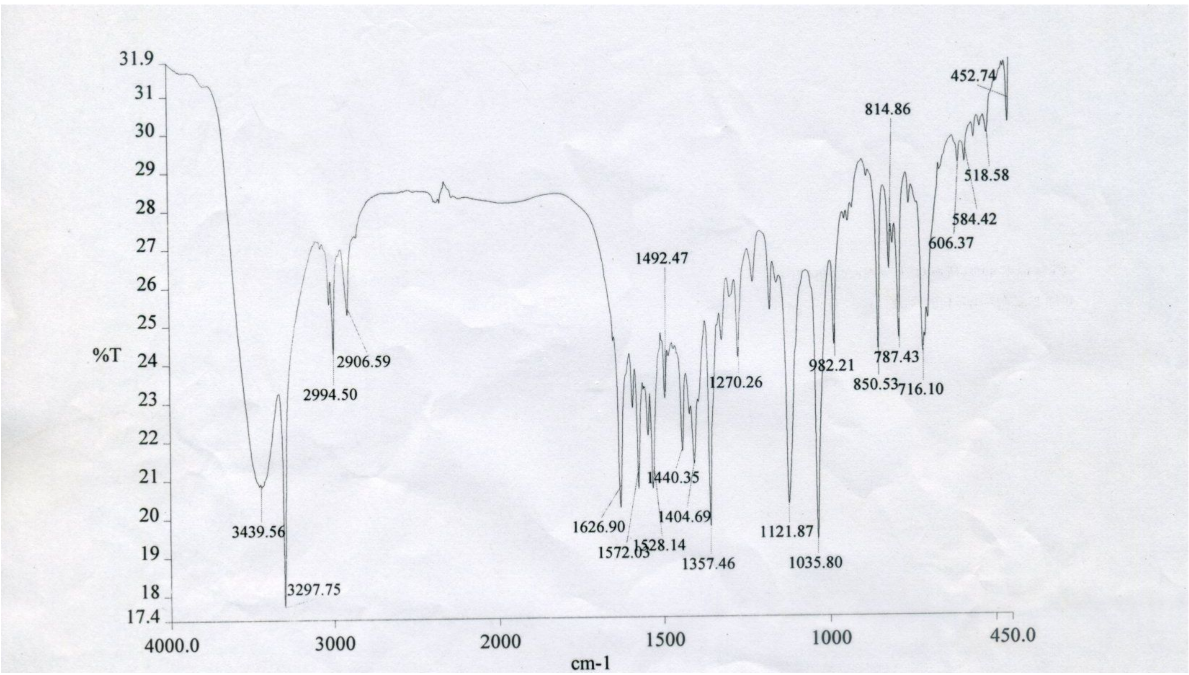


**Figure S10.** IR (KBr) spectra of **3**

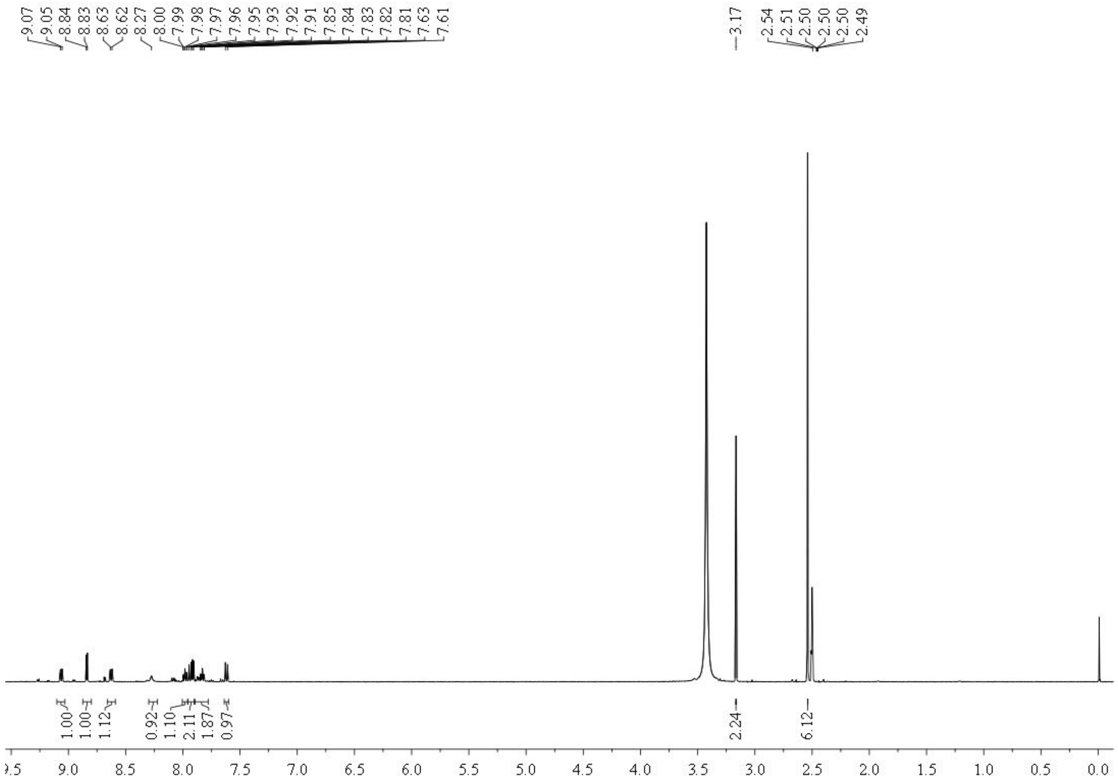


**Figure S11.** $^{1}$H NMR (600MHz, DMSO-$d_6$) for **3**

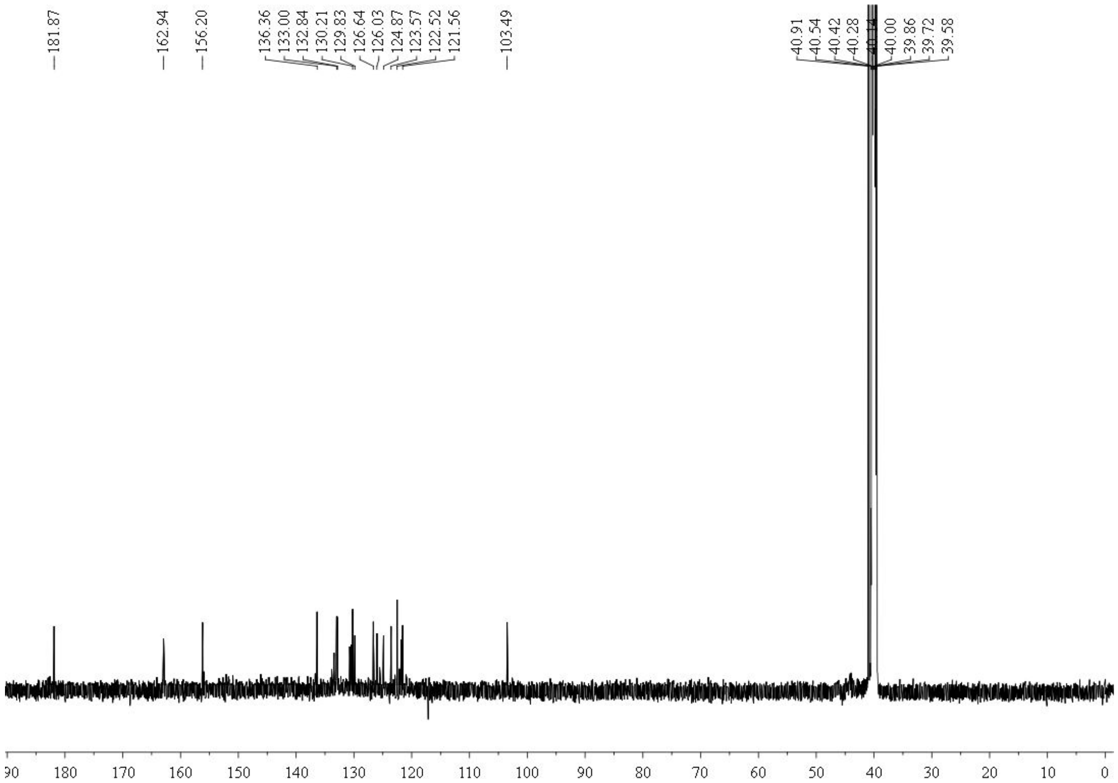


**Figure S12.** $^{13}$C NMR (600MHz, DMSO-$d_6$) for **3**

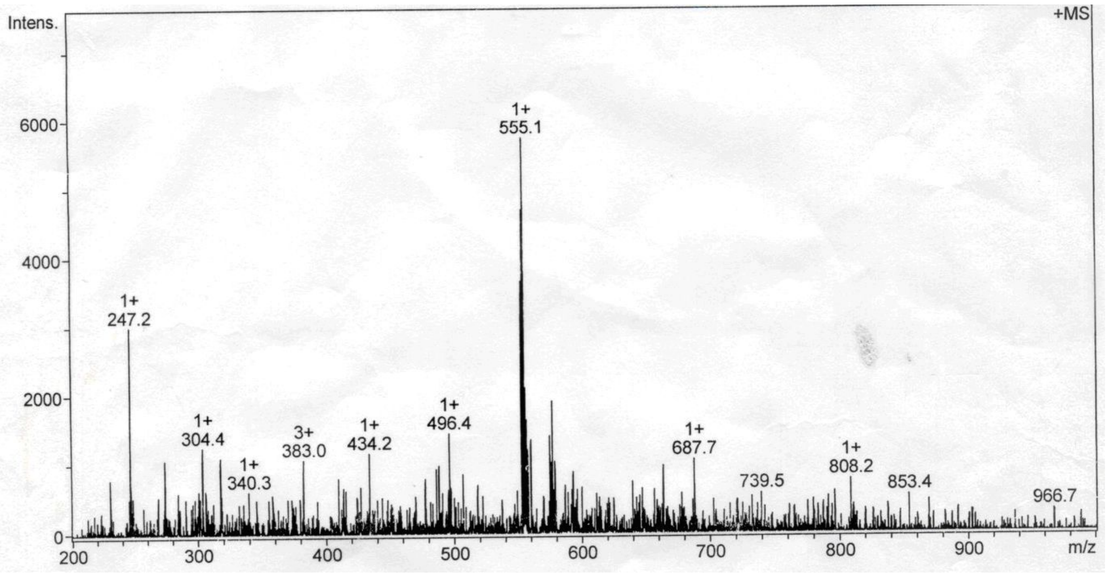


**Figure S13**. MS-EI spectra of **3**

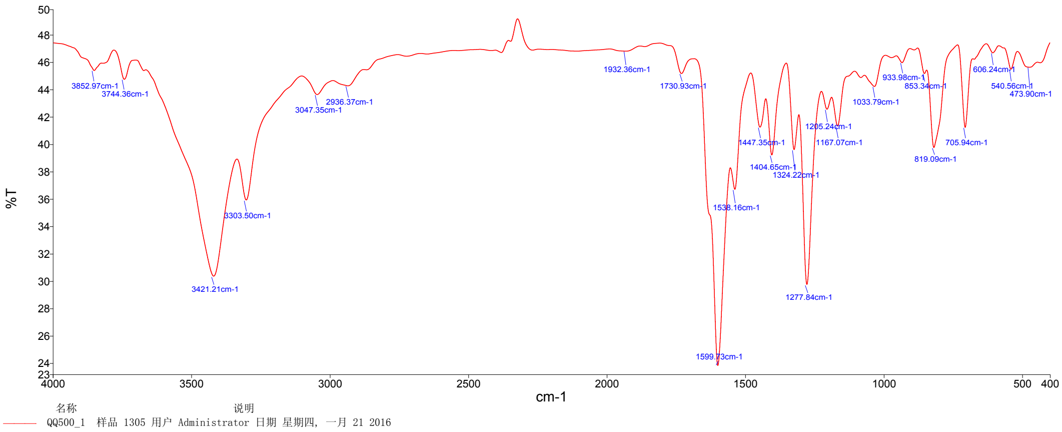


**Figure S14.** IR (KBr) spectra of $\mathbf{L^b}$

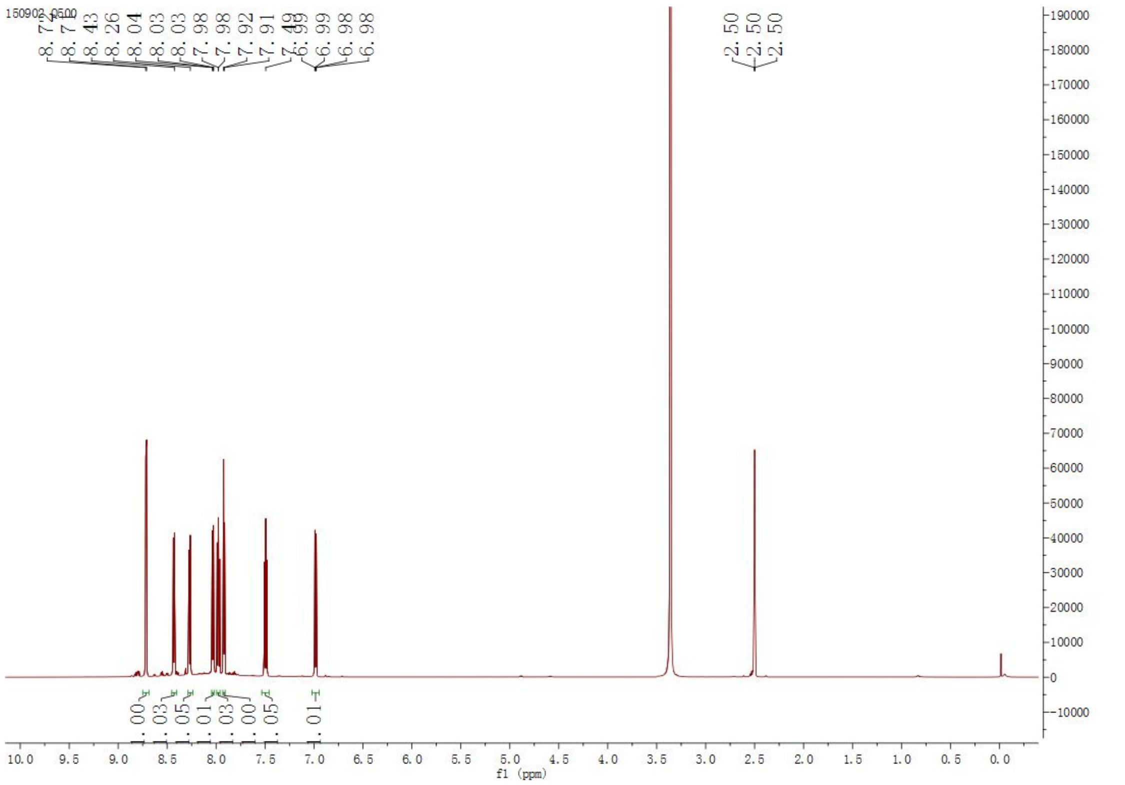

**Figure S15.** $^{1}H$ NMR (600MHz, DMSO-d$_6$) for $\mathbf{L^b}$

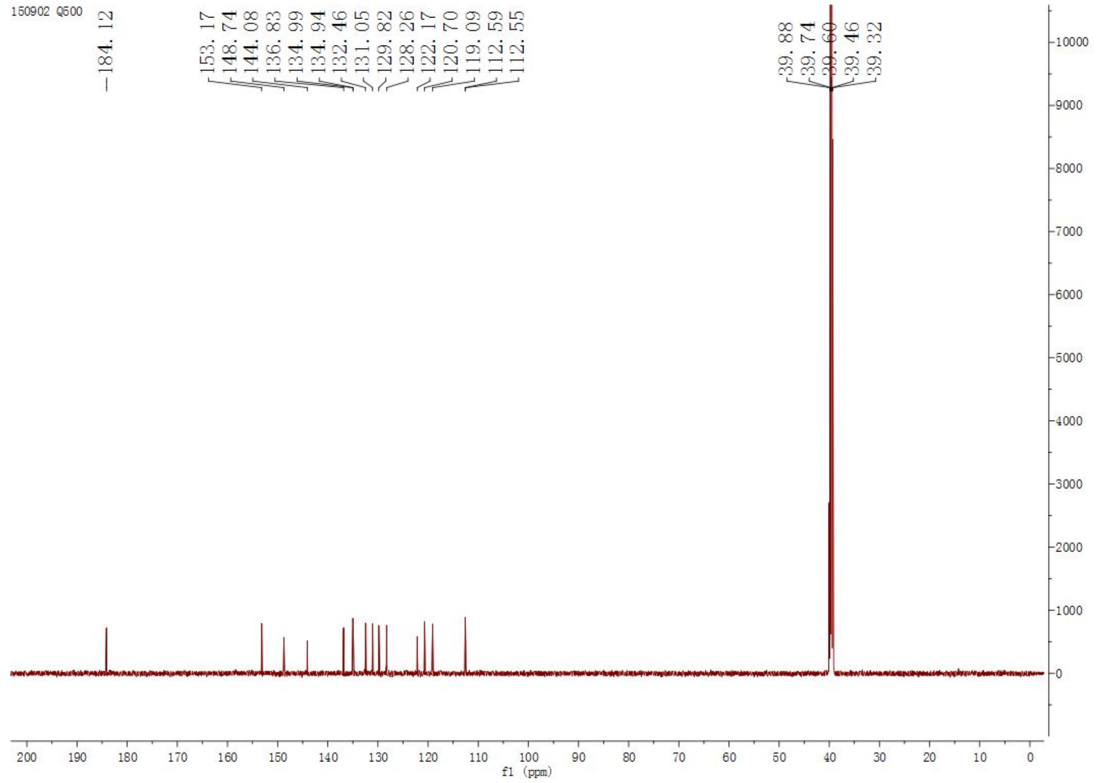

**Figure S16.** $^{13}C$ NMR (600MHz, DMSO-d$_6$) for $\mathbf{L^b}$

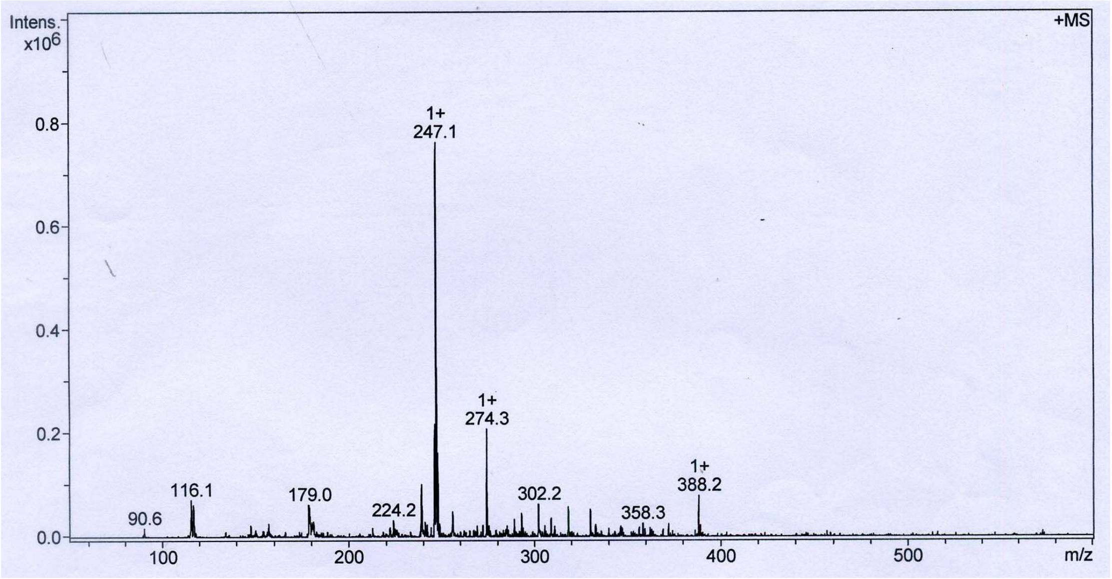


**Figure S17.** MS-EI spectra of $\mathbf{L^b}$

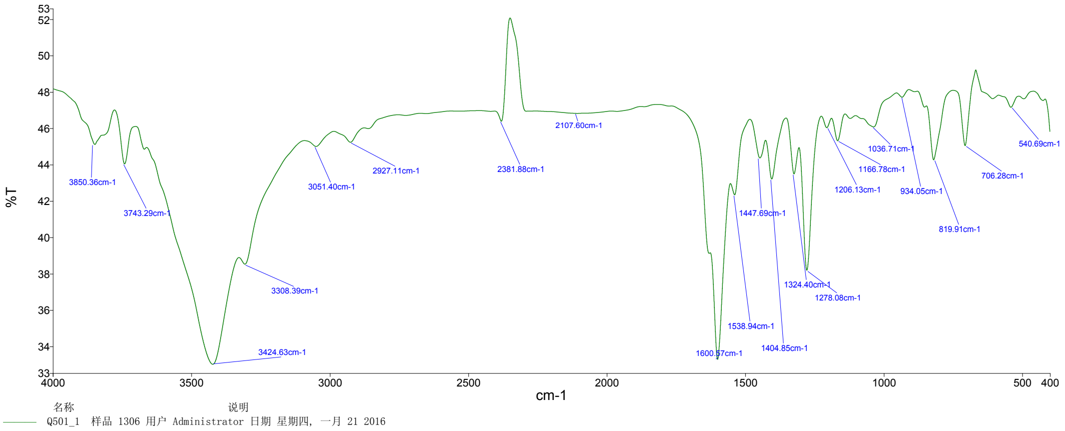


**Figure S18.** IR (KBr) spectra of **4**

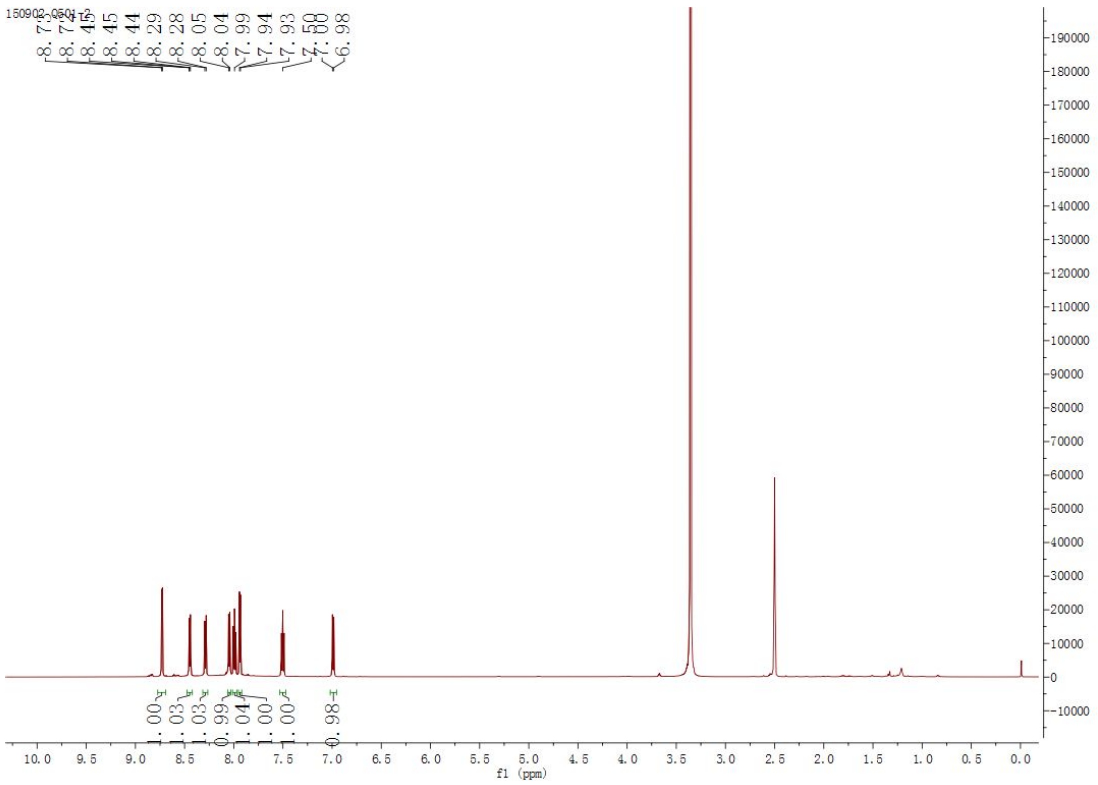


**Figure S19.** $^{1}$H NMR (600MHz, DMSO-$d_6$) for **4**

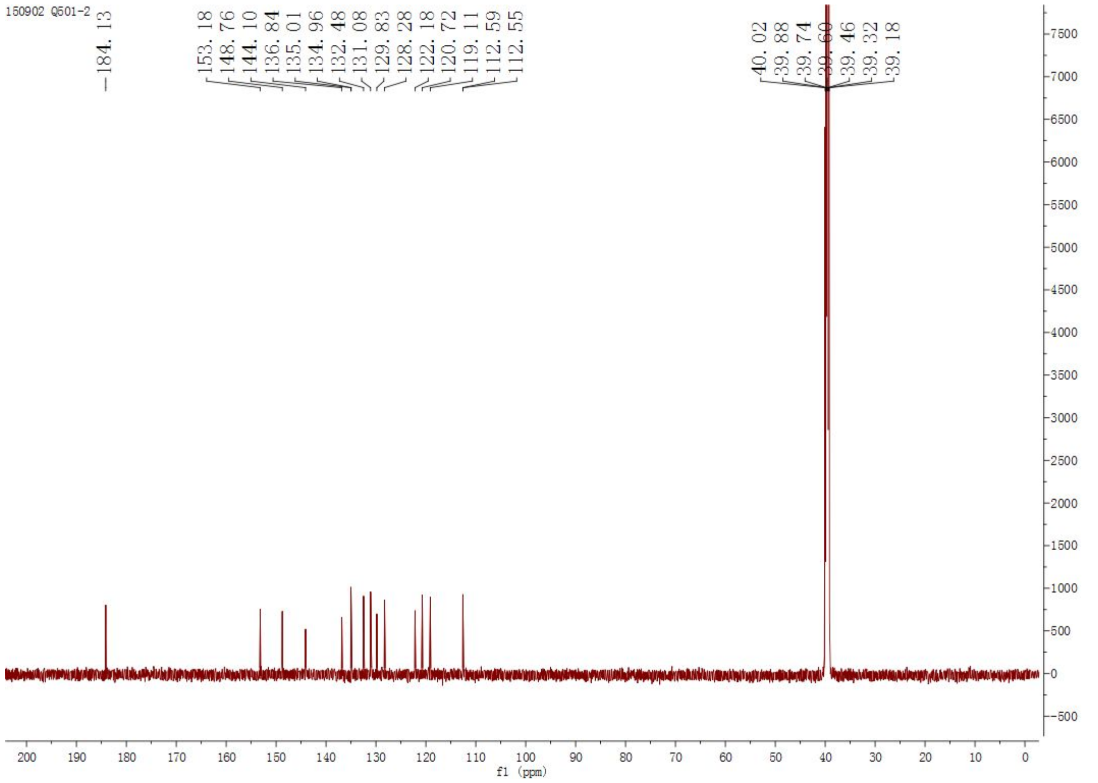


**Figure S20.** $^{13}$C NMR (600MHz, DMSO-$d_6$) for **4**

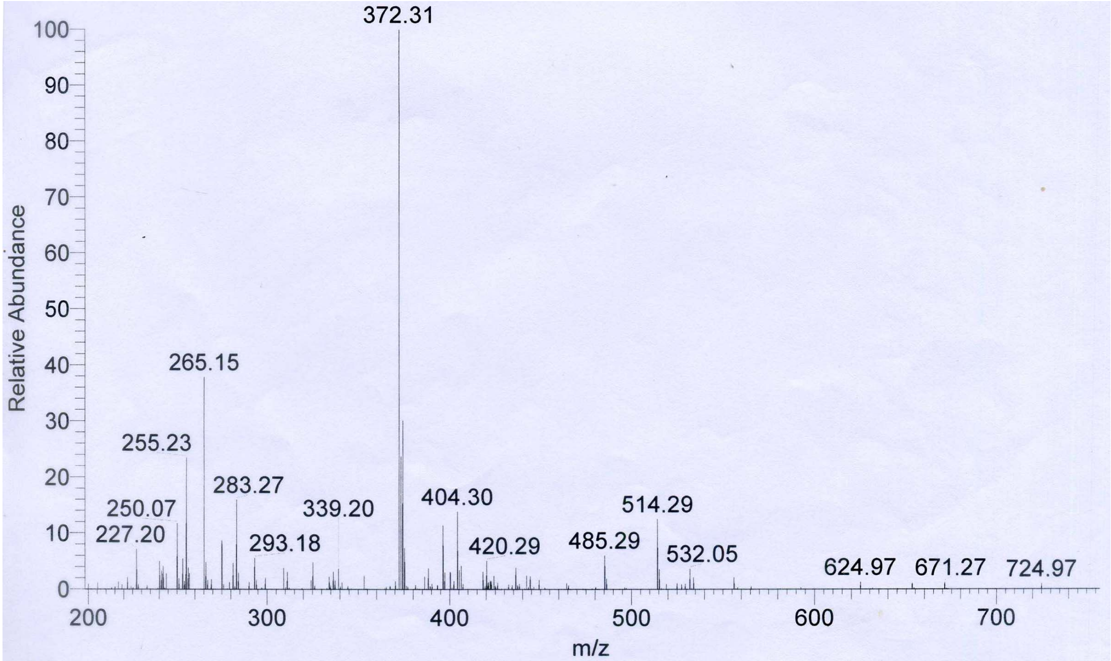


**Figure S21.** MS-EI spectra of **4**

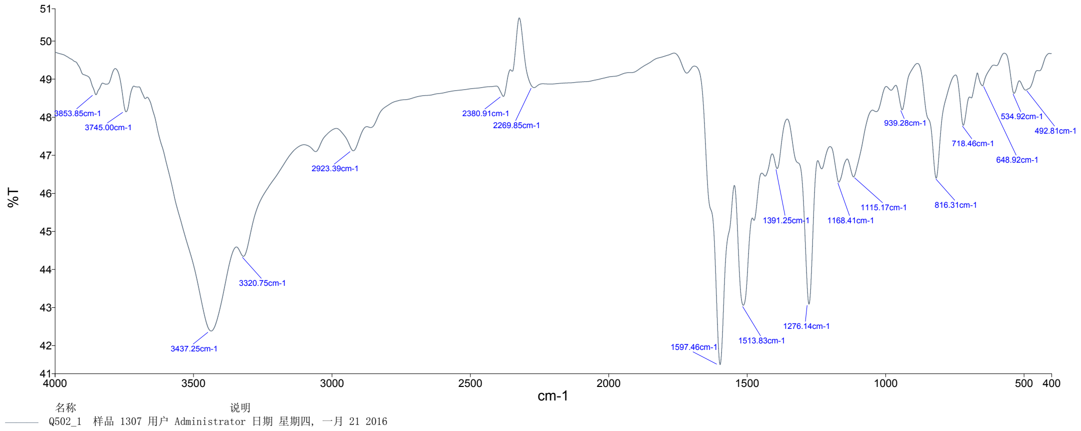


**Figure S22.** IR (KBr) spectra of **5**

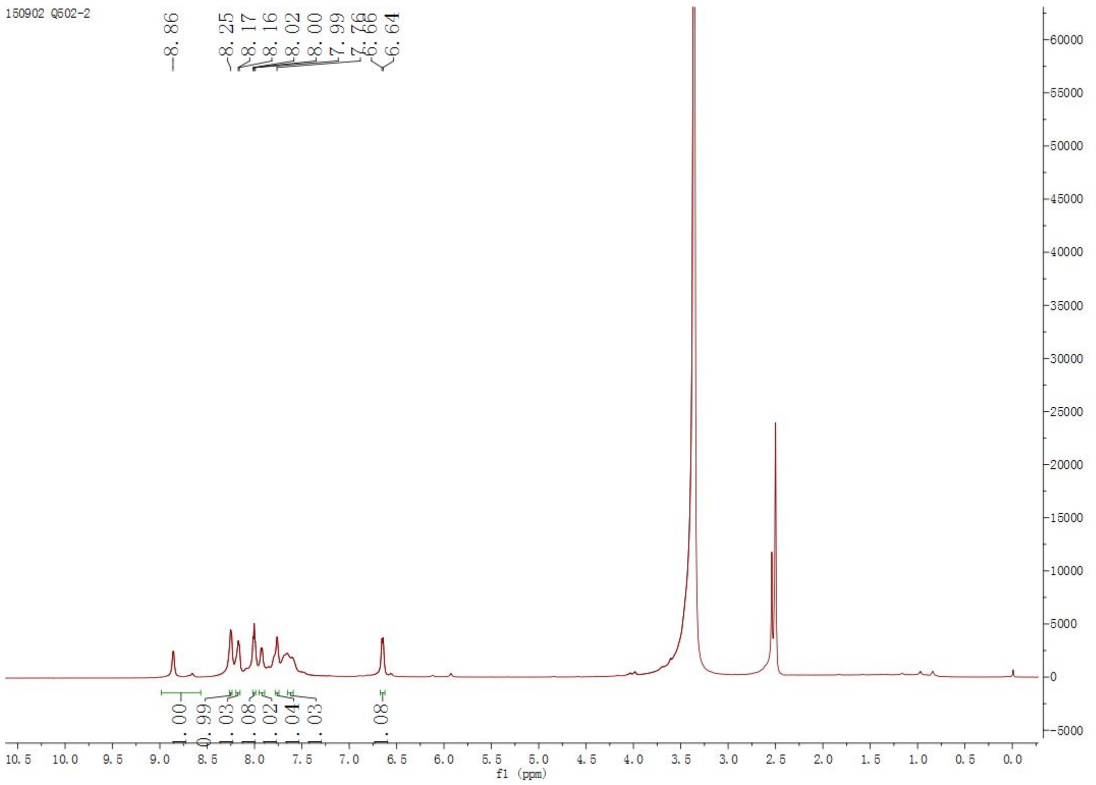


**Figure S23.** $^{1}$H NMR (600MHz, DMSO-$d_6$) for **5**

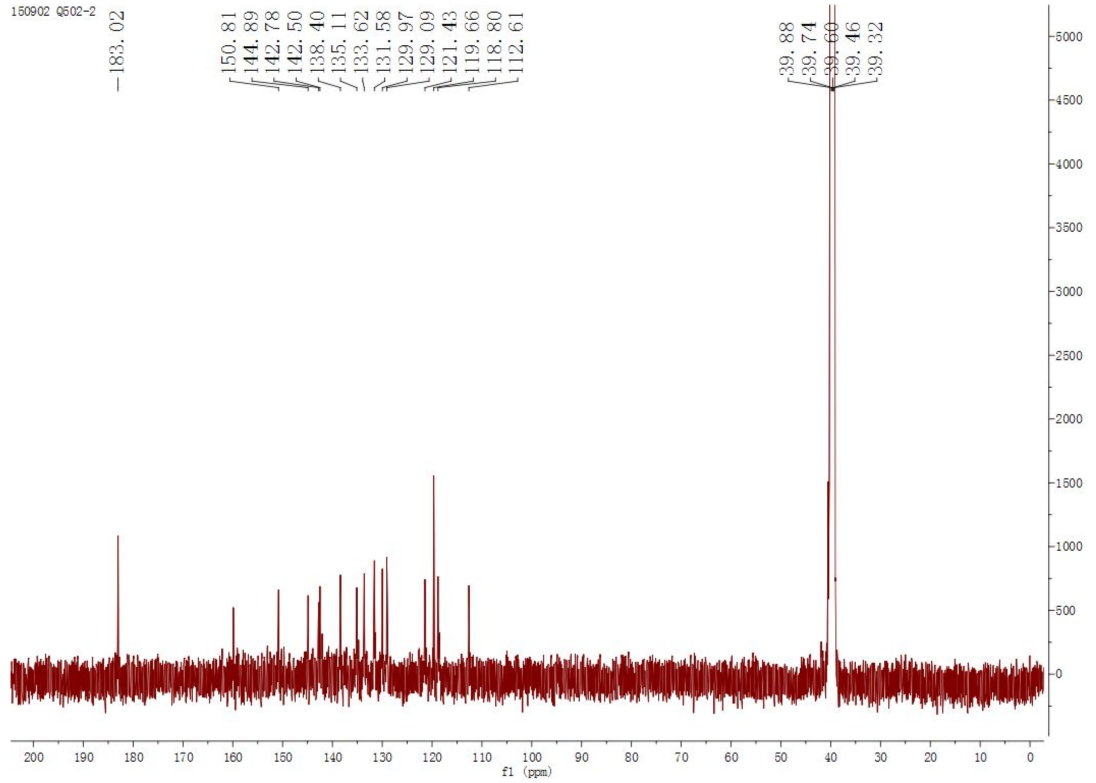


**Figure S24.** $^{13}$C NMR (600MHz, DMSO-$d_6$) for **5**

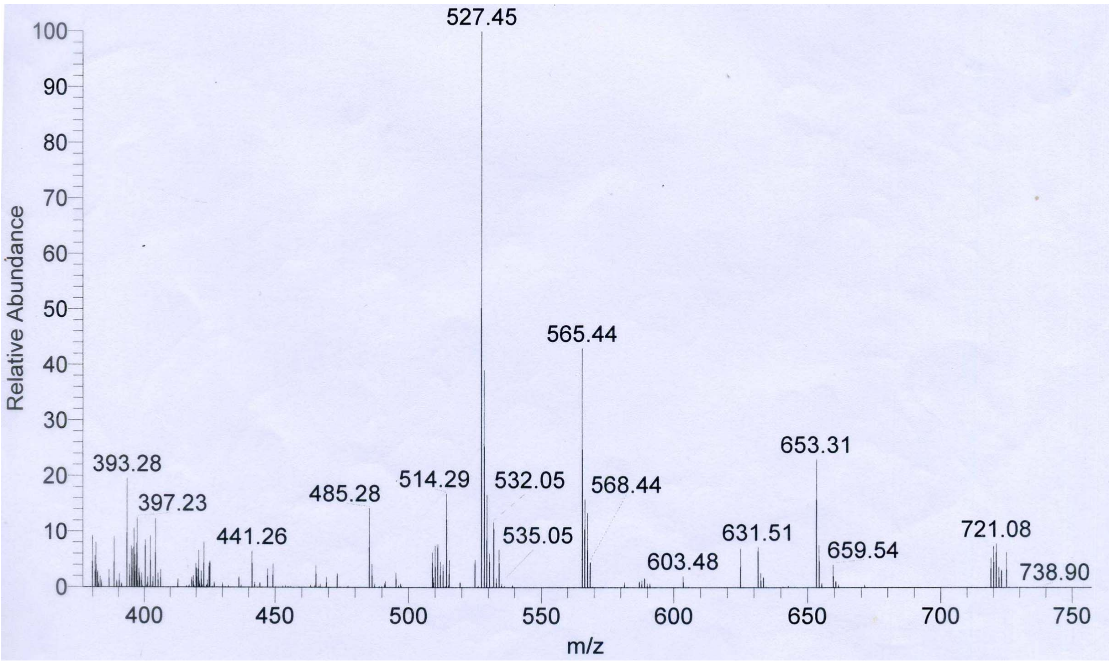


**Figure S25.** MS-EI spectra of **5**

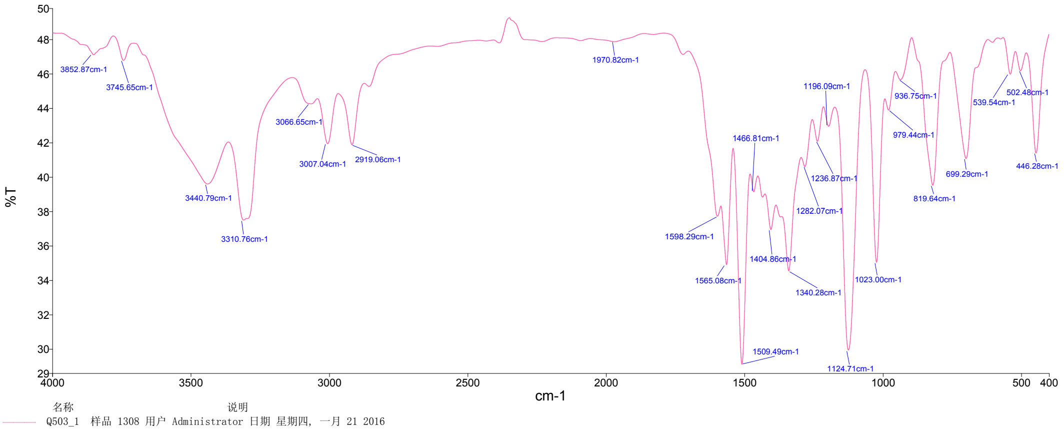


**Figure S26.** IR (KBr) spectra of **6**

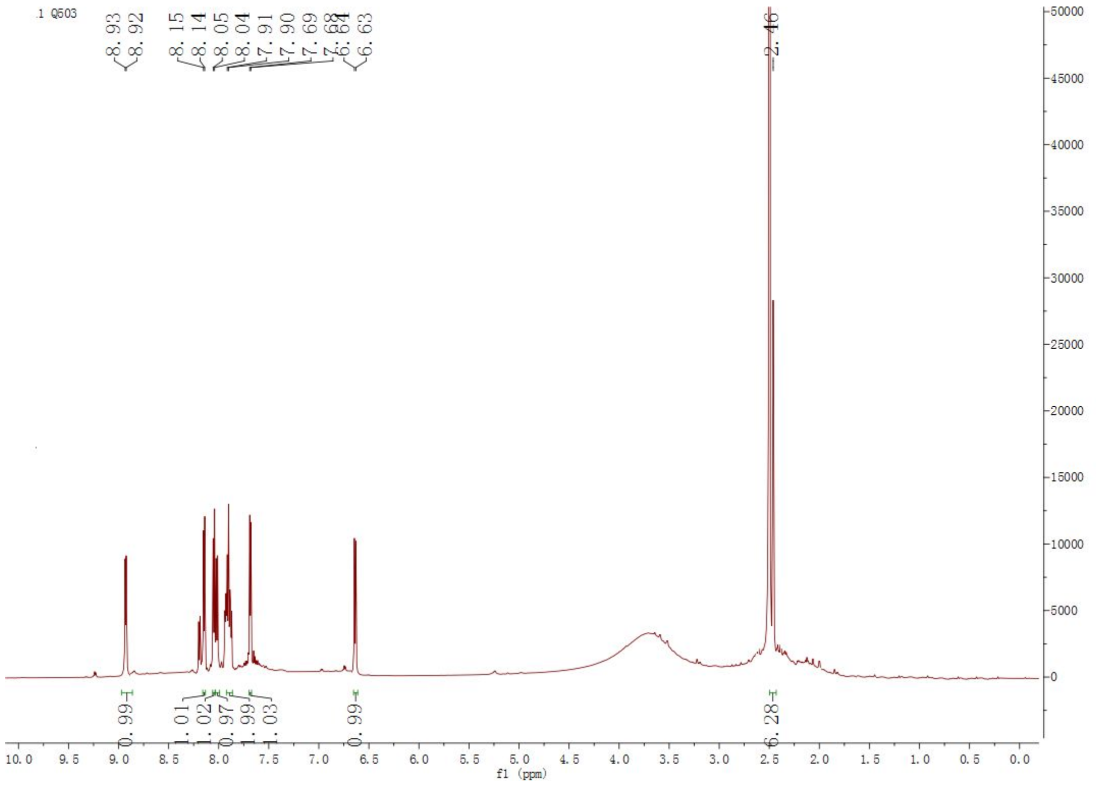


**Figure S27.** $^{1}$H NMR (600MHz, DMSO-d$_6$) for **6**

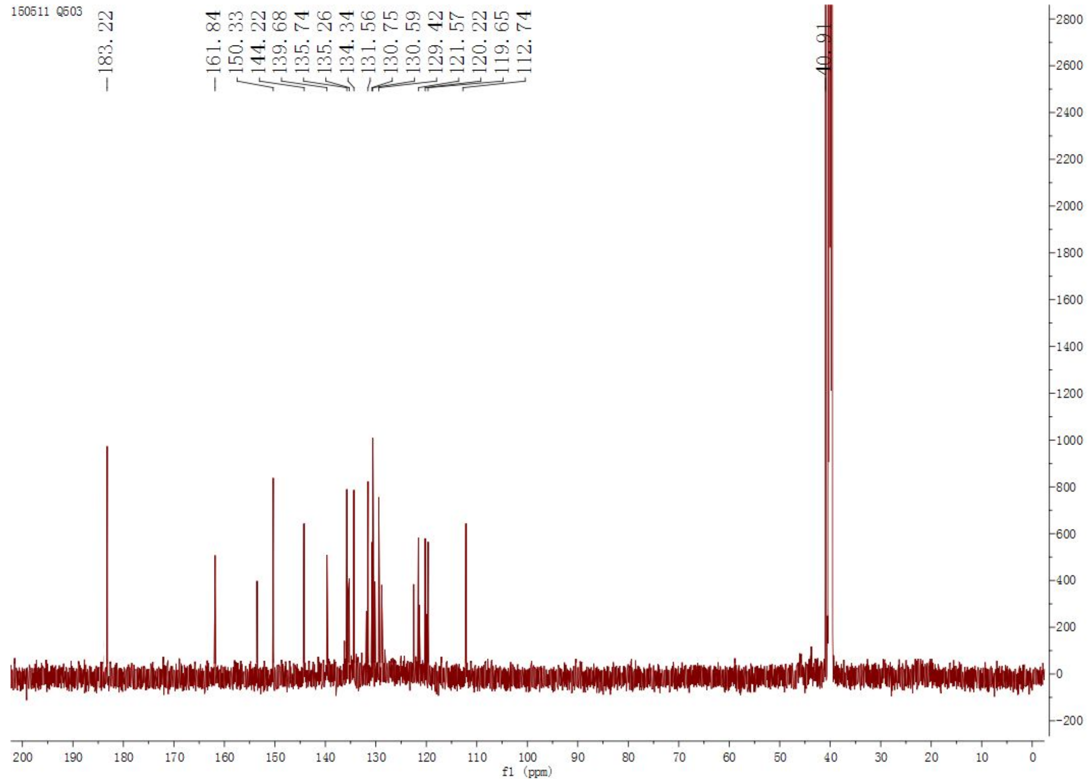


**Figure S28.** $^{13}$C NMR (600MHz, DMSO-d$_6$) for **6**

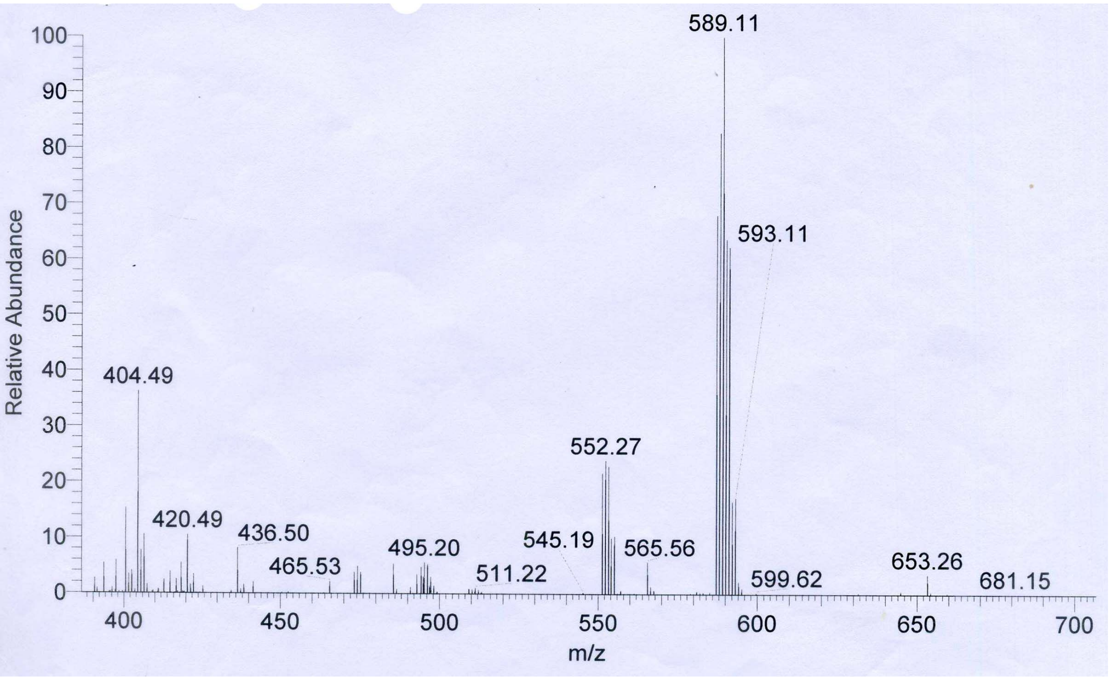


**Figure S29.** MS-EI spectra of **6**

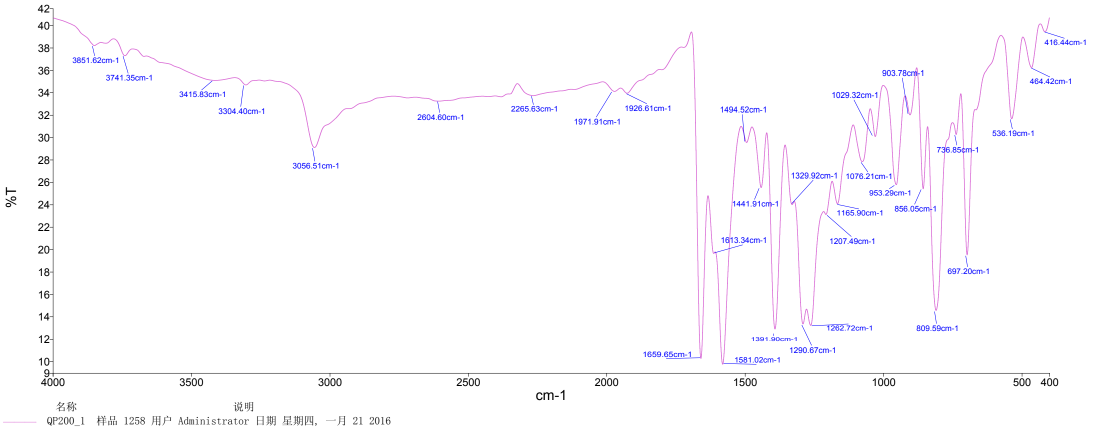


**Figure S30.** IR (KBr) spectra of $\mathbf{L^c}$

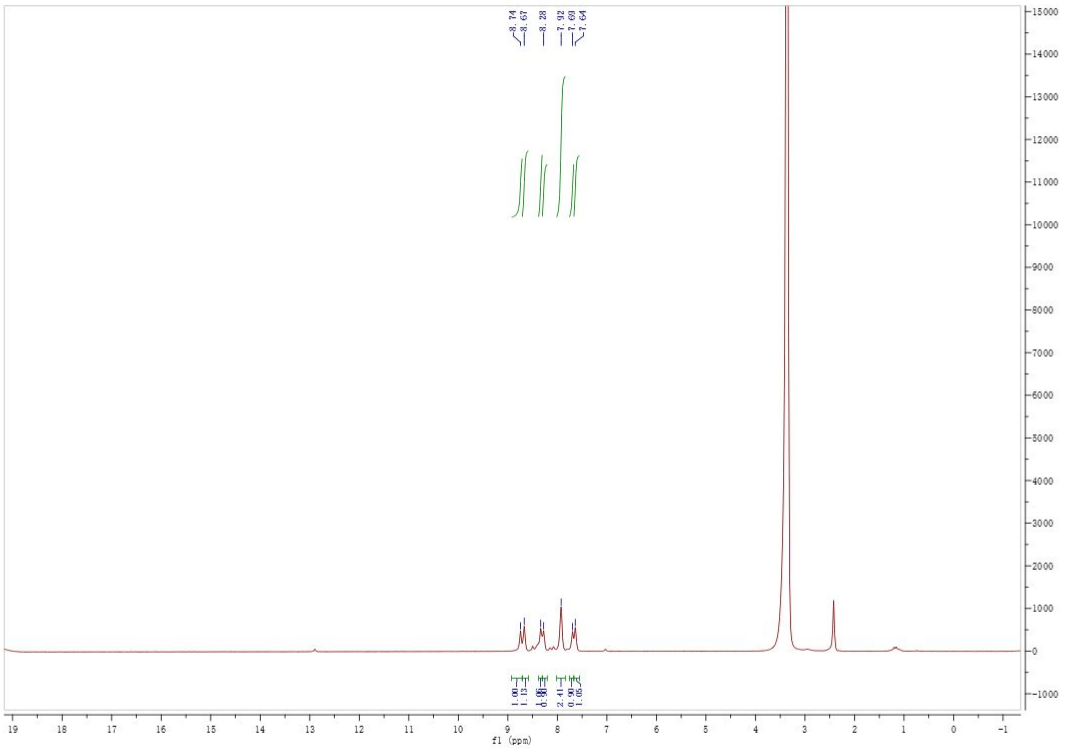


**Figure S31.** $^{1}$H NMR (600MHz, DMSO-d$_6$) for **L$^c$**

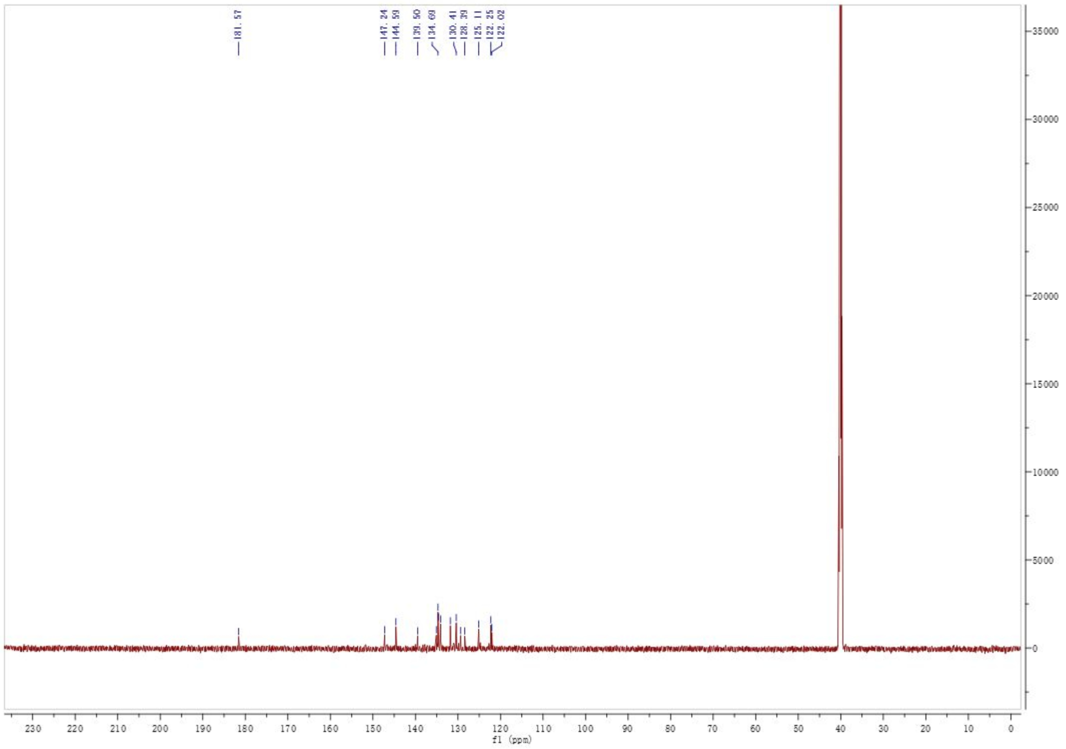


**Figure S32.** $^{13}$C NMR (600MHz, DMSO-d$_6$) for **L$^c$**

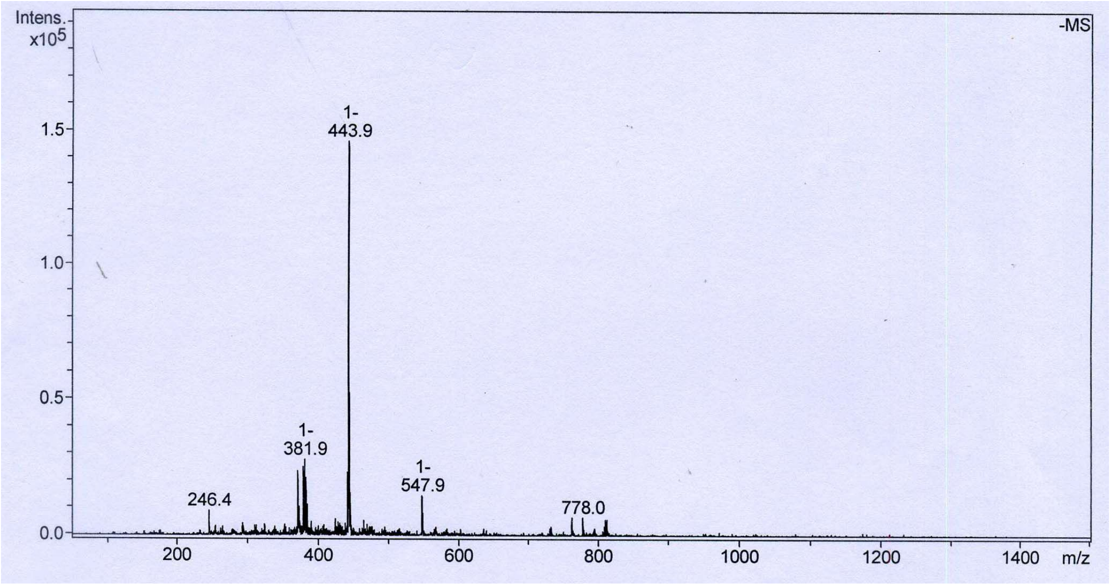


**Figure S33.** MS-EI spectra of **L**$^c$

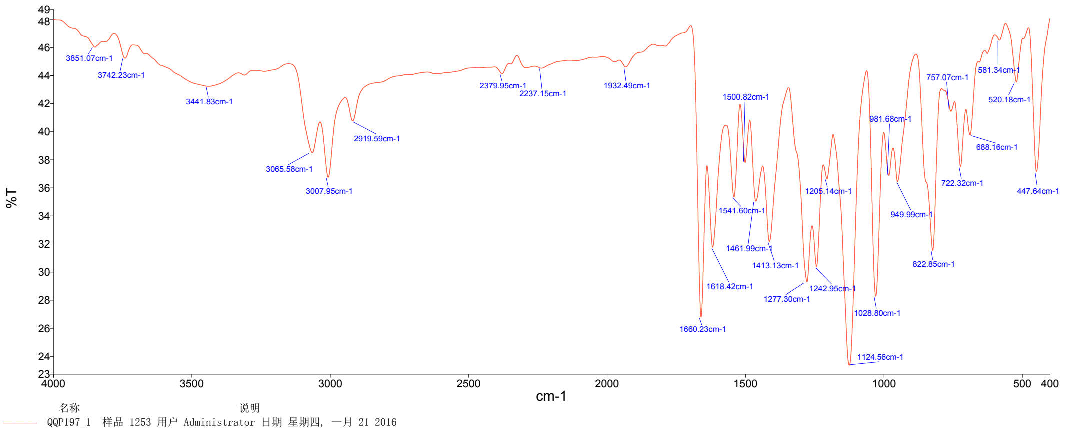


**Figure S34.** IR (KBr) spectra of **7**

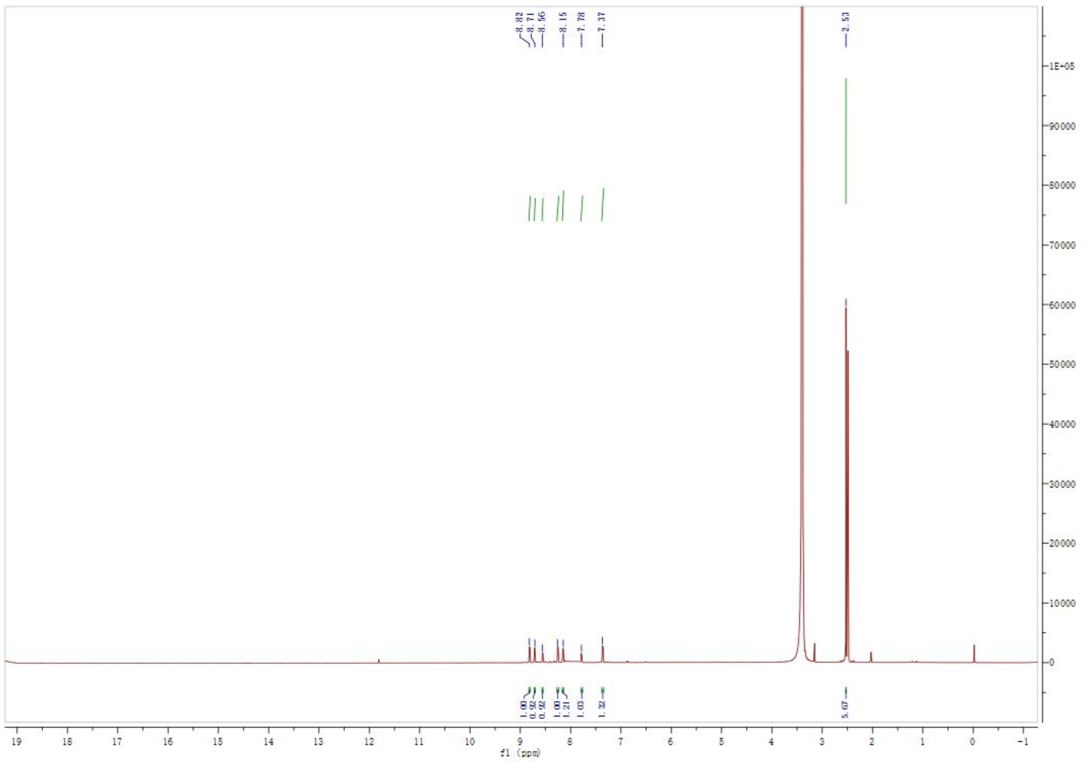


**Figure S35.** $^{1}H$ NMR (600MHz, DMSO-$d_6$) for **7**

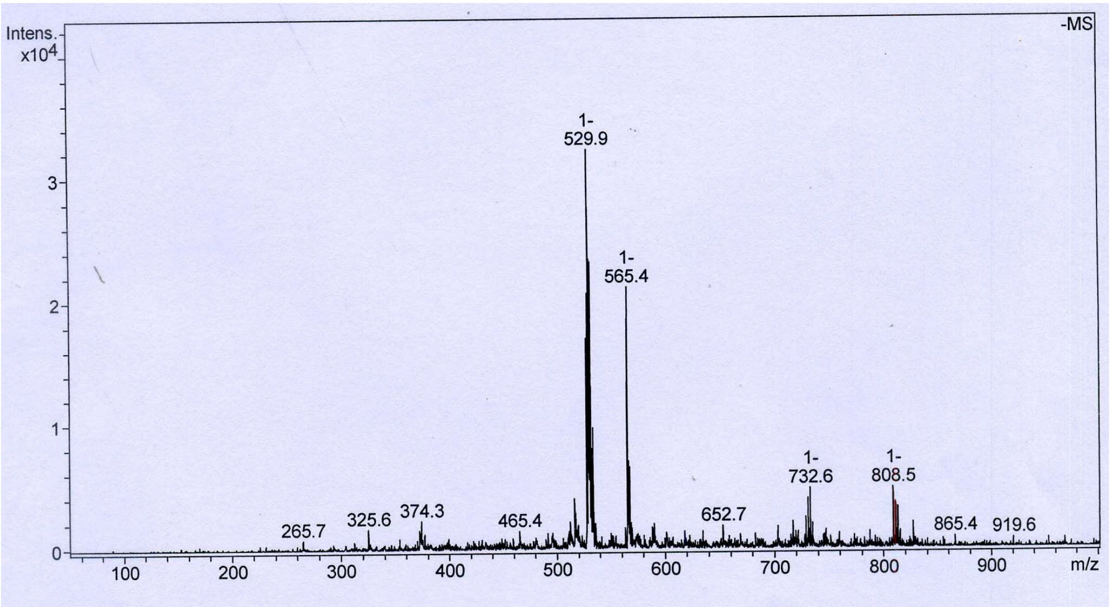


**Figure S36.** MS-EI spectra of **7**

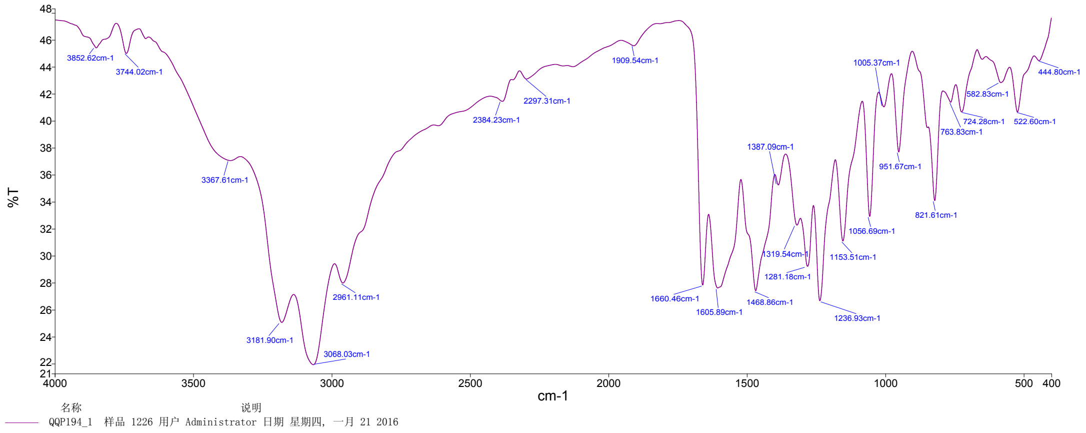


**Figure S37.** IR (KBr) spectra of **8**

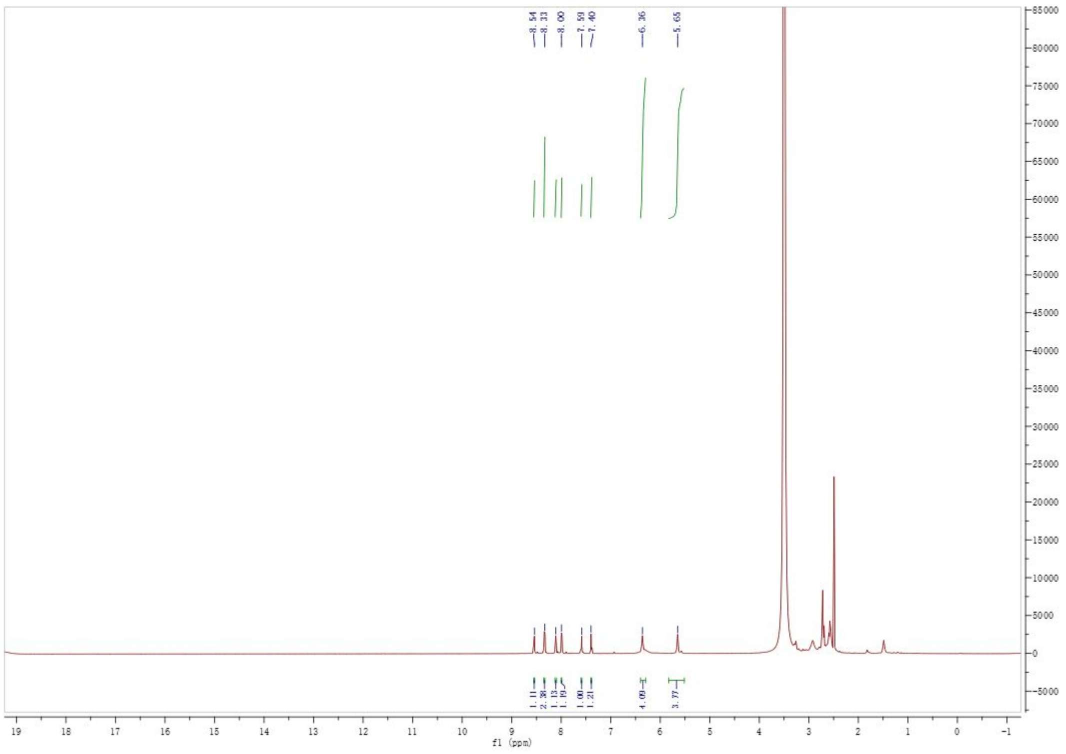


**Figure S38.** $^{1}$H NMR (600MHz, DMSO-d$_6$) for **8**

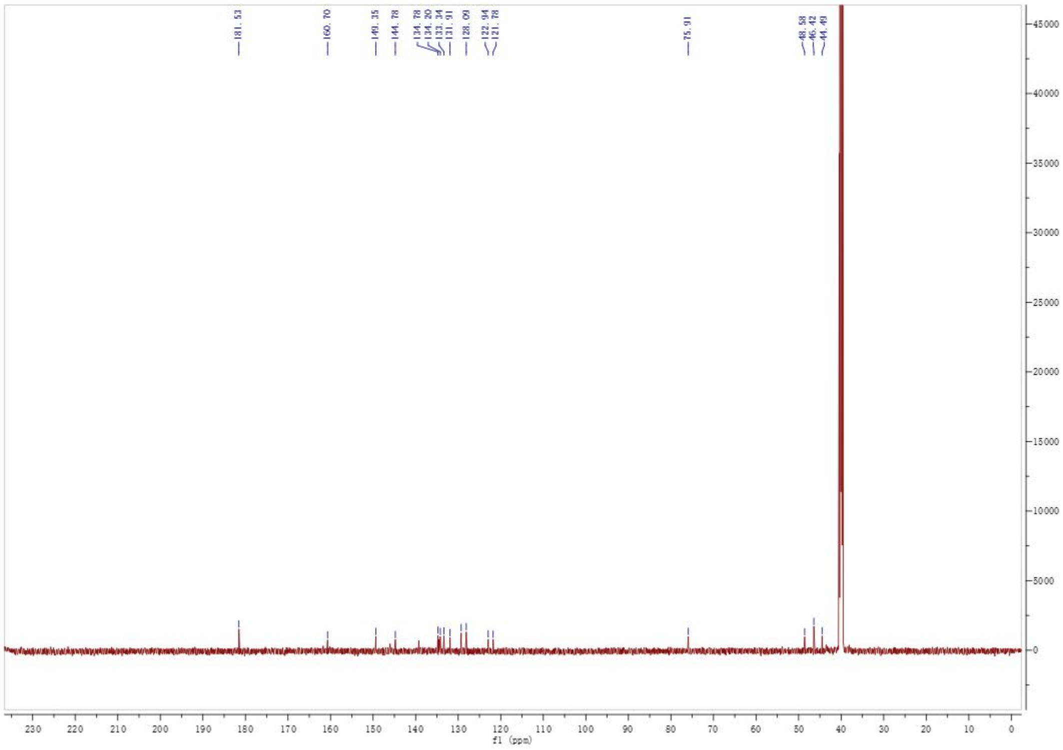


**Figure S39.** $^{13}$C NMR (600MHz, DMSO-$d_6$) for **8**

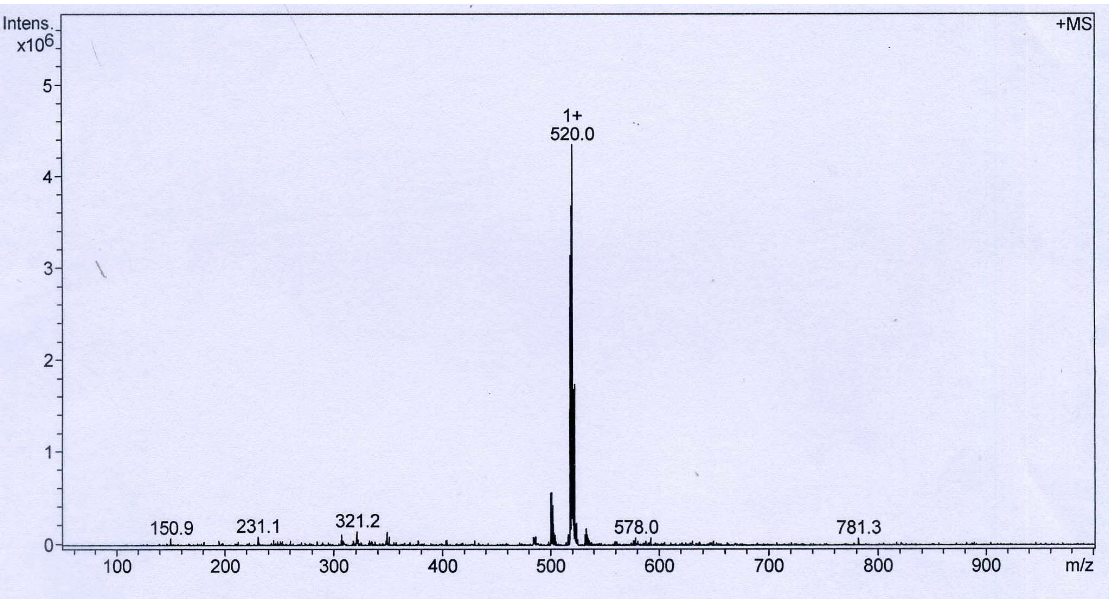


**Figure S40.** MS-EI spectra of **8**

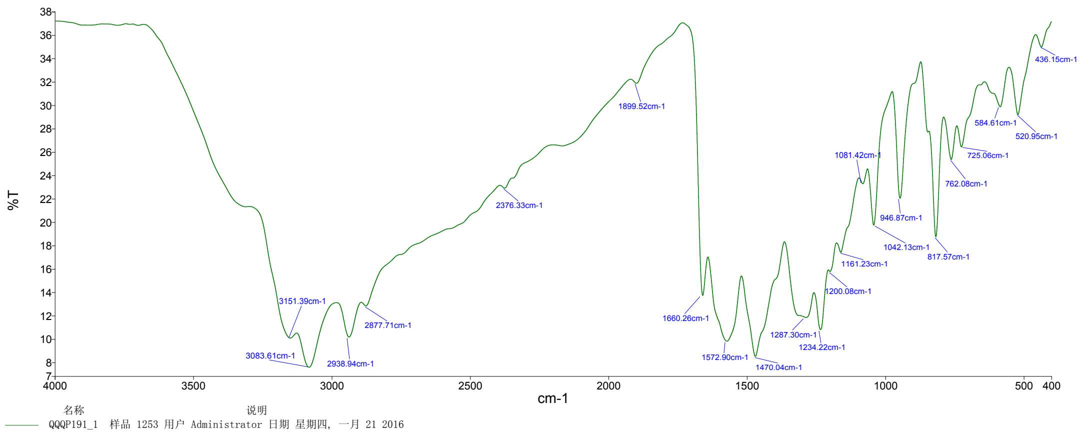


**Figure S41.** IR (KBr) spectra of **9**

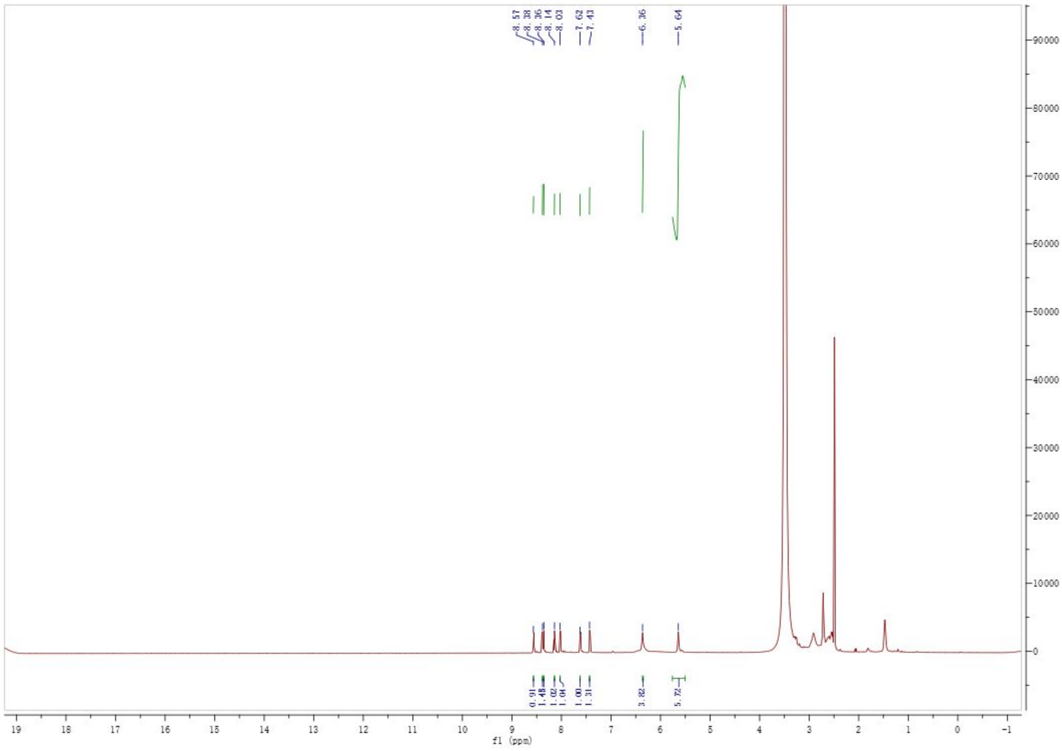


**Figure S42.** $^{1}$H NMR (600MHz, DMSO-$d_6$) for **9**

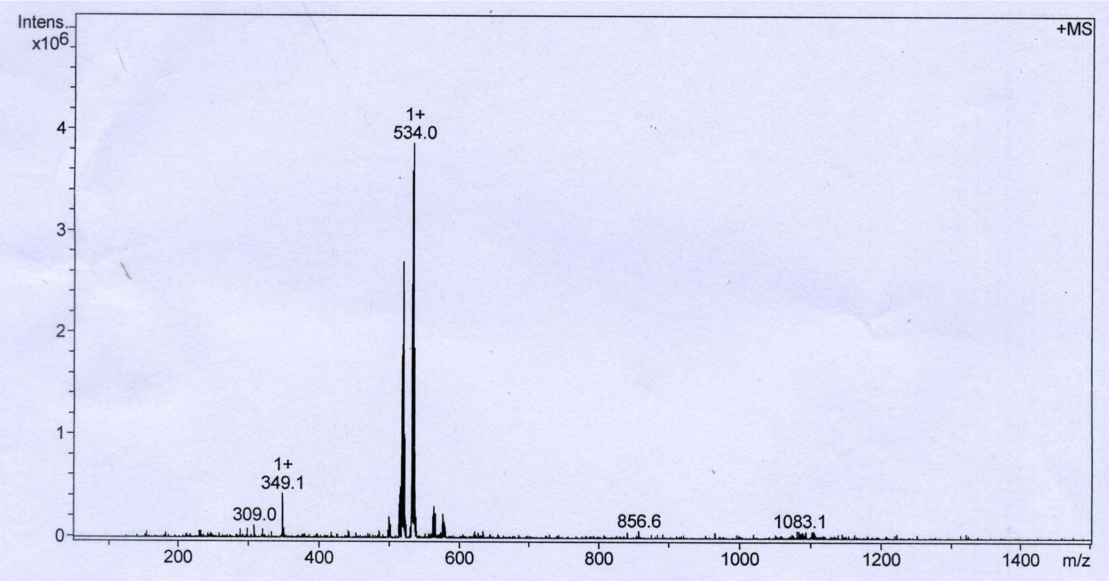


**Figure S43.** MS-EI spectra of **9**

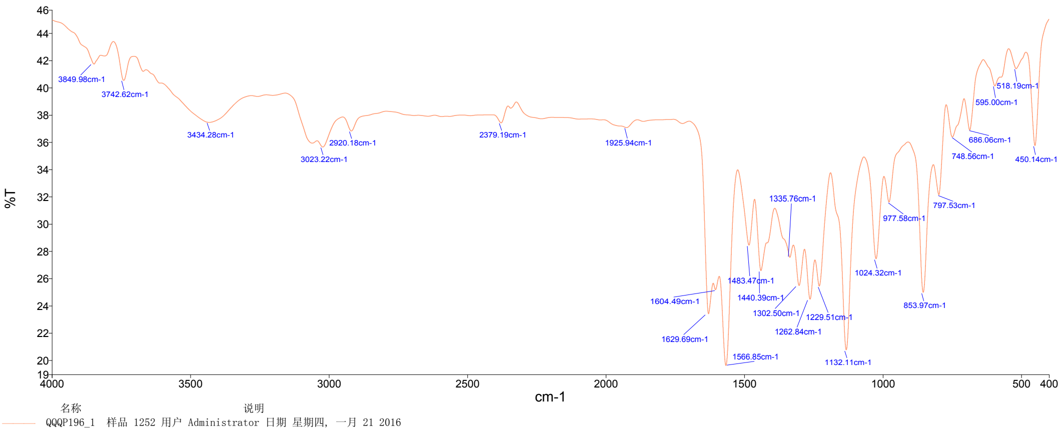


**Figure S44.** IR (KBr) spectra of **10**

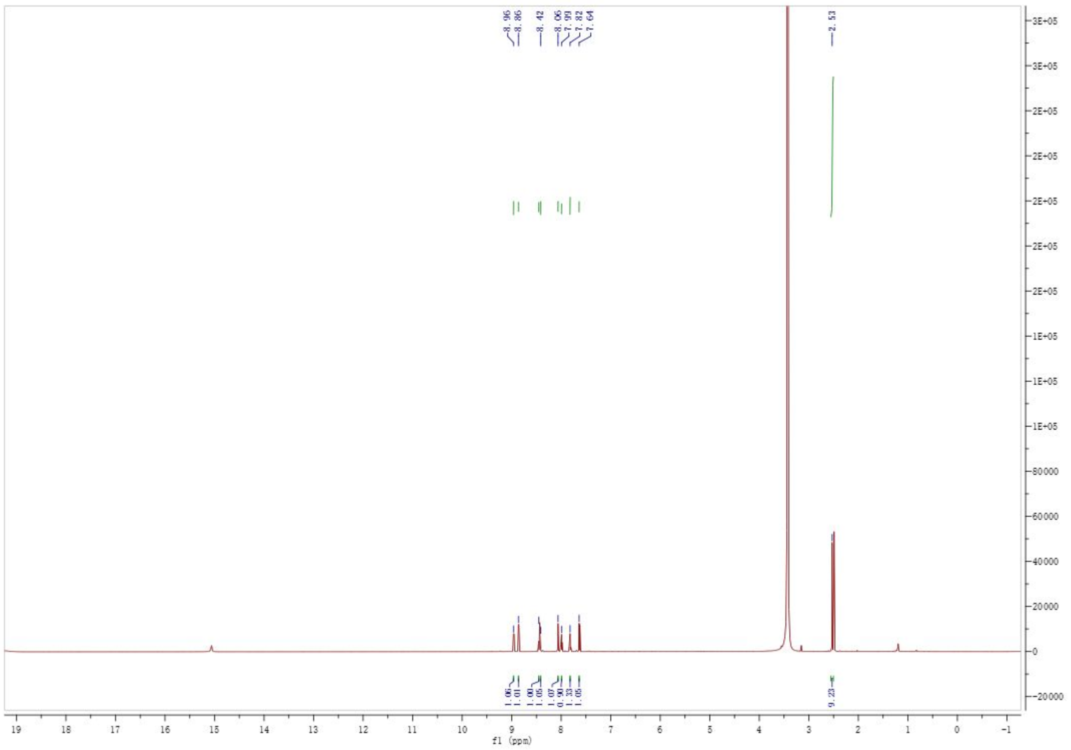


**Figure S45.** $^1$H NMR (600MHz, DMSO-$d_6$) for **10**

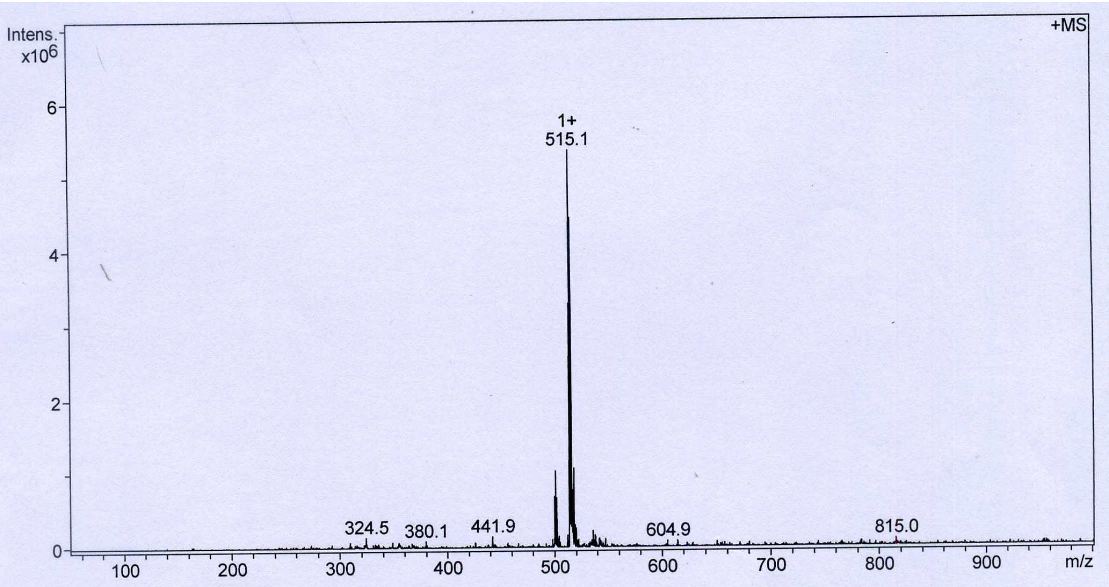

**Figure S46.** MS-EI spectra of **10**

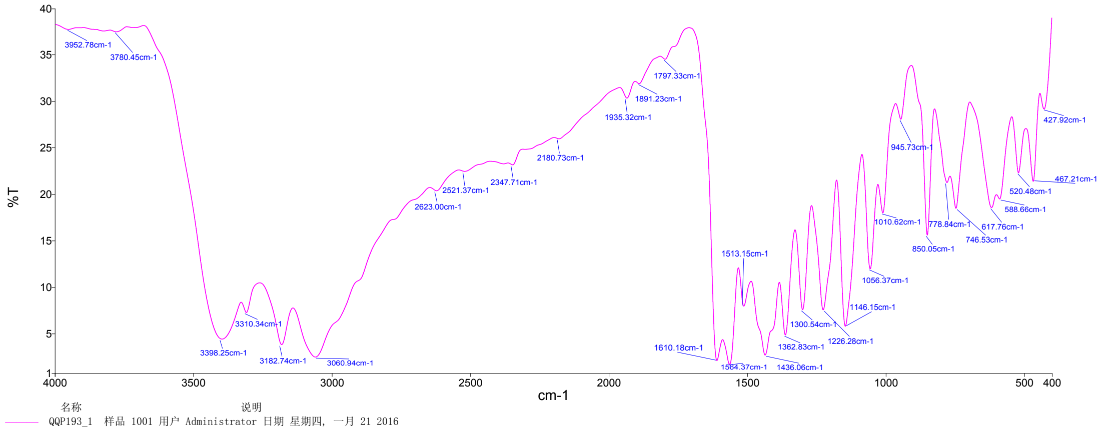

**Figure S47.** IR (KBr) spectra of **11**

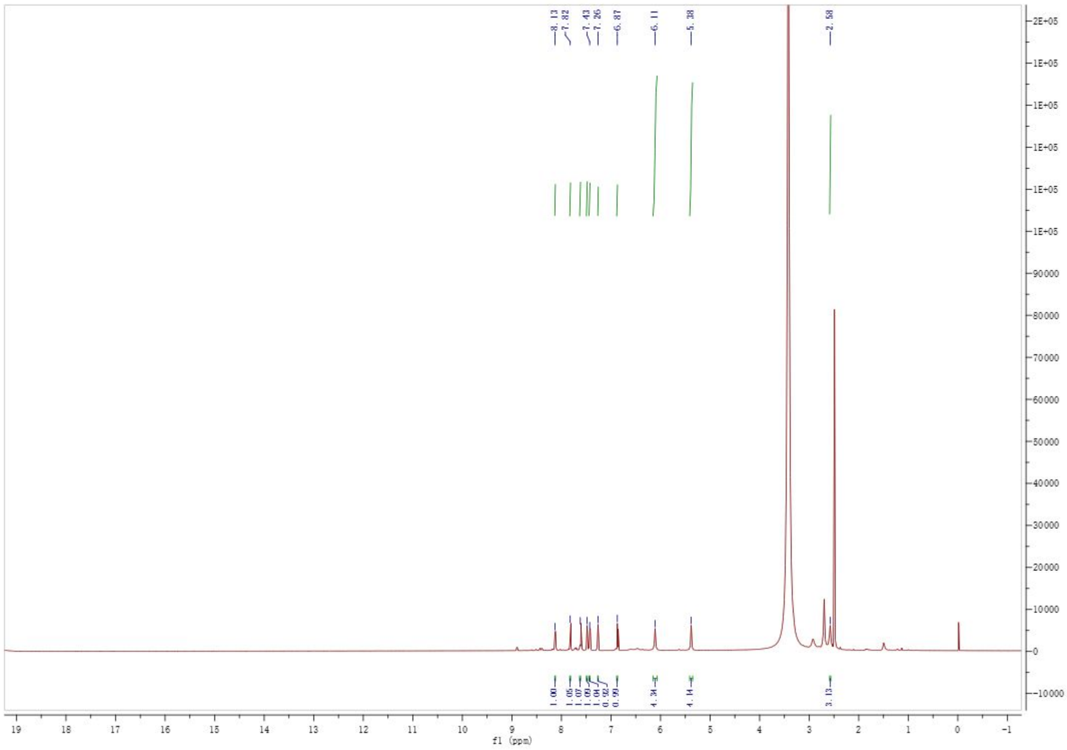

**Figure S48.** $^1$H NMR (600MHz, DMSO-d$_6$) for **11**

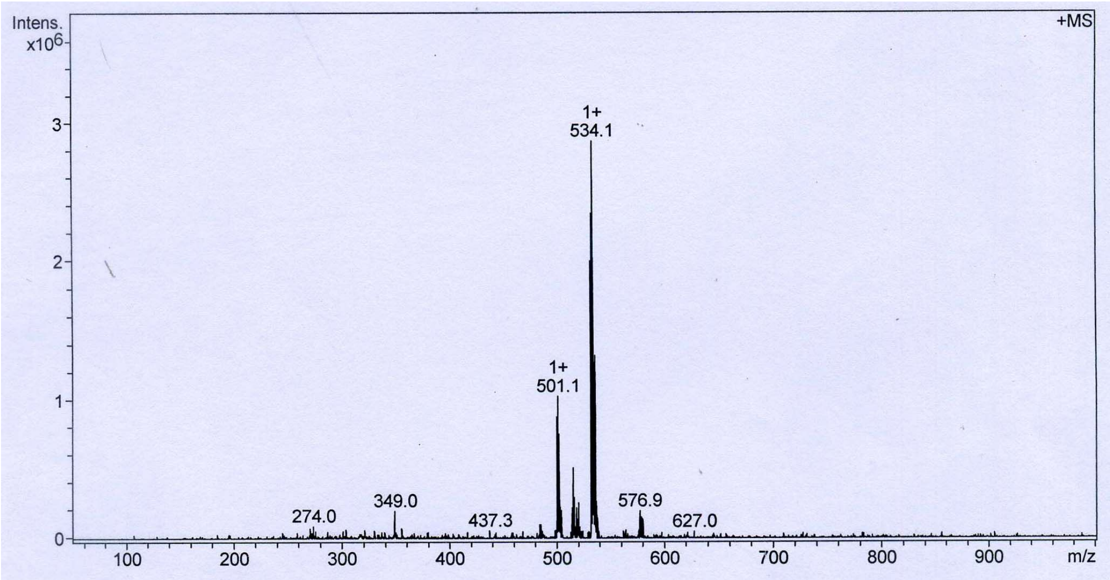


**Figure S49.** MS-EI spectra of **11**

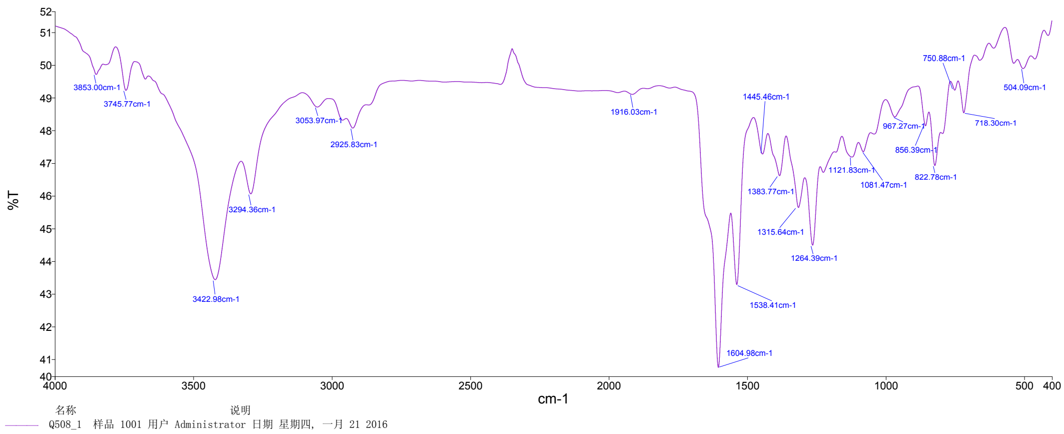


**Figure S50.** IR (KBr) spectra of $\mathbf{L^d}$

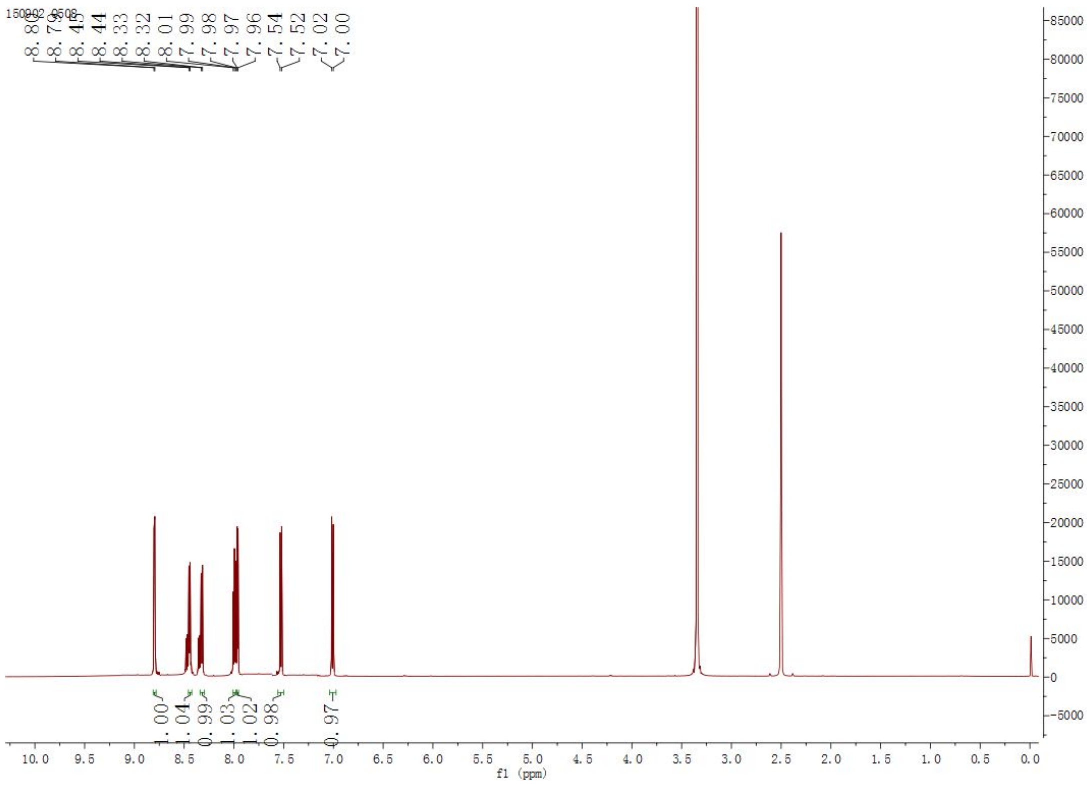

**Figure S51.** $^{1}H$ NMR (600MHz, DMSO-$d_6$) for $\mathbf{L^d}$

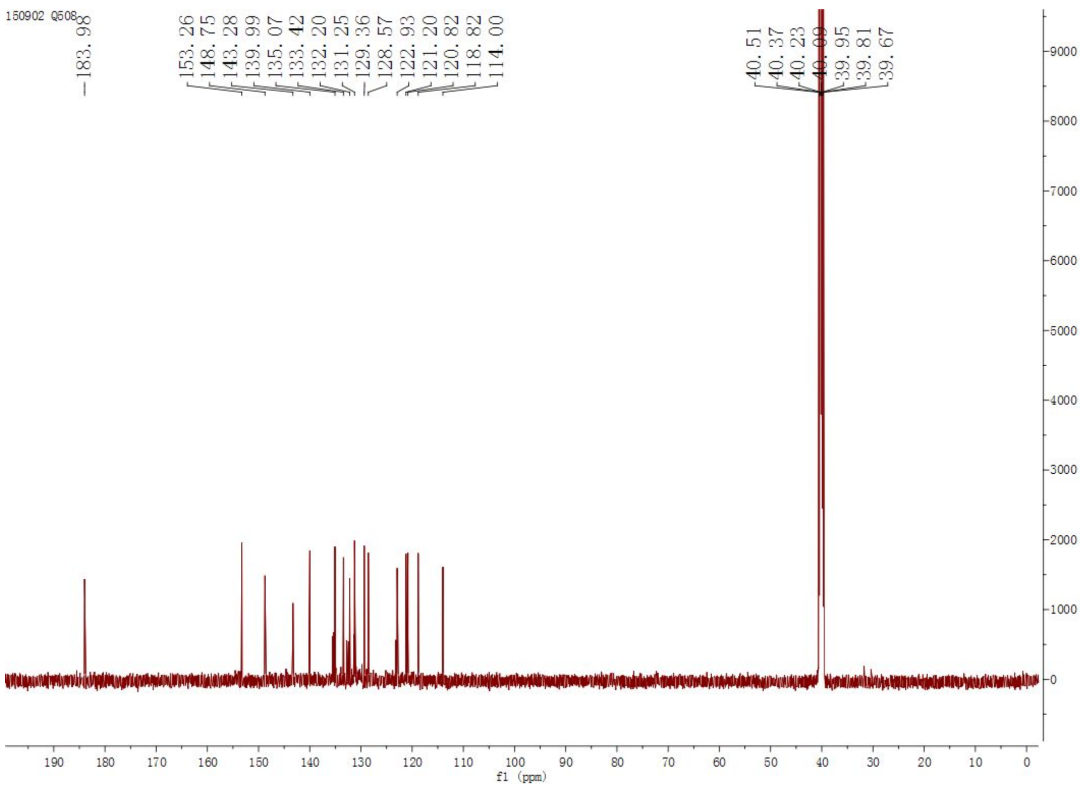

**Figure S52.** $^{13}C$ NMR (600MHz, DMSO-$d_6$) for $\mathbf{L^d}$

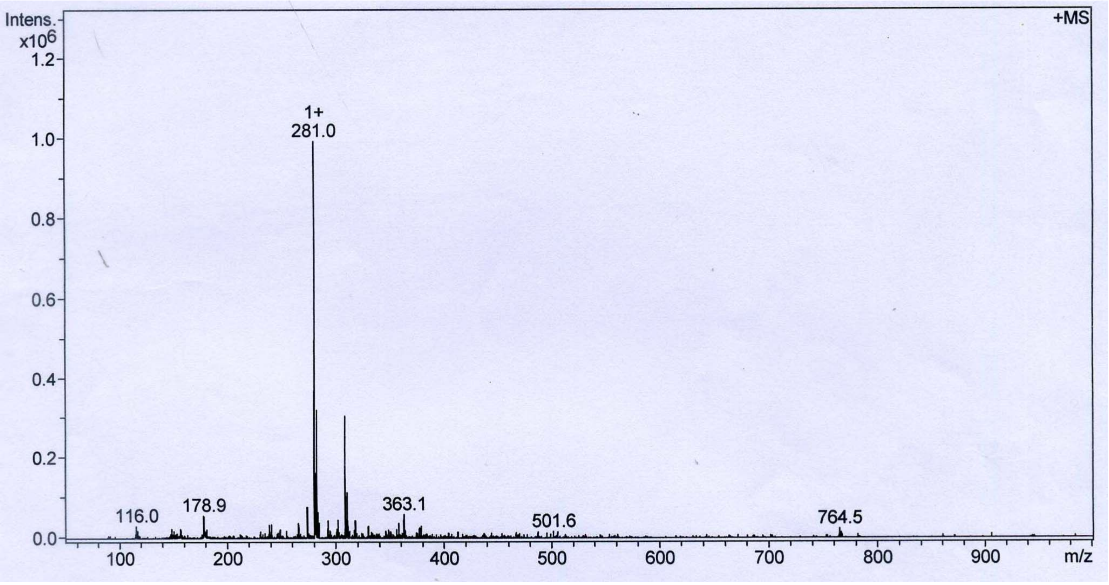


**Figure S53.** MS-EI spectra of $\mathbf{L^d}$

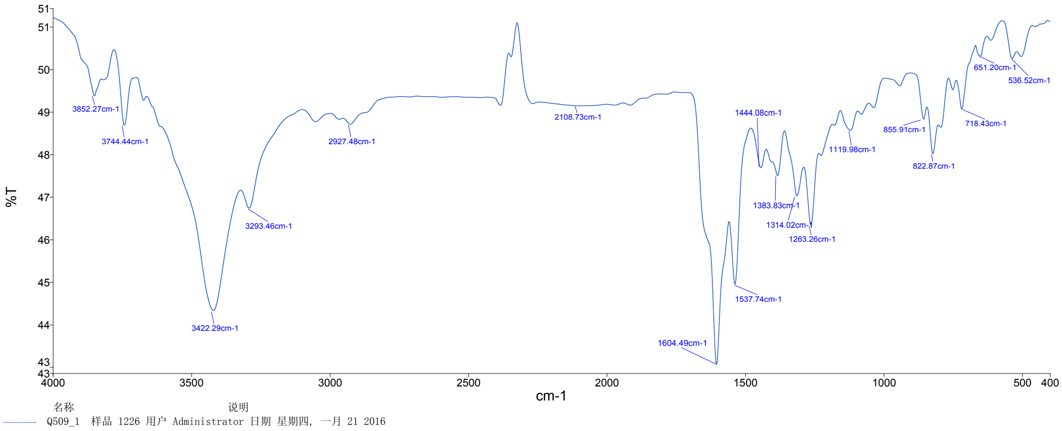


**Figure S54.** IR (KBr) spectra of **12**

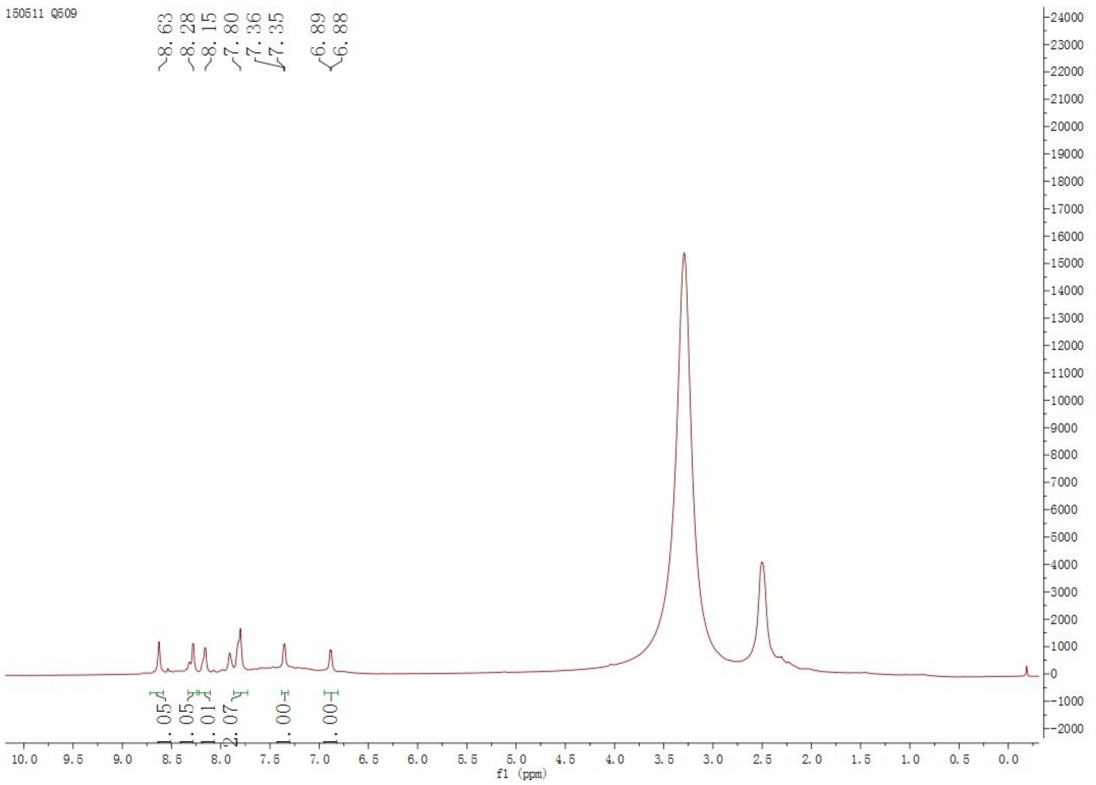


**Figure S55.** $^{1}$H NMR (600MHz, DMSO-$d_6$) for **12**

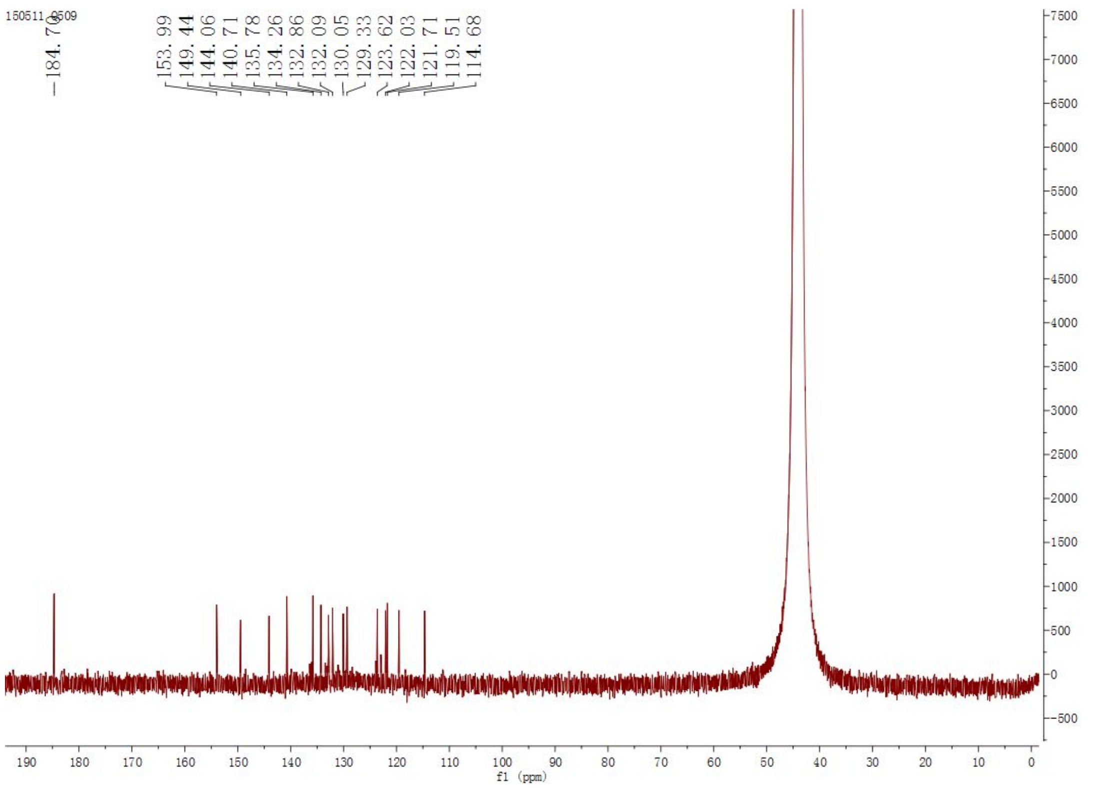


**Figure S56.** $^{13}$C NMR (600MHz, DMSO-$d_6$) for **12**

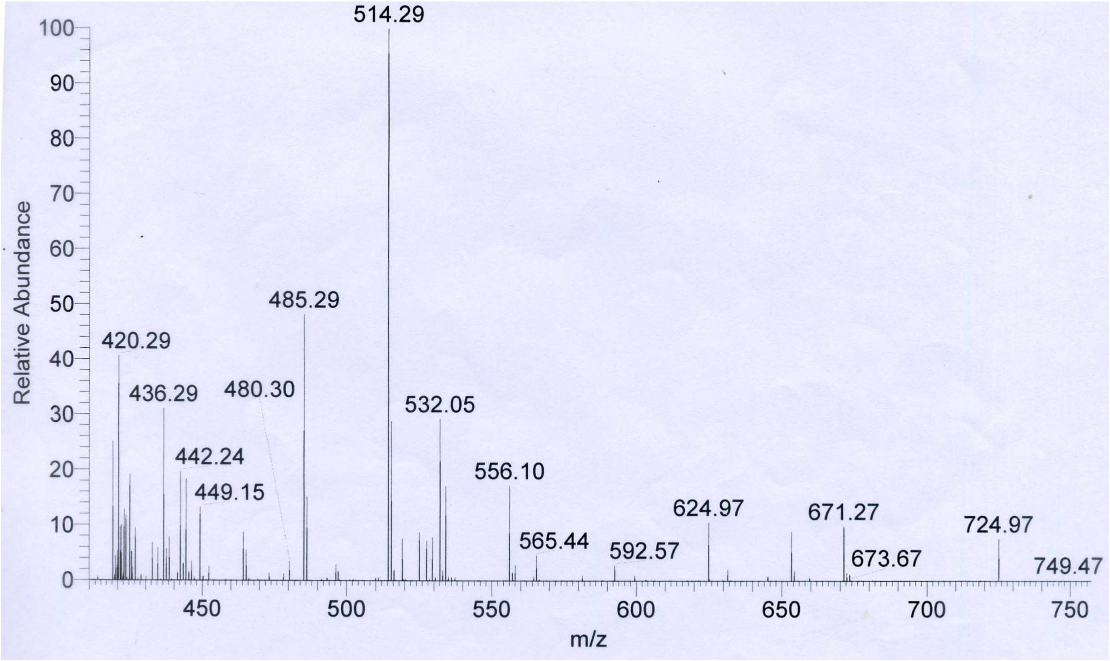


**Figure S57.** MS-EI spectra of **12**

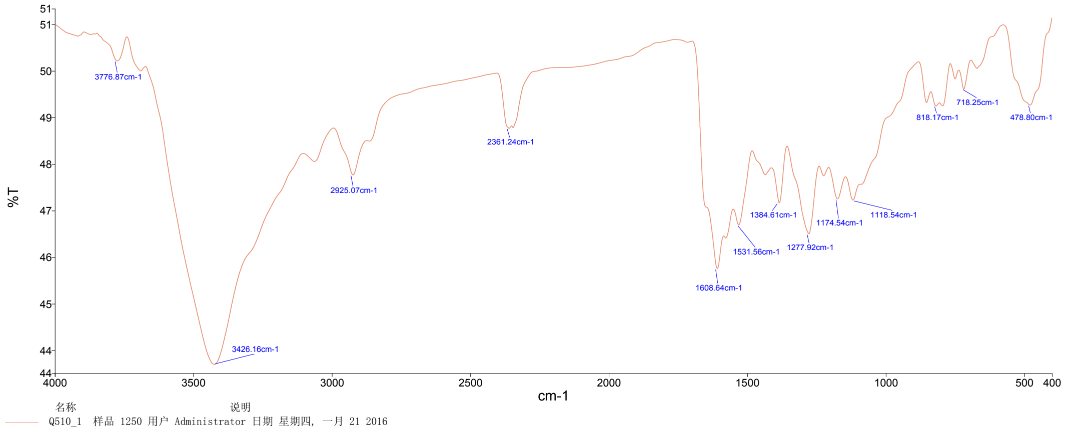


**Figure S58.** IR (KBr) spectra of **13**

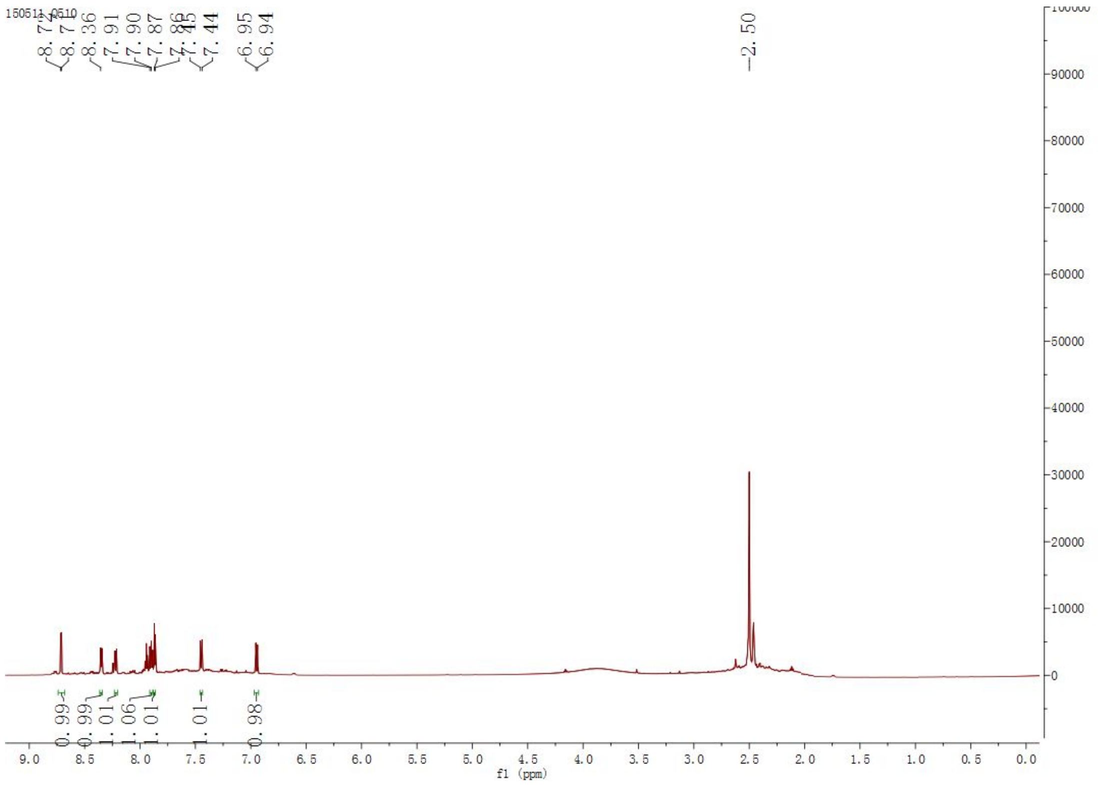


**Figure S59.** $^{1}$H NMR (600MHz, DMSO-$d_6$) for **13**

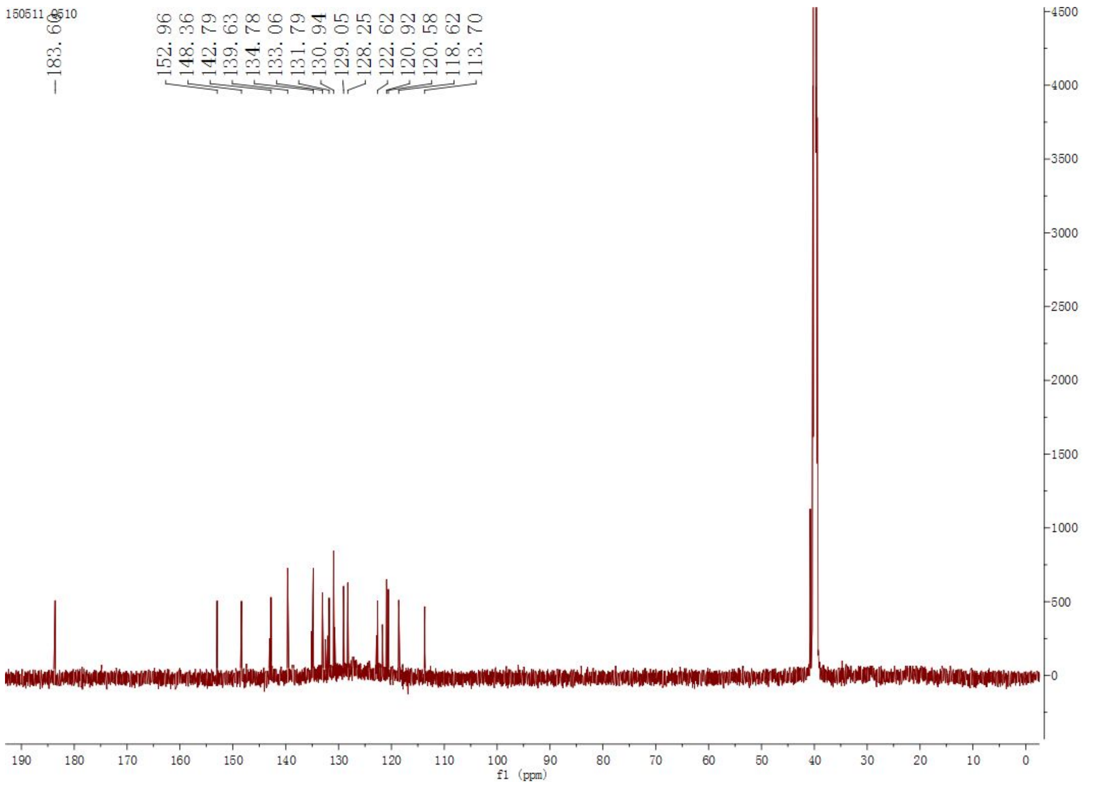


**Figure S60.** $^{13}$C NMR (600MHz, DMSO-$d_6$) for **13**

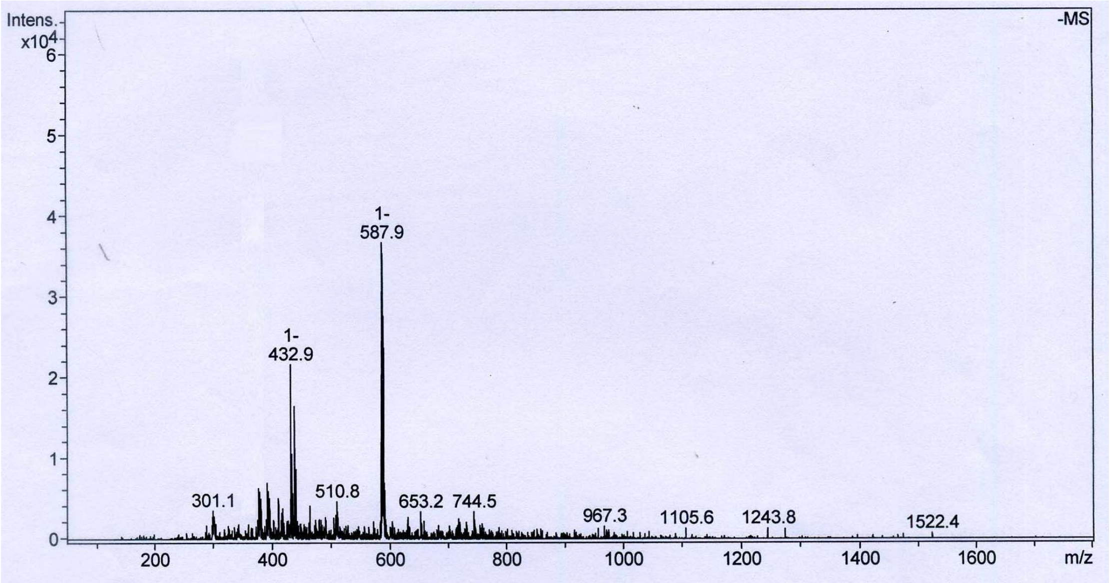

**Figure S61.** MS-EI spectra of **13**

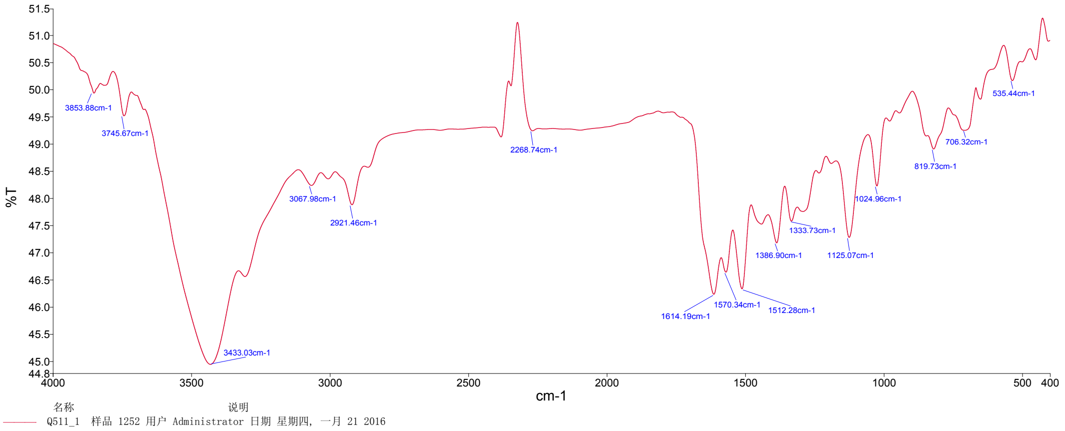

**Figure S62.** IR (KBr) spectra of **14**

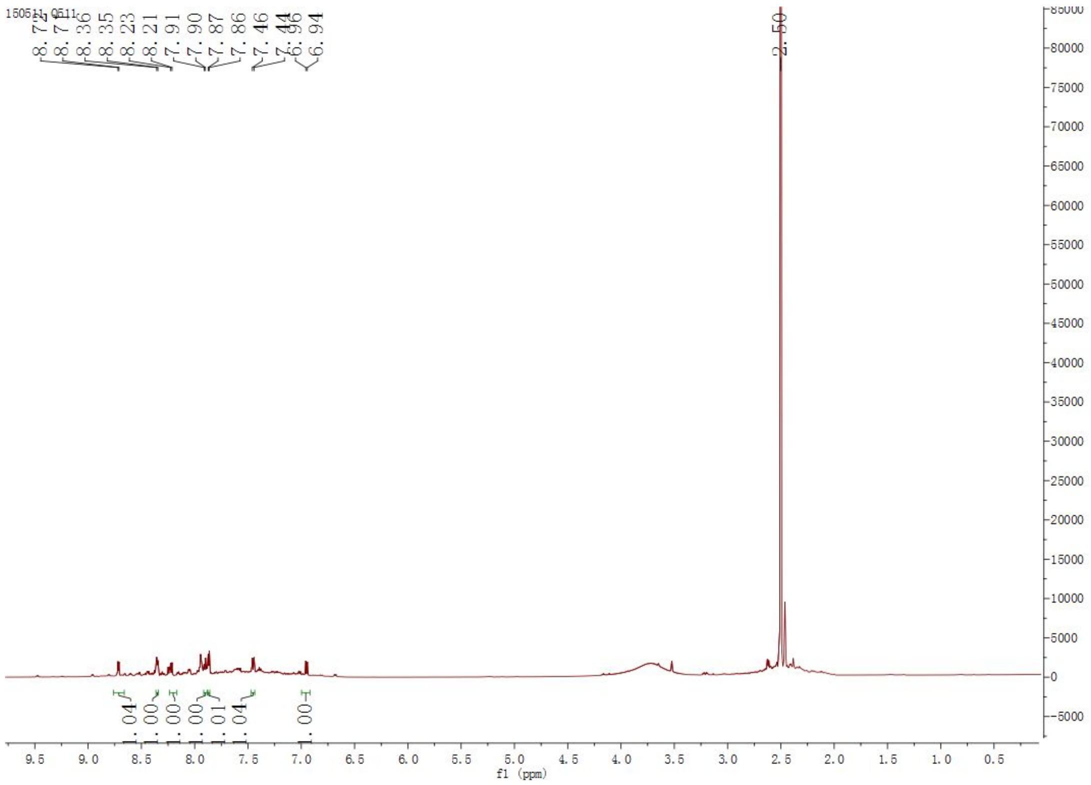

**Figure S63.** $^{1}H$ NMR (600MHz, DMSO-$d_6$) for **14**

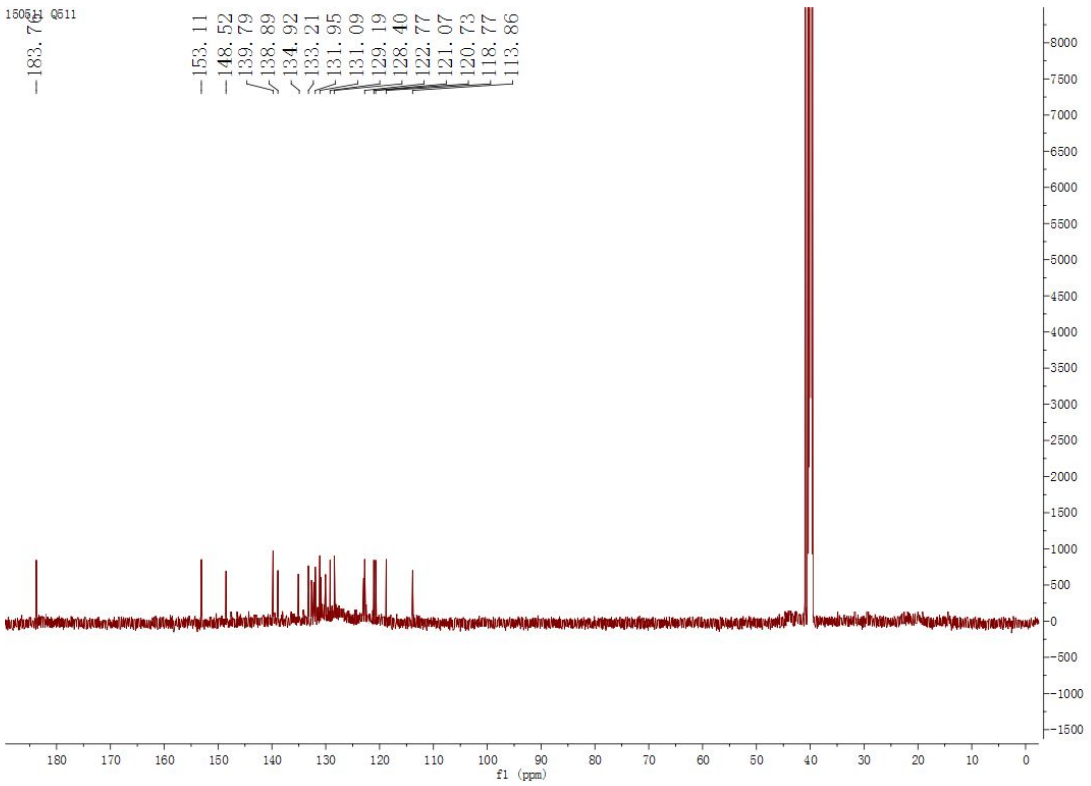

**Figure S64.** $^{13}C$ NMR (600MHz, DMSO-$d_6$) for **14**

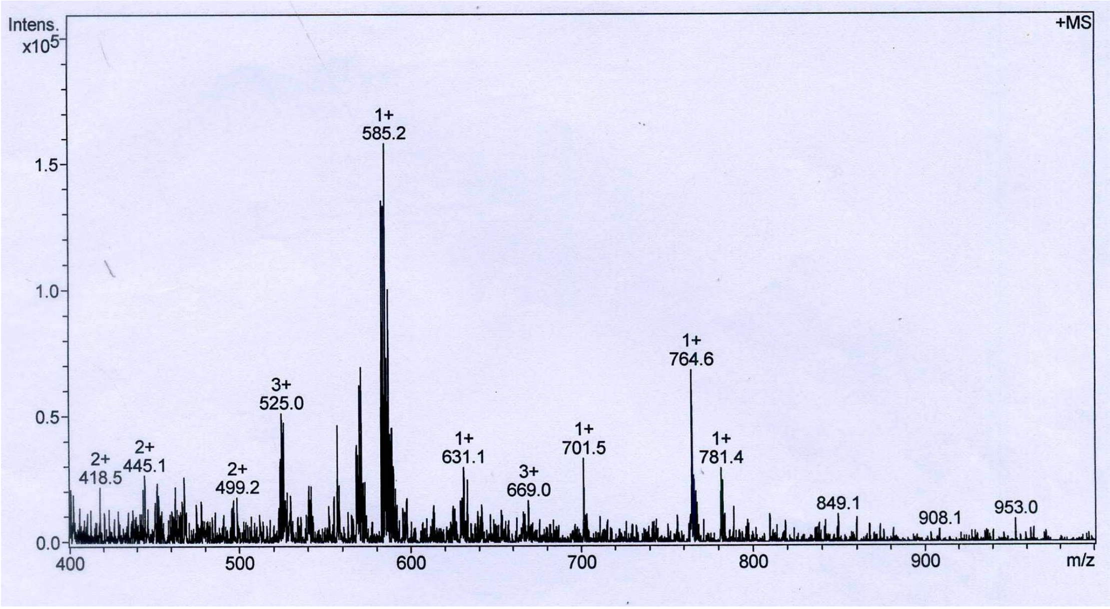


**Figure S65.** MS-EI spectra of **14**

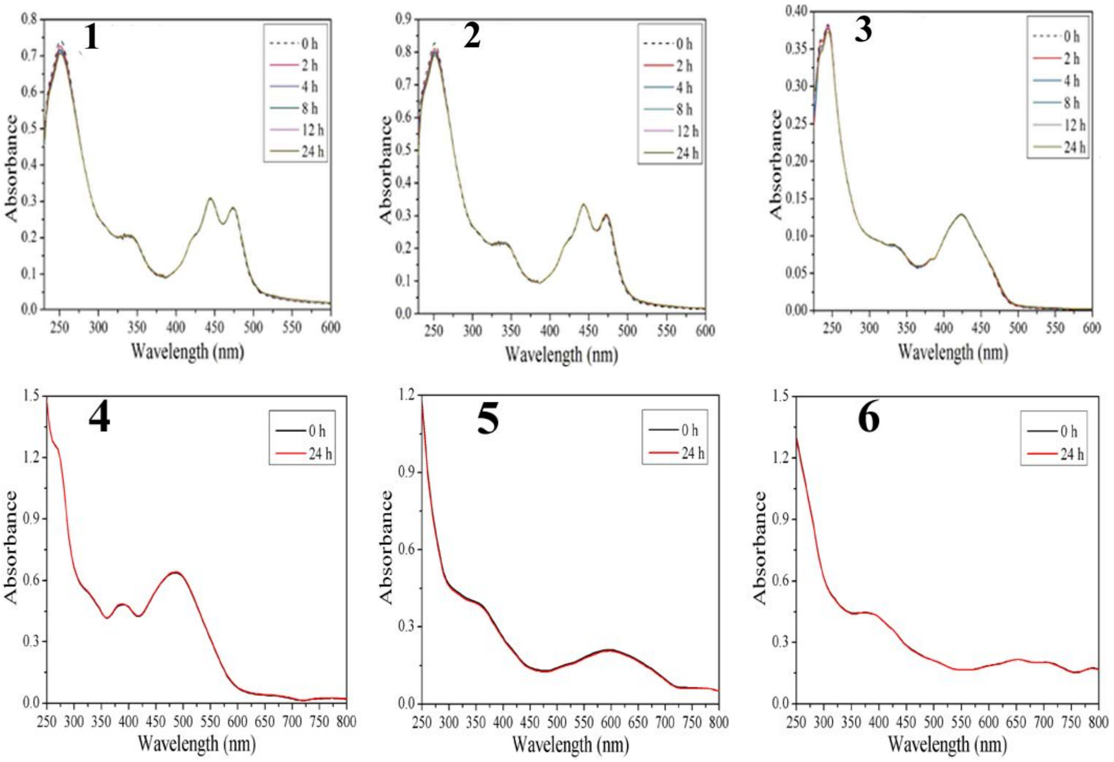


**Figure S66.** UV-Vis absorption spectra of **1**–**6** in Tris-KCl-HCl solution ($3.0\times10^{-5}$ M) in the time course 0 and 24 h, respectively.

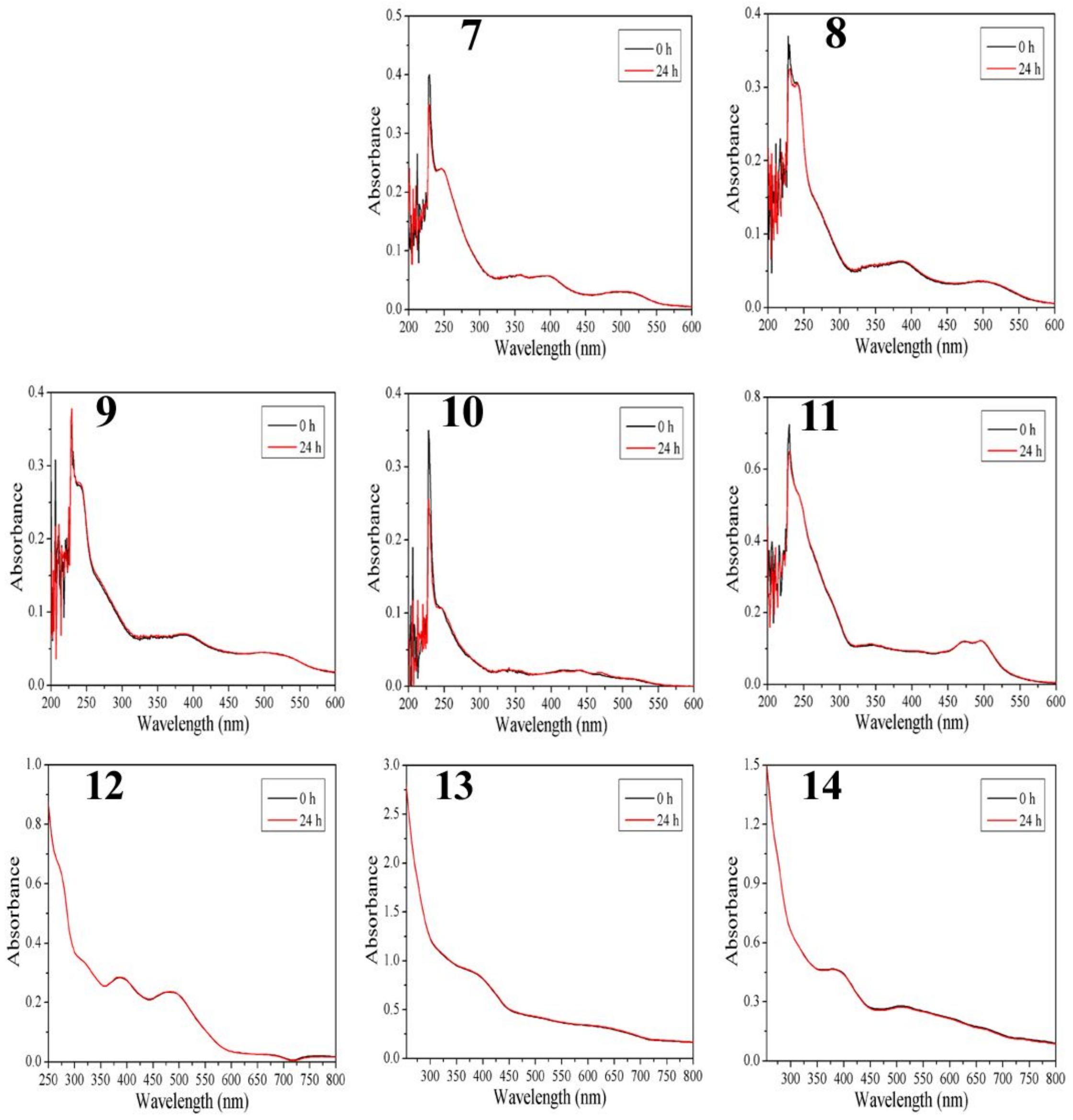


**Figure S67.** UV-Vis absorption spectra of **6–14** in Tris-KCl-HCl solution ($3.0\times10^{-5}$ M) in the time course 0 and 24 h, respectively.

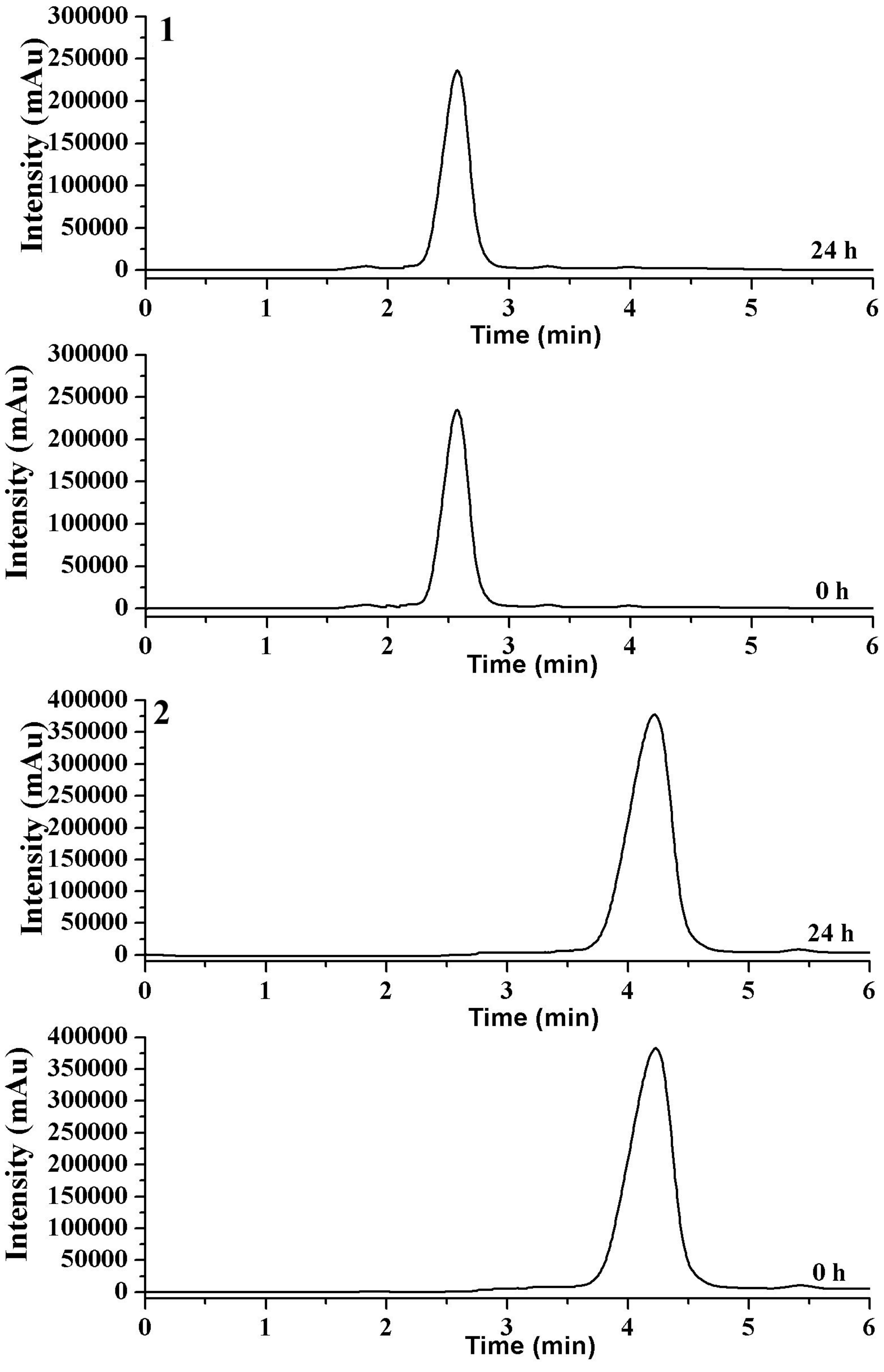
1
Intensity (mAu)
300000
250000
200000
150000
100000
50000
0
24 h
0 h
Time (min)
0
1
2
3
4
5
6
2
Intensity (mAu)
400000
350000
300000
250000
200000
150000
100000
50000
0
24 h
0 h
Time (min)

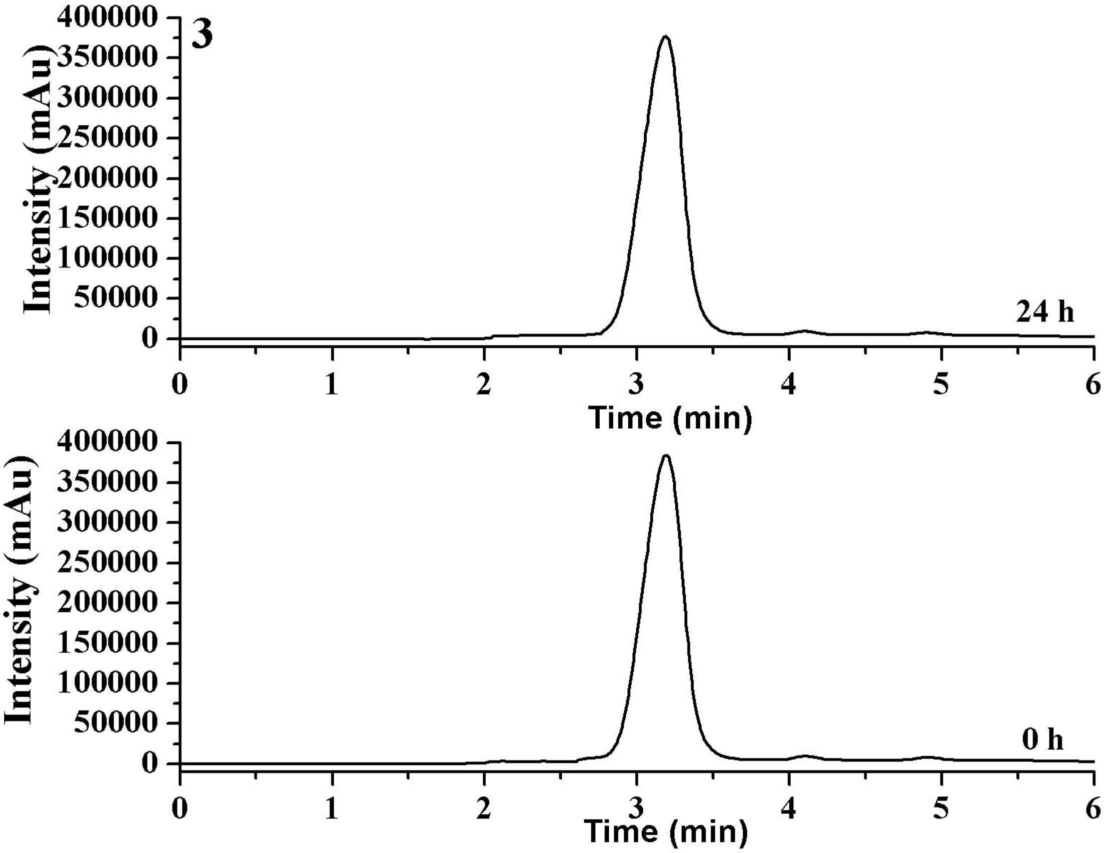
3
Intensity (mAu)
400000
350000
300000
250000
200000
150000
100000
50000
0
24 h
0 h
0
1
2
3
4
5
6
Time (min)

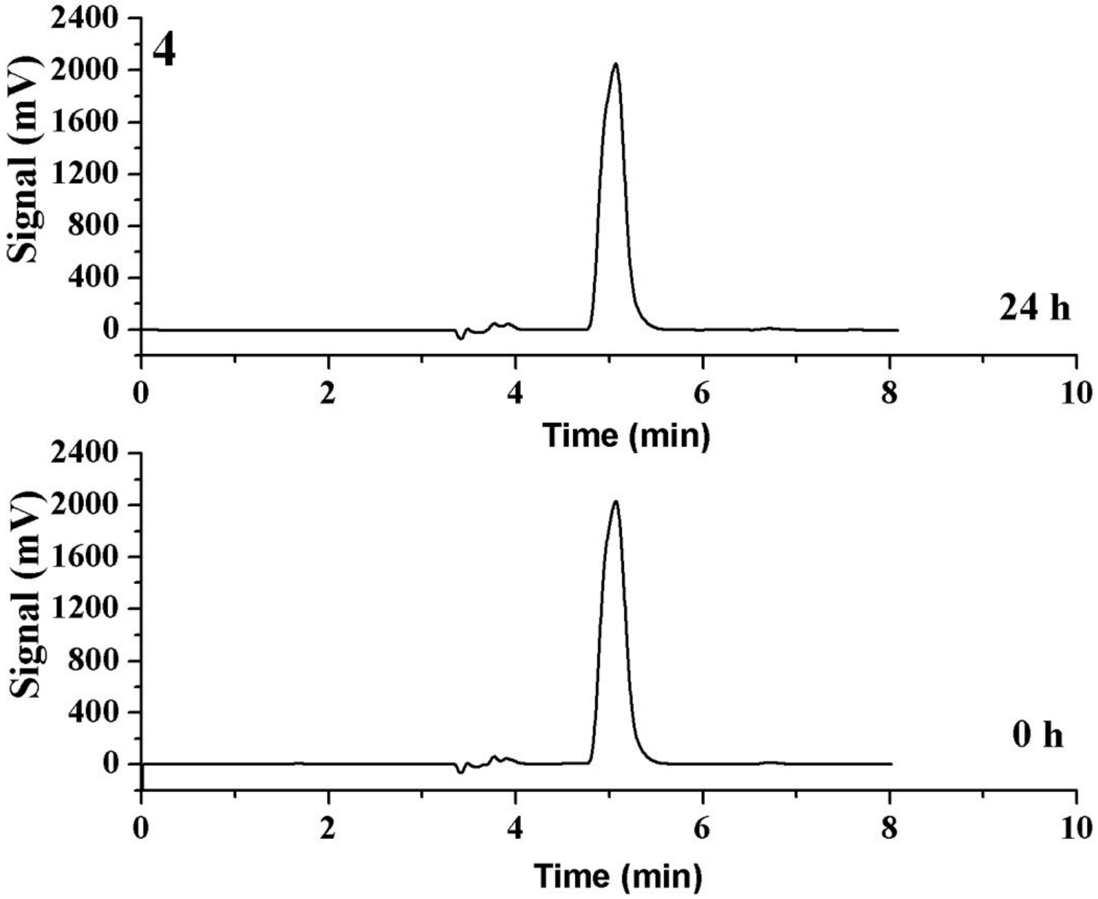
4
Signal (mV)
2400
2000
1600
1200
800
400
0
24 h
0 h
0
2
4
6
8
10
Time (min)

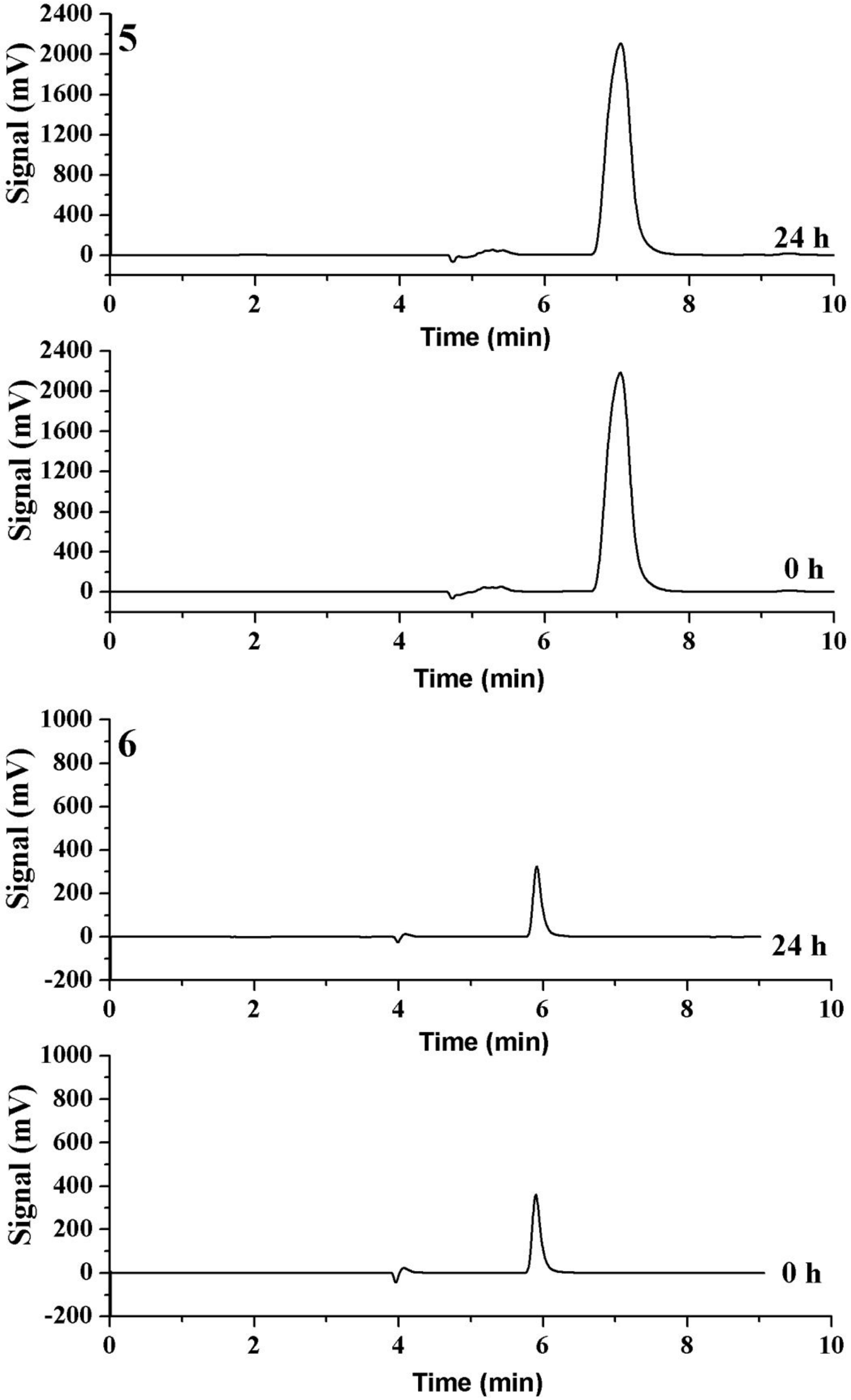
5
Signal (mV)
Time (min)
24 h
0 h
6
Signal (mV)
Time (min)
24 h
0 h

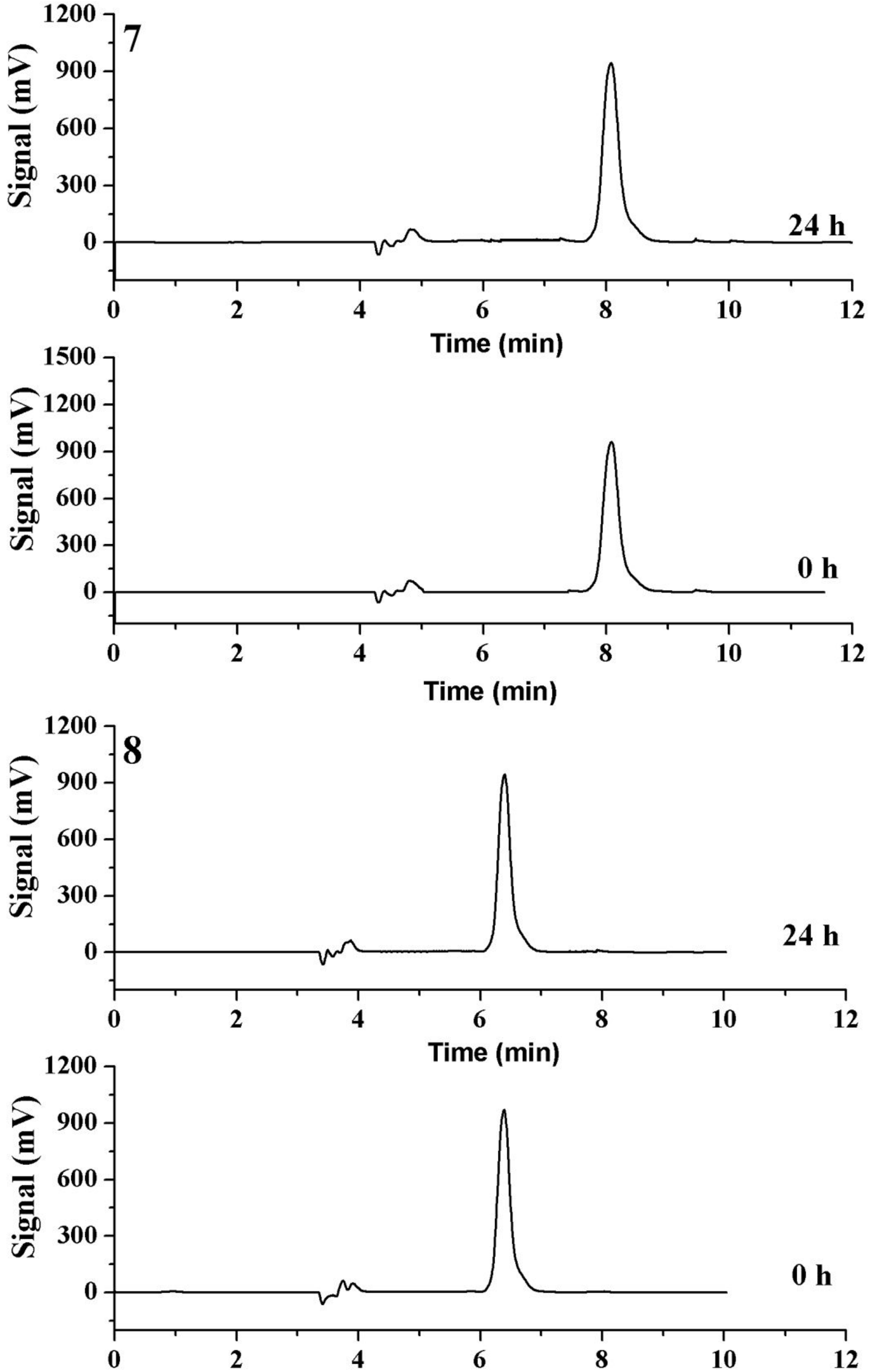
7
Signal (mV)
1200
900
600
300
0
24 h
0
2
4
6
8
10
12
Time (min)
Signal (mV)
1500
1200
900
600
300
0
0 h
0
2
4
6
8
10
12
Time (min)
8
Signal (mV)
1200
900
600
300
0
24 h
0
2
4
6
8
10
12
Time (min)
Signal (mV)
1200
900
600
300
0
0 h
0
2
4
6
8
10
12
Time (min)

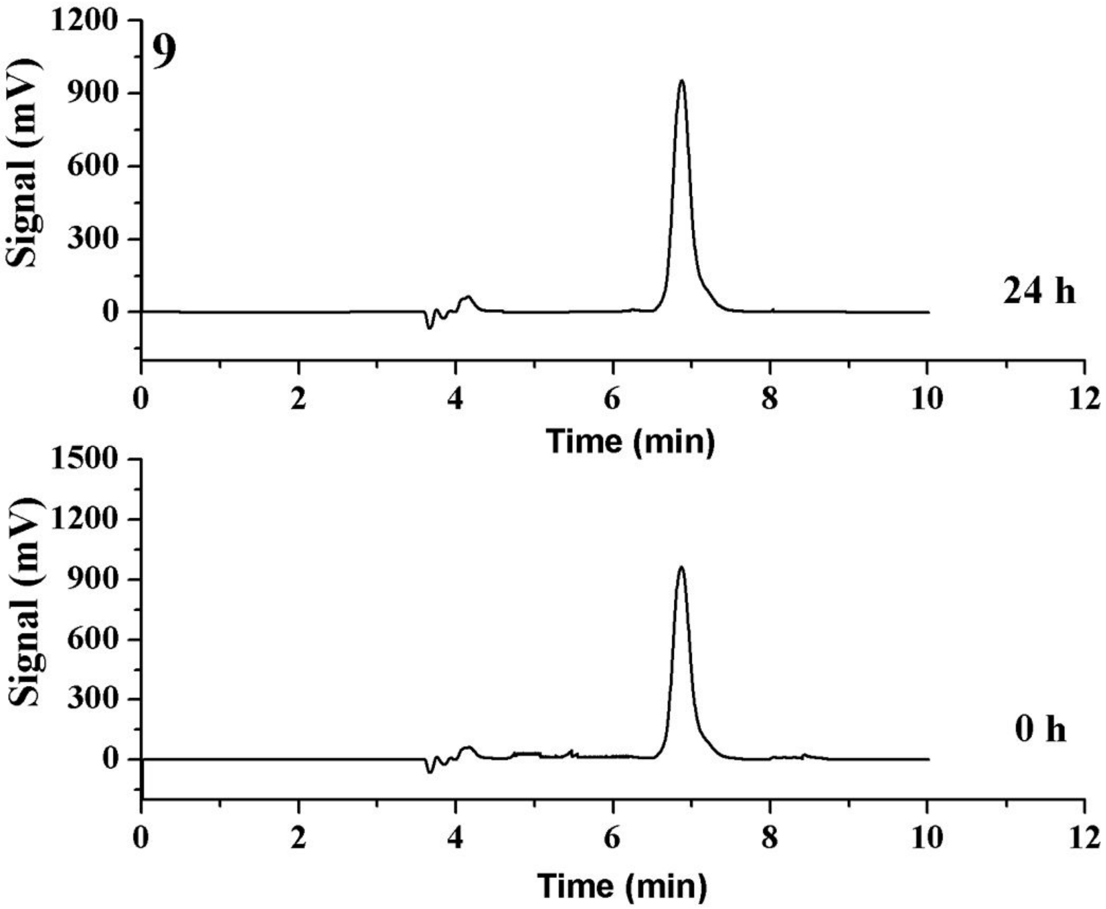
9
Signal (mV)
1200
900
600
300
0
24 h
0
2
4
6
8
10
12
Time (min)
Signal (mV)
1500
1200
900
600
300
0
0 h
0
2
4
6
8
10
12
Time (min)

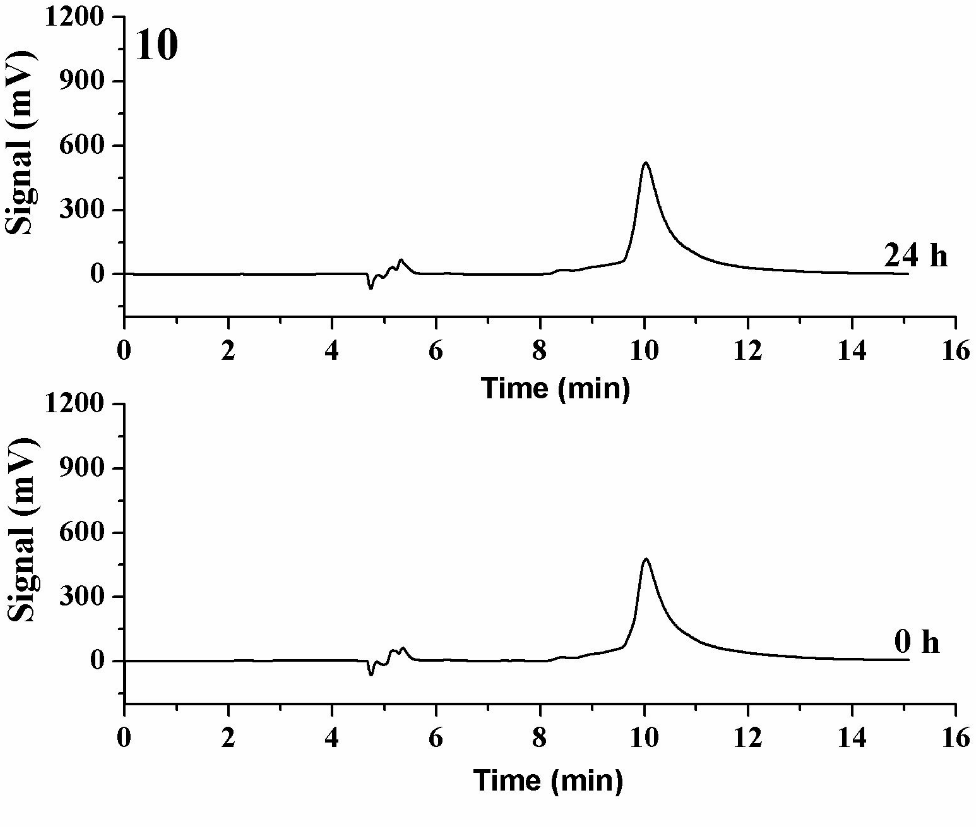
10
Signal (mV)
1200
900
600
300
0
24 h
0
2
4
6
8
10
12
14
16
Time (min)
Signal (mV)
1200
900
600
300
0
0 h
0
2
4
6
8
10
12
14
16
Time (min)

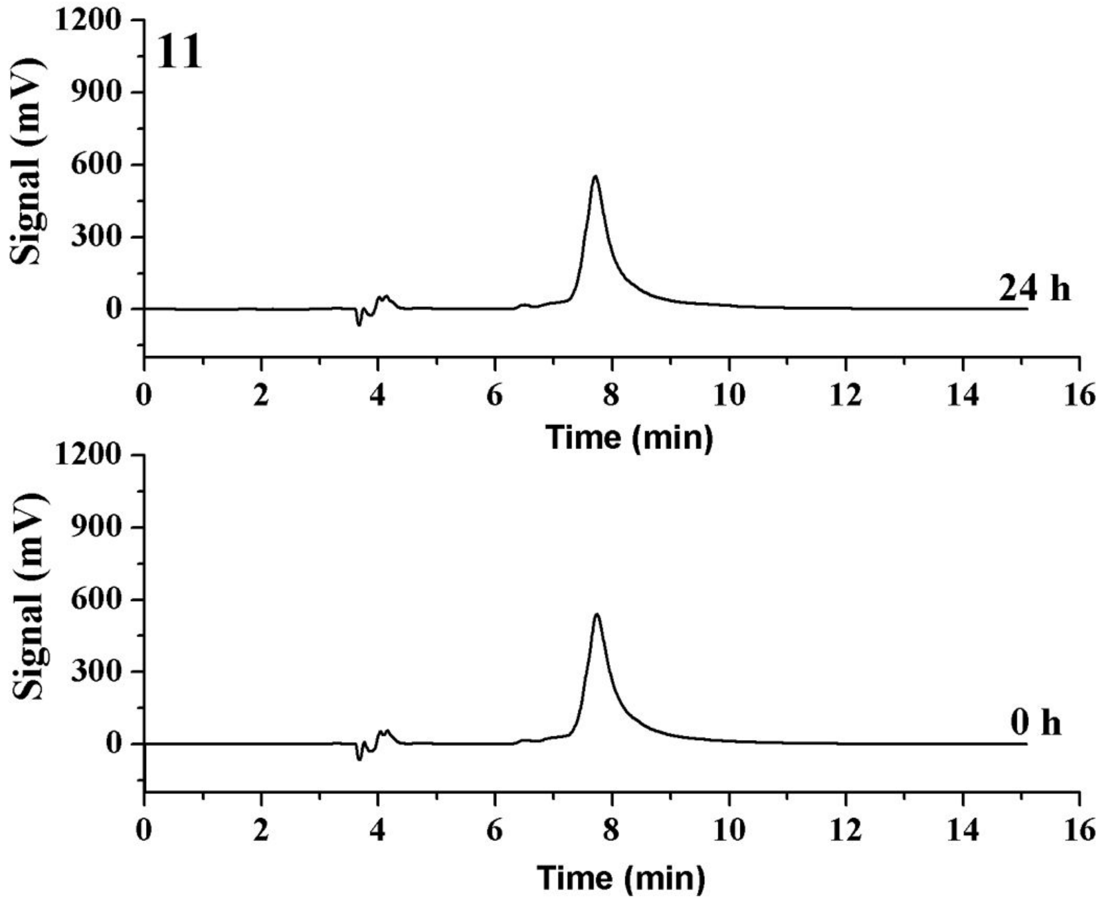
11
Signal (mV)
1200
900
600
300
0
24 h
0 h
0
2
4
6
8
10
12
14
16
Time (min)

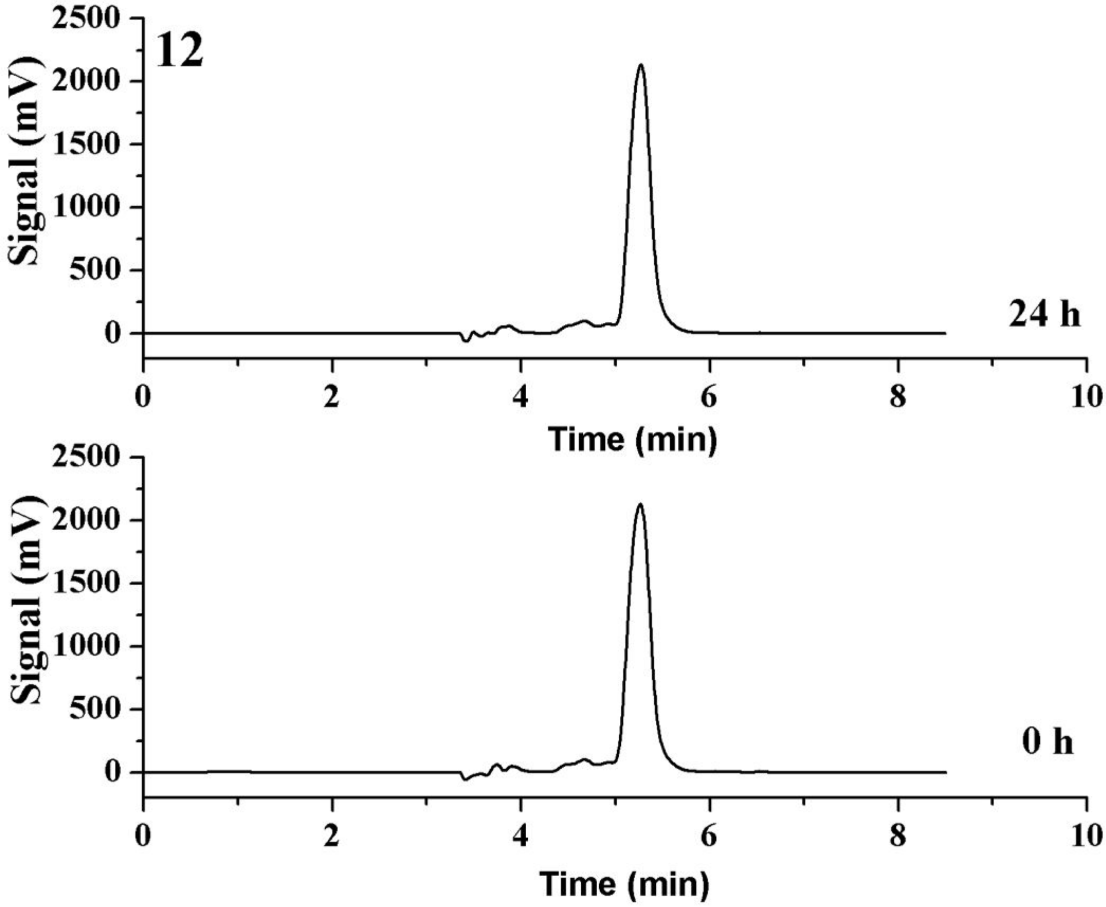
12
Signal (mV)
2500
2000
1500
1000
500
0
24 h
0 h
0
2
4
6
8
10
Time (min)

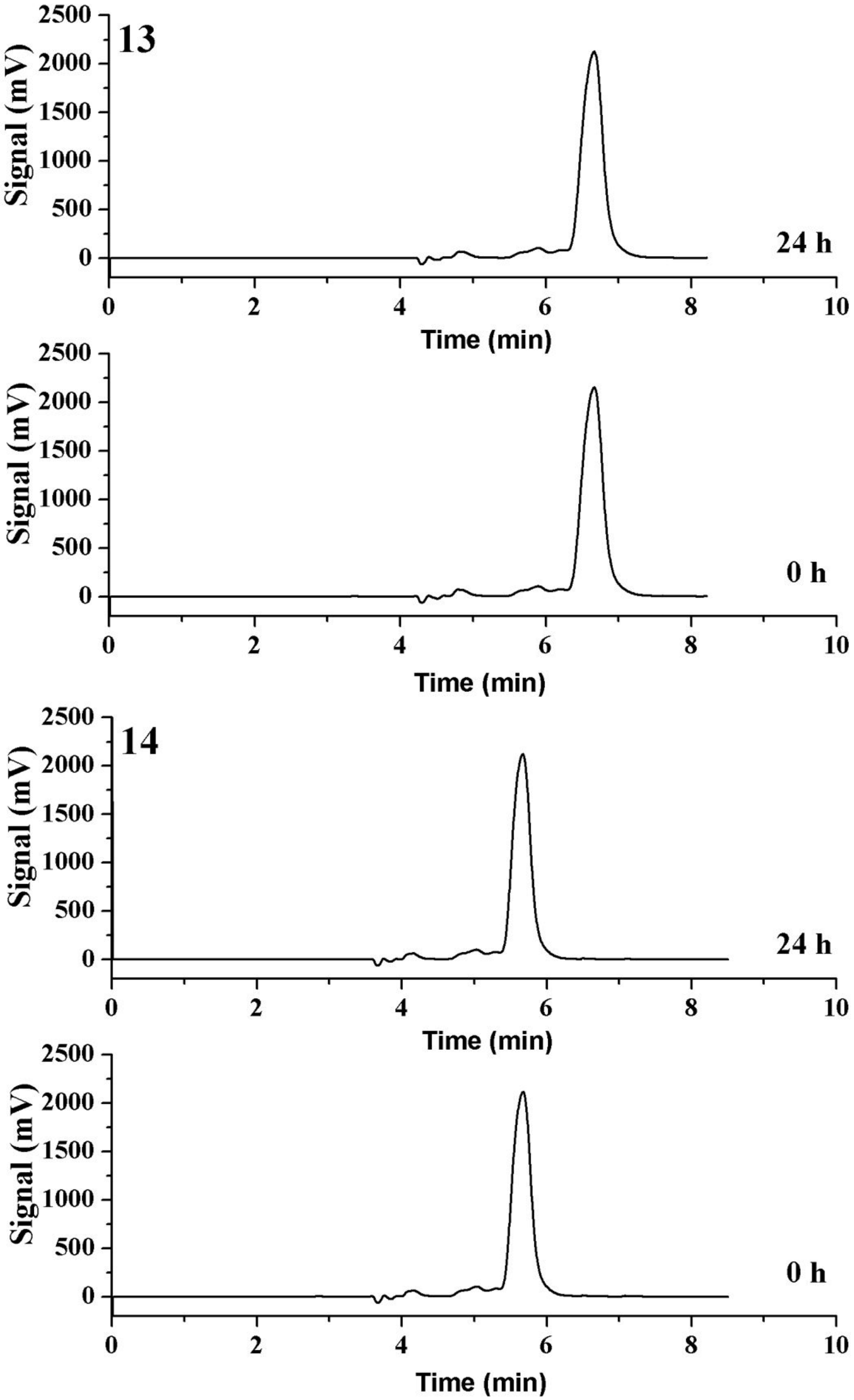

**Figure S68.** HPLC spectra for **1–14** in DMSO ($2.0\times10^{-3}$ M) with 0 h and 24 h.

Column: Inertsustain C18 column (LC-20AT/SPD-20A (or UV2302II/P2302II) HPLC COLUMN, 150 mm×5.0 μm I.D.). Column temperature: 37.0 ºC. Mobile phase: methol/$H_2O$ containing 0.01% TFA (90:10 methol/$H_2O$). Flow rate: 1.0 mL/min. Injection volume: 2. 0×$10^{-4}$ M.

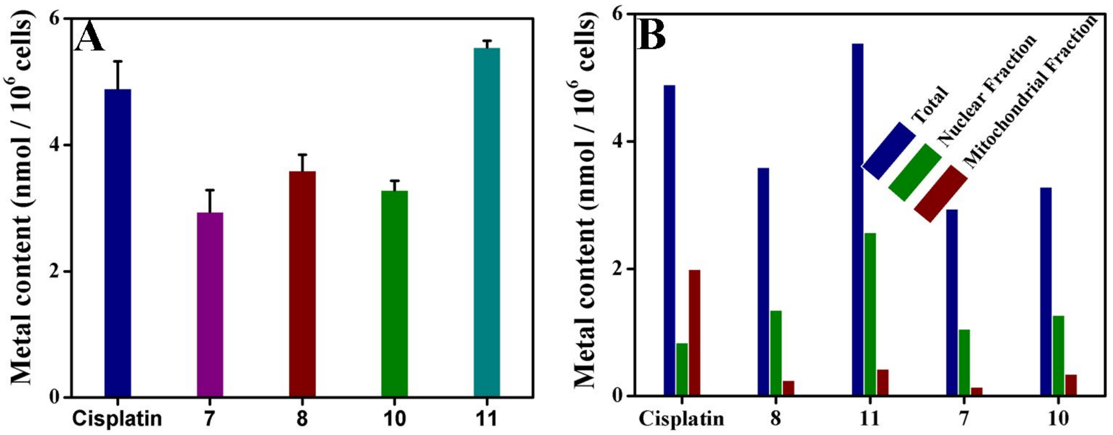


**Figure S69.** Hep-G2 cells were treated with cisplatin (10 $\mu$M), **7** (16 $\mu$M), **8** (10 $\mu$M), **10** (12 $\mu$M), and **11** (6 $\mu$M) for 24 h at 37 °C, respectively. Metal contents in whole cell (A) and in different fractions (B) were measured by ICP-MS. Control cells were treated with vehicle (1% DMSO). Data shown are mean values ± standard deviations of three independent measurements for each experiment.

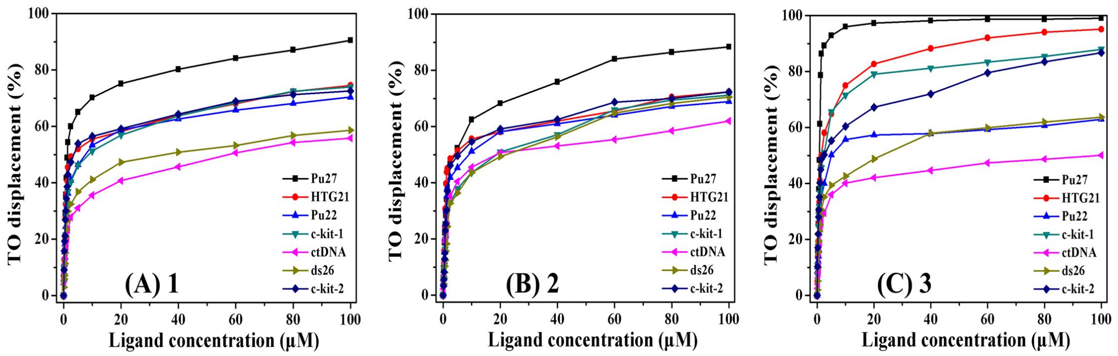


**Figure S70.** FID results onto **1**–**3** toward DNA in sodium cacodylate buffer solution.

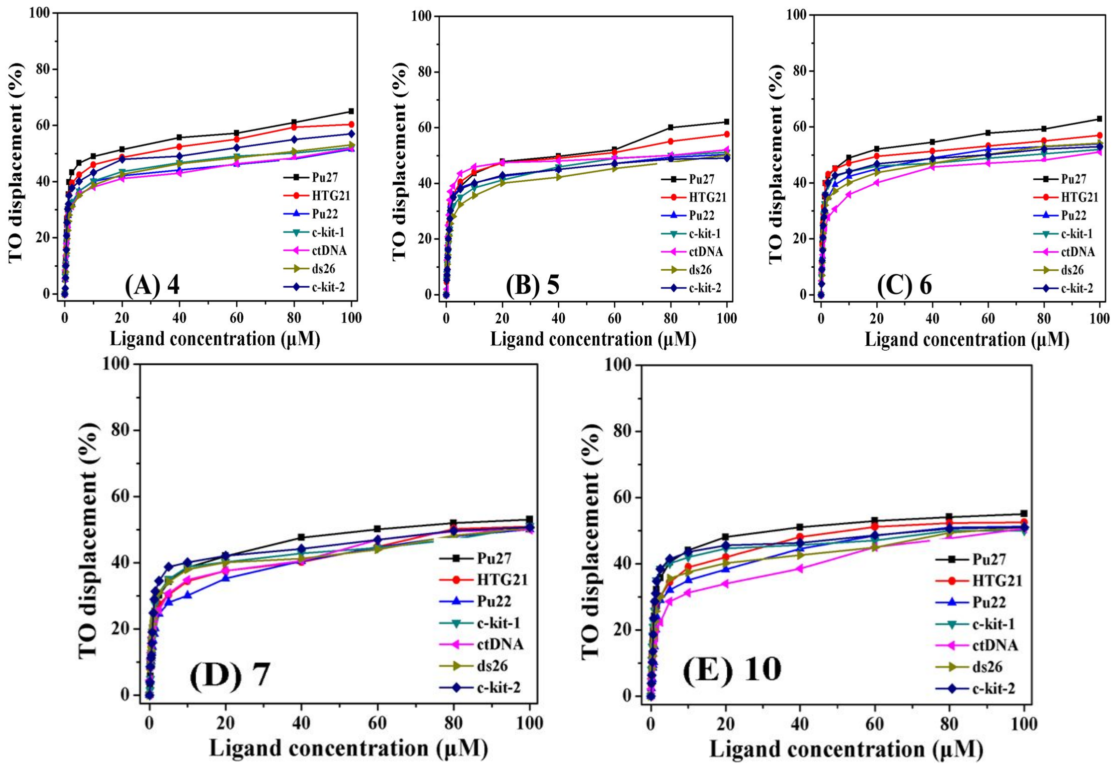


**Figure S71.** FID results onto **4**–**6**, **7** and **10** toward DNA in sodium cacodylate buffer solution.

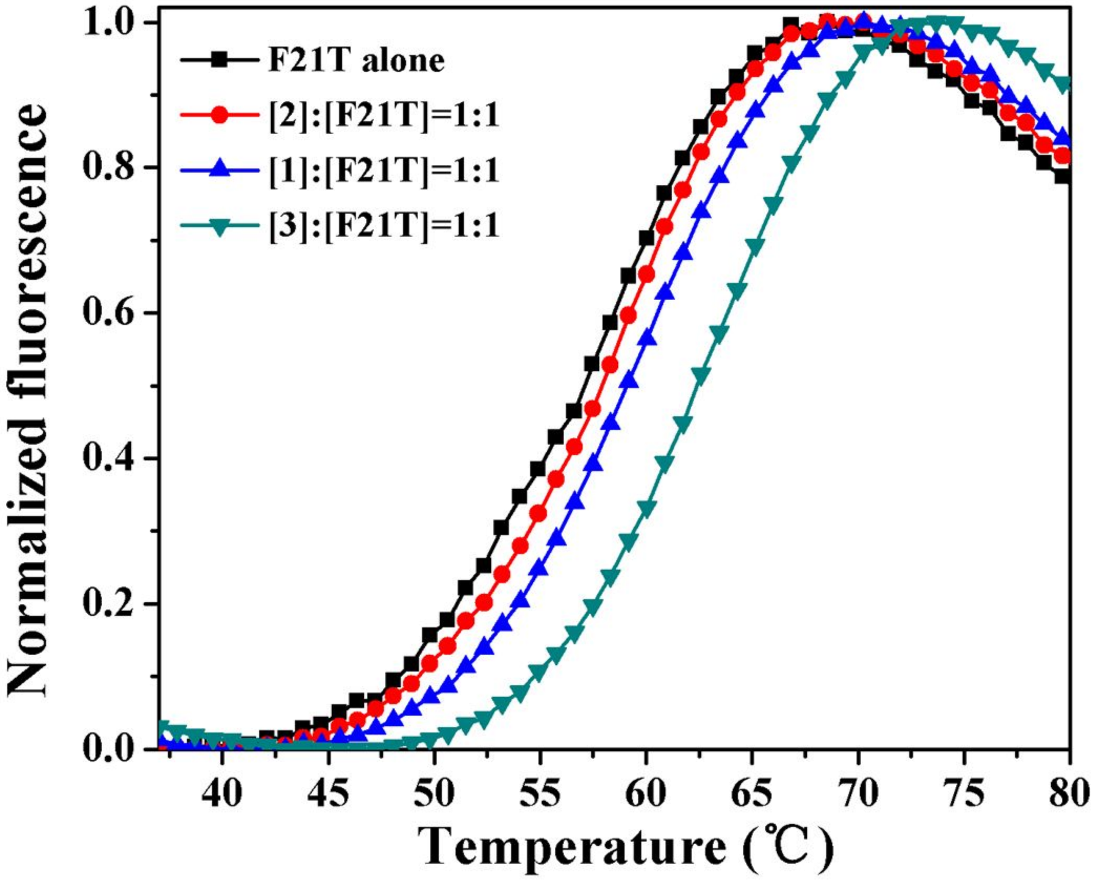


**Figure S72.** FRET melting curves for experiments carried out with F21T (1.0 μM in 10 mM Tris-HCl, 100 mM KCl, pH 7.35) with **1**–**3** binding to F21T.

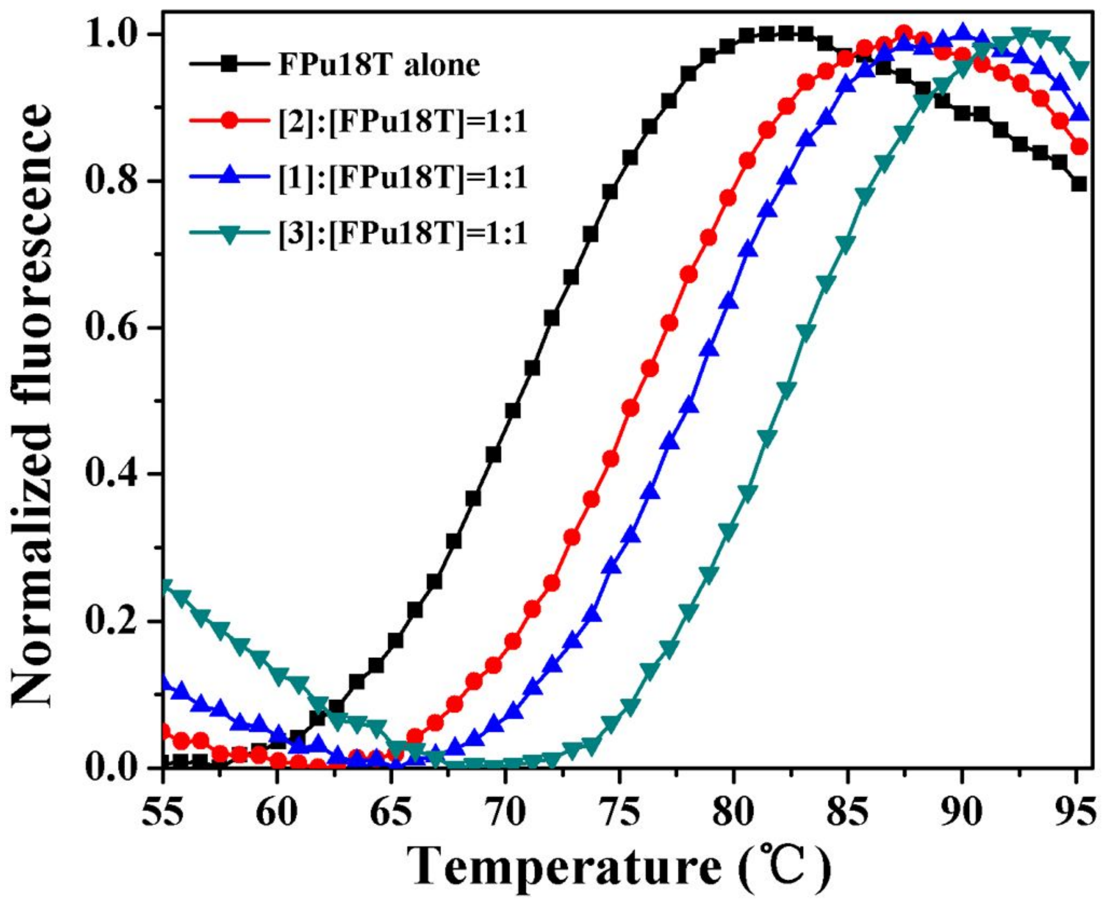


**Figure S73**. FRET melting curves for experiments carried out with FPu18T (1.0 μM in 10 mM Tris-HCl, 100 mM KCl, pH 7.35) with binding to FPu18T.

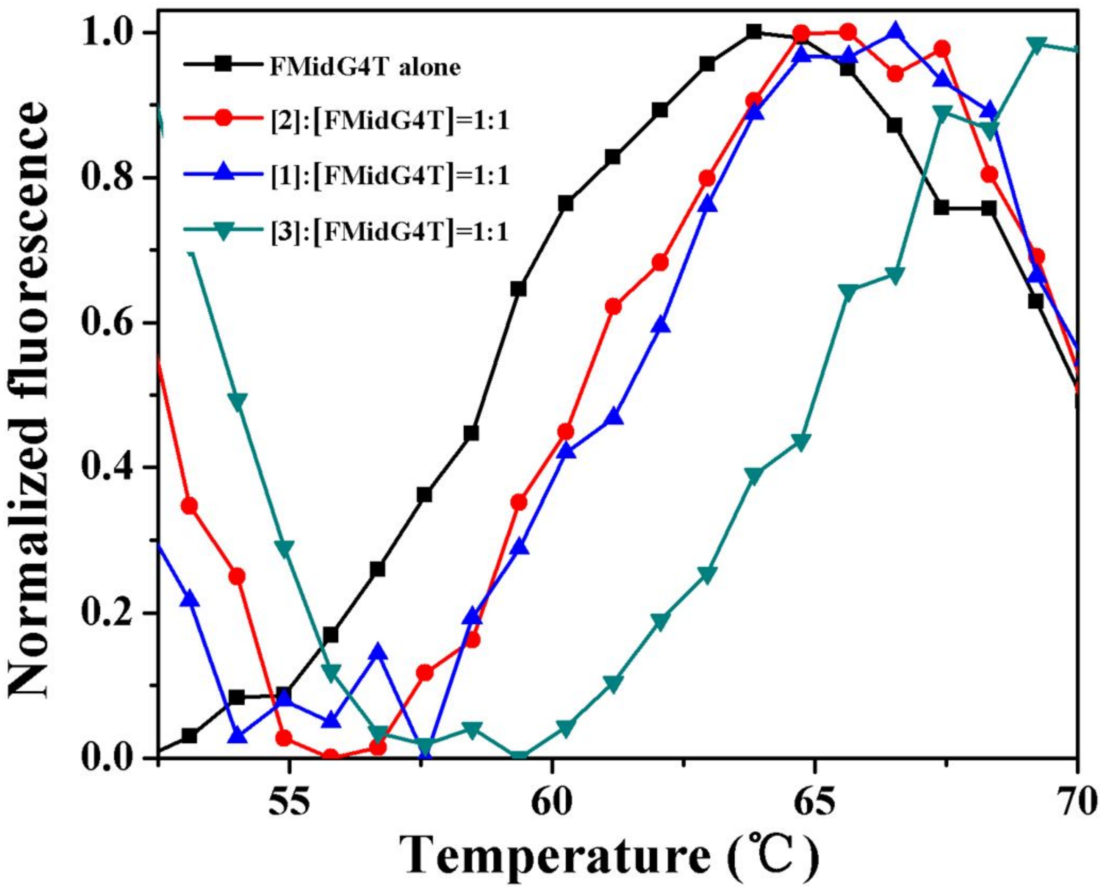


**Figure S74.** FRET melting curves for experiments carried out with FMidG4T (1.0 μM in 10 mM Tris-HCl, 100 mM KCl, pH 7.35) with **1**–**3** binding to FMidG4T, respectively.

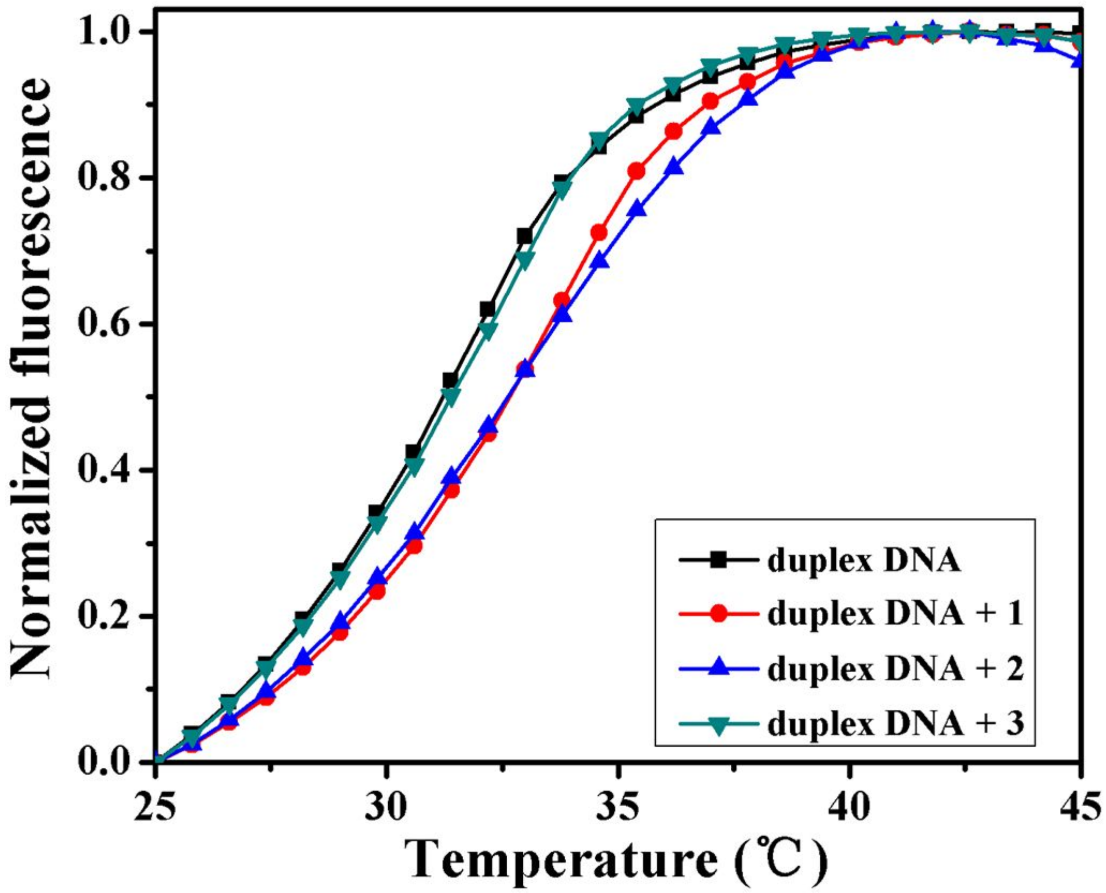


**Figure S75.** FRET melting curves for experiments carried out with duplex DNA (F32T+H20M, 1.0 μM in 10 mM Tris-HCl, 100 mM KCl, pH 7.35) with **1**–**3** binding to duplex DNA, respectively.

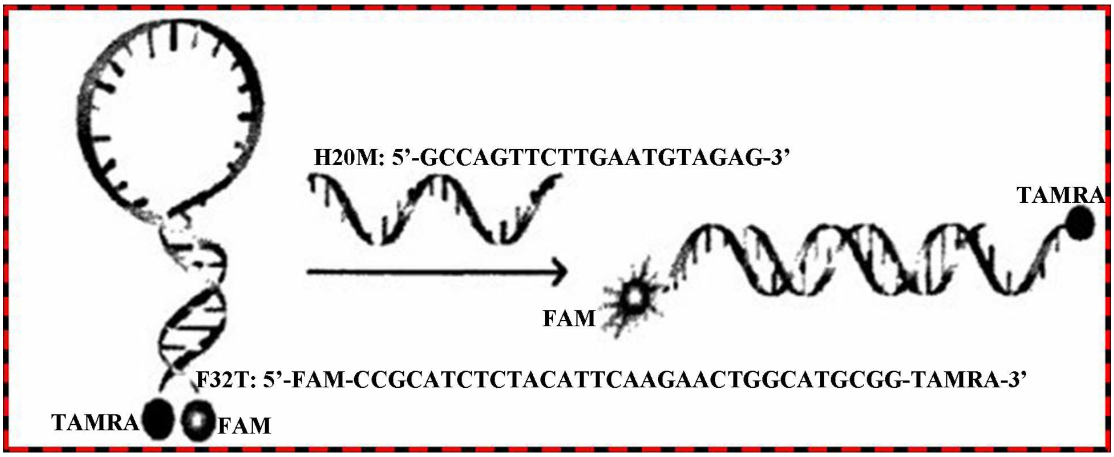


**Figure S76.** Duplex DNA (F32T: 5’-FAM-CCGCATCTCTACATTCAAGAACTGGCATGCGG-TAMRA-3’; H20M: 5’-GCCAGTTCTTGAATGTAGAG-3’).

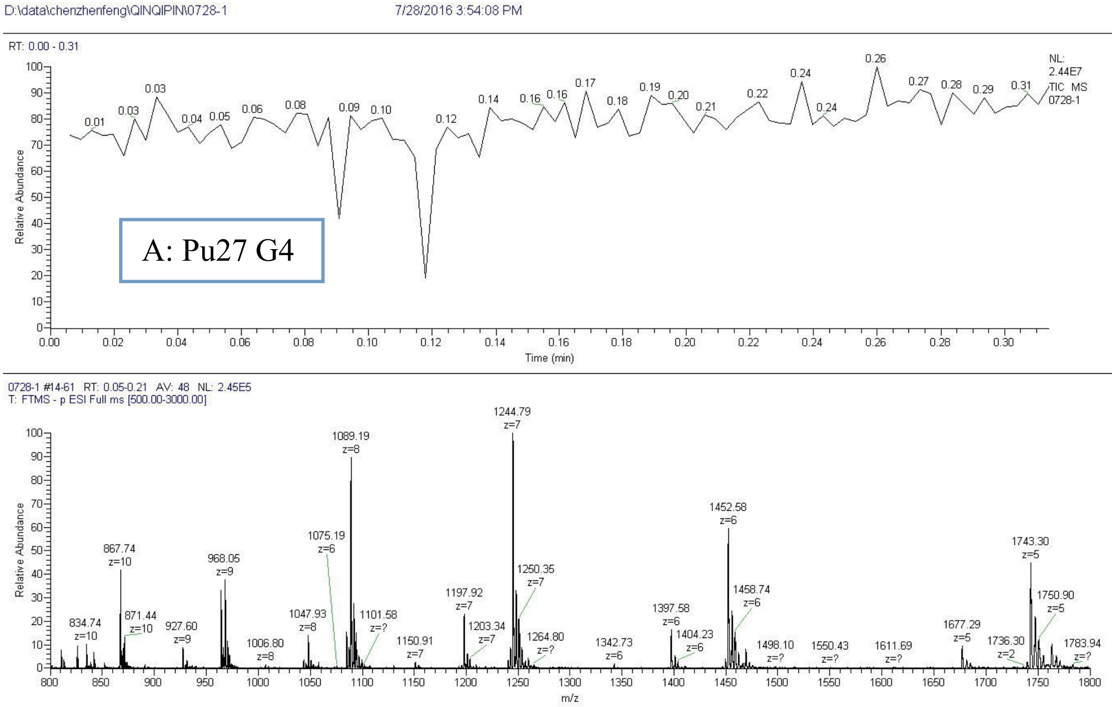

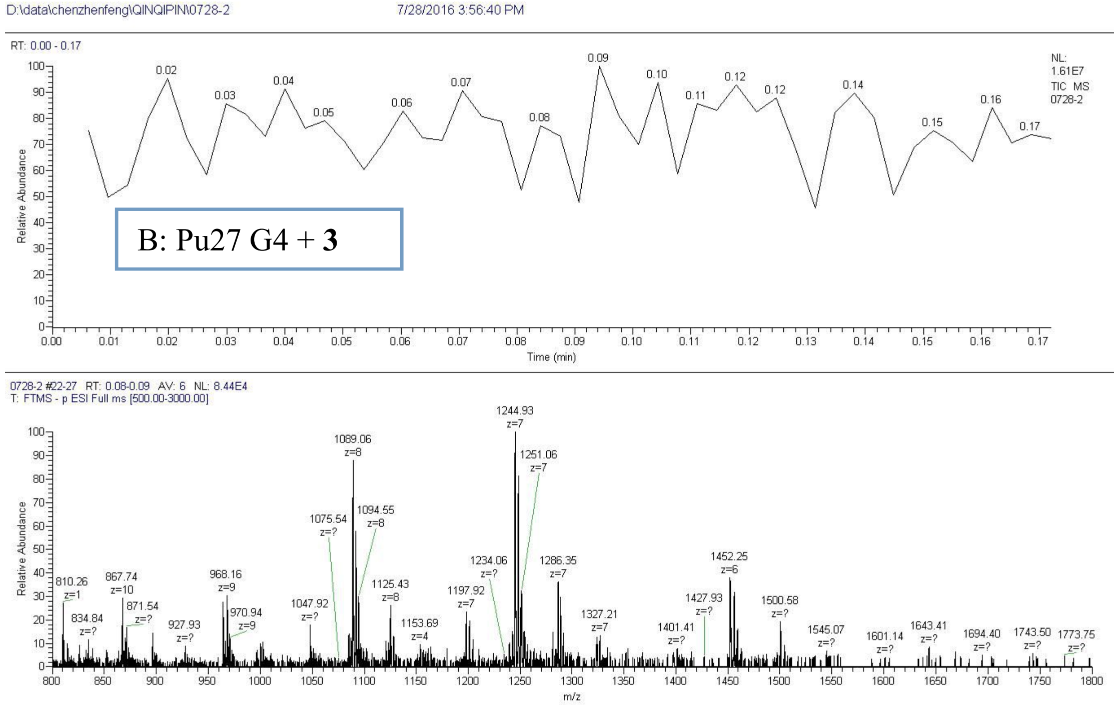
D:\data\chenzhenfeng\QINQIPIN\0728-2
7/28/2016 3:56:40 PM
RT: 0.00 - 0.17
NL: 1.61E7 TIC MS 0728-2
B: Pu27 G4 + **3**
Relative Abundance
Time (min)
0728-2 #22-27 RT: 0.08-0.09 AV: 6 NL: 8.44E4
T: FTMS - p ESI Full ms [500.00-3000.00]
1244.93 z=7
1089.06 z=8
1251.06 z=7
1094.55 z=8
1075.54 z=?
1234.06 z=?
1286.35 z=7
1452.25 z=6
968.16 z=9
867.74 z=10
810.26 z=1
1125.43 z=8
1197.92 z=7
1427.93 z=?
1500.58 z=?
m/z
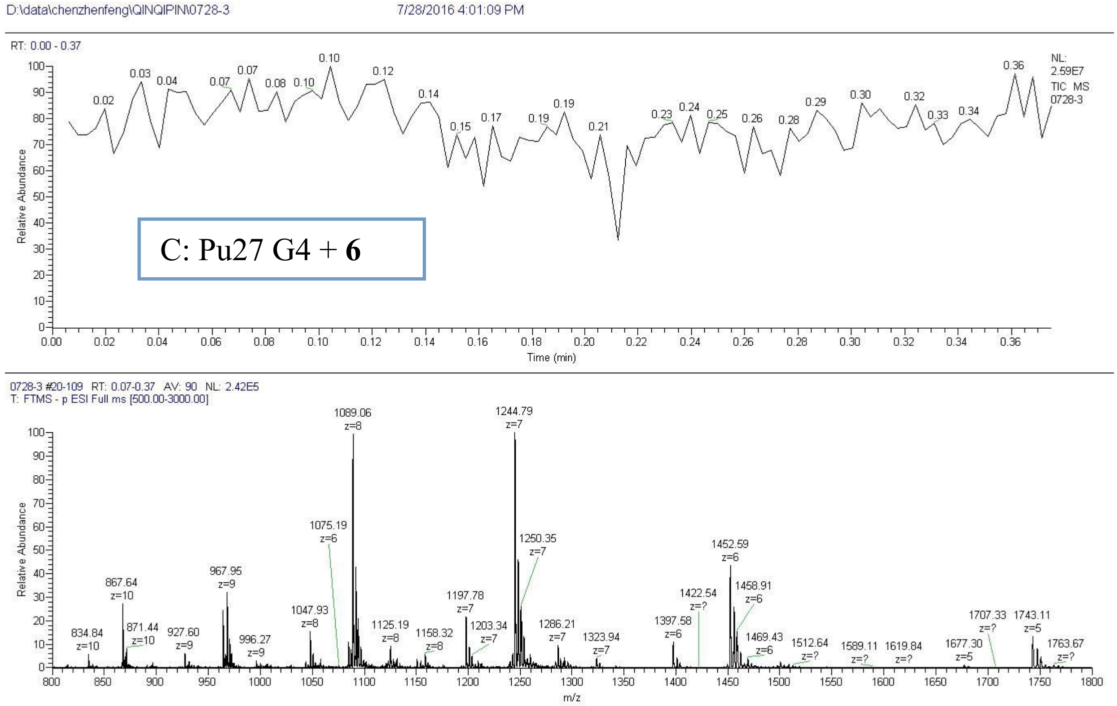
D:\data\chenzhenfeng\QINQIPIN\0728-3
7/28/2016 4:01:09 PM
RT: 0.00 - 0.37
NL: 2.59E7 TIC MS 0728-3
C: Pu27 G4 + **6**
Relative Abundance
Time (min)
0728-3 #20-109 RT: 0.07-0.37 AV: 90 NL: 2.42E5
T: FTMS - p ESI Full ms [500.00-3000.00]
1089.06 z=8
1244.79 z=7
1075.19 z=6
1250.35 z=7
1452.59 z=6
967.95 z=9
867.64 z=10
1458.91 z=6
1422.54 z=?
1197.78 z=7
1047.93 z=8
1743.11 z=5
1707.33 z=?
m/z

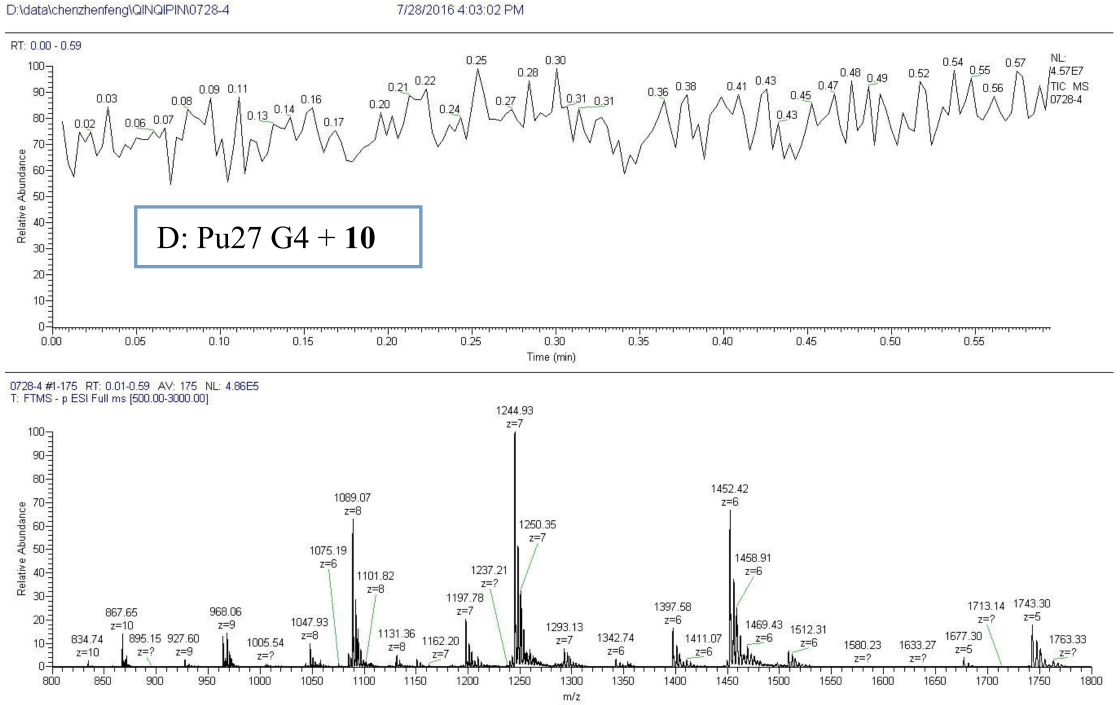


Figure S77. ESI-MS spectrum of the Pu27 G-quadruplex (A) and it cross-linked complexes products (B–D).

**Table S10.** The changes of CD spectra of binding to three G-quadruplexes DNA by **1–3**.

| compds | DNA | Extent of change at 258—268 nm (%) | Extent of change at 290—295 nm (%) |
|---|---|---|---|
| **1** | G4-HTG21 | 53.33↓ | 22.33↓ (—) |
| | G4-Pu27 | 125.00↓ | — |
| | G4-Pu39 | 43.85↓ | — |
| **2** | G4-HTG21 | 48.63↓ | 8.64↓ (—) |
| | G4-Pu27 | 51.14↓ | — |
| | G4-Pu39 | 20.44↓ | — |
| **3** | G4-HTG21 | 96.08↓ | 55.99↓ (—) |
| | G4-Pu27 | 146.59↓ | — |
| | G4-Pu39 | 48.02↓ | — |

"—"represents no obvious changes in the peaks.

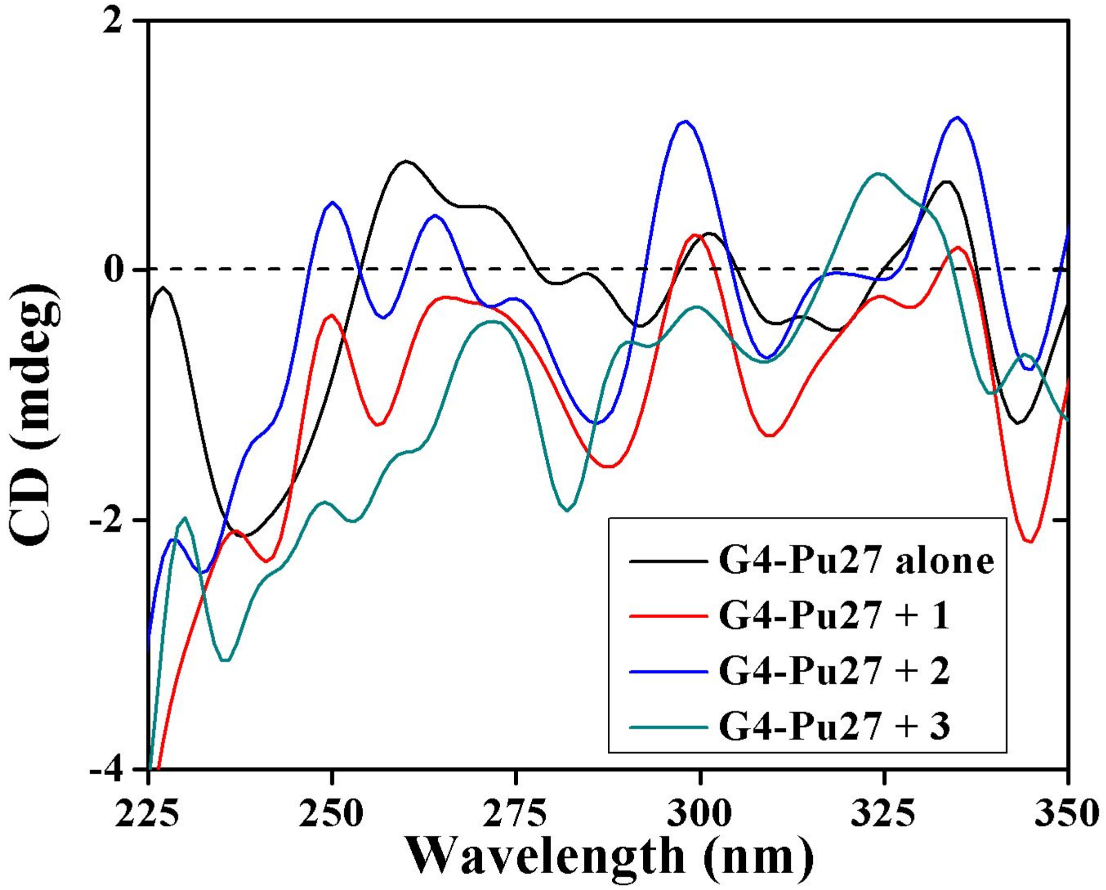


**Figure S78.** CD spectra of G-quadruplex Pu27 (1 $\mu$M) in the presence of $K^+$ upon addition of **1–3** (1 $\mu$M).

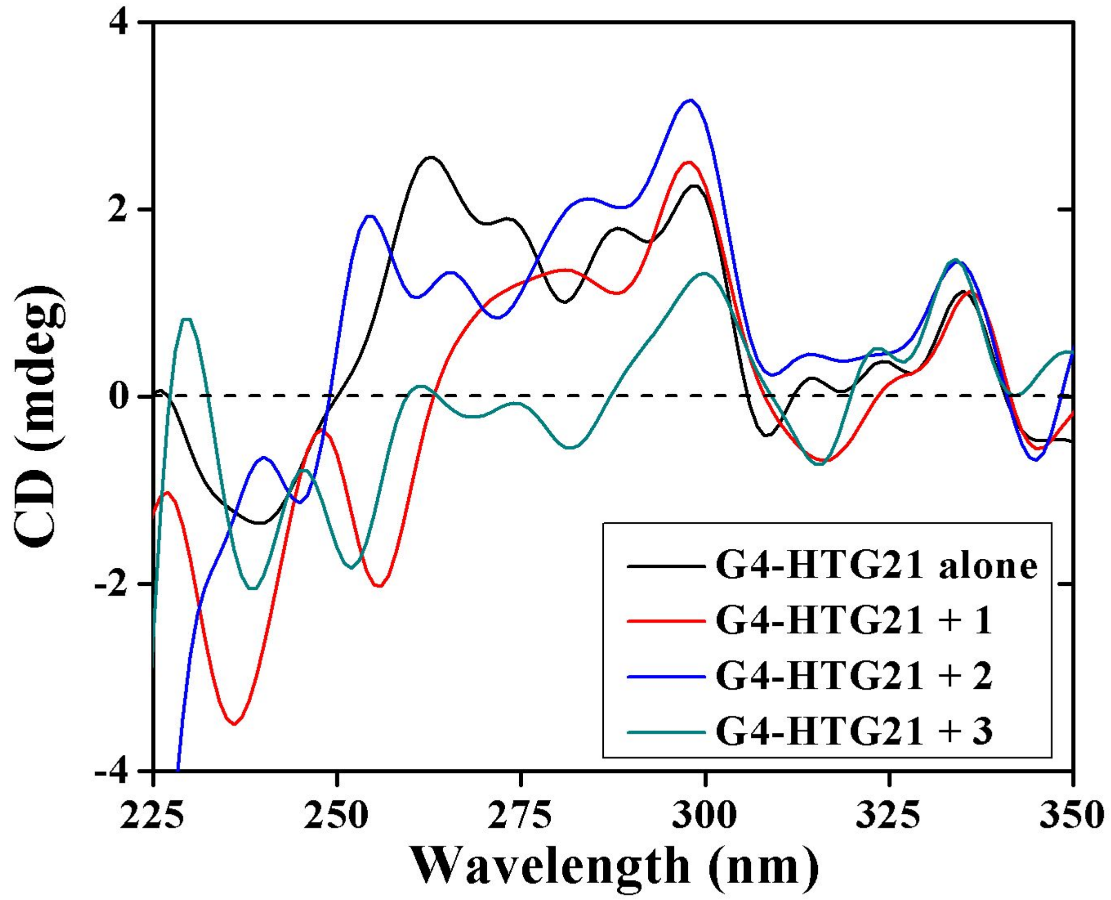


**Figure S79.** CD spectra of Human Telomeric G-quadruplex DNA (1 $\mu$M) in the presence of $K^+$ upon addition of **1–3** (1 $\mu$M).

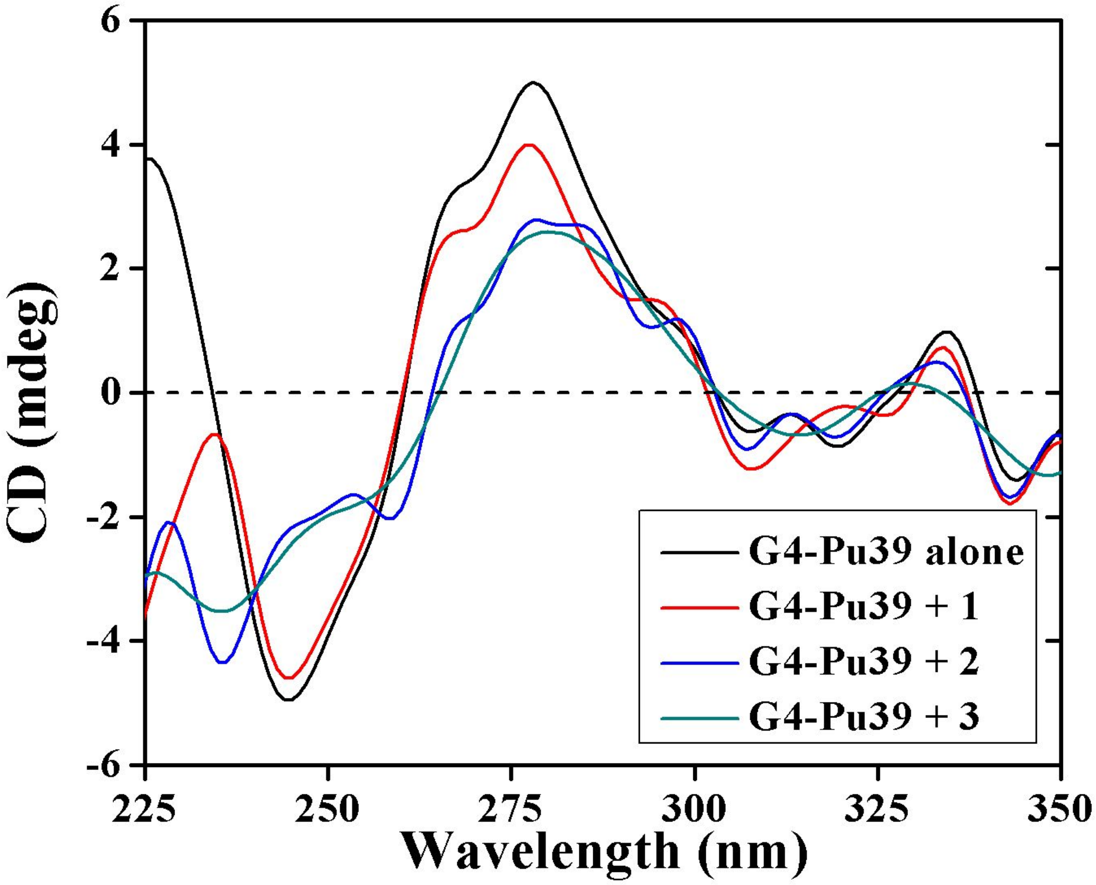


**Figure S80.** CD spectra of G-quadruplex Pu39 (1 $\mu$M) in the presence of $K^+$ upon addition of **1–3** (1 $\mu$M).

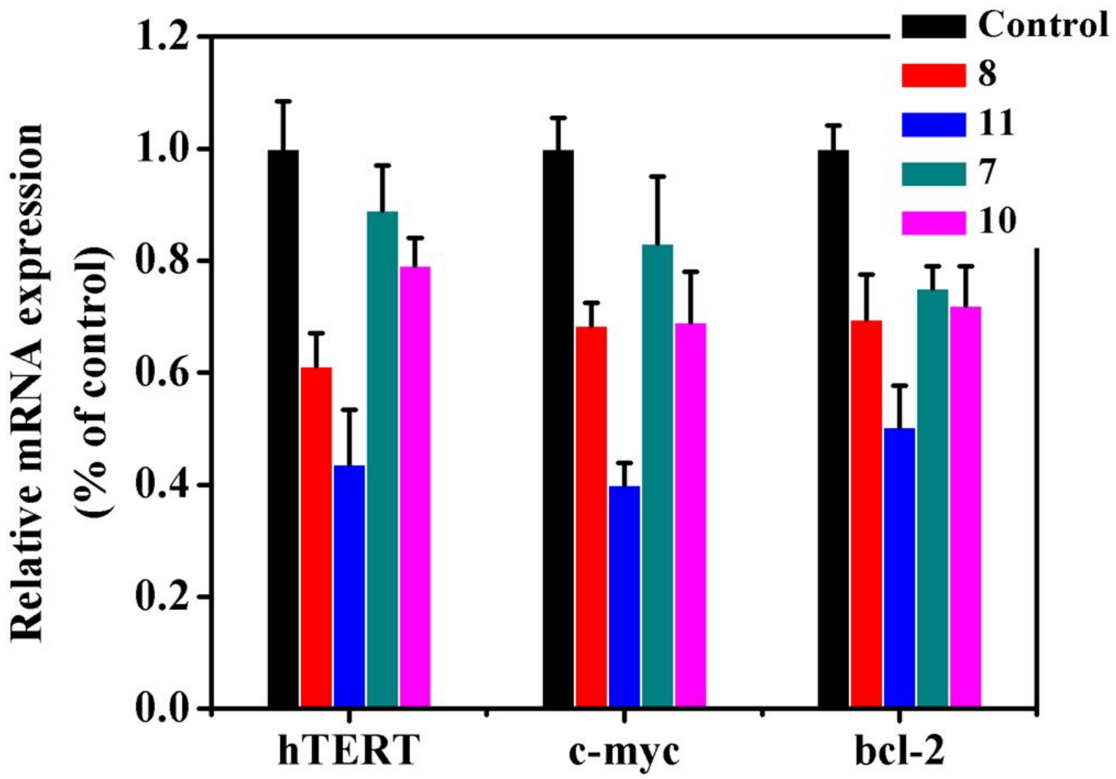


**Figure S81.** qRT-PCR was used to determine the expression of hTERT, c-myc and bcl-2 in the Hep-G2 cells treated with **7** (16 $\mu$M), **8** (10 $\mu$M), **10** (12 $\mu$M), and **11** (6 $\mu$M), respectively. $5\times10^5$ Hep-G2 cells were treated in a 25-$cm^2$ flask with medium (no drug), **7** (16 $\mu$M), **8** (10 $\mu$M), **10** (12 $\mu$M), and **11** (6 $\mu$M) for 24 h, and the total RNA was extracted and subjected to reverse transcription, followed by PCR for hTERT, c-myc or bcl-2 and GAPDH (control).

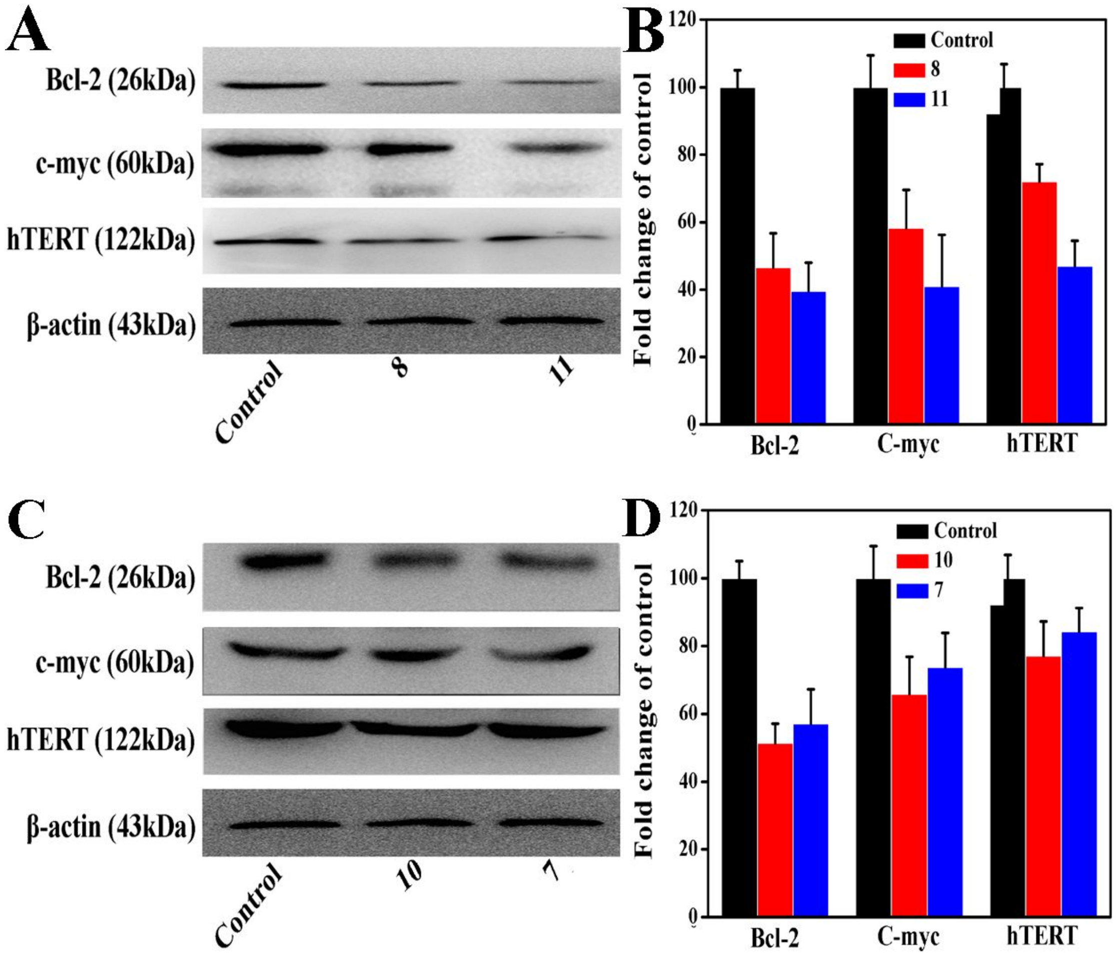


**Figure S82.** Western blot was used to determine the expression of hTERT, c-myc, and bcl-2 in Hep-G2 cells treated with **7** (16 $\mu$M), **8** (10 $\mu$M), **10** (12 $\mu$M), and **11** (6 $\mu$M) for 24 h, respectively. (A and C) hTERT, bcl-2, and c-myc protein levels in Hep-G2 cells were analyzed by western blot. (B and D) The whole-cell extracts were prepared and analyzed by Western blot analysis using antibodies against hTERT, bcl-2, and c-myc. The same blots were stripped and reprobed with β-actin antibody to show equal protein loading. Western blotting bands from three independent measurements were quantified with Image J. in (B and D).

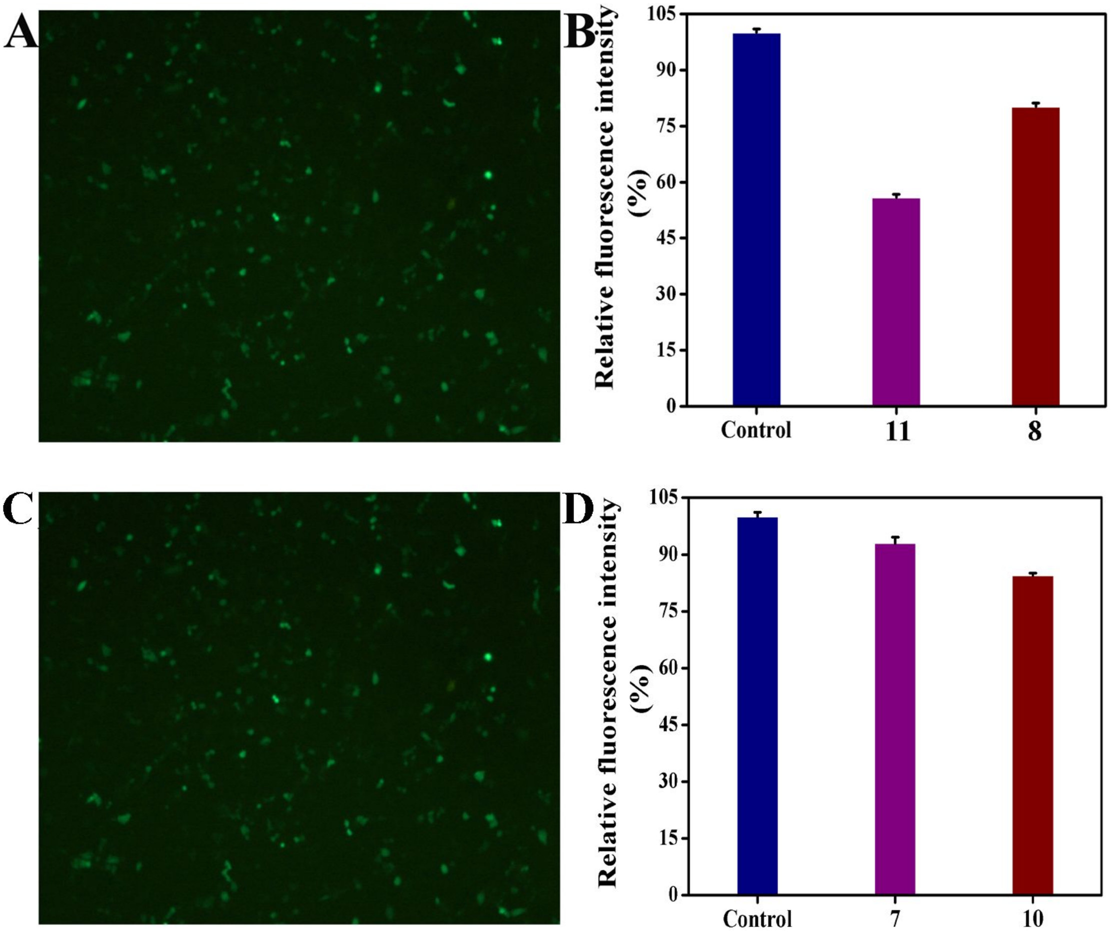


**Figure S83.** The transfection of EGFP (A and C) and c-myc (B and D) plasmid vector in Hep-G2 cells. Complexes **7** (16 $\mu$M), **8** (10 $\mu$M), **10** (12 $\mu$M), and **11** (6 $\mu$M) were added into medium after 6.0 h of transfection from 2.0 $\mu$g c-myc plasmid by Lipofectamine 2000 (Invitrogen) into Hep-G2 cells, respectively. After another 24.0 h of drug treatment, the cells were studied by luciferase reporter gene assay kit.

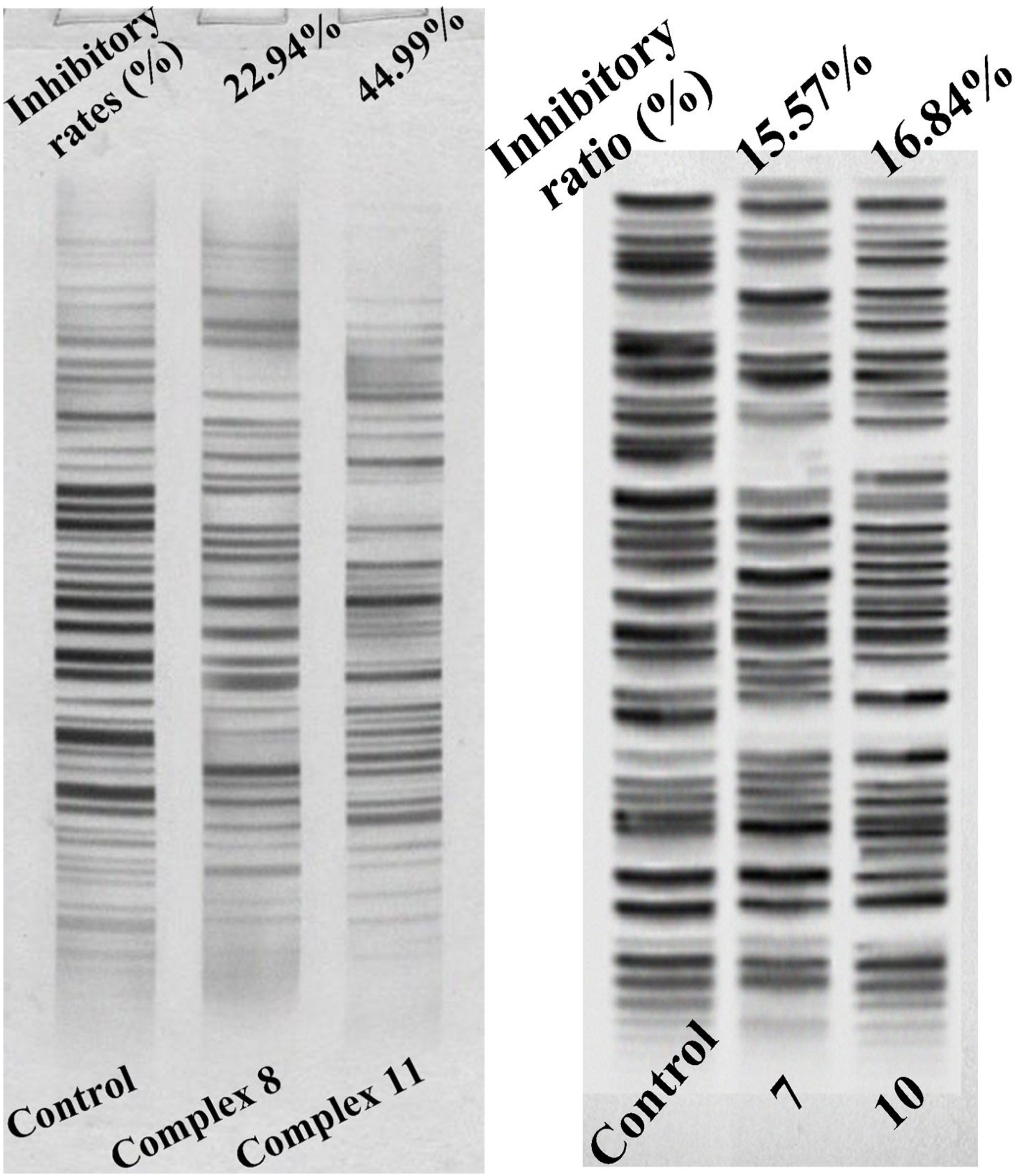


**Figure S84.** The influence of **7** (16 $\mu$M), **8** (10 $\mu$M), **10** (12 $\mu$M), and **11** (6 $\mu$M) on the telomerase activity of the Hep-G2 cells (24 h for each complex, respectively).

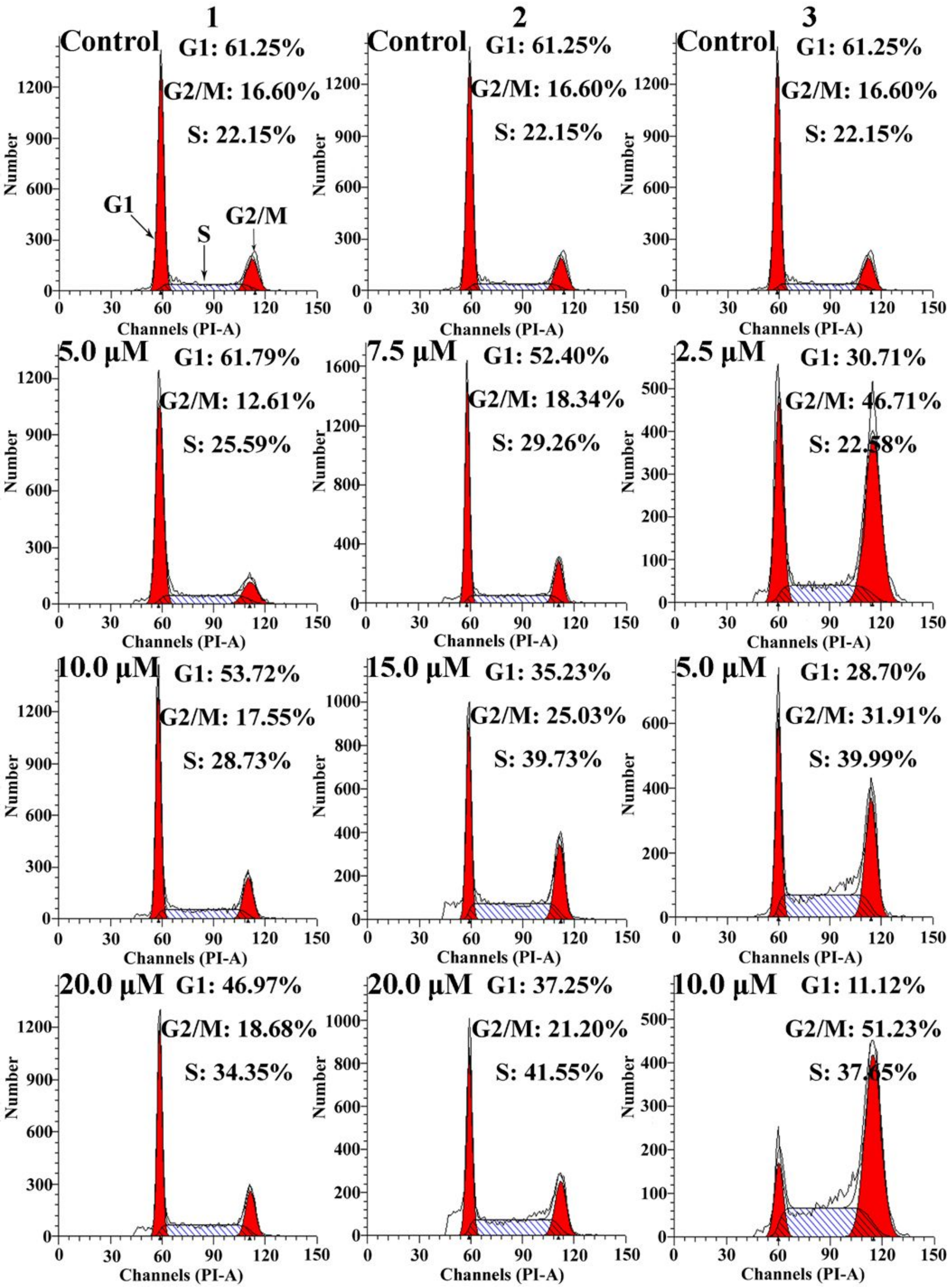


**Figure S85.** Effect of cell cycle of Hep-G2 cells treated with **1**–**3** for 24 h comparing with the control cells.

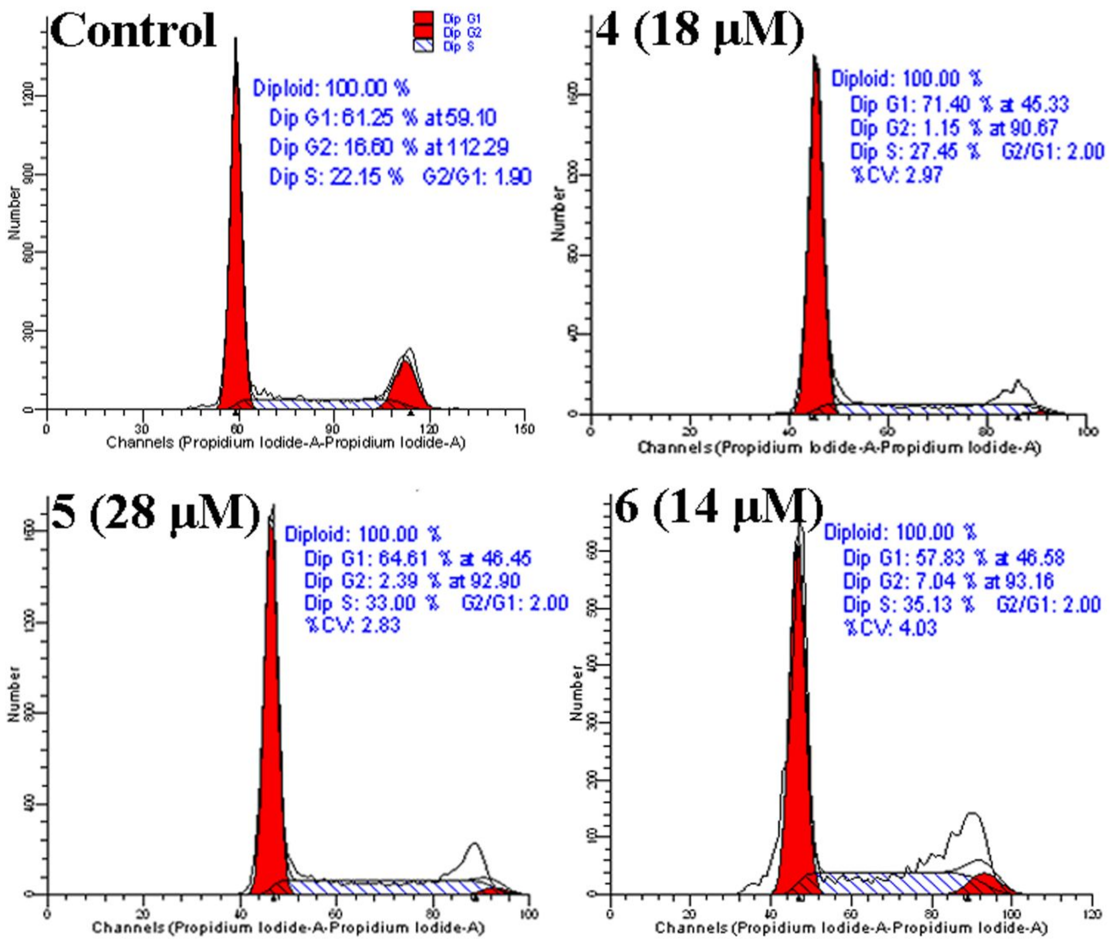


**Figure S86.** Effect of cell cycle of Hep-G2 cells treated with **4**–**6** for 24 h comparing with the control cells.

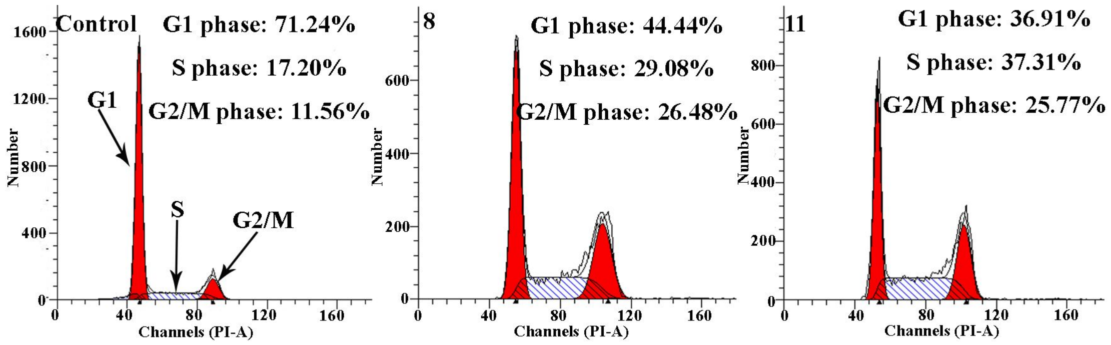


**Figure S87.** Effect of cell cycle of Hep-G2 cells treated with **8** (10 $\mu$M) and **11** (6 $\mu$M) for 24 h comparing with the control cells.

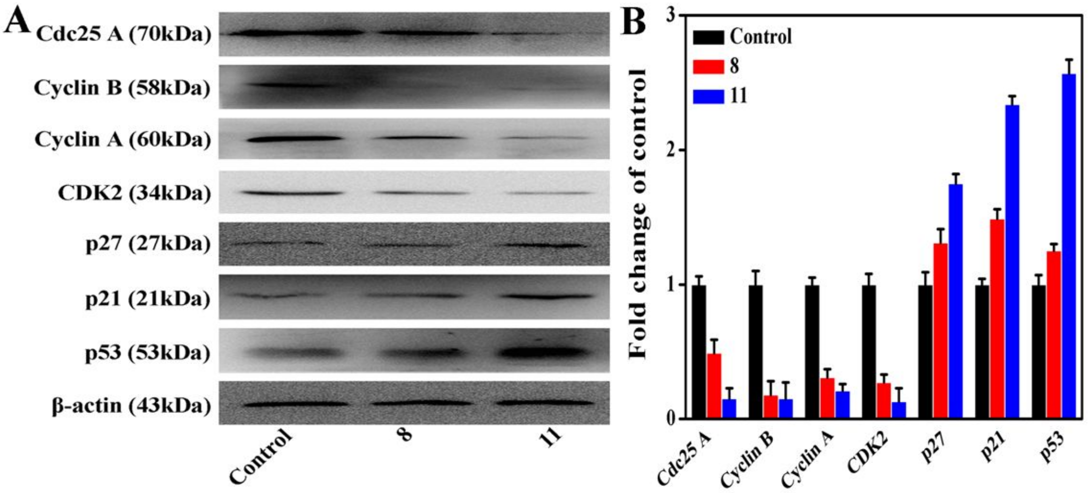


**Figure S88.** Western blot was used to determine the expression of cell cycle protein regulators in Hep-G2 cells treated with **8** (10 $\mu$M) and **11** (6 $\mu$M) for 24 h, respectively. (A) Cell cycle protein regulators protein levels in Hep-G2 cells were analyzed by western blot. (B) The whole-cell extracts were prepared and analyzed by Western blot analysis using antibodies against cell cycle protein regulators proteins. The same blots were stripped and reprobed with β-actin antibody to show equal protein loading. Western blotting bands from three independent measurements were quantified with Image J. in (B).

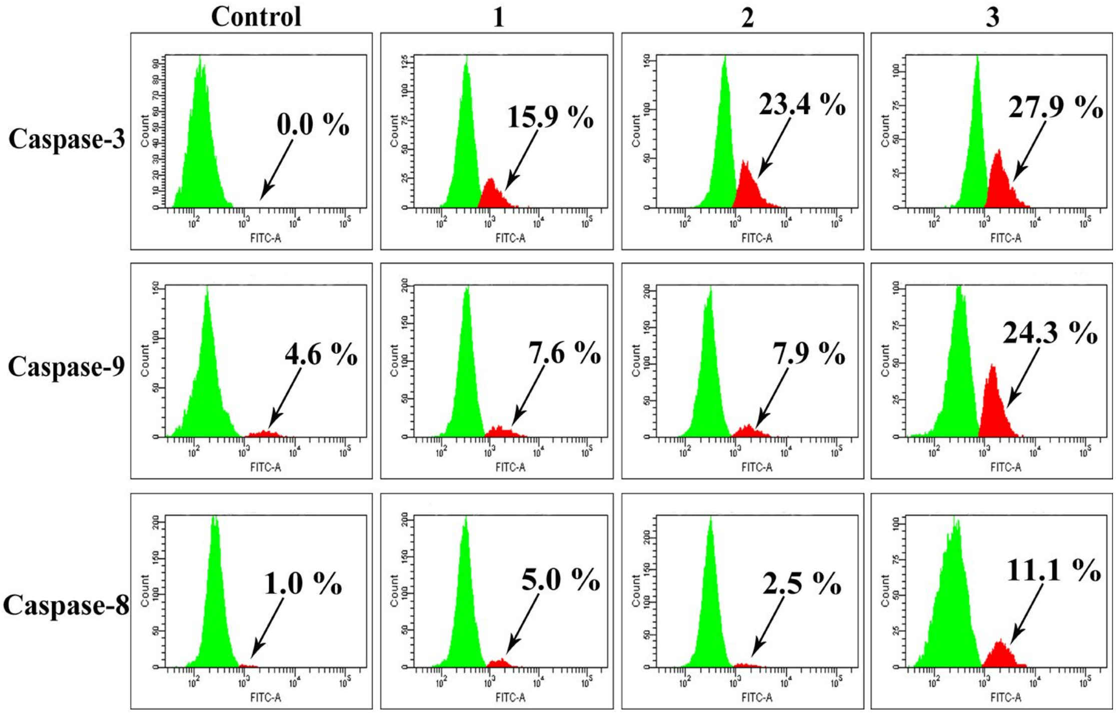


**Figure S89.** The expression levels of caspase-3/8/9 proteins in Hep-G2 cells treated with **1** (8 $\mu$M), **2** (15 $\mu$M) and **3** (5 $\mu$M) for 24 h, respectively. Caspase-3/8/9 were assessed by the CasPGLOW fluorescein activite caspase-3/8/9 staining kits.

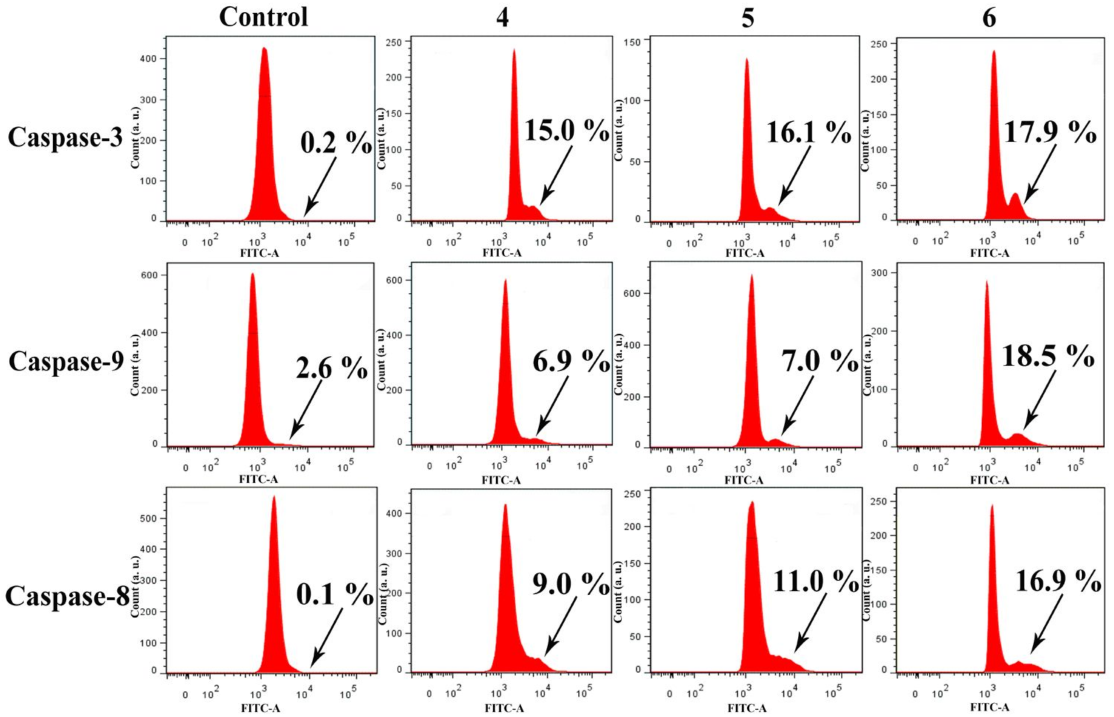


**Figure S90.** The expression levels of caspase-3/8/9 proteins in Hep-G2 cells treated with **4** (18 $\mu$M), **5** (28 $\mu$M) and **6** (14 $\mu$M) for 24 h, respectively. Caspase-3/8/9 were

assessed by the CasPGLOW fluorescein activite caspase-3/8/9 staining kits.

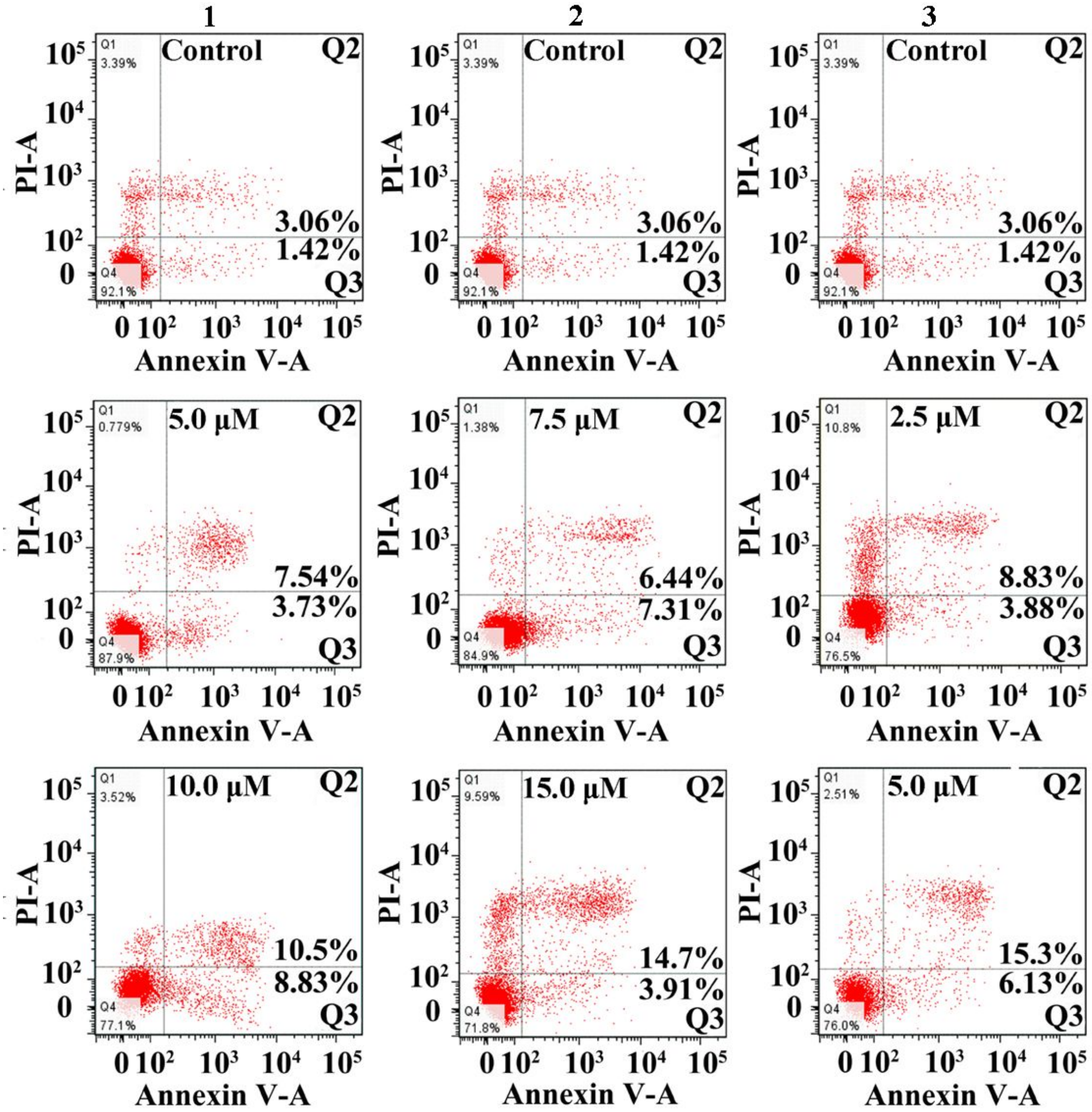


**Figure S91.** Apoptosis of Hep-G2 cells treated with **1** (5, 10 $\mu$M), **2** (7.5, 15 $\mu$M) and **3** (2.5, 5 $\mu$M) for 24 h, comparing with the control cells.

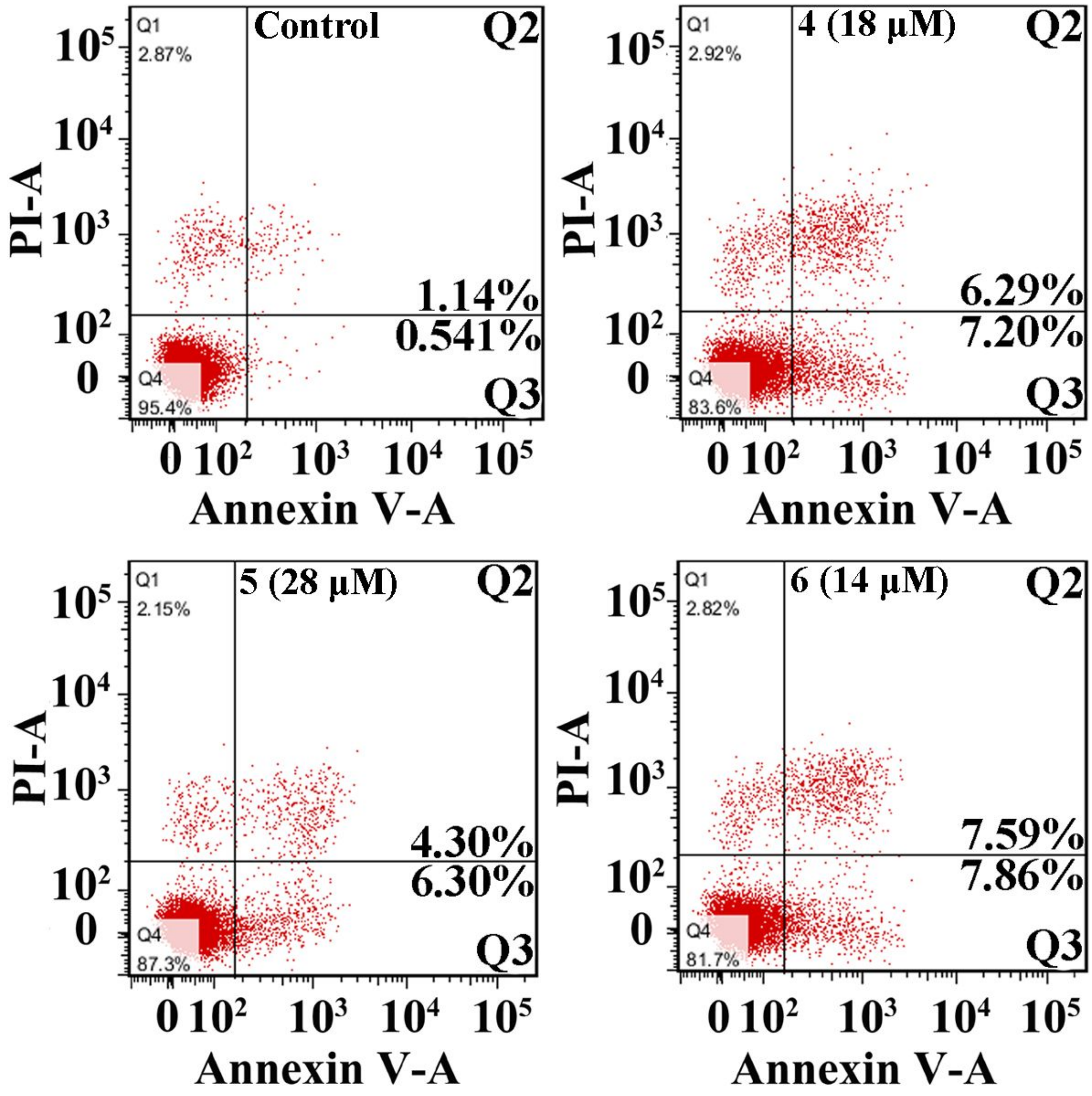


**Figure S92.** Apoptosis of Hep-G2 cells treated with **4**–**6** for 24 h, comparing with the control cells.

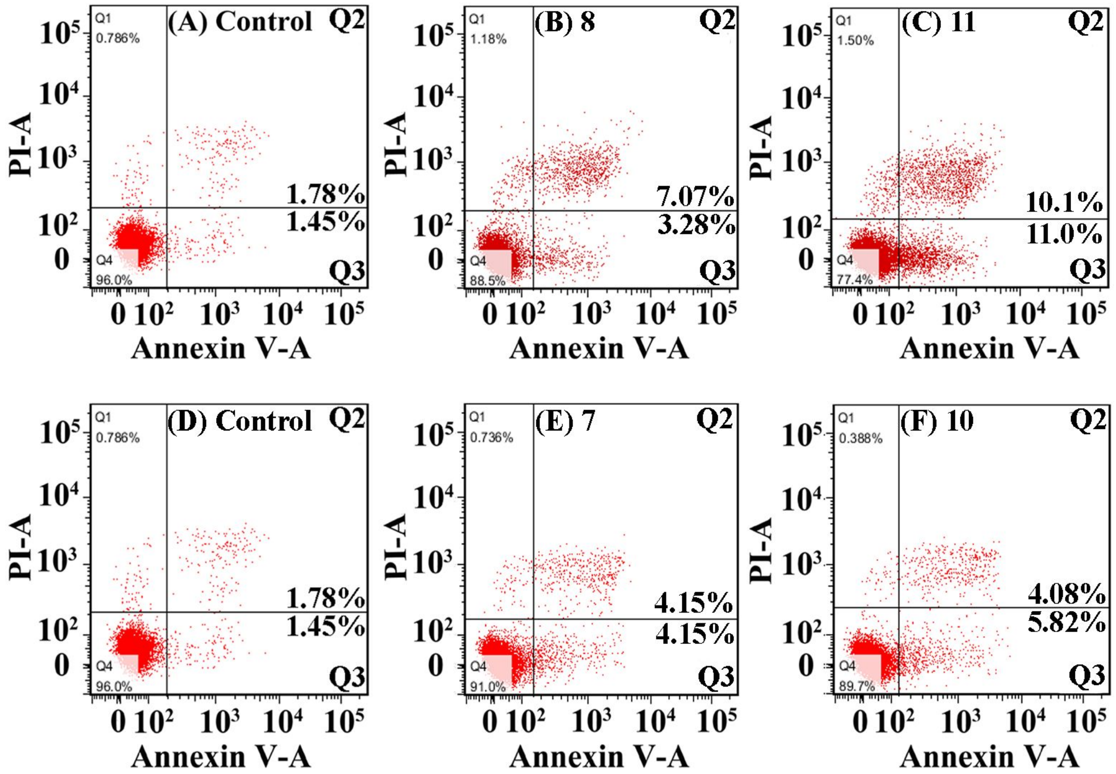


**Figure S93.** Apoptosis of Hep-G2 cells treated with complexes **7** (16 $\mu$M), **8** (10 $\mu$M), **10** (12 $\mu$M), and **11** (6 $\mu$M) for 24 h, comparing with the control cells.

**Table S11.** Changes in relative expression for tumor metastasis genes in the Hep-G2 cells after treated with **3** (5 $\mu$M) for 24 h. The table lists genes that exhibit a difference in expression in the Hep-G2 cells sample when compared to control. The raw threshold cycle (Ct) values seen in the samples are also listed for comparison.

| Gene | Fold Change | Fold Up- or Down-Regulation | p value | Comments |
|---|---|---|---|---|
| ABL1 | 1.04 | **-2.06** | **0.000106** | OKAY |
| ACD | **0.48** | -1.11 | 0.592598 | OKAY |
| AKT1 | 0.90 | **-2.54** | **0.001588** | OKAY |
| ATM | **0.39** | **-2.85** | **0.001258** | OKAY |
| ATP5C1 | **0.35** | 1.09 | 0.531206 | OKAY |
| BCL2 | 1.09 | **-2.03** | **0.000328** | OKAY |
| BLM | **0.49** | **-2.11** | **0.000220** | OKAY |
| CDK2 | **0.47** | **-2.35** | **0.000509** | OKAY |
| CHEK1 | **0.43** | -1.43 | **0.032170** | OKAY |
| CHEK2 | 0.70 | **-2.71** | **0.000669** | OKAY |
| DCLRE1B | **0.37** | 1.60 | **0.019424** | OKAY |
| DCLRE1C | 1.60 | 1.26 | 0.148415 | OKAY |
| DKC1 | 1.26 | -1.34 | 0.118258 | OKAY |
| EGF | 0.75 | **-6.30** | **0.003193** | A |
| EME1 | **0.16** | **-3.05** | **0.000599** | OKAY |
| ERCC1 | **0.33** | 1.25 | **0.025240** | OKAY |
| ERCC4 | 1.25 | -1.45 | **0.014919** | OKAY |
| GAR1 | 0.69 | -1.21 | **0.013440** | OKAY |
| HAT1 | 0.82 | -1.37 | **0.049188** | OKAY |
| HNRNPA2B1 | 0.73 | -1.31 | 0.326764 | OKAY |
| HNRNPD | 0.76 | -1.37 | **0.000813** | OKAY |
| HSP90AA1 | 0.73 | 1.73 | **0.000383** | OKAY |
| HSPA1L | 1.73 | **22.01** | **0.000186** | OKAY |
| IGF1 | **22.01** | **2.16** | 0.096890 | B |
| KRAS | **2.16** | **-1.96** | **0.005396** | OKAY |
| KRIT1 | 0.51 | -1.26 | **0.000206** | OKAY |
| MEN1 | 0.79 | **-2.80** | **0.000037** | OKAY |
| MRE11A | **0.36** | **-2.00** | **0.000355** | OKAY |
| MSH2 | 0.50 | -1.63 | **0.000863** | OKAY |
| MSH3 | 0.61 | **-2.03** | **0.000605** | OKAY |
| MUS81 | **0.49** | 1.05 | 0.222393 | OKAY |
| MYC | 1.05 | -1.40 | **0.008494** | OKAY |
| NBN | 0.72 | **-2.86** | **0.007132** | OKAY |
| NCL | **0.35** | -1.01 | 0.835997 | OKAY |
| NHP2 | 0.99 | 1.35 | 0.074933 | OKAY |
| NOP10 | 1.35 | 1.05 | 0.680931 | OKAY |
| OBFC1 | 1.05 | -1.09 | 0.166839 | OKAY |
| PARP1 | 0.91 | **-2.09** | **0.000785** | OKAY |
| PAX8 | **0.48** | **2.28** | 0.141213 | B |
| PIF1 | **2.28** | **-13.13** | **0.000022** | OKAY |
| PINX1 | **0.08** | 1.37 | **0.000100** | OKAY |
| PLK1 | 1.37 | **-5.77** | **0.000002** | OKAY |
| POT1 | **0.17** | **-2.15** | **0.000078** | OKAY |
| PPARG | **0.47** | -1.61 | **0.004668** | OKAY |

| | | | | |
|---|---|---|---|---|
| PPP2R1A | 0.62 | -1.60 | **0.001826** | OKAY |
| PPP2R1B | 0.63 | **-3.39** | **0.000375** | OKAY |
| PRKCA | **0.29** | **-1.86** | **0.021859** | OKAY |
| PRKCB | 0.54 | **-2.33** | **0.007050** | OKAY |
| PRKDC | **0.43** | **-2.63** | **0.003003** | OKAY |
| PTGES3 | **0.38** | -1.69 | **0.000506** | OKAY |
| PURA | 0.59 | -1.31 | **0.023329** | OKAY |
| RAD17 | 0.76 | **-2.08** | **0.000138** | OKAY |
| RAD50 | **0.48** | 1.42 | **0.027566** | OKAY |
| RAP1A | 1.42 | -1.73 | **0.002700** | OKAY |
| RAPGEF1 | 0.58 | **-2.64** | **0.000001** | OKAY |
| RASSF1 | **0.38** | **-1.84** | 0.079601 | OKAY |
| RB1 | 0.54 | **-2.78** | **0.000677** | OKAY |
| RFC1 | **0.36** | -1.41 | 0.075036 | OKAY |
| RIF1 | 0.71 | 1.34 | 0.221779 | OKAY |
| RTEL1 | 1.34 | **-1.96** | **0.049130** | OKAY |
| SART1 | 0.51 | 1.60 | **0.001759** | OKAY |
| SIRT2 | 1.60 | 1.07 | 0.653316 | OKAY |
| SIRT6 | 1.07 | **2.16** | **0.000233** | OKAY |
| SLX4 | **2.16** | **-4.58** | **0.000045** | OKAY |
| SMAD3 | **0.22** | **-5.27** | **0.000166** | OKAY |
| SMG6 | **0.19** | **-2.95** | **0.000274** | OKAY |
| SP1 | **0.34** | **-2.01** | **0.000304** | OKAY |
| SSB | **0.50** | 1.12 | **0.048193** | OKAY |
| SUN1 | 1.12 | **-2.86** | **0.000041** | OKAY |
| TEP1 | **0.35** | **-2.65** | **0.000405** | OKAY |
| TERF1 | **0.38** | **2.93** | **0.000222** | OKAY |
| TERF2 | **2.93** | 1.39 | 0.137657 | OKAY |
| TERF2IP | 1.39 | **2.58** | **0.000036** | OKAY |
| TERT | **2.58** | 1.70 | **0.000128** | C |
| TGFB1 | 1.70 | -1.29 | **0.000090** | OKAY |
| TINF2 | 0.77 | -1.01 | 0.897145 | OKAY |
| TNKS | 0.99 | -1.46 | **0.000704** | OKAY |
| TNKS2 | 0.68 | 1.21 | **0.042412** | OKAY |
| TP53 | 1.21 | -1.31 | 0.163029 | OKAY |
| TP53BP1 | 0.76 | **-1.89** | **0.000723** | OKAY |
| TPP1 | 0.53 | -1.19 | **0.039131** | OKAY |
| WRAP53 | 0.84 | 1.01 | 0.988918 | OKAY |
| XRCC5 | 1.01 | **-2.09** | **0.025597** | OKAY |
| XRCC6 | **0.48** | 1.59 | 0.141964 | OKAY |
| ACTB | 1.59 | -1.11 | 0.069097 | OKAY |
| B2M | 0.90 | 1.10 | 0.446184 | OKAY |
| GAPDH | 1.10 | 1.16 | 0.158508 | OKAY |
| HPRT1 | 1.16 | -1.20 | **0.007448** | OKAY |
| RPLP0 | 0.84 | 1.04 | 0.483564 | OKAY |

**Legend: Fold-Change** ($2^{-\text{Delta Delta Ct}}$) is the normalized gene expression ($2^{-\text{Delta Ct}}$) in the Test Sample divided the normalized gene expression ($2^{-\text{Delta Ct}}$) in the Control Sample.

**Fold-Regulation** represents fold-change results in a biologically meaningful way. Fold-change values greater than one indicate a positive- or an up-regulation, and the fold-regulation is equal to the fold-change

Fold-change values less than one indicate a negative or down-regulation, and the fold-regulation is the negative inverse of the fold-change.

Fold-change and fold-regulation values greater than 2 are indicated in red; fold-change values less than 0.5 and fold-regulation values less than -2 are indicated in blue.

**p-values:** The p values are calculated based on a Student's t-test of the replicate $2^{-\text{Delta Ct}}$ values for each gene in the control group and treatment groups, and p values less than 0.05 are indicated in red.

**Comments:** A: This gene's average threshold cycle is relatively high (>30) in either the control or the test sample, and is reasonably low in the other sample (<30).

These data mean that the gene's expression is relatively low in one sample and reasonably detected in the other sample suggesting that the actual fold-change value is at least as large as the calculated and reported fold-change result. This fold-change result may also have greater variations if p value >0.05; therefore, it is important to have a sufficient number of biological replicates to validate the result for this gene. B: This gene's average threshold cycle is relatively high (>30), meaning that its relative expression level is low, in both control and test samples, and the p-value for the fold-change is either unavailable or relatively high (p>0.05). This fold-change result may also have greater variations; therefore, it is important to have a sufficient number of biological replicates to validate the result for this gene.

C: This gene's average threshold cycle is either not determined or greater than the defined cut-off (deault 35), in both samples meaning that its expression was undetected, making this fold-change result erroneous and un-interpretable.

**Table S12.** Changes in relative expression for tumor metastasis genes in the Hep-G2 cells after treated with **6** (14 $\mu$M) for 24 h. The table lists genes that exhibit a difference in expression in the Hep-G2 cells sample when compared to control. The raw threshold cycle (Ct) values seen in the samples are also listed for comparison.

| Gene | Fold Change | p value | Fold Up- or Down-Regulation | Comments |
|---|---|---|---|---|
| ABL1 | 1.26 | 0.009096 | 1.26 | OKAY |
| ACD | 2.52 | 0.007837 | 2.52 | OKAY |
| AKT1 | 0.82 | 0.037982 | -1.22 | OKAY |
| ATM | 1.21 | 0.213791 | 1.21 | OKAY |
| ATP5C1 | 1.18 | 0.311845 | 1.18 | OKAY |
| BCL2 | 2.17 | 0.000693 | 2.17 | OKAY |
| BLM | 0.92 | 0.348503 | -1.08 | OKAY |
| CDK2 | 0.69 | 0.000550 | -1.46 | OKAY |
| CHEK1 | 0.99 | 0.913998 | -1.01 | OKAY |
| CHEK2 | 0.77 | 0.018257 | -1.30 | OKAY |

| | | | | |
|---|---|---|---|---|
| DCLRE1B | 0.79 | 0.099675 | -1.26 | OKAY |
| DCLRE1C | 1.53 | 0.010419 | 1.53 | OKAY |
| DKC1 | 1.41 | 0.008022 | 1.41 | OKAY |
| EGF | 1.51 | 0.018683 | 1.51 | OKAY |
| EME1 | 1.34 | 0.000183 | 1.34 | OKAY |
| ERCC1 | 2.03 | 0.000083 | 2.03 | OKAY |
| ERCC4 | 1.24 | 0.000178 | 1.24 | OKAY |
| GAR1 | 1.22 | 0.006819 | 1.22 | OKAY |
| HAT1 | 1.20 | 0.052824 | 1.20 | OKAY |
| HNRNPA2B1 | 1.21 | 0.021847 | 1.21 | OKAY |
| HNRNPD | 1.08 | 0.083753 | 1.08 | OKAY |
| HSP90AA1 | 0.70 | 0.126243 | -1.43 | OKAY |
| HSPA1L | 1.11 | 0.566742 | 1.11 | OKAY |
| IGF1 | 1.40 | 0.358580 | 1.40 | B |
| KRAS | 0.82 | 0.036939 | -1.22 | OKAY |
| KRIT1 | 1.38 | 0.012566 | 1.38 | OKAY |
| MEN1 | 1.38 | 0.000218 | 1.38 | OKAY |
| MRE11A | 1.19 | 0.060253 | 1.19 | OKAY |
| MSH2 | 1.10 | 0.236094 | 1.10 | OKAY |
| MSH3 | 1.44 | 0.000641 | 1.44 | OKAY |
| MUS81 | 2.87 | 0.000221 | 2.87 | OKAY |
| MYC | 0.70 | 0.000541 | -1.43 | OKAY |
| NBN | 1.32 | 0.002350 | 1.32 | OKAY |
| NCL | 0.81 | 0.005195 | -1.23 | OKAY |
| NHP2 | 1.37 | 0.094085 | 1.37 | OKAY |
| NOP10 | 0.93 | 0.434078 | -1.08 | OKAY |
| OBFC1 | 1.48 | 0.026051 | 1.48 | OKAY |
| PARP1 | 0.85 | 0.011109 | -1.18 | OKAY |
| PAX8 | 1.27 | 0.723984 | 1.27 | B |
| PIF1 | 0.92 | 0.139792 | -1.09 | OKAY |
| PINX1 | 2.38 | 0.001509 | 2.38 | OKAY |
| PLK1 | 0.78 | 0.005470 | -1.29 | OKAY |
| POT1 | 1.30 | 0.053576 | 1.30 | OKAY |
| PPARG | 2.02 | 0.000039 | 2.02 | OKAY |
| PPP2R1A | 1.28 | 0.010078 | 1.28 | OKAY |
| PPP2R1B | 0.53 | 0.000236 | -1.87 | OKAY |
| PRKCA | 0.89 | 0.033451 | -1.12 | OKAY |
| PRKCB | 1.42 | 0.456864 | 1.42 | B |
| PRKDC | 1.15 | 0.158060 | 1.15 | OKAY |
| PTGES3 | 0.87 | 0.173152 | -1.14 | OKAY |
| PURA | 6.78 | 0.005815 | 6.78 | OKAY |
| RAD17 | 1.63 | 0.003590 | 1.63 | OKAY |

| | | | | |
|---|---|---|---|---|
| RAD50 | 3.91 | 0.001826 | 3.91 | OKAY |
| RAP1A | 1.37 | 0.021408 | 1.37 | OKAY |
| RAPGEF1 | 1.09 | 0.107023 | 1.09 | OKAY |
| RASSF1 | 1.13 | 0.223041 | 1.13 | OKAY |
| RB1 | 0.89 | 0.013352 | -1.12 | OKAY |
| RFC1 | 1.62 | 0.001540 | 1.62 | OKAY |
| RIF1 | 1.22 | 0.113565 | 1.22 | OKAY |
| RTEL1 | 1.19 | 0.511687 | 1.19 | OKAY |
| SART1 | 1.62 | 0.021482 | 1.62 | OKAY |
| SIRT2 | 1.31 | 0.031627 | 1.31 | OKAY |
| SIRT6 | 1.98 | 0.000329 | 1.98 | OKAY |
| SLX4 | 1.19 | 0.272236 | 1.19 | OKAY |
| SMAD3 | 0.83 | 0.069652 | -1.20 | OKAY |
| SMG6 | 1.19 | 0.025579 | 1.19 | OKAY |
| SP1 | 1.29 | 0.342795 | 1.29 | OKAY |
| SSB | 1.52 | 0.010236 | 1.52 | OKAY |
| SUN1 | 0.84 | 0.028003 | -1.19 | OKAY |
| TEP1 | 1.42 | 0.009199 | 1.42 | OKAY |
| TERF1 | 1.78 | 0.000232 | 1.78 | OKAY |
| TERF2 | 0.88 | 0.193555 | -1.14 | OKAY |
| TERF2IP | 1.36 | 0.054101 | 1.36 | OKAY |
| TERT | 1.25 | 0.000971 | 1.25 | C |
| TGFB1 | 0.90 | 0.111569 | -1.12 | OKAY |
| TINF2 | 1.34 | 0.013902 | 1.34 | OKAY |
| TNKS | 1.30 | 0.000878 | 1.30 | OKAY |
| TNKS2 | 0.79 | 0.247605 | -1.27 | OKAY |
| TP53 | 2.45 | 0.000260 | 2.45 | OKAY |
| TP53BP1 | 1.28 | 0.001874 | 1.28 | OKAY |
| TPP1 | 1.27 | 0.074605 | 1.27 | OKAY |
| WRAP53 | 1.14 | 0.718285 | 1.14 | OKAY |
| XRCC5 | 0.90 | 0.535556 | -1.11 | OKAY |
| XRCC6 | 1.21 | 0.112237 | 1.21 | OKAY |
| ACTB | 0.77 | 0.000752 | -1.30 | OKAY |
| B2M | 1.34 | 0.009505 | 1.34 | OKAY |
| GAPDH | 1.12 | 0.018435 | 1.12 | OKAY |
| HPRT1 | 0.71 | 0.000911 | -1.41 | OKAY |
| RPLP0 | 1.23 | 0.002680 | 1.23 | OKAY |

**Legend: Fold-Change** ($2^{-\text{Delta Delta Ct}}$) is the normalized gene expression ($2^{-\text{Delta Ct}}$) in the Test Sample divided the normalized gene expression ($2^{-\text{Delta Ct}}$) in the Control Sample.

**Fold-Regulation** represents fold-change results in a biologically meaningful way. Fold-change values greater than one indicate a positive- or an up-regulation, and the fold-regulation is equal to the fold-change

Fold-change values less than one indicate a negative or down-regulation, and the fold-regulation is the negative inverse of the fold-change.

Fold-change and fold-regulation values greater than 2 are indicated in red; fold-change values less than 0.5 and fold-regulation values less than -2 are indicated in blue.

**p-values:** The p values are calculated based on a Student's t-test of the replicate $2^{-Delta\ Ct}$ values for each gene in the control group and treatment groups, and p values less than 0.05 are indicated in red.

**Comments:** A: This gene's average threshold cycle is relatively high (>30) in either the control or the test sample, and is reasonably low in the other sample (<30).

These data mean that the gene's expression is relatively low in one sample and reasonably detected in the other sample suggesting that the actual fold-change value is at least as large as the calculated and reported fold-change result. This fold-change result may also have greater variations if p value >0.05; therefore, it is important to have a sufficient number of biological replicates to validate the result for this gene. B: This gene's average threshold cycle is relatively high (>30), meaning that its relative expression level is low, in both control and test samples, and the p-value for the fold-change is either unavailable or relatively high (p>0.05). This fold-change result may also have greater variations; therefore, it is important to have a sufficient number of biological replicates to validate the result for this gene.

C: This gene's average threshold cycle is either not determined or greater than the defined cut-off (deault 35), in both samples meaning that its expression was undetected, making this fold-change result erroneous and un-interpretable.

***In Vivo* Anticancer Activity toward BEL-7402 Xenograft Tumor.**

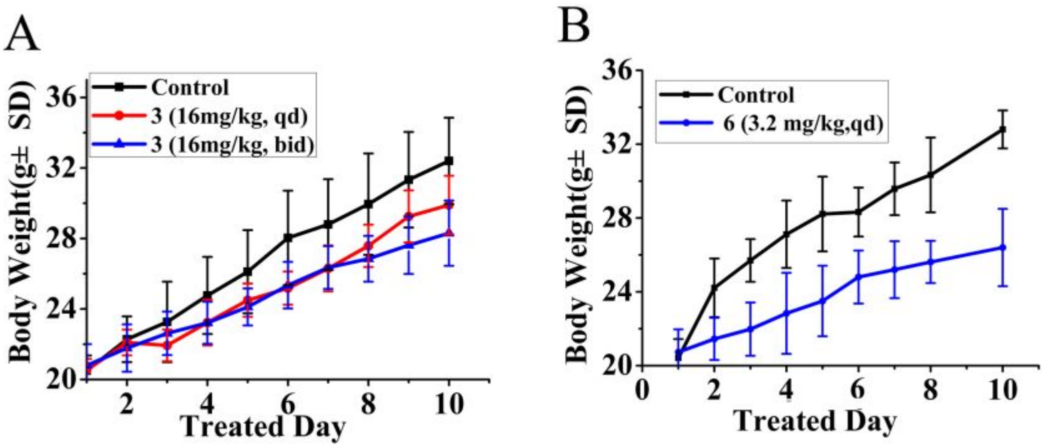


**Figure S94.** The average body weight of KM mice in treated with complex **3** and **6**.

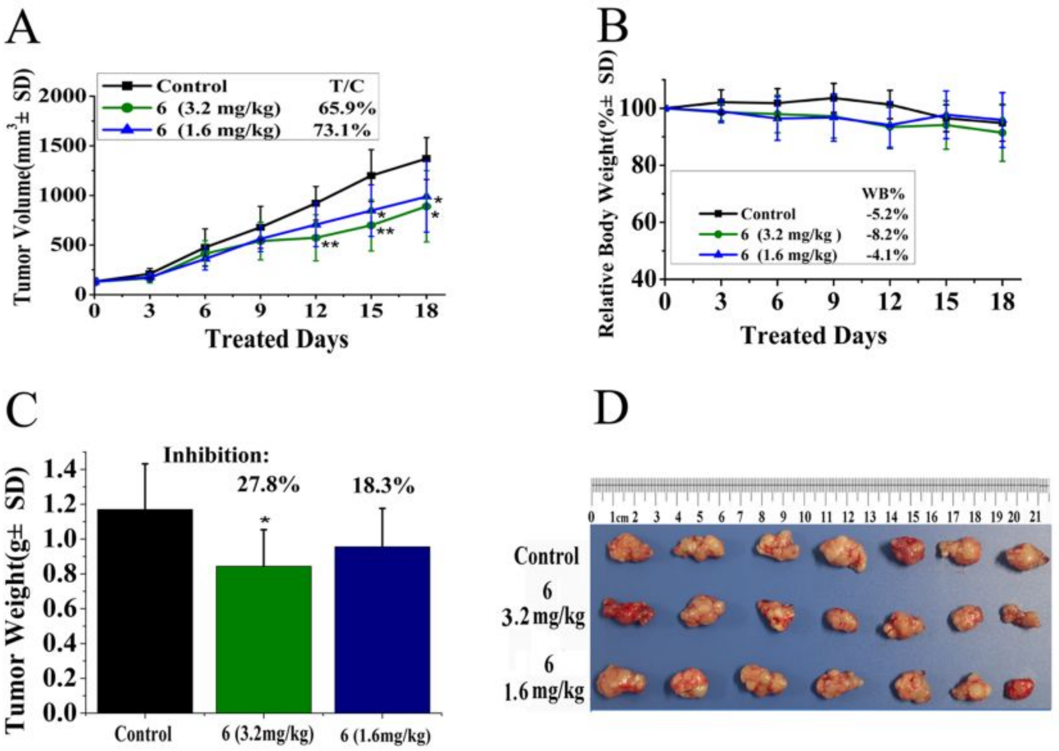


**Figure S95.** *In vivo* anticancer activity of **6** in mice bearing BEL-7402 tumor xenograft. (A) Effect of **6** (3.2, 1.6 mg/kg/qd), cisplatin (2 mg/kg/q2d), or vehicle (5%DMSO in saline, v/v) on growth of tumor xenograft. Tumor growth is tracked by the mean tumor volume ($mm^3$) ± SD (n=7) and calculated as the relative tumor increment rate (T/C, %), (**) $P$<0.01, (*) $P$<0.05, *p vs* vehicle control. (B) Body weight change (presented as % change from initial weight). (C) Tumor weight was recorded after the mice were killed.(*) $P$<0.05, *p vs* vehicle control. (D) Photographs of tumor from treatment groups and control group.

**Table S13**. The tumor volume in **3**, **6**-treated and non-treated mice from the date of surgery to the study end point in the BEL-7402 xenograft model.

| **Group** | **Tumor Volume ($mm^3$)** (start) | (end) | **T/C (%)** |
|---|---|---|---|
| Control | 103.64±22.18 | 1842.75±539.14 | - |
| Complex **3** (8 mg/kg) | 103.53±31.26 | 873.15±374.22 | 48.9[b] |
| Complex **3** (16 mg/kg) | 105.73±25.03 | 705.81±203.92 | 37.5[a] |
| Cisplatin (2 mg/kg) | 104.79±32.12 | 566.82±331.08 | 30.7[a] |
| Control | 131.92±38.73 | 1371.21±210.89 | |
| Complex **6** (1.6 mg/kg) | 129.93±37.71 | 987.92±357.66 | 65.9[b] |
| Complex **6** (3.2 mg/kg) | 129.89±30.84 | 889.69±359.98 | 73.1[b] |

a mean $p$<0.01, b mean $p$<0.05, *p vs* vehicle control

**Table S14.** Average body weight in **3**, **6**-treated and non-treated mice from the date of surgery to the study end point in the BEL-7402 xenogfart model.

| Group | Body Weight (g) (start) | Body Weight (g) (end) | RBW (%) (end) |
|---|---|---|---|
| Control | 22.6±0.7 | 23.7±1.3 | 104.6±7.1 |
| Complex **3** (8mg/kg) | 22.4±1.3 | 21.4±1.1 | 94.91±6.7 |
| Complex **3** (16mg/kg) | 22.3±1.2 | 21.1±1.5 | 95.68±3.6 |
| Cisplatin (2mg/kg) | 22.5±1.1 | 19.6±0.9 | 87.11±4.8 |
| Control | 22.7±1.6 | 21.5±1.4 | 94.8±6.4 |
| Complex **6** (1.6mg/kg) | 22.7±1.4 | 21.8±1.5 | 95.9±9.6 |
| Complex **6** (3.2 mg/kg) | 23.1±1.9 | 21.1±1.2 | 91.4±10 |

**Table S15.** In Vivo Anticancer Activity of **3** and **6** toward Human Hepatoma (BEL-7402) Tumor Xenograft.

| Group | average tumor weight(mean ± SD g) | inhibition of tumor growth(%) |
|---|---|---|
| Control | 2.16±0.68 | - |
| Complex **3** ( 8mg/kg) | 1.06±0.64 | 51.2[b] |
| Complex **3** ( 16mg/kg) | 0.8±0.34 | 63.1[a] |
| Cisplatin ( 2 mg/kg) | 0.52±0.29 | 75.9[a] |
| Control | 1.17±0.26 | - |
| Complex **6** (1.6mg/kg) | 0.96±0.22 | 18.3 |
| Complex **6** ( 3.2 mg/kg) | 0.84±0.21 | 27.8[b] |

a mean $p$<0.01, b mean $p$<0.05, $p$ *vs* control.

## ■ Experimental Section

**Synthesis and Characterization of Ligands**. Synthesis and characterization of 6-amino-oxoisoaporphine ($L^a$), 8-amino-oxoisoaporphine ($L^b$), 8-chloro-oxoisoaporphine ($L^c$) and 10-chloro-11-amino-oxoisoaporphine ($L^d$) have been reported,[1-5] very similar to the synthesis of 1-azabenzanthrone or 6-hydroxyloxoisoaporphine alkaloid scaffolds.[5]

**Synthesis of 6-amino-oxoisoaporphine ($L^a$)**. 6-amino-oxoisoaporphine ($L^a$) was synthesized by using modified method reported by Chen and Tang.[1-5] A mixture of 4-chloro phenylethylamine (74.0 g, 0.47 mol) and phthalic anhydride (70.0 g, 0.47 mol) in methylbenzene (500.0 mL) was refluxed for 6.0 h. After cooling to 0−5 °C, crystals of 4-chlorophenylethylphthalimide (98.8 g, 73.2%) were separated. These crystals were filtered and washed with methylbenzene (100.0 mL). The product was pure enough for the next reaction.

To a mixture of sodium chloride (9.23 g, 0.16 mol) and anhydrous aluminium chloride (105.0 g, 0.80 mol) were slowly added 4-chlorophenylethylphthalimide (99.0 g, 0.35 mol) at 180.0 °C for 3.0 h. Finally, the reaction mixture was cooled down to 37.0 °C , and the product was separated by filtration, finely ground and poured slowly into concentrated sulfuric acid (300.0 mL) at 140.0 °C. The mixture was stirred and heated at 230.0 °C for 3.0 h. After being cooled, the solution was poured onto ice (800.0 g). Sodium hydroxide was added until pH 3−4 was obtained and the resultant precipitate was filtered off and washed successively with dilute aqueous sodium hydroxide and water to give the crude product 6-chloro-oxoisoaporphine, which was extracted with acetic acid.

The extract was concentrated under reduced pressure and the resultant precipitate of 6-chloro-oxoisoaporphine was washed, dried, and sublimed at 140.0 ° C under a

pressure of 1 mm mercury to give the 6-chloro-oxoisoaporphine (27.7 g, 30.0%) as light yellow solid.

The reaction kettle was added to 6-chloro-oxoisoaporphine (0.2655 g) and concentrated ammonium hydroxide (50.0 mL), and the mixture was stirred at 185 °C for 24.0 h, it was cooled to room temperature, filtered, and washed with the ultra pure water. The crude solid of 6-amino-oxoisoaporphine was purified by column chromatography using $CH_3Cl$/petroleum ether (1:3) as elution to afford a solid 6-amino-oxoisoaporphine ($L^a$). Yield (0.1230 g, 50.0%). $^1$H NMR (500 MHz, DMSO-$d_6$): δ 7.08 (d，1H，J=9.5 Hz), 7.87 – 7.90 (m，1H)，7.98 (t，1H，*J*=7.1 Hz)，8.06 (d，1H，*J*=9.5 Hz)，8.73 (d，1H，*J*=8.2 Hz)，8.78 (d，1H，*J*=5.0 Hz)，9.21 (d，1H，*J*=8.1Hz )，9.52 (s，Ar–NH，1H)，12.43 (s，Ar–NH，1H). ESI-MS m/z: 247.1 $[M+H]^+$. Elemental analysis calcd (%) for $C_{16}H_{10}N_2O$: C 78.03, H 4.09, N 11.38; found: C 78.01, H 4.14, N 11.42.

**Synthesis of 8-amino-oxoisoaporphine ($L^b$) and 8-chloro-oxoisoaporphine ($L^c$).** Replacing 4-chloro phenylethylamine and phthalic anhydride with phenylethylamine and 3-chloro-phthalic anhydride with the procedure for 6-amino-oxoisoaporphine ($L^a$) gave rise to 8-amino-oxoisoaporphine ($L^b$) and 8-chloro-oxoisoaporphine ($L^c$).

Data for 8-amino-oxoisoaporphine ($L^b$): The brown color product suitable for structural characterization. Yield (0.0984 g, 40.0%). $^1$H NMR (600 MHz, DMSO-$d_6$) δ 8.71 (d, *J* = 5.5 Hz, 1H), 8.43 (dd, *J* = 7.2, 0.8 Hz, 1H), 8.27 (d, *J* = 7.6 Hz, 1H), 8.03 (dd, *J* = 7.4, 0.9 Hz, 1H), 7.98 (dd, *J* = 8.0, 7.4 Hz, 1H), 7.92 (d, *J* = 5.5 Hz, 1H), 7.54 – 7.43 (m, 1H), 6.98 (dd, *J* = 8.4, 0.9 Hz, 1H). $^{13}$C NMR (151 MHz, DMSO-$d_6$) δ 184.12, 153.17, 148.74, 144.08, 136.83, 134.99, 134.94, 132.46, 131.05, 129.82, 128.26, 122.17, 120.70, 119.09, 112.59, 112.55. ESI-MS m/z: 247.1 $[M+H]^+$; IR

(KBr): 3853, 3744, 3421, 3304, 3047, 2936, 1932, 1731, 1600, 1538, 1447, 1405, 1324, 1278, 1205, 1167, 1034, 934, 853, 819, 706, 606, 541, 474 $cm^{-1}$. Elemental analysis calcd (%) for $C_{16}H_{10}N_2O$: C 78.03, H 6.09, N 11.38; found: C 77.96, H 6.14, N 12.42.

Data for 8-chloro-oxoisoaporphine ($L^c$): The yellow color product (8-chloro-oxoisoaporphine ($L^c$)) suitable for structural characterization. Yield (0.0191 g, 72.0%). $^1H$ NMR (600 MHz, DMSO-*d*6) δ 8.74 (s, 1H), 8.67 (s, 1H), 8.34 (s, 1H), 8.28 (s, 1H), 7.92 (s, 2H), 7.69 (s, 1H), 7.64 (s, 1H). $^{13}C$ NMR (151 MHz, DMSO-*d*6) δ 181.57, 147.24, 144.59, 139.50, 135.07, 134.69, 134.60, 134.08, 131.80, 130.41, 129.41, 128.39, 125.11, 122.25, 122.02. ESI-MS m/z: 443.9 $[M+Cl+DMSO+2CH_3OH]^-$; IR (KBr): 3852, 3741, 3304, 3056, 1972, 1660, 1613, 1581, 1495, 1442, 1392, 1330, 1291, 1263, 1207, 1166, 1076, 1029, 953, 904, 856, 810, 737, 697, 536, 464, 416 $cm^{-1}$. Elemental analysis calcd (%) for $C_{16}H_8ClNO$: C 72.33, H 3.03, N 5.27; found: C 72.29, H 3.10, N 5.21.

**Synthesis of 10-chloro-11-amino-oxoisoaporphine ($L^d$).** 10-chloro-11-amino-oxoisoaporphine ($L^d$) was synthesized by using modified method reported by Chen and Tang.[1-7] A mixture of phenylethylamine (45.0 g, 0.1 mol) and 3-chloro phthalic anhydride (57.0 g, 0.1 mol) in methylbenzene (500.0 mL) was refluxed for 6.0 h. After cooling to 0 – 5 ° C, crystals of 4-chloro-2-(2-phenylethyl)-1*H*-isoindole-1,3(2*H*)-dione (27.94 g, 98.0%) were separated. These crystals were filtered and washed with methylbenzene (100 mL). The product was pure enough for the next reaction.

To a mixture of sodium chloride (4.67 g, 0.08 mol) and anhydrous aluminium chloride (53.0 g, 0.4 mol) was slowly added 4-chloro-2-(2-phenylethyl)-1*H*-isoindole-1,3(2*H*)-dione at 140 ° C for 30 min. The

reaction was allowed to continue at 220.0 ℃ for 3.0 h. Finally, the reaction mixture was cooled down to 37.0 °C , and the product was separated by filtration, finely ground and poured slowly into concentrated sulfuric acid (600.0 mL) at 80.0 °C. The mixture was stirred and heated at 230.0 °C for 3.0 h. After being cooled, the solution was poured onto ice (600.0 g). Sodium hydroxide was added until pH 2 – 3 was obtained and the resultant precipitate was filtered off and washed successively with dilute aqueous sodium hydroxide and water to give the crude product 10-chloro-oxoisoaporphine, which was extracted with acetic acid.

The extract was concentrated under reduced pressure and the resultant precipitate of 10-chloro-oxoisoaporphine was washed, dried, and sublimed at 140.0 °C under a pressure of 1 mm mercury by petroleum ether/ethyl acetate (30:1) to give the 10-chloro-oxoisoaporphine (35.0 %) as light yellow solid.

3.0 mL of 62 % nitric acid was added to a mixture of concentrated sulfuric acid (6.0 mL) and 10-chloro-oxoisoaporphine (2.0 g, 7.5 mmol) in a round bottom flask (50 mL), and the mixture was stirred at 50 °C for 4.0 h. After being cooled, the solution was poured into ice (150.0 g). Ammonia was added until pH = 8-9 was obtained and the precipitate was filtered off and washed with water to give the crude product. Recrystallization from benzene afforded 10-chloro-11-nitro-oxoisoaporphine (1.99 g, 85.0%) as yellow solid.

To a stirred suspension of 10-chloro-11-nitro-oxoisoaporphine (1.99 g, 6.4 mmol) in ethanol (140.0 mL) was added a solution of sodium sulfide nonahydrate (6.7 g) and sodium hydroxide (2.6 g) in water (60.0 mL). The mixture was heated at reflux for 4.5 h and left to stand overnight. The ethanol was removed in vacuo and the residue cooled to 0 – 5 °C. The resulting precipitate was collected by filtration, washed with water, and dried. The crude solid of 10-chloro-11-amino-oxoisoaporphine was

purified by column chromatography using petroleum ether/ethyl acetate (20:1) as elution to afford a solid 10-chloro-11-amino-oxoisoaporphine ($L^d$). Yield (0.6826 g, 38.0%). $^1$H NMR (600 MHz, DMSO-$d_6$) δ 8.80 (d, $J$ = 5.4 Hz, 1H), 8.45 (d, $J$ = 7.2 Hz, 1H), 8.32 (d, $J$ = 8.1 Hz, 1H), 8.01 – 7.98 (m, 1H), 7.96 (d, $J$ = 5.5 Hz, 1H), 7.53 (d, $J$ = 9.1 Hz, 1H), 7.01 (d, $J$ = 9.1 Hz, 1H). $^{13}$C NMR (151 MHz, DMSO-$d_6$) δ 183.98, 153.26, 148.75, 143.28, 139.99, 135.07, 133.42, 132.20, 131.25, 129.36, 128.57, 122.93, 121.20, 120.82, 118.82, 114.00. ESI-MS m/z: 281.0 $[M+H]^+$; IR (KBr): 3853, 3746, 3423, 3294, 3054, 2926, 1916, 1605, 1538, 1445, 1384, 1316, 1264, 1122, 1081, 967, 856, 823, 751, 718, 504 cm$^{-1}$. Elemental analysis calcd (%) for $C_{16}H_9ClN_2O$: C 68.46, H 3.23, N 9.98; found: C 68.50, H 3.18, N 10.02.

**The interaction of complex with Pu27 G4 determined by ESI-MS.** Each complex (100.0 μM) reacted with Pu27 G4 DNA (final concentration of Pu27 G-quadruplex, 2000.0 μM) in 10 mM pH 7.4 TBS (containing 1.0 mM EDTA) at room temperature for 24.0 h (DMSO < 1%), in which the ratio of complex: G4-Pu27 DNA was 1:20.[9–12] Then the ESI-MS spectra of the reacting solution were obtained on Thermofisher Scientific Exactive LC-MS spectrometer (MeCN:$H_2O$ = 90:10).[5,8,9–12]

**Uptake of metal (Pt, Ni and Pd) in Hep-G2 cells.** Hep-G2 cells (~10 million cells) were treated with complexes **1** (8 $\mu$M), **2** (15 $\mu$M), **3** (5 $\mu$M), **4** (18 $\mu$M), cisplatin (10 $\mu$M), **5** (28 $\mu$M), **6** (14 $\mu$M), **7** (16 $\mu$M), **8** (10 $\mu$M), **10** (12 $\mu$M) and **11** (6 $\mu$M) for 8.0 h at 37 °C in a humidified 5% $CO_2$ incubator. The spent media was removed, and the Hep-G2 cells were washed with 5.0 mL of PBS, scraped, and collected in 5.0 mL of PBS. The scrapped cells were spun down, by centrifuging at 2500 rpm for 10.0 min. The cell pellet obtained was dissolved in 1.0 M NaOH (1.0 mL) and diluted with 2.0% (v/v) $HNO_3$ (5.0 mL) for determining whole cell metal (Pt, Ni and/or Pd) content. Another set was treated similarly, nuclear fraction and

mitochondrial fraction were isolated as described by Schreiber et al.[13] And the final solution was made up to 5.0 mL using 2.0% (v/v) $HNO_3$. The amount of metal (Pt, Ni and/or Pd) taken up by the Hep-G2 cells was determined by ICP-MS. The instrument was calibrated for metal (Pt, Ni and/or Pd) using standard solutions containing 10, 50, 100, 500 and 1000 ppb each metal (Pt, Ni or Pd).

## ■ References

1 Tang, H., Wang, Z.-Y., Zhong, S.-M. & Qin, J.-K. Synthesis and antitumor activity of 6-substituted 1-azabenzanthrone. *J. Guangxi Normal University* **30**, 78−82 (2012).

2 Tang, H. 8-Substituted oxoisoaporphine derivatives as acetylcholinesterase inhibitor and their preparation. *Faming Zhuanli Shenqing* (2014), CN 103923009 A 20140716.

3 Tang, H. 11-substituted oxoisoaporphine derivative, its synthesis method and application. *Faming Zhuanli Shenqing* (2014), CN 103923010 A 20140716.

4 Tang, H. *et al.* Synthesis, biological evaluation and molecular modeling of oxoisoaporphine and oxoaporphine derivatives as new dual inhibitors of acetylcholinesterase/butyrylcholinesterase. *Eur. J. Med. Chem.* **44**, 2523−2532 (2009).

5 Chen, Z.-F. *et al.* Stabilization of G-quadruplex DNA, inhibition of telomerase activity and tumor cell apoptosis of organoplatinum(II) complexes with oxoisoaporphine. *J. Med. Chem.* **58**, 2159−2179 (2015).

6 Tang, H. *et al.* Oxoisoaporphine alkaloid derivatives: Synthesis, DNA binding affinity and cytotoxicity. *Eur. J. Med. Chem.* **43**, 973−980 (2008).

7 Tang, H. *et al.* Novel oxoisoaporphine-based inhibitors of acetyl- and butyrylcholinesterase and acetylcholinesterase-induced beta-amyloid aggregation. *Bioorg. Med. Chem. Lett.* **22**, 2257−2261 (2012).

8 Yuan, L. *et al.* Existence of G-quadruplex structures in promoter region of oncogenes confirmed bu G-quadruplex DNA cross-lingkong strategy. *Sci. Rep.* **3**, 1811 (2013).

9 Zhu, J. *et al.* DNA cross-linking patterns induced by an antitumor-active trnuclear platinum complexes and comparison with its dinuclear analogue. *Chem. –Eur. J.* **15**, 5245−5253 (2009).

10 Reed, J. E., Arnal, A. A., Neidle, S. & Vilar, R. Stabilization of G-quadruplex DNA and inhibition of telomerase activity by square-planar nickel(II) complexes. *J. Am. Chem. Soc.* **128**, 5992–5993 (2006).

11 Bruijnincx, P. C. A. & Sadler, P. J. New trends for metal complexes with anticancer activity. *Curr. Opin. Chem. Biol.* **12**, 197–206 (2008).

12 Lu, Y.-J. *et al.* Molecular engineering of thiazole orange dye: change of fluorescent signaling from universal to specific upon binding with nucleic acids in bioassay. *ACS Chem. Biol.* **11**, 1019–1029 (2016).

13 Schreiber, E., Matthias, P., Mueller, M. M. & Schaffner, W. Rapid detection of octamer binding proteins with 'mini-extracts' prepared from a small number of cells. *Nucleic Acids Res.* **17**, 6419 (1989).
